# Histamine H_1_- and H_4_-receptor expression in human colon-derived cell lines

**DOI:** 10.1007/s00210-023-02565-8

**Published:** 2023-06-10

**Authors:** Jasper Carsten Schrammel, Martin König, Miriam Frommer, Kaya Saskia Andersen, Marla Kirsten, Roland Seifert, Detlef Neumann, Bastian Schirmer

**Affiliations:** Institute of Pharmacology, Hannover Medical School, 30623 Hannover, Germany

**Keywords:** Colitis, Colorectal carcinoma, Histamine, PCR, Cell line

## Abstract

In previous studies, we demonstrated the involvement of H_4_R in inflammatory bowel disease (IBD) and IBD-associated colon cancer in mice and could ascribe H_4_R-mediated histamine function to colon epithelial cells. The transferability of obtained data to humans is however lacking. Functional expression of H_4_R on colon epithelial cells is a prerequisite to pursue the hypothesis of involvement of H_4_R in carcinogenesis. Thus, we here compared the expression of histamine receptor subtypes in a series of cell lines. Out of these, three colon-derived cell lines displaying different combinations of H_1_R and H_4_R expression were submitted to functional analyses. Human hematopoietic HMC-1, HL-60, and U937, lung-derived A549 and Calu-3, and colorectal LoVo, SW 480, Caco-2, HT-29, and HCT116 cells were included in the study. mRNA expression was quantified by RT-qPCR. For functional analyses, Caco-2, HT-29, and HCT116 cells were treated by incubation with 1 – 10 µM histamine in the presence or absence of selective histamine receptor antagonists. Calcium mobilization, cAMP accumulation, and cell proliferation were measured by fluorimetry, mass spectrometry, and real-time bioimpedance measurements, respectively. Histamine receptor expression was heterogeneous in the cell lines tested. In most cell lines, we detected H_1_R mRNA while H_4_R mRNAs were found only occasionally. The colon-derived epithelial cell lines LoVo, SW480, and HT-29 expressed H_1_R mRNA exclusively, while in HCT116 cells H_1_R and H_4_R mRNAs and in CaCo-2 H_2_R mRNA were detectable. Subsequent functional analyses in HT29, Caco-2, and HCT116 cells, however, indicated that only HT-29 responded to histamine stimulation, by means of H_1_R. For a detailed analysis of histamine receptor function, esp. that of H_1_R and H_4_R, in human colon-derived cell lines, the cell lines tested here are not fully convenient unless genetically modified.

**Supplementary information:**

The online version contains supplementary material available at 10.1007/s00210-023-02565-8.

## Introduction

Histamine (2-(4-imidazolyl)-ethylamine) is a biogenic amine involved in a broad variety of (patho)physiological processes (Seifert et al. 2013; Tiligada and Ennis 2020). Most prominent functions of histamine are the triggering of allergic symptoms, and the regulation of gastric acid production, since so-called ‘antihistamines’ can be used to pharmacologically interfere with allergy and acid-related gastric disorders (Barocelli and Ballabeni 2003; Thangam et al. 2018). Histamine also acts as neurotransmitter, a function that latterly gained attraction since the H_3_R inverse agonist pitolisant targeting the histaminergic system has been approved for the therapy of narcolepsy (Sadek et al. 2016). Finally, histamine is also a mediator of inflammation, involved in acute and chronic inflammatory processes (Neumann et al. 2014).

Functional selectivity within the histamine system is based on the existence of four different histamine receptor subtypes that demonstrate differential cellular expression patterns (Morini et al. 2008; Strasser et al. 2013). Histamine receptors belong to the class A rhodopsin-like G protein-coupled receptors (GPCR) and are referred to as histamine H_1_ receptor (H_1_R), H_2_R, H_3_R, and H_4_R (Neumann and Seifert 2014). Expression of H_3_R is largely restricted to the presynaptic membrane of neuronal cells, where it reduces mediator release upon activation (Nieto-Alamilla et al. 2016a). H_1_R and H_2_R are present quite ubiquitously and regulate allergic / inflammatory reactions and gastric acid, respectively, as mentioned above. H_4_R, lastly, was originally identified on hematopoietic cells, implicating its involvement in inflammation (Schirmer and Neumann 2021). However, several independent studies also demonstrate its expression on non-hematopoietic cells today (Cianchi et al. 2005; Morini et al. 2008; Connelly et al. 2009; Rossbach and Bäumer 2014; Nieto-Alamilla et al. 2016b; Schaper et al. 2016; Schirmer et al. 2020a).

While at least H_1_R, H_2_R, and H_4_R can be identified in human colon tissue (Beermann et al. 2012; Rossbach and Bäumer 2014; Schirmer and Neumann 2021), their specific cellular expression profile within this tissue remains unexploited. It can be assumed that H_1_Rs are present on colon epithelial cells. In the stomach, H_2_Rs are functionally expressed on parietal cells, but regarding colon expression, data are sparse. H_4_Rs are expressed by intraepithelial immune cells, such as mast cells, and most probably by epithelial cells, too. Indeed, in some preliminary experiments using human colon-derived organoids, we detected mRNA encoding H_1_R and H_4_R, but not that encoding H_2_R (unpublished).

Using mouse models for colitis and colitis-associated colon carcinoma in combination with genetic and pharmacological manipulation, we previously demonstrated the involvement of H_4_R in the respective pathologies (Schirmer et al. 2015, 2020b). Moreover, we were able to ascribe H_4_R-mediated histamine function to normal mouse colon epithelial cells (Schirmer et al. 2020a). However, our studies so far lacked mechanistic insight into the function of H_4_R on colon epithelial cells and the transferability to the human system. Nevertheless, available expression data from human biopsies document that H_4_R expression in tumor tissues is reduced as compared to healthy tissue (Boer et al. 2008; Fang et al. 2011). In synopsis, these data hint towards a function of H_4_R in epithelial tumorigenesis. Besides animals, cell lines are versatile models to investigate cellular and molecular mechanisms of a given system. In contrast to studies involving animals, cell-based models are ethically favorable, easier and cheaper to handle, and, most importantly, able to produce a large amount of samples, frequently necessary for biochemical analyses. Evidence for an expression of H_4_R on colon epithelial cell lines is of high interest to further pursuing the hypothesis of a direct involvement of the H_4_R in epithelial tumorigenesis.

Thus, in the present study, we comprehensively screened cell lines for their H_1_R and H_4_R mRNA expression profile and their reactivity to histamine stimulation, three originating from hematopoietic cells and 7 epithelial cell lines, with an emphasis on those derived from human colon tissue. We provide data indicating that human colon-derived cell lines mostly express H_1_R, while expression of H_4_R is detected only occasionally. Combining these expression data with functional analyses, we learned that they do not necessarily correspond with each other.

## Materials and methods

### Materials

If not stated otherwise, all chemicals were obtained from Sigma-Aldrich (Taufenkirchen, Germany). Primers and probes used for the quantitative PCR were purchased from Applied Biosystems (Darmstadt, Germany). The H_4_R-selective antagonist JNJ7777120 (1-[(5-chloro-1H-indol-2-yl) carbonyl]-4-methyl¬perazine) was kindly provided by Dr. Armin Buschauer (University of Regensburg, Germany).

### Cell culture

The cell lines were obtained from LGC Standards (ATCC) (Wesel, Germany) and were maintained strictly as indicated by the supplier (www.lgcstandards.com). Cell culture media and fetal calf serum (FCS) were from Sigma-Aldrich. Main characteristics (Ahmed et al. 2013) and culture media of the individual cell lines used are summarized in Table 1 (Table 1) and supplementary table 1 (Table S1), respectively.

**Table 1:** Selected characteristics^1^ of CRC cell lines used in this study

Cell line	MSI Status	CIMP	CIN	KRAS	BRAF	PIK3CA	PTEN	TP53
Lovo	MSI	-	-	G13D; A14V	wt	wt	wt	wt
Caco-2	MSS	+	+	wt	wt	wt	wt	E204X
HCT-116	MSI	+	-	G13D	wt	H1047R	wt	wt
HT-29	MSS	+	+	wt	V600E	P449T	wt	R273H
SW480	MSS	-	+	G12V	wt	wt	wt	R273H;P309S

^1^*CIN* chromosomal instability; *MSI* microsatellite instable; *MSS* microsatellite stable; *CIMP* CpG island methylator phenotype; *X* stop codon; *wt* wild type

### RNA extraction and real-time PCR

Cells were washed twice with phosphate-buffered saline (PBS) and total RNA was extracted using the NucleoSpin RNA kit (Macherey–Nagel, Düren, Germany) that includes degradation of contaminating DNA by DNase, according to the manufacturer’s protocol. Two μg RNA were reverse-transcribed into cDNA using oligo dT primers (Fermentas, Rockford, IL, USA) and RevertAid Reverse Transcriptase (Fermentas). Real-time PCR was performed using the TaqMan probe-based detection method. Buffers and TaqMan probes were purchased from Applied Biosystems (Darmstadt, Germany; Table 2) and the assay was performed according to the manufacture’s protocol. For standardization, the reference gene β-actin was employed. In order to control specific amplification of mRNA, for reverse transcription (RT) every RNA sample was processed in parallel both in the presence (+ RT) and in the absence (-RT) of the enzyme reverse transcriptase. Thus, qPCR was run with two templates for each sample, of which the -RT template served as negative control that, in case of occurrence of an amplification product, indicates DNA contamination in the corresponding RNA sample or unspecific amplification products. In addition, no-template controls were performed and only if these resulted negative, the corresponding analytic qPCR data from + RT and –RT templates were included in the evaluation. Resulting data are not reported as C_T_ or ΔC_T_ values, but the normalized reporter (ΔRn) values are plotted for each amplification cycle, corresponding to the fluorescence signal from the reporter dye normalized to the fluorescence signal of the passive reporter subtracted by the baseline value. Since, in addition, we showed both the samples values (+ RT) and the – to our opinion – most specific negative controls (-RT), the data are provided in a most transparent manner, enabling the reader to judge their validity.

**Table 2:** TaqMan assays used in this study

Target	Order number	Assay Design	Supplier
β-actin	Hs99999903_m1	Amplicon spans exons	Applied Biosystems, Waltham, MA, USA
β-actin	Hs.PT.39a.22214847	Amplicon spans exons	Integrated DNA Technologies, Coralville, IA, USA
H_1_R	Hs00911670_s1	Primers/probes within protein-coding exon; detects gDNA	Applied Biosystems, Waltham, MA, USA
H_2_R	Hs00254569_s1	Primers/probes within protein-coding exon; detects gDNA	Applied Biosystems, Waltham, MA, USA
H_3_R	Hs00200610_m1	Probe spans exons	Applied Biosystems, Waltham, MA, USA
H_4_R	Hs00222094_m1 (used for carcinoma cell lines)	Probe spans exons detects isoform 1	Applied Biosystems, Waltham, MA, USA
H_4_R	Hs01010880_m1 (used for leukemic cell lines)	Probe spans exons detects isoform 1	Applied Biosystems, Waltham, MA, USA

### Calcium mobilization assay (adapted from (Kao et al. 2010))

Cells were cultured under standard conditions in cell culture flasks until they reached ~ 75% confluence, harvested and seeded in black 96-well plates at a density of 2.5 × 10^4^ cells/well. After 24 h, cells were incubated for 1 h at 37 °C with 5 µM of the Ca^2+^-sensitive fluorescent dye Calbryte 520-AM (K_D_ = 1200 nM;  λ_ex,max_ = 492 nm, λ_em,max_ = 514 nm) in Krebs-HEPES buffer (120 mM NaCl, 20 mM HEPES, 4.7 mM KCl, 1.2 mM KH_2_PO_4_, 1.2 mM MgSO_4_, 1.25 mM CaCl_2_, 10 mM glucose, pH 7.4), containing 0.04% Pluronic F-127 and 1 mM probenecid (Liao et al. 2021). To remove any excess dye, labelling solution was replaced with Krebs-HEPES buffer containing 1 mM probenecid. In antagonist studies, the buffer was supplemented with 10 µM of the respective antagonist (JNJ7777120/cetirizine/famotidine). Fluorescence was detected using a BioTek™ Synergy™ 4 microplate reader using optical filters (excitation filter: 485/20 nm, emission filter: 528/20 nm). After detection of baseline signal for 3 min, histamine was added to yield the final concentrations indicated and the signal was detected for 2 min. Then, Triton X-100 at a final concentration of 0.5% (w/v) was added and the maximum signal (F_max_) was detected over a period of 3 min. Finally, EGTA was added at a final concentration of 15 mM and the minimum signal (F_min_) was detected for additional 3 min. The increase in rel. [Ca^2+^]_i_ was calculated as the difference between rel. [Ca^2+^]_i_ at baseline and rel. [Ca^2+^]_i_ after stimulation, which were calculated from the fluorescence data using the following equation:

$${rel.\;\left[{Ca}^{2+}\right]}_i=K_D\frac{F-F_{min}}{F_{max}-F}$$

### cAMP accumulation assay

Cells were seeded in 6-well plates at 1 × 10^6^ cells/well and cultured for 24 h. Cells were stimulated for 10 min at 37 °C with 10 µM forskolin in the presence or absence of histamine at the concentrations indicated. After removal of medium the cells were washed once with PBS and 300 µl of extraction solvent (AcN/MeOH/H_2_O (2:2:1)) containing 25 ng/ml tenofovir (internal standard for HPLC–MS/MS method (Beste et al. 2012)) were added to the wells. The cells were harvested by scraping into ice-cold extraction solvent. Scraper and wells were washed twice with 400 µl extraction solvent without internal standard and the respective extracts (1100 µl final volume) were combined. To optimize protein precipitation, extracts were incubated at 95 °C for 10 min and frozen for at least 2 h at -80 °C. Precipitated protein was spun down by centrifugation for 10 min at 20,800 × g. Supernatant fluids were transferred into a new tube and evaporated at 40 °C under nitrogen flow until complete dryness. Residual material was dissolved in 150 µl H_2_O. Samples were analyzed on an API 5500 mass spectrometer (AB SCIEX, Framingham, MA, USA) after HPLC-separation using a Zorbax Eclipse column XDB-C18 1.8 µm 50 × 4.6 (Agilent Technologies, Santa Clara, CA). cAMP concentrations in samples were calculated according to standards containing defined cAMP concentrations. The protein pellets were dried at RT and dissolved in 800 µl 0.1 M NaOH at 95 °C for 15 min. Protein concentrations were quantified using BCA-assay (Thermo Fisher Scientific, Waltham, MA, USA). cAMP concentrations were calculated in relation to the total protein concentration (pmol cAMP/mg protein).

### Metabolic activity (alamarBlue) assay

Cells were seeded in 96-well plates at a density of 5 × 10^3^ cells/well in technical quadruplicates, cultured under standard conditions for 24 h, and then stimulated by adding 10 µM (final) histamine and further incubation for 24 h. Subsequently, alamarBlue® reagent (Bio-Rad Laboratories GmbH, Feldkirchen, Germany) was added to the wells to a final concentration of 10% and cultured for an additional 4 h under standard conditions. Absorption at 570 and 600 nm was measured using the BioTek™ Synergy™ 4 microplate reader and metabolic activity was calculated in relation to an unstimulated control using the following equation according to the manufacturer’s protocol:

$$metabolic\;activity\;(\%)=\frac{\left(O2\times A2\right)-(O1\times A1)}{\left(R1\times N2\right)-\left(R2\times N1\right)}\times100$$

O1 = molar extinction coefficient (E) of oxidized alamarBlue at 570 nm, O2 = E of oxidized alamarBlue at 600 nm, R1 = E of reduced alamarBlue at 570 nm, R2 = E of reduced alamarBlue at 600 nm, A1 = absorbance of test wells at 570 nm, A2 = absorbance of test wells at 600 nm, N1 = absorbance of negative control well at 570 nm, N2 = absorbance of negative control well at 600 nm.

### Proliferation (xCelligence) assay

Cellular proliferation was measured using the xCELLigence™ 16S real-time cell analyzer (RTCA) (ACEA Biosciences, San Diego, USA). By continuous measurement of changes in impedance between the microelectrodes on the bottom of the wells of the E-Plates®, the xCELLigence™ system allows for real-time analysis of cellular proliferation. A reference value was generated by adding 50 µl medium to the wells and measuring the impedance without cells. Subsequently, 10^4^ cells were seeded into each well. To allow attachment of the cells, the E-Plate® was incubated for 30 min at room temperature, before histamine and cytochalasin B, which served as a positive control, were added to a final concentration of 10 µM and 1 µg/ml, respectively. Thereafter, the E-Plates® were incubated at 37 °C under standard cell culture conditions. Impedance was measured every 15 min for at least 72 h. Ranges of exponential growth were defined for every cell line and doubling time was calculated using the xCELLigence™ RTCA software.

### Cell membrane protein identification by HPLC–MS

HCT116 cells were seeded with 2 × 10^6^ cells in each of two 100 mm-petri dishes and cultured under standard conditions to reach 80% confluency. Cells were washed with PBS and lysed in ice-cold urea buffer (8 M urea, 50 mM Tris/HCl, pH 7.5). The lysates were pooled, sonicated, and cellular debris was removed by centrifugation (18,000 × g, 10 min, 4 °C). Half of the resulting supernatant (total lysate) was submitted to membrane preparation by ultracentrifugation (60,000 g, 1 h, 4 °C). The resulting pellet was resuspended in 50 µl urea buffer and the protein concentration determined by BCA assay (Thermo Fisher Scientific).

An aliquot of the suspension was mixed with Laemmli buffer and incubated for 5 min at 95 °C, and then the proteins were alkylated by incubation with acrylamide at a final concentration of 2% (w/v) at room temperature for 30 min. The proteins, separated by SDS PAGE, were visualized by Coomassie Brilliant Blue staining and the proteins-containing lane of the gel was cut out and minced into 1 mm^3^ pieces. The resulting pieces were destained with 50% acetonitril (ACN), 50 mM ammonium bicarbonate (ABC), dehydrated with 100% ACN, dryed in a vacuum centrifuge, and rehydrated in 10 ng/µl sequencing grade trypsin (Promega) in 10% ACN, 40 mM ABC for 1 h on ice. Protein digestion, which was performed over-night at 37 °C, was stopped by adding 50% ACN, 0,1% trifluor acetic acid (TFA) and incubation at 37 °C for 1 h. After drying the samples in a vacuum centrifuge, they were redissolved in 2% ACN, 0.1% TFA and stored at -20 °C until analysis.

Peptide samples were separated with a nano-flow ultra-high pressure liquid chromatography system (RSLC, Thermo Scientific) equipped with a trapping column (3 µm C18 particle, 2 cm length, 75 µm ID, Acclaim PepMap, Thermo Scientific) and a 50 cm long separation column (2 µm C18 particle, 75 µm ID, Acclaim PepMap, Thermo Scientific). Peptide mixtures were injected, enriched and desalted on the trapping column at a flow rate of 6 µl/min with 0.1% TFA for 5 min. The trapping column was switched online with the separating column and peptides were eluted with a multi-step binary gradient: linear gradient of buffer B (80% ACN, 0.1% formic acid) in buffer A (0.1% formic acid) from 4 to 25% in 30 min, 25% to 50% in 10 min, 50% to 90% in 5 min and 10 min at 90% B. The column was reconditioned to 4% B in 15 min. The flow rate was 250 nl/min and the column temperature was set to 45 °C. The RSLC system was coupled online via a Nano Spray Soure II (Thermo Scientific) to Orbitrap Exploris 240 mass spectrometer. Metal-coated fused-silica emitters (SilicaTip, 10 µm i.d., New Objectives) and a voltage of 2.1 kV were used for the electrospray. Overview scans were acquired at a resolution of 120 k in a mass range of m/z 300–1500. Precursor ions of charges two or higher and a minimum intensity of 4000 counts were selected for HCD fragmentation with a normalized collision energy of 38.0, an activation time of 10 ms and an activation Q of 0.250. Active exclusion was set to 70 s within a mass window of 10 ppm of the specific m/z value.

Raw data were processed using Max Quant software (version 1.5), and Perseus software (version 1.6.2.3) and human entries of uniprot DB. Proteins were stated identified by a false discovery rate of 0.01 on protein and peptide level.

### Statistical analysis

If not stated otherwise, statistical analyses were performed by calculating means ± SD of at least three independent determinations. Analysis of significance was performed using Student’s t-test or one-way ANOVA with Holm-Sidaks post-test for linear parameters (GraphPad Prism 5). p-values of ≤ 0.05 (*) were considered significant.

## Results

### Histamine receptor subtype mRNA expression

The detection of histamine receptor subtype expression is burdened with some difficulties, similar to that of several other GPCRs as well (Lu and Bartfai 2009; Michel et al. 2009; Beermann et al. 2012; Marchalant et al. 2014). Selective antibodies recognizing H_4_R, validated by rigorous analyses, are still missing. Thus, identification and quantification of H_4_R expression is restricted to mRNA analyses and to functional studies involving selective inhibitors. Here, we started with RT-qPCR analyses, measuring the mRNA abundance of H_1_R, H_2_R, H_3_R, H_4_R and *β*-actin in several cell lines, grown under normal cell culture conditions without any additional treatment. Resulting qPCR cycle number-dependent ΔRn values are reported in supplementary Figs. 1 and 2 (Figures S1 and S2) and are summarized in Table 3 (Table 3). *β*-actin mRNA, used as reference gene control, was readily detected in all + RT templates of every cell line tested, and, albeit at very high cycle numbers, in all –RT templates, too. Thus, minor DNA contaminations or unspecific amplifications seem to have been present, rendering it necessary to implement this control in all qPCR analyses.

**Table 3:** Summary of histamine receptor mRNA expression quantification^1^ in cell lines

Cell line	H_1_R	H_2_R	H_3_R	H_4_R
HMC1	+	+	-	+
HL-60	+	( +)	-	( +)
U937	+	+	-	-
A549	( +)	( +)	( +)	-
Calu-3	+	-	( +)	-
LoVo	+	-	( +)	-
SW 480	+	-	( +)	-
Caco-2	-	( +)	+	-
HT-29	+	-	-	-
HCT116	+	-	-	+

^1^(+ : significant amounts of mRNA detectable; ( +): negligible amounts of mRNA detectable; -: mRNA not detectable)

Our major interest focuses on H_1_R and H_4_R on colon epithelial cells of human origin. Thus, we chose five human epithelial cell lines of colorectal origin (LoVo, SW480, Caco-2, HT-29, and HCT116) and, as controls, two human lung epithelial cell lines (A549, Calu-3), and three cell lines of human hematopoietic origin (HMC1, HL-60, and U937). In all cell lines analyzed, H_1_R mRNA was certainly present, with the exception of A549 cells, in which H_1_R mRNA was, if at all, very sparse, and Caco-2 cells, in which H_1_R mRNA was not detectable. H_4_R mRNA was present in two out of the three hematopoietic cell lines analyzed, HMC1 and HL-60, but not in U937 cells (Werner et al. 2014). In cell lines of epithelial origin, only HCT116 cells readily demonstrated the presence of H_4_R mRNA. In synopsis, it appeared that the herein analyzed cell lines of epithelial origin with some exceptions express H_1_R mRNA and only occasionally H_4_R mRNA.

mRNA encoding H_2_R was readily detected in HMC-1 and U937 cells and, in negligible amounts only, in HL-60, A549 and Caco-2 cells. In all remaining cell lines, there was no evidence of H_2_R expression detectable. H_3_R expression was not detectable in cell lines of hematopoietic origin, but present in Caco-2 cells and, albeit at negligible amounts, in the lung epithelial cell lines and in LoVo, SW480.

### Histamine-induced calcium mobilization

**Fig. 1:**
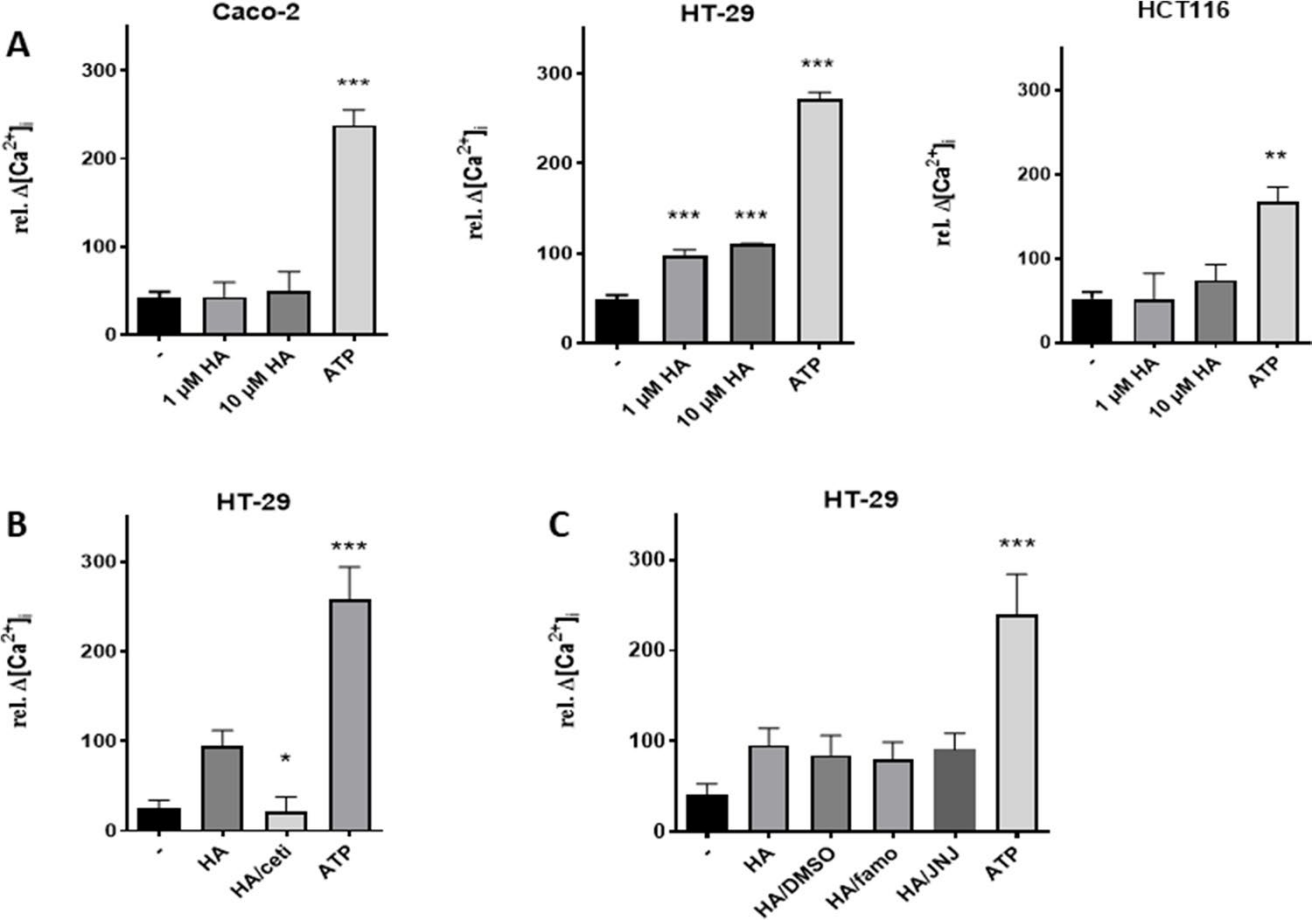
Intracellular calcium ion mobilization. Cells of the indicated cell lines were stimulated with histamine (HA; if not stated otherwise 10 µM) in the absence (**A**) or presence of 10 µM cetirizine (ceti) (**B**), or 10 µM famotidine (famo) or 10 µM JNJ7777120 (JNJ) (**C**) and intracellular calcium ion mobilization was monitored by Calbryte 520 fluorescence measurement. Addition of ATP (100 µM) or DMSO (0.1%; solvent of JNJ7777120) served as controls. Reported are the means ± SD of 2–3 independent experiments, each run in duplicates. (**: p < 0.01; ***: p < 0.001 *vs.* untreated (**A**) or HA-treated (**B**); one-way ANOVA with Holm-Sidak’s post test)

For the following functional analyses, we selected, corresponding to our major interest, human colon-derived epithelial cell lines that differ in the presence of H_1_R and H_4_R mRNA and focused the analyses on these receptor subtypes. Caco-2 cell presented neither H_1_R nor H_4_R mRNA, HT-29 expressed only H_1_R mRNA, and HCT116 cells demonstrated the presence of H_1_R and H_4_R mRNA. A cell line which exclusively expressed endogenous H_4_R mRNA was not identified in this study. Mobilization of cytosolic calcium ions is linked to H_1_R and H_4_R activation, which mainly couple to G_αq_ and G_αi/o_ proteins, respectively (Beermann et al. 2014). Thus, the cellular response of the three selected cell lines to histamine stimulation was analyzed by calcium ion mobilization assay, i.e. by quantification of stimulation-induced alteration of rel. [Ca^2+^]_i_ (Fig. 1). The addition of 10 µM ATP to the cells served as positive control. Only if ATP led to enhancement of rel. [Ca^2+^]_i,_, the corresponding analytical data gained by stimulation using histamine receptor ligands were evaluated. Neither in Caco-2 cells nor in HCT116 cells histamine efficiently induced calcium mobilization (Fig. 1A). In HT-29 cells, in contrast, a small but robust histamine-induced mobilization of calcium ions appeared. This effect was inhibited by the H_1_R-selective antagonist cetirizine, but not by the H_2_R-selective antagonist famotidine nor by the H_4_R-selective antagonist JNJ7777120 (Fig. 1B).

### Histamine-induced cAMP accumulation and proliferation

**Fig. 2:**
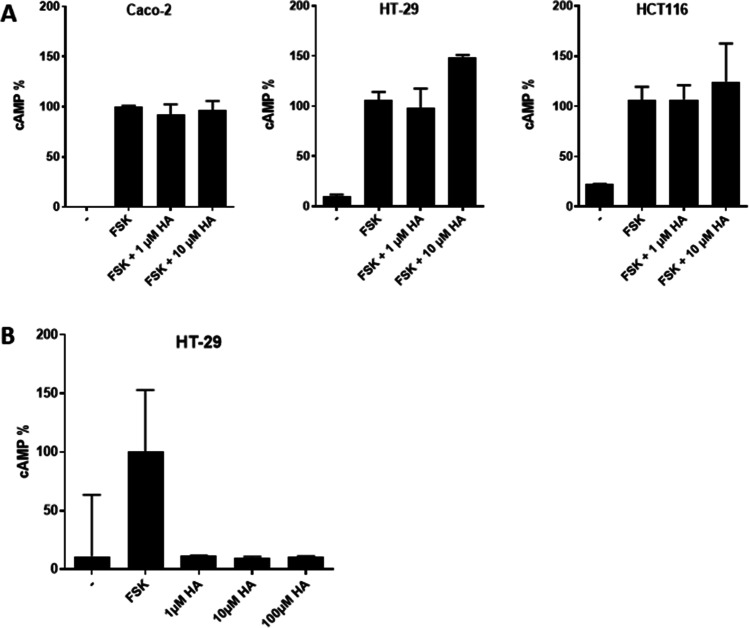
cAMP accumulation. Cells of the indicated cell lines were cultured for 10 min in normal cell culture medium (-), in medium supplemented with forskolin (FSK), or in medium supplemented with increasing concentrations of histamine (HA) either (**A**) or not (**B**) in combination with FSK. Cells were harvested and cAMP concentrations were quantified by LC–MS/MS. For each cell line, one randomly chosen concentration (pmol cAMP/mg total cellular protein) of FSK-treated cells (A: Caco-2: 1,972; HT-29: 0,224; HCT116: 0,187; B: HT-29: 0,337) was set at 100% and the other values were calculated correspondingly. Reported are means ± SD of 1–3 independent analyses, each run in technical du- tri-, or quadruplicates

Furthermore, cytosolic cAMP, associated with G_αs_ – and G_αi/o_ -induced signaling, was quantified in the selected cell lines without stimulation and after incubation with histamine in the presence (Fig. 2A) and absence (Fig. 2B) of forskolin, an activator of adenylyl cyclases, and histamine. Forskolin stimulation resulted in a robust generation of cAMP in the three cell lines analyzed. The addition of histamine, however, did not induce cAMP accumulation nor did it modulate the forskolin-induced response.

**Fig. 3:**
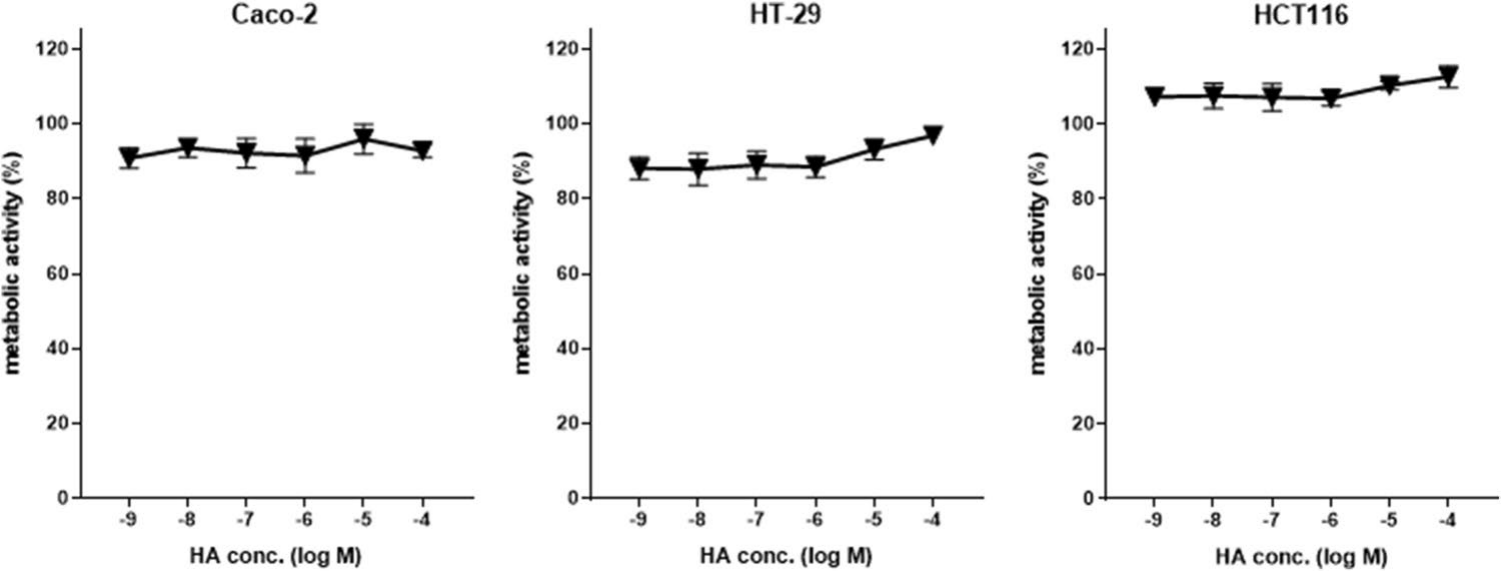
Metabolic activity of cells. Cells of the indicated cell lines were cultured for 24 h either in normal cell culture medium or in medium supplemented with increasing concentrations of histamine (HA). During the last 4 h, the dye resazurin (alamarBlue) was added and conversion of the dye was photometrically quantified. The color change, indicative for mitochondrial/metabolic activity, was calculated in relation to that of untreated cells (= 100%). Reported are means ± SD of 3–4 independent analyses, each run in technical quadruplicates

**Fig. 4:**
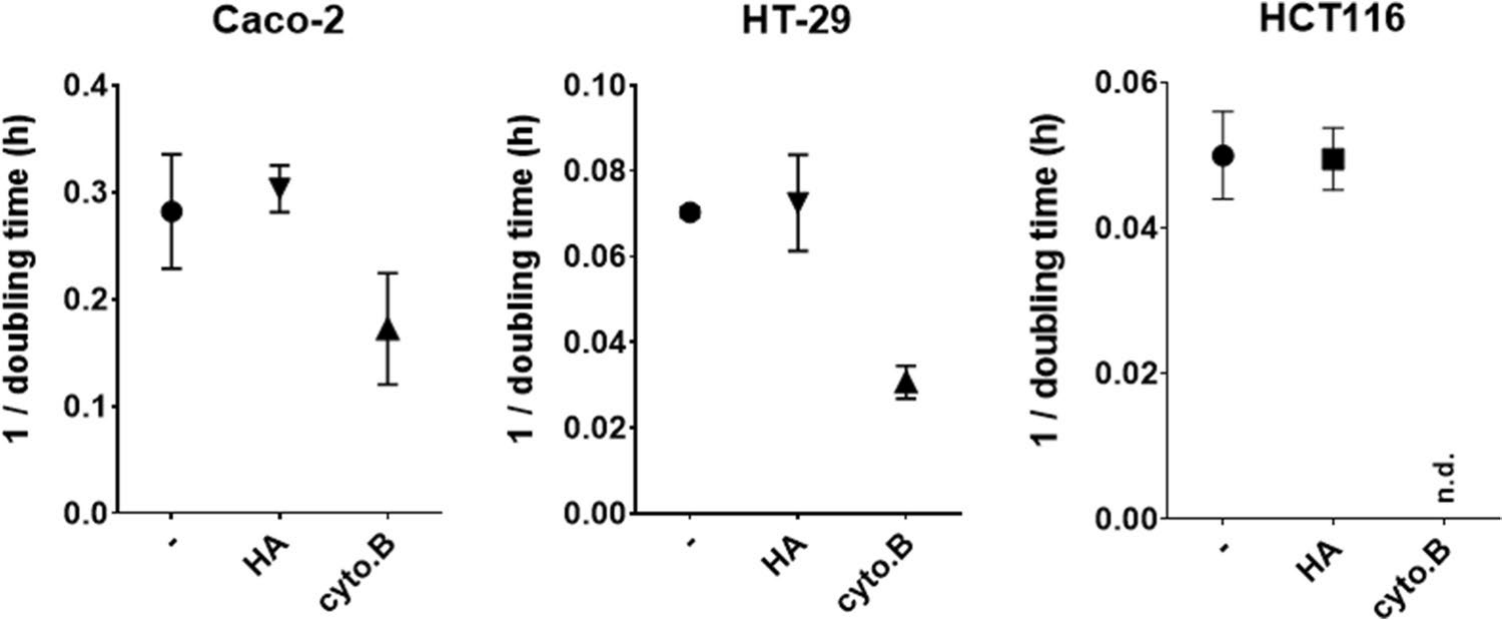
Proliferation of cells. Indicated cells were seeded in normal cell culture medium and cultured either in the absence (-) or in the presence of 10 µM histamine (HA) or 1 µg/ml cytochalasin B (cyto B). Cell behavior was continuously analyzed label-free by biosensors (RTCA; real-time cell analysis) for 96 h. From the slopes of the resulting curves, doubling times were calculated specifically for each cell line and treatment

Proliferation is a key feature discriminating cancer from normal cells. Whether or not histamine interferes in the cancer cell phenotype was thus analyzed in Caco-2, HCT116, and HT-29 cells applying two different methods (Figs. 3 and 4) in the presence or absence of histamine. Histamine stimulation did not demonstrate any impact on metabolic activity (Fig. 3) or proliferation (Fig. 4), while cytochalasin B, a known inhibitor of actin polymerization, significantly enhanced doubling time, thus reduced proliferation (Fig. 4).

### Analysis of H_1_R and H_4_R protein expression in HCT116

Due to the lack of tools for the reliable identification of histamine receptor proteins, a possible presence of H_1_R and H_4_R in HCT116 cell membranes was analyzed by HPLC–MS. A total of 4867 proteins were identified (Table S2), including several GPCR. Surprisingly, ions indicative for H_1_R and H_4_R proteins were not detected.

## Discussion

### Histamine receptor expression analyses

The cell/tissue type-specific expression of histamine receptor subtypes, especially that of pro-inflammatory H_4_R, is still a matter of debate. While H_4_R originally has been identified in cells of hematopoietic origin, today several studies report its expression in other tissues, too. Earlier, we provided evidence for H_4_R expression in healthy colon epithelial cells of mice and man. These data were based on mRNA quantification only, since tools to reliably detect H_4_R protein were not available, but they were supported by specific functional analyses (Schirmer et al. 2020a). Data provided by others indicate that the expression of H_4_R in human colon adenoma/carcinoma is decreased (Boer et al. 2008; Fang et al. 2011), questioning whether cell lines of human colon epithelial origin, which are mostly derived from adenomas/carcinomas, provide useful models to investigate H_4_R functions. These reasons together with the fact that H_1_R, that provide pro-inflammatory functions as well as H_4_R, is ubiquitously expressed (Jutel et al. 2009) led us to focus this study on the histamine receptor subtypes H_1_R and H_4_R.

### H_1_R and H_4_R mRNA in colon cell lines

The qPCR analyses of H_1_R mRNA affirmed its ubiquitous expression patterns (Jutel et al. 2009). Most importantly for this study, H_1_R was detected in all human colon-derived cell lines with the exception of Caco-2, in which its presence was at best uncertain. The presence of H_4_R mRNA was reliably verified in HMC1 and less pronounced in HL-60 cells, while it was undetectable in any of the tested human colon epithelial cell lines, except HCT116 cells. Other authors have presented histamine receptor expression, including H_4_R, in colonic cell lines, also in those, we used in our study (Cianchi et al. 2005; Boer et al. 2008). This difference may be based on technical issues. We used cells directly obtained from a commercial repository, who permanently keeps them under genetic control, and handled the cells strictly according to the repository’s recommendations, while in the compared studies cell lines were provided by cooperating laboratories. In addition, detection of H_4_R by immunostaining has raised some uncertainties (Boer et al. 2008) and the specificity of the primer sequences provided in one of the studies could not be verified using the NCBI BLAST algorithm and the human RefSeq mRNA database (25.04.2022). Thus, as far as we evaluate mRNA expression only, among the cell lines derived from colorectal carcinoma/adenoma, nearly all express H_1_R but only HCT116 cells express H_4_R.

### Histamine-induced functions in Caco-2, HT-29, and HCT116 cells

The presence of a specific mRNA generally results in its protein expression, however, some exceptions from this rule have been described (Liu et al. 2016). Moreover, the quantity of a specific mRNA does not necessarily correspond to the quantity of its translated protein. Thus, we checked the histamine receptors functions in a representative subset of colon cancer cell lines. As expected, Caco-2 cells were refractory to histamine stimulation in all assays performed, confirming the absence of histamine receptor expression in these cells. HT-29 cells in contrast, not only express H_1_R mRNA, but also mobilize cytosolic calcium ions in response to histamine stimulation in a H_1_R-selective manner. cAMP accumulation, basal or induced by non-selective adenylyl cyclase (AC) activation, was not modified by histamine, as observed in HEK293 cells exogenously expressing the mouse H_1_R (Beermann et al. 2014). The regulation of AC activation by H_1_R is driven by the G protein subunit G_βγ_ (Maruko et al. 2005) and, subsequently, by cytosolic calcium ions either or not in combination with calmodulin (MacNeil et al. 1985). HT-29 cells express calmodulin (Chattopadhyay et al. 2013) and demonstrate a robust calcium response to histamine stimulation, excluding cell type-specific disruption of this signaling pathway. Thus, the AC isoforms expressed and/or functional in HT-29 probably do not belong to those regulated by the calcium/calmodulin module, i.e. AC1 and AC8 (Freeman and MacNaughton 2004). Moreover, a significant histamine-induced activation of p42/44 and p38 MAPK in HT-29 cells could not be detected in this study (Fig. S3), as already observed by Uwada et al. (Uwada et al. 2017). Thus, the response of HT-29 cells to histamine stimulation seems to be very selective, which will be analyzed in a subsequent study.

HCT116 cells, although H_1_R and H_4_R mRNA species have been detected by RT-PCR, did respond to histamine stimulation neither by calcium mobilization nor by cAMP accumulation. This may be due to the disruption of necessary signaling modules or, most proximal, by alterations of the receptors. While sequence analyses of the H_1_R and H_4_R DNAs obtained from HCT116 cells demonstrated no mutations, HPLC–MS analyses were unable to detect any signs for H_1_R and H_4_R proteins in HCT116 cells. Of course, this may be based on the rather low numbers of histamine receptor protein molecules expressed, which remain, although this method provide a high sensitivity, below detection. Thus, receptor protein expression is still possible, but, if at all, on a very low level, questioning its functional relevance. The molecular bases of the discrepancy between mRNA and protein expression as discussed above (Liu et al. 2016) is still enigmatic. Thus, while unmodified Caco-2 cells and HCT116 cells are useless as models to investigate H_1_R and H_4_R functions, HT-29 cells serve as reliably model to analyze H_1_R function in a human colon-derived cell line. A reliable model for the analysis of endogenously expressed H_4_R or H_1_R and H_4_R in combination was not detected among the cell lines tested. The analysis of H_1_R and H_4_R in combination is of utmost interest, since in a previous study we could demonstrate a functional synergism of H_1_R and H_4_R activating the MAPK pathway (Beermann et al. 2015). This finding was reproduced and further substantiated by Verweij et al. (Verweij et al. 2020), who provided evidence that at least the H_4_R relays the primary receptor signal to MAPKs via β-arrestin signaling. The H_4_R may, of course, be involved in more than one kinase pathway. Thus, it will be crucial to further characterizing the functional impact of H_1_R and H_4_R in colon cancer cell lines using *e.g.* phosphoproteomics.

### Histamine and proliferation in Caco-2, HT-29, and HCT116 cells

Enhanced cellular metabolism and proliferation are hallmarks of cancer cells. Several authors have proposed an effect of histamine on these functions (Cianchi et al. 2005; Massari et al. 2020). In the three cell lines analyzed in detail in this study, histamine did not demonstrate any such effect, not even in HT-29 cells, that express a functional H_1_R. Thus, we conclude that the possible impact of histamine on colon tumor cell proliferation is not mediated via H_1_R. Indeed, regulation of cell proliferation by histamine may be mediated exclusively by H_4_R and, thus, could not be detected in the cell lines analyzed. The lack of functional H_4_R expression—regulated either on the level of transcription (Caco-2, HT-29) or of translation (HCT116)—may be explained by the colorectal carcinoma origin of the cell lines, in which a reduced H_4_R expression has already been demonstrated (Boer et al. 2008; Fang et al. 2011). By such mechanism, carcinoma cells would evade the inhibition of proliferation mediated by histamine via H_4_R.

In conclusion, in the present study we provide evidence that human colon-derived cell lines mostly express H_1_R, while functional expression of H_4_R is not detected only occasionally. Histamine-induced cellular functions do not necessarily reflect the H_X_R expression profile. For a comprehensive and detailed analysis of H_X_R function in human colon epithelial cells, the cell lines tested in this study are not fully convenient unless genetically modified.

### Supplementary Information

Below is the link to the electronic supplementary material.

Supplementary file1 (PPTX 2750 kb)

Supplementary file2 (PPTX 2729 kb)

Supplementary file3 (PPTX 1279 kb)

Supplementary file4 (PDF 90 kb)

Supplementary file5 (PDF 534 kb)

## Data Availability

The datasets generated during and/or analyzed during the current study are available from the corresponding author on reasonable request.
