# Supplementary material for: Histamine H1- and H4-receptor expression in human colon-derived cell lines
Source: Naunyn Schmiedebergs Arch Pharmacol. 2023 Jun 10;396(12):3683–93. doi: 10.1007/s00210-023-02565-8 (PMC10643376; doi:10.1007/s00210-023-02565-8)
Supplement: Supplementary file 1 — Supplementary file1 (PPTX 2750 kb) [file 210_2023_2565_MOESM1_ESM.pptx]

## Slide 1
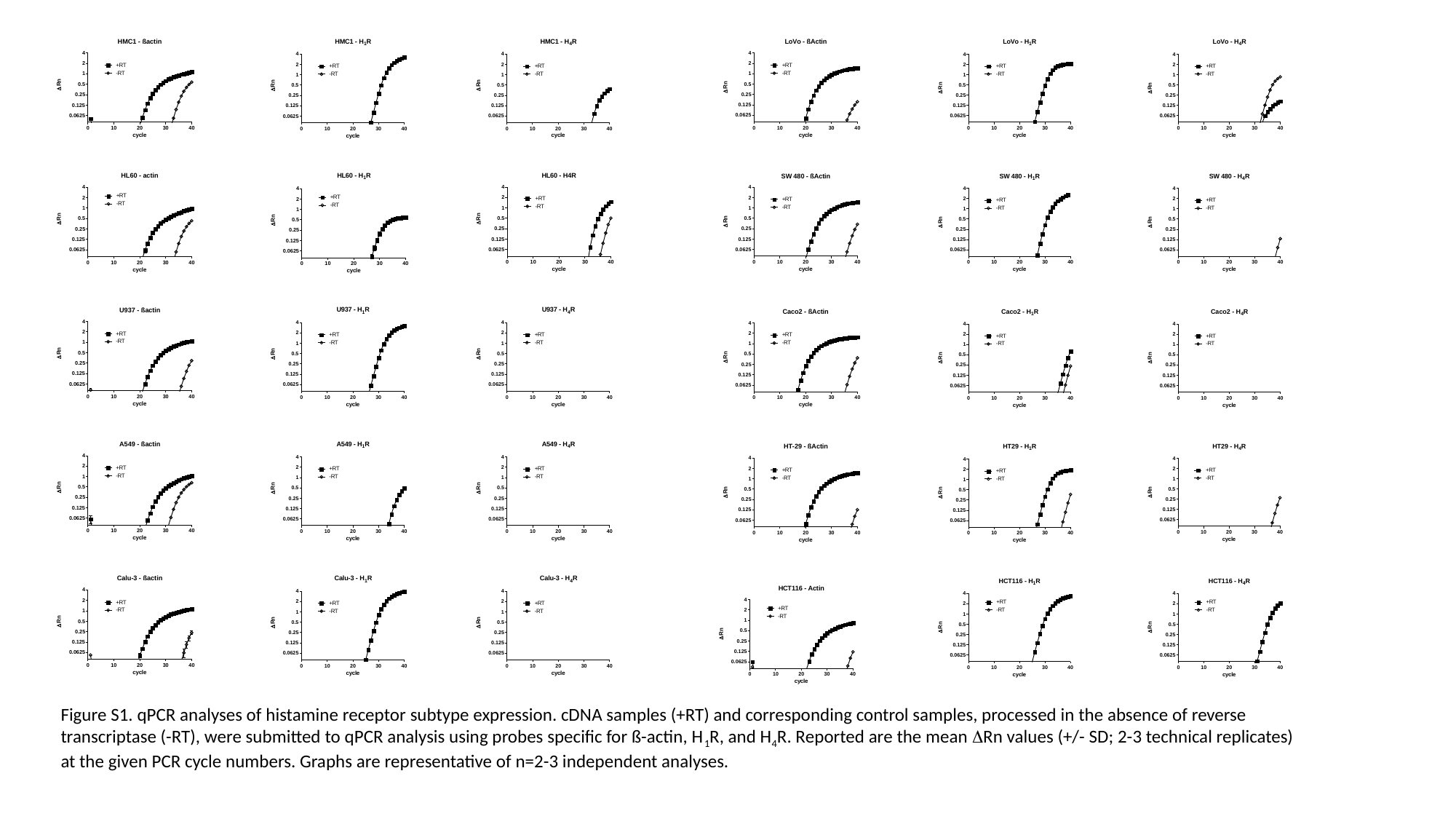

Figure S1. qPCR analyses of histamine receptor subtype expression. cDNA samples (+RT) and corresponding control samples, processed in the absence of reverse transcriptase (-RT), were submitted to qPCR analysis using probes specific for ß-actin, H1R, and H4R. Reported are the mean ΔRn values (+/- SD; 2-3 technical replicates) at the given PCR cycle numbers. Graphs are representative of n=2-3 independent analyses.
