# Supplementary material for: Histamine H1- and H4-receptor expression in human colon-derived cell lines
Source: Naunyn Schmiedebergs Arch Pharmacol. 2023 Jun 10;396(12):3683–93. doi: 10.1007/s00210-023-02565-8 (PMC10643376; doi:10.1007/s00210-023-02565-8)
Supplement: Supplementary file 3 — Supplementary file3 (PPTX 1279 kb) [file 210_2023_2565_MOESM3_ESM.pptx]

## Slide 1
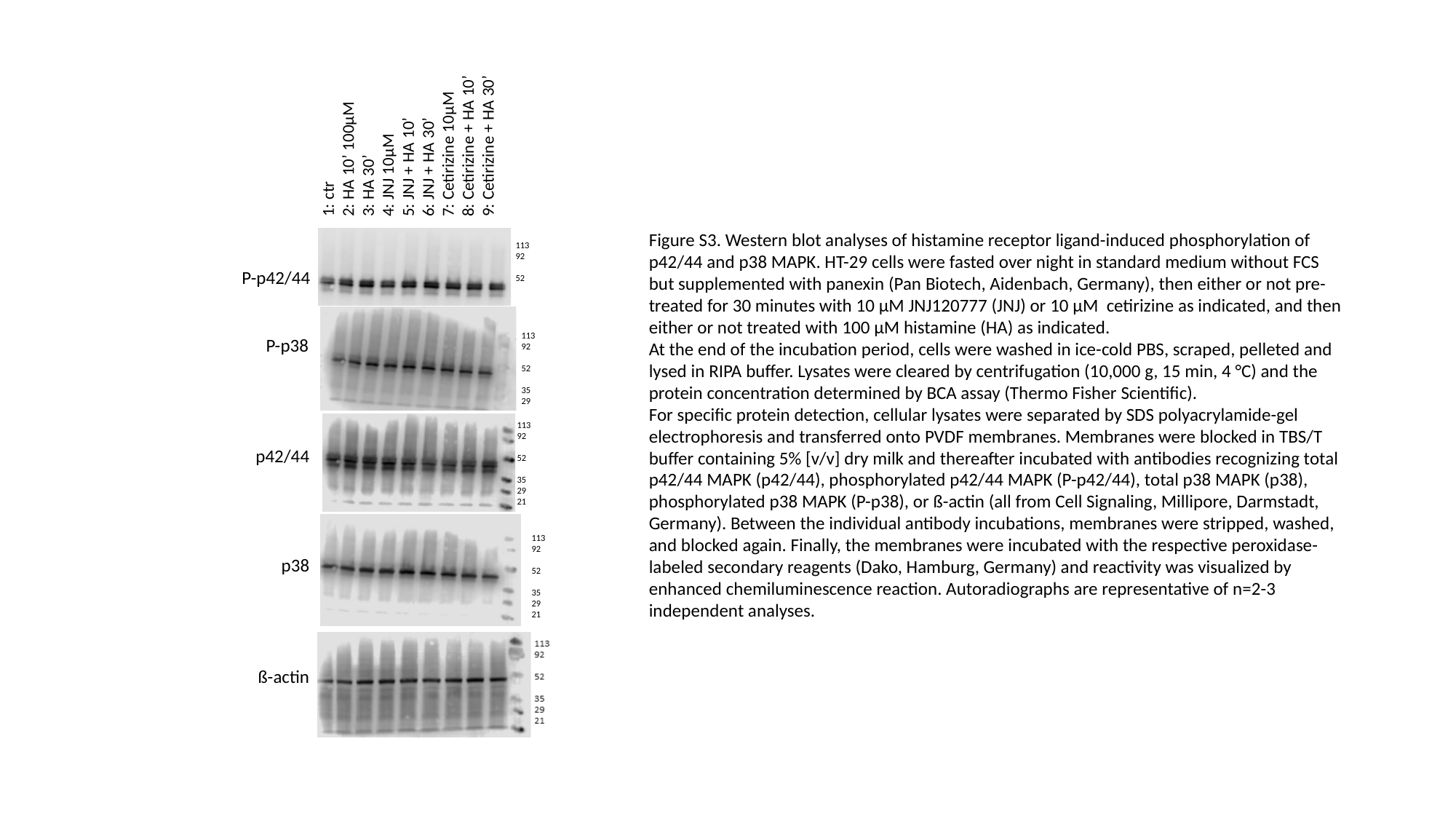

1: ctr
2: HA 10’ 100µM
3: HA 30’
4: JNJ 10µM
5: JNJ + HA 10’
6: JNJ + HA 30’
7: Cetirizine 10µM
8: Cetirizine + HA 10’
9: Cetirizine + HA 30’
Figure S3. Western blot analyses of histamine receptor ligand-induced phosphorylation of p42/44 and p38 MAPK. HT-29 cells were fasted over night in standard medium without FCS but supplemented with panexin (Pan Biotech, Aidenbach, Germany), then either or not pre-treated for 30 minutes with 10 µM JNJ120777 (JNJ) or 10 µM cetirizine as indicated, and then either or not treated with 100 µM histamine (HA) as indicated.
At the end of the incubation period, cells were washed in ice-cold PBS, scraped, pelleted and lysed in RIPA buffer. Lysates were cleared by centrifugation (10,000 g, 15 min, 4 °C) and the protein concentration determined by BCA assay (Thermo Fisher Scientific).
For specific protein detection, cellular lysates were separated by SDS polyacrylamide-gel electrophoresis and transferred onto PVDF membranes. Membranes were blocked in TBS/T buffer containing 5% [v/v] dry milk and thereafter incubated with antibodies recognizing total p42/44 MAPK (p42/44), phosphorylated p42/44 MAPK (P-p42/44), total p38 MAPK (p38), phosphorylated p38 MAPK (P-p38), or ß-actin (all from Cell Signaling, Millipore, Darmstadt, Germany). Between the individual antibody incubations, membranes were stripped, washed, and blocked again. Finally, the membranes were incubated with the respective peroxidase-labeled secondary reagents (Dako, Hamburg, Germany) and reactivity was visualized by enhanced chemiluminescence reaction. Autoradiographs are representative of n=2-3 independent analyses.
113
92
52
P-p42/44
113
92
52
35
29
P-p38
113
92
52
35
29
21
p42/44
113
92
52
35
29
21
p38
ß-actin
