# Supplementary material for: Histamine H1- and H4-receptor expression in human colon-derived cell lines
Source: Naunyn Schmiedebergs Arch Pharmacol. 2023 Jun 10;396(12):3683–93. doi: 10.1007/s00210-023-02565-8 (PMC10643376; doi:10.1007/s00210-023-02565-8)
Supplement: Supplementary file 4 — Supplementary file4 (PDF 90 kb) [file 210_2023_2565_MOESM4_ESM.pdf]

Table S1: Composition of the individual cell culture media.

Cell line	Cell type	Medium	FCS (%)	Supplement *	CO_2 (%)
**HMC1**	Mast cell leukemia	Iscoves MEM	10	0,5 mM DTT	5
**HL-60**	Pro-myeloblast	Iscoves MEM	10		5
**U937**	Histiocytic lymphoma	RPMI 1640	10		5
**A549**	Lung epithelia carcinoma	DMEM F12	10		5
**Calu-3**	Lung adenocarcinoma	MEM	10		5
**LoVo**	Colorectal adenocarcinoma	DMEM F12	10		5
**SW 480**	Colorectal adenocarzinoma	DMEM F12	10		5
**CaCo-2**	Colorectal carcinoma	MEM	20		5
**HT-29**	Colorectal carcinoma	Mc Coys 5a	10		5
**HCT116**	Colorectal carcinoma	Mc Coys 5a	10		5

* All media were additionally supplemented with 100 u/ml penicillin/100 µg/ml streptomycin and 2 mM L-glutamate.
