# Supplementary material for: Histamine H1- and H4-receptor expression in human colon-derived cell lines
Source: Naunyn Schmiedebergs Arch Pharmacol. 2023 Jun 10;396(12):3683–93. doi: 10.1007/s00210-023-02565-8 (PMC10643376; doi:10.1007/s00210-023-02565-8)
Supplement: Supplementary file 5 — Supplementary file5 (PDF 534 kb) [file 210_2023_2565_MOESM5_ESM.pdf]

Peptides	Unique peptides	Sequence coverage [%]	Mol. weight [kDa]	Score	Majority protein IDs	Protein names
21	2	81.6	41.792	323.31	P63261	Actin, cytoplasmic 2;Actin, cytoplasmic 2, N-terminally processed
39	39	76.3	61.054	323.31	P10809	60 kDa heat shock protein, mitochondrial
48	43	72.2	69.293	323.31	sp|P02769|;CON__P02769	
47	30	71	83.263	323.31	P08238	Heat shock protein HSP 90-beta
17	17	68.6	30.772	323.31	P21796	Voltage-dependent anion-selective channel protein 1
41	41	68.2	75.829	323.31	sp|Q29443|;CON__Q29443;sp|Q0IIK2|;CON__Q0IIK2	
24	22	68.2	47.168	323.31	P06733;P06733-2	Alpha-enolase
17	14	67.5	29.174	323.31	P62258;P62258-2	14-3-3 protein epsilon
36	5	67.2	57.936	323.31	sp|P14618|;P14618;P14618-3;sp|FA40-17|PKM2	Pyruvate kinase PKM
14	11	65.4	35.924	323.31	P67809	Nuclease-sensitive element-binding protein 1
50	33	64.9	104.85	323.31	O43707;O43707-2;O43707-3	Alpha-actinin-4
15	15	60.5	31.566	323.31	P45880;P45880-2;P45880-1	Voltage-dependent anion-selective channel protein 2
42	26	58.7	103.06	323.31	P12814;P12814-3;P12814-2;P12814-4	Alpha-actinin-1
42	16	58.1	56.608	323.31	P05787-2; P05787; sp|P05787|;CON__P05787	Keratin, type II cytoskeletal 8
38	34	57.5	96.864	323.31	Q92598;Q92598-2;Q92598-3;Q92598-4	Heat shock protein 105 kDa
39	36	56.2	94.33	323.31	P34932	Heat shock 70 kDa protein 4
14	10	55.5	28.302	323.31	P61981	14-3-3 protein gamma;14-3-3 protein gamma, N-terminally processed
12	2	55.5	24.488	323.31	Q15907;Q15907-2	Ras-related protein Rab-11B
27	27	55.2	88.549	323.31	Q13263;Q13263-2	Transcription intermediary factor 1-beta
63	63	55	191.48	323.31	Q02952;Q02952-3;Q02952-2	A-kinase anchor protein 12
11	11	54.8	23.356	323.31	P09211	Glutathione S-transferase P
29	29	53.2	66.022	323.31	Q07065	Cytoskeleton-associated protein 4
33	33	50.9	89.321	323.31	P55072	Transitional endoplasmic reticulum ATPase
22	2	50.4	69.602	323.31	O60506;O60506-2	Heterogeneous nuclear ribonucleoprotein Q
31	31	50.2	72.932	323.31	P13667	Protein disulfide-isomerase A4
26	25	49.8	66.408	323.31	P20700	Lamin-B1
58	58	49.6	187.89	323.31	Q00610-2;Q00610	Clathrin heavy chain 1
87	76	49.3	280.01	323.31	P21333-2;P21333	Filamin-A
142	142	49.1	629.09	323.31	Q09666	Neuroblast differentiation-associated protein AHNAK
88	71	49.1	226.53	323.31	P35579;P35579-2	Myosin-9
44	28	48.2	113	323.31	P05023-4;P05023;P05023-3;P05023-2	Sodium/potassium-transporting ATPase subunit alpha-1
52	52	46.5	157.9	323.31	P42704	Leucine-rich PPR motif-containing protein, mitochondrial
60	58	46	189.25	323.31	P46940	Ras GTPase-activating-like protein IQGAP1
176	1	45.3	531.78	323.31	Q15149;Q15149-7;Q15149-8;Q15149-9;Q15149-5;Q15149-6	Plectin
87	87	43.2	273.42	323.31	P49327	Fatty acid synthase;[Acyl-carrier-protein] S-acetyltransferase;[Acyl-carrier-protein] S-malonyltransferase;3-oxoacyl-[acyl-carrier-protein] synthase;3-oxoacyl-[acyl-carrier-protein] reductase;3-hydroxyacyl-[acyl-carrier-protein] dehydratase;Enoyl-[acyl-carrier-protein] reductase;Oleoyl-[acyl-carrier-protein] hydrolase
48	48	42.8	152.45	323.31	Q9P2E9;Q9P2E9-3	Ribosome-binding protein 1
58	1	41.7	195.01	323.31	P16144-2;P16144-4;P16144-3	Integrin beta-4
46	46	39.3	164.34	323.31	sp|E24146|;CON__ENSEMBL:ENSBTAP00000024146	
8	8	39.1	12.074	323.31	P06454-2;P06454	Prothymosin alpha;Prothymosin alpha, N-terminally processed;Thymosin alpha-1
26	26	38.9	97.169	323.31	Q14974;Q14974-2	Importin subunit beta-1
72	72	37.1	285.09	323.31	Q13813-2;Q13813;Q13813-3	Spectrin alpha chain, non-erythrocytic 1
66	61	37	274.61	323.31	Q01082;Q01082-3;Q01082-2	Spectrin beta chain, non-erythrocytic 1
19	17	32.5	85.595	323.31	Q01813;Q01813-2	ATP-dependent 6-phosphofructokinase, platelet type

107	107	32.1	469.08	323.31	P78527;P78527-2	DNA-dependent protein kinase catalytic subunit
111	111	30.8	532.4	323.31	Q14204	Cytoplasmic dynein 1 heavy chain 1
8	3	50.3	21.768	316.49	P61586	Transforming protein RhoA
25	25	33.5	107.78	311.28	P55060-3;P55060;P55060-4	Exportin-2
67	67	36.1	273.6	305.4	Q6P2Q9	Pre-mRNA-processing-splicing factor 8
23	0	63.2	50.151	304.3	P68363;sp|E16242|;P68363-2	Tubulin alpha-1B chain
20	20	23.6	138.38	298	P28290;P28290-3;P28290-2	Sperm-specific antigen 2
82	82	49.3	233.47	291.7	Q9NZM1-6;Q9NZM1;Q9NZM1-3;Q9NZM1-2;Q9NZM1-5	Myoferlin
29	29	45.5	73.68	290.84	P38646	Stress-70 protein, mitochondrial
27	27	67.8	38.604	290.01	P07355;P07355-2;A6NMY6	Annexin A2;Putative annexin A2-like protein
82	72	45.4	275.66	285.03	O75369-2;O75369-9;O75369;O75369-8;O75369-6;O75369-3;O75369-7;O75369-5;O75369-4	Filamin-B
14	10	62.4	27.745	282.77	P63104;P63104-2	14-3-3 protein zeta/delta
70	2	28.5	377.13	280.99	Q14789-2;Q14789;Q14789-3	Golgin subfamily B member 1
21	21	61.6	38.714	279.94	P04083	Annexin A1
39	30	49.6	109.68	279.64	P33176	Kinesin-1 heavy chain
22	18	40.8	58.826	277.26	P13645;sp|P13645|;CON__P13645	Keratin, type I cytoskeletal 10
21	1	40	67.993	277.1	P08195;P08195-4	4F2 cell-surface antigen heavy chain
27	25	39.2	108.27	260.67	P29317	Ephrin type-A receptor 2
35	33	45.7	96.637	259.23	sp|FA79-17|;P14625	Endoplasmin
60	60	35	269.76	254.81	Q9Y490	Talin-1
21	9	40.3	66.217	254.37	Q9NVI7-2;Q9NVI7;Q9NVI7-3	ATPase family AAA domain-containing protein 3A
26	26	63.9	49.541	253.43	P49411	Elongation factor Tu, mitochondrial
25	25	41.1	90.98	252.74	P25205;P25205-2	DNA replication licensing factor MCM3
15	2	55.5	36.053	252.67	P04406	Glyceraldehyde-3-phosphate dehydrogenase
8	8	14.9	68.124	248.33	sp|P15636|;CON__P15636	
21	21	52.4	56.559	246.04	P06576	ATP synthase subunit beta, mitochondrial
17	17	60.6	36.112	243.28	P05198	Eukaryotic translation initiation factor 2 subunit 1
10	10	59.1	24.454	243.09	P37802-2;P37802	Transgelin-2
29	29	37.7	113.8	240.02	P22314-2;P22314	Ubiquitin-like modifier-activating enzyme 1
42	42	38.4	152.99	239.82	Q86UP2-4;Q86UP2;Q86UP2-2;Q86UP2-3	Kinectin
33	27	38.3	114.76	239.79	P16615;P16615-5;P16615-2;P16615-3;P16615-4	Sarcoplasmic/endoplasmic reticulum calcium ATPase 2
43	31	62.8	84.659	237.27	P07900;P07900-2	Heat shock protein HSP 90-alpha
17	1	53	38.746	232.95	P09651;P09651-3;Q32P51	Heterogeneous nuclear ribonucleoprotein A1;Heterogeneous nuclear ribonucleoprotein A1, N-terminally processed;Heterogeneous nuclear ribonucleoprotein A1-like 2
7	3	52	19.794	232.41	P19105;O14950	Myosin regulatory light chain 12A;Myosin regulatory light chain 12B
76	76	33.6	292.75	232.13	Q92616	Translational activator GCN1
24	17	65.2	50.663	228.67	P50395;P50395-2	Rab GDP dissociation inhibitor beta
25	25	54.2	57.924	228.52	P50991;P50991-2	T-complex protein 1 subunit delta
5	5	21.5	40.513	227.32	Q9UBS4	DnaJ homolog subfamily B member 11
48	48	31	238.26	221.46	Q14980;Q14980-2;Q14980-4;Q14980-3	Nuclear mitotic apparatus protein 1
21	21	43.6	66.726	220.96	Q9Y262;Q9Y262-2	Eukaryotic translation initiation factor 3 subunit L
26	26	60	57.488	216.06	P78371;P78371-2	T-complex protein 1 subunit beta
27	25	54.3	73.114	213.52	Q92945	Far upstream element-binding protein 2
24	24	34.2	90.583	211.87	Q00839;Q00839-2	Heterogeneous nuclear ribonucleoprotein U
21	1	58.4	49.83	211.81	P68371	Tubulin beta-4B chain
31	31	32.8	144.5	211.77	P41252	Isoleucine--tRNA ligase, cytoplasmic
28	12	55.2	70.051	211.24	P0DMV8;P0DMV9;P0DMV8-2	Heat shock 70 kDa protein 1A;Heat shock 70 kDa protein 1B
25	25	63.4	51.804	211.21	Q02790	Peptidyl-prolyl cis-trans isomerase FKBP4;Peptidyl-prolyl cis-trans isomerase FKBP4, N-terminally processed
24	24	42.6	75.378	208.26	P54136;P54136-2	Arginine--tRNA ligase, cytoplasmic

12	5	64.9	22.677	207.08	P62820;P62820-2;P62820-3	Ras-related protein Rab-1A
32	3	52.1	74.139	206.89	P02545;P02545-3;P02545-6;P02545-5;P02545-4	Prelamin-A/C;Lamin-A/C
24	14	27.7	130.62	206.86	P20020-6;P20020;P20020-4;P20020-1;P20020-5;P20020-2	Plasma membrane calcium-transporting ATPase 1
24	24	38.8	88.884	205.65	O75534;O75534-4;O75534-2;O75534-3	Cold shock domain-containing protein E1
22	22	41	82.999	204.84	P40939	Trifunctional enzyme subunit alpha, mitochondrial;Long-chain enoyl-CoA hydratase;Long chain 3-hydroxyacyl-CoA dehydrogenase
20	20	25.4	104.1	203.47	Q99613-2;Q99613;B5ME19	Eukaryotic translation initiation factor 3 subunit C;Eukaryotic translation initiation factor 3 subunit C-like protein
13	9	62.4	27.764	200.23	P27348	14-3-3 protein theta
35	34	51.5	72.332	198.99	P11021	78 kDa glucose-regulated protein
43	42	53.6	95.337	197.96	P13639	Elongation factor 2
22	22	33.1	76.613	196.35	P19338	Nucleolin
18	18	54.1	43.786	195.48	Q9UQ80;Q9UQ80-2	Proliferation-associated protein 2G4
22	22	24.6	135.58	194.88	Q15393;Q15393-3	Splicing factor 3B subunit 3
30	30	42.2	100.07	194.72	P35221;P35221-2;P35221-3	Catenin alpha-1
28	28	60.4	60.343	194.28	P17987	T-complex protein 1 subunit alpha
32	32	40.5	71.502	191.81	P27824-2;P27824;P27824-3	Calnexin
14	14	50.8	33.296	188.77	Q99623;Q99623-2	Prohibitin-2
20	20	42.1	63.836	188.63	Q86UE4	Protein LYRIC
32	1	75.5	44.105	188.46	P08727	Keratin, type I cytoskeletal 19
49	49	28.7	244.5	187.33	O75643	U5 small nuclear ribonucleoprotein 200 kDa helicase
13	3	42.4	53.341	185.42	sp|Q3MHN5|;CON__Q3MHN5	
10	7	50.5	24.336	184.72	P31947-2;P31947	14-3-3 protein sigma
16	16	40.4	52.771	184.3	P28838-2;P28838	Cytosol aminopeptidase
12	7	64.8	27.85	184.09	P31946-2;P31946	14-3-3 protein beta/alpha;14-3-3 protein beta/alpha, N-terminally processed
17	17	56.9	49.203	182.9	P17980	26S protease regulatory subunit 6A
20	20	32	96.771	182.18	Q8WUM4-2;Q8WUM4	Programmed cell death 6-interacting protein
19	19	44.1	51.83	181.81	Q01518-2;Q01518	Adenylyl cyclase-associated protein 1
23	19	76.4	39.42	180.86	P04075;P04075-2	Fructose-bisphosphate aldolase A
27	27	59.8	56.782	180.34	P30101	Protein disulfide-isomerase A3
30	29	41.9	125.54	179.9	O00410-3;O00410;O00410-2	Importin-5
34	29	64.7	70.897	179.46	P11142;P11142-2	Heat shock cognate 71 kDa protein
16	15	39.7	64.132	179.17	P14866;P14866-2	Heterogeneous nuclear ribonucleoprotein L
12	9	57.7	28.218	178.77	Q04917	14-3-3 protein eta
17	17	44.5	52.904	178.29	O00232;O00232-2	26S proteasome non-ATPase regulatory subunit 12
4	4	26.8	25.125	178.19	Q9BTT0-3;Q9BTT0	Acidic leucine-rich nuclear phosphoprotein 32 family member E
28	28	64.4	57.116	177.1	P07237	Protein disulfide-isomerase
11	11	39.2	40.542	176.44	Q9Y295	Developmentally-regulated GTP-binding protein 1
19	17	42.1	67.56	176.2	Q96AE4;Q96AE4-2	Far upstream element-binding protein 1
19	18	49.5	53.454	175.88	P34897-3;P34897;P34897-2	Serine hydroxymethyltransferase, mitochondrial
10	10	50.4	27.887	175.83	O14818;O14818-4;O14818-2	Proteasome subunit alpha type-7
15	15	73.9	31.236	172.49	Q13126;Q13126-4;Q13126-3;Q13126-2;Q13126-7;Q13126-6;Q13126-5	S-methyl-5-thioadenosine phosphorylase
25	25	42.8	82.704	171.58	P13010	X-ray repair cross-complementing protein 5
9	9	39.3	40.736	170.87	P55036;P55036-2	26S proteasome non-ATPase regulatory subunit 4
40	39	26.5	242.98	170.76	P27708	CAD protein;Glutamine-dependent carbamoyl-phosphate synthase;Aspartate carbamoyltransferase;Dihydroorotase
26	17	65.8	48.057	169.84	P05783	Keratin, type I cytoskeletal 18
27	27	38.6	107.77	169.8	P22102;P22102-2	Trifunctional purine biosynthetic protein adenosine-3;Phosphoribosylamine--glycine ligase;Phosphoribosylformylglycinamidine cyclo-ligase;Phosphoribosylglycinamide formyltransferase
23	23	36.7	94.622	168.51	P43243;P43243-2	Matrin-3
7	7	39.6	29.999	167.42	Q15691	Microtubule-associated protein RP/EB family member 1

23	23	33.3	105.84	165.93	Q99460;Q99460-2	26S proteasome non-ATPase regulatory subunit 1
16	16	32.3	76.096	165.31	O96005;O96005-4;O96005-3	Cleft lip and palate transmembrane protein 1
27	1	47.9	62.639	164.71	P31948;P31948-3	Stress-induced-phosphoprotein 1
5	5	22.4	39.933	163.78	Q96A49	Synapse-associated protein 1
10	8	31.3	45.671	162.14	P52597	Heterogeneous nuclear ribonucleoprotein F;Heterogeneous nuclear ribonucleoprotein F, N-terminally processed
26	26	34.4	122.29	162.03	Q14126	Desmoglein-2
34	24	50.7	69.412	161.21	P15311	Ezrin
12	12	53.3	28.768	160.68	P12004	Proliferating cell nuclear antigen
33	21	50.9	66.038	160.66	P04264;sp|P04264|;CON__P04264	Keratin, type II cytoskeletal 1
43	43	11.6	571.85	159.96	Q5T4S7-3;Q5T4S7-4;Q5T4S7;Q5T4S7-2	E3 ubiquitin-protein ligase UBR4
22	21	48.6	62.129	159.86	sp|P35527|;CON__P35527;P35527	Keratin, type I cytoskeletal 9
23	23	33	107.89	158.52	O60763;O60763-2	General vesicular transport factor p115
13	13	39.8	49.184	157.86	P62191;P62191-2	26S protease regulatory subunit 4
19	19	30.1	102.64	157.78	Q15424;Q15424-4;Q15424-3;Q15424-2	Scaffold attachment factor B1
36	36	17.8	331.77	156.41	P15924;P15924-2;P15924-3	Desmoplakin
46	46	23	267.29	155.99	P12270	Nucleoprotein TPR
27	27	27.4	126.97	154.28	Q16531	DNA damage-binding protein 1
22	22	41.5	79.467	153.55	P28331;P28331-2;P28331-4;P28331-5;P28331-3	NADH-ubiquinone oxidoreductase 75 kDa subunit, mitochondrial
14	14	22.7	85.034	153.45	Q96PD2;Q96PD2-2	Discoidin, CUB and LCCL domain-containing protein 2
12	3	41.1	40.84	153.03	P04439;P04439-2	HLA class I histocompatibility antigen, A-3 alpha chain
11	10	47.4	35.98	152.86	P32322-3;P32322;P32322-2	Pyrroline-5-carboxylate reductase 1, mitochondrial
24	24	34.2	111.33	151.9	Q9Y4L1;Q9Y4L1-2	Hypoxia up-regulated protein 1
5	5	43.9	17.259	151.46	Q04837	Single-stranded DNA-binding protein, mitochondrial
12	8	67.5	18.502	151.27	P23528	Cofilin-1
13	7	52.9	37.377	150.02	P62873;P62873-2	Guanine nucleotide-binding protein G(I)/G(S)/G(T) subunit beta-1
13	8	43.9	40.45	150.01	P04899;P04899-4;P04899-6;P04899-3;P04899-5;P04899-2	Guanine nucleotide-binding protein G(i) subunit alpha-2
30	1	51.5	95.337	149.67	Q12906;Q12906-5;Q12906-2;Q12906-3	Interleukin enhancer-binding factor 3
37	37	39.1	137.22	148.9	Q92896-2;Q92896;Q92896-3	Golgi apparatus protein 1
42	41	36.2	170.59	148.85	P07814	Bifunctional glutamate/proline--tRNA ligase;Glutamate--tRNA ligase;Proline--tRNA ligase
31	29	46.2	97.717	148.59	Q9Y678	Coatomer subunit gamma-1
12	12	44.6	34.932	148.14	P16422	Epithelial cell adhesion molecule
30	30	37.8	123.38	147.57	O14980	Exportin-1
22	22	44.5	69.284	146.97	P09960;P09960-4;P09960-2;P09960-3	Leukotriene A-4 hydrolase
15	15	39.7	67.723	146.45	P04844-2;P04844	Dolichyl-diphosphooligosaccharide--protein glycosyltransferase subunit 2
4	4	28.7	25.387	146.14	Q9BV86;Q9BV86-2	N-terminal Xaa-Pro-Lys N-methyltransferase 1;N-terminal Xaa-Pro-Lys N-methyltransferase 1, N-terminally processed
13	13	73.4	23.489	146.11	P51149	Ras-related protein Rab-7a
30	19	47.3	65.865	146.07	CON__P35908;sp|P35908|;P35908;CON__P35908v2	Keratin, type II cytoskeletal 2 epidermal
21	20	42.3	73.243	146.01	O00571;O00571-2;O15523-2;O15523	ATP-dependent RNA helicase DDX3X;ATP-dependent RNA helicase DDX3Y
27	27	49.6	80.109	145.35	Q12931;Q12931-2	Heat shock protein 75 kDa, mitochondrial
9	7	32.5	39.617	144.47	O75367;O75367-3;O75367-2	Core histone macro-H2A.1
12	12	41	38.534	143.43	Q9UJZ1;Q9UJZ1-2	Stomatin-like protein 2, mitochondrial
30	30	47.2	84.87	143.32	P02786	Transferrin receptor protein 1;Transferrin receptor protein 1, serum form
20	20	22.2	141.45	143.19	Q00341;Q00341-2	Vigilin
19	19	50.1	54.529	142.03	Q16658	Fascin
13	13	20.3	82.593	141.52	P46459;P46459-2	Vesicle-fusing ATPase
9	9	47.6	32.575	141.45	P06748;P06748-2;P06748-3	Nucleophosmin
37	25	27.3	232.53	141.07	P35580-4;P35580;P35580-3;P35580-2;P35580-5	Myosin-10
33	2	47.7	82.624	140.7	Q16891-2	MICOS complex subunit MIC60
19	19	62.5	41.428	140.67	P00558-2;P00558	Phosphoglycerate kinase 1

23	23	36.4	83.353	140.54	O95202	LETM1 and EF-hand domain-containing protein 1, mitochondrial
6	6	12.8	70.832	140.35	O43252	Bifunctional 3-phosphoadenosine 5-phosphosulfate synthase 1;Sulfate adenylyltransferase;Adenylyl-sulfate kinase
14	14	23.8	85.104	140.04	Q1KMD3	Heterogeneous nuclear ribonucleoprotein U-like protein 2
34	34	38.3	123.8	139.92	P18206;P18206-2	Vinculin
33	33	62.8	59.62	139.11	P50990;P50990-2;P50990-3	T-complex protein 1 subunit theta
25	25	27.6	119.09	139.05	P23229-4;P23229-2;P23229-9;P23229-6;P23229-5;P23229-3;P23229;P23229-7	Integrin alpha-6;Integrin alpha-6 heavy chain;Integrin alpha-6 light chain;Processed integrin alpha-6
6	1	42.1	18.871	138.26	P62633-8;P62633-4	Cellular nucleic acid-binding protein
12	6	63.2	23.545	138.23	P61019;P61019-2	Ras-related protein Rab-2A
11	11	48.4	35.611	138.12	O75821	Eukaryotic translation initiation factor 3 subunit G
25	22	52.9	56.108	137.39	P47895	Aldehyde dehydrogenase family 1 member A3
22	22	32.4	91.706	137.32	Q96QK1	Vacuolar protein sorting-associated protein 35
30	30	39.2	103.28	137.22	P55786;P55786-2	Puromycin-sensitive aminopeptidase
9	9	52.4	21.057	137.15	P30086	Phosphatidylethanolamine-binding protein 1;Hippocampal cholinergic neurostimulating peptide
23	23	26.3	109.44	136.57	Q14697-2;Q14697	Neutral alpha-glucosidase AB
38	38	39.4	140.96	135.65	Q08211	ATP-dependent RNA helicase A
26	23	37	93.133	135.55	P06737-2;P06737	Glycogen phosphorylase, liver form
12	9	78.7	23.482	135.44	P51148;P51148-2	Ras-related protein Rab-5C
19	19	25.1	88.583	134.83	Q9Y4W6	AFG3-like protein 2
22	22	40.5	76.715	134.69	P49915;P49915-2	GMP synthase [glutamine-hydrolyzing]
32	32	44.3	106.81	134.4	P49588;P49588-2	Alanine--tRNA ligase, cytoplasmic
13	13	78.8	26.922	133.94	O00299	Chloride intracellular channel protein 1
13	13	40.5	47.079	133.77	P22234;P22234-2	Multifunctional protein ADE2;Phosphoribosylaminoimidazole-succinocarboxamide synthase;Phosphoribosylaminoimidazole carboxylase
5	5	35.5	23.625	133.62	Q9HBH0;Q9HBH0-2	Rho-related GTP-binding protein RhoF
12	12	27.4	68.137	133.41	P27694	Replication protein A 70 kDa DNA-binding subunit;Replication protein A 70 kDa DNA-binding subunit, N-terminally processed
19	17	49.3	54.231	132.91	Q15233;Q15233-2	Non-POU domain-containing octamer-binding protein
17	6	30.9	72.572	132.44	Q5T9A4;Q5T9A4-3	ATPase family AAA domain-containing protein 3B
14	14	34.6	55.317	132.02	Q96A33-2;Q96A33	Coiled-coil domain-containing protein 47
16	16	41	59.256	131.87	P11413;P11413-2;P11413-3	Glucose-6-phosphate 1-dehydrogenase
23	10	54.5	59.366	131.73	Q99832;Q99832-3;Q99832-4	T-complex protein 1 subunit eta
15	15	41.5	51.872	130.75	P52209-2;P52209	6-phosphogluconate dehydrogenase, decarboxylating
21	21	35.4	74.976	130.31	Q8N392;Q8N392-2	Rho GTPase-activating protein 18
19	9	53.9	46.153	129.47	P60842;P60842-2	Eukaryotic initiation factor 4A-I
14	14	22.2	91.679	129.17	Q8NE71-2;Q8NE71	ATP-binding cassette sub-family F member 1
19	18	57.8	37.429	128.48	P22626;P22626-2	Heterogeneous nuclear ribonucleoproteins A2/B1
17	17	69.2	30.791	128.39	P60174;P60174-1;P60174-4	Triosephosphate isomerase
27	27	46.6	69.842	127.06	P12956;P12956-2	X-ray repair cross-complementing protein 6
31	31	33.5	145.83	126.52	O75533	Splicing factor 3B subunit 1
13	13	37.9	54.416	126.01	P26196	Probable ATP-dependent RNA helicase DDX6
21	20	58.4	45.626	124.56	P62195;P62195-2	26S protease regulatory subunit 8
21	18	33.8	96.695	124.32	P11216	Glycogen phosphorylase, brain form
11	11	28.2	60.966	124.08	P49023-2;P49023;P49023-3;P49023-4	Paxillin
26	26	41	92.48	123.97	P55884;P55884-2	Eukaryotic translation initiation factor 3 subunit B
19	19	30.6	99.045	123.67	P35606-2;P35606	Coatomer subunit beta
10	10	15.7	93.36	123.15	P13591-1;P13591;P13591-4;P13591-3;P13591-5;P13591-6	Neural cell adhesion molecule 1
10	10	30.2	56.769	121.33	Q9BZZ5-2;Q9BZZ5;Q9BZZ5-3;Q9BZZ5-5;Q9BZZ5-6;Q9BZZ5-1	Apoptosis inhibitor 5
2	2	14.8	24.551	121.29	P19388	DNA-directed RNA polymerases I, II, and III subunit RPABC1

14	12	43.7	57.221	120.94	P26599;P26599-2;P26599-3	Polypyrimidine tract-binding protein 1
21	21	46.7	57.136	119.74	P14868;P14868-2	Aspartate--tRNA ligase, cytoplasmic
10	10	20.6	60.103	119.21	O75475;O75475-3;O75475-2	PC4 and SFRS1-interacting protein
2	2	39.5	8.5435	119.06	O75506	Heat shock factor-binding protein 1
9	7	58.5	20.811	119.03	Q13185	Chromobox protein homolog 3
6	6	25.9	34.086	118.91	Q13190-2;Q13190;Q13190-3;Q13190-4	Syntaxin-5
7	7	51.7	16.837	118.86	P0DP25;P0DP24;P0DP23	
5	1	15.9	40.313	118.76	Q9BWF3;Q9BWF3-4;Q9BWF3-3;Q9BWF3-2	RNA-binding protein 4
3	3	37.5	12.259	117.82	P84090	Enhancer of rudimentary homolog
14	14	49.2	35.882	117.67	P09525;P09525-2	Annexin A4
9	9	51	24.205	117.65	P62241	40S ribosomal protein S8
16	16	73.2	35.076	117.49	P63244	Guanine nucleotide-binding protein subunit beta-2-like 1;Guanine nucleotide-binding protein subunit beta-2-like 1, N-terminally processed
46	35	20.9	358.2	117.31	P49792	E3 SUMO-protein ligase RanBP2
13	8	35.2	49.229	116.87	P31943	Heterogeneous nuclear ribonucleoprotein H;Heterogeneous nuclear ribonucleoprotein H, N-terminally processed
30	30	18.4	242.37	116.77	Q9H583	HEAT repeat-containing protein 1;HEAT repeat-containing protein 1, N-terminally processed
28	28	20.9	205.12	116.61	O15031	Plexin-B2
14	14	32.4	63.146	116.34	P06744;P06744-2	Glucose-6-phosphate isomerase
11	11	39.3	53.165	116.18	P23381;P23381-2	Tryptophan--tRNA ligase, cytoplasmic;T1-TrpRS;T2-TrpRS
16	3	57.2	38.631	116.05	P62136-2;P62136;P62136-3	Serine/threonine-protein phosphatase PP1-alpha catalytic subunit
19	1	59.5	43.135	115.99	Q8NC51-3;Q8NC51-4	Plasminogen activator inhibitor 1 RNA-binding protein
21	18	35.8	80.419	115.98	O95573	Long-chain-fatty-acid--CoA ligase 3
3	3	59.3	9.6138	115.76	O43504	Ragulator complex protein LAMTOR5
32	32	36.5	113.08	115.72	P09874	Poly [ADP-ribose] polymerase 1
20	1	41.9	78.099	115.57	O00429-4;O00429-2;O00429-9;O00429-8	Dynamin-1-like protein
18	18	40.4	67.877	115.53	P29401;P29401-2	Transketolase
16	16	35.2	50.315	115.44	P20073-2;P20073	Annexin A7
20	20	71.6	35.936	114.75	P08758	Annexin A5
14	14	43.7	36.375	114.48	P12429	Annexin A3
22	22	23.5	129.29	114.12	P17301	Integrin alpha-2
8	8	36.1	24.976	113.81	P49755	Transmembrane emp24 domain-containing protein 10
16	16	25.2	88.885	113.07	Q15459;Q15459-2	Splicing factor 3A subunit 1
4	4	8	73.953	112.63	Q9UNN5;Q9UNN5-2	FAS-associated factor 1
10	10	25.5	48.162	112.34	Q9Y6E2	Basic leucine zipper and W2 domain-containing protein 2
18	18	63	42.644	111.86	P12277	Creatine kinase B-type
5	1	14.8	49.35	110.93	Q8IUI8-2	Cytokine receptor-like factor 3
33	30	23.9	175.62	110.47	Q04637-8;Q04637-7;Q04637-5;Q04637-4;Q04637-3;Q04637;Q04637-9;Q04637-6	Eukaryotic translation initiation factor 4 gamma 1
11	11	54.7	28.804	110.38	P18669;P15259	Phosphoglycerate mutase 1;Phosphoglycerate mutase 2
31	31	48.2	101.89	110	P49736	DNA replication licensing factor MCM2
13	13	36.5	52.645	109.92	P31930	Cytochrome b-c1 complex subunit 1, mitochondrial
14	14	23.8	103.17	108.99	Q14157-4;Q14157-1;Q14157-3;Q14157;Q14157-5	Ubiquitin-associated protein 2-like
10	10	36.8	42.592	108.81	P39748;P39748-2	Flap endonuclease 1
21	21	32.8	103.14	107.71	Q8N163-2;Q8N163	Cell cycle and apoptosis regulator protein 2
25	21	43.3	85.496	107.7	P35222	Catenin beta-1
20	20	37.7	79.994	107.62	P17655;P17655-2	Calpain-2 catalytic subunit
13	13	23	88.972	107.09	P46087-2;P46087;P46087-4;P46087-3	Probable 28S rRNA (cytosine(4447)-C(5))-methyltransferase
12	12	34.6	52.837	106.96	Q9Y2T3-3;Q9Y2T3;Q9Y2T3-2	Guanine deaminase
16	14	44.8	58.162	106.79	Q16555-2;Q16555	Dihydropyrimidinase-related protein 2
10	10	29.2	51.353	106.58	Q9UNF0-2;Q9UNF0	Protein kinase C and casein kinase substrate in neurons protein 2

18	2	32.7	70.942	106.11	O43390;O43390-3	Heterogeneous nuclear ribonucleoprotein R
26	26	38.1	92.888	105.89	Q14566	DNA replication licensing factor MCM6
13	13	31.8	64.735	104.94	P38606-2;P38606	V-type proton ATPase catalytic subunit A
31	31	66.5	59.67	104.92	P48643;P48643-2	T-complex protein 1 subunit epsilon
20	12	41.6	75.491	104.86	P42166	Lamina-associated polypeptide 2, isoform alpha;Thymopoietin;Thymopentin
5	5	25.1	33.879	104.03	Q9NP79;Q9NP79-2	Vacuolar protein sorting-associated protein VTA1 homolog
16	16	44.6	66.193	103.86	O75083;O75083-3	WD repeat-containing protein 1
28	28	35.1	118.39	103.81	Q13308;Q13308-6;Q13308-4;Q13308-2;Q13308-3;Q13308-5	Inactive tyrosine-protein kinase 7
3	3	25.7	20.332	103.66	O00193	Small acidic protein
24	23	36.7	105.38	103.51	Q15029-2;Q15029;Q15029-3	116 kDa U5 small nuclear ribonucleoprotein component
5	5	21.2	30.394	103.45	O15260;O15260-2;O15260-3	Surfeit locus protein 4
4	4	15.6	42.07	103.28	Q9BX40;Q9BX40-2	Protein LSM14 homolog B
22	22	31.7	96.557	103.17	P33991	DNA replication licensing factor MCM4
38	35	19.5	304.1	103.17	Q14573	Inositol 1,4,5-trisphosphate receptor type 3
5	5	20.5	43.239	102.9	P18615;P18615-4;P18615-3	Negative elongation factor E
8	8	16.1	88.598	102.86	Q9NP58-4;Q9NP58	ATP-binding cassette sub-family B member 6, mitochondrial
7	7	41.5	20.9	102.8	P61081	NEDD8-conjugating enzyme Ubc12
12	9	51	23.548	102.71	P20340-2;P20340;P20340-4	Ras-related protein Rab-6A
15	15	17.5	141.54	102.71	Q9UQE7	Structural maintenance of chromosomes protein 3
6	6	19.3	51.212	102.6	Q13451;Q13451-2	Peptidyl-prolyl cis-trans isomerase FKBP5;Peptidyl-prolyl cis-trans isomerase FKBP5, N-terminally processed
12	12	45.9	34.93	102.37	Q96AG4	Leucine-rich repeat-containing protein 59
8	7	23.9	49.027	102.14	P34896-2;P34896;P34896-3;P34896-4	Serine hydroxymethyltransferase, cytosolic
16	16	55.4	48.141	101.87	P27797	Calreticulin
9	9	63.2	27.335	101.7	Q9UKY7;Q9UKY7-2	Protein CDV3 homolog
25	25	30	123.03	101.54	Q92900-2;Q92900	Regulator of nonsense transcripts 1
13	13	57.2	33.932	101.29	Q9NUJ1;Q9NUJ1-2	Mycophenolic acid acyl-glucuronide esterase, mitochondrial
6	2	32.7	23.467	101.29	P63000-2;P63000;P60763	Ras-related C3 botulinum toxin substrate 1;Ras-related C3 botulinum toxin substrate 3
2	2	17.2	22.092	101.16	O43583	Density-regulated protein
26	26	47.2	73.62	100.86	P52272-2;P52272	Heterogeneous nuclear ribonucleoprotein M
20	20	41.8	59.75	100.37	P25705;P25705-2;P25705-3	ATP synthase subunit alpha, mitochondrial
23	23	19.2	168.59	100.37	Q8TEQ6	Gem-associated protein 5
18	18	46.3	59.177	100.1	P14314-2;P14314	Glucosidase 2 subunit beta
8	7	51.3	21.308	99.922	P84095	Rho-related GTP-binding protein RhoG
28	28	56.5	60.533	99.897	P49368;P49368-2	T-complex protein 1 subunit gamma
26	26	19.1	213.7	99.891	Q5JRA6;Q5JRA6-2	Melanoma inhibitory activity protein 3
29	29	41.5	102	99.888	Q7KZF4	Staphylococcal nuclease domain-containing protein 1
29	29	39.7	107.14	99.846	P53618	Coatomer subunit beta
9	9	20.4	75.022	99.785	O94776	Metastasis-associated protein MTA2
10	10	88.6	15.054	99.39	P07737	Profilin-1
24	19	26.3	121.9	99.288	O60264	SWI/SNF-related matrix-associated actin-dependent regulator of chromatin subfamily A member 5
5	5	30.5	31.362	98.683	Q07021	Complement component 1 Q subcomponent-binding protein, mitochondrial
12	12	46.5	41.487	98.302	Q15019;Q15019-3;Q15019-2	Septin-2
2	2	17.3	18.884	98.289	P23434	Glycine cleavage system H protein, mitochondrial
10	10	71.9	15.164	98.077	Q01469	Fatty acid-binding protein, epidermal
13	13	41.7	45.803	97.317	O60664-4;O60664-3;O60664	Perilipin-3
19	19	29.8	93.487	97.154	Q8N1F7;Q8N1F7-2	Nuclear pore complex protein Nup93
6	6	21.2	36.813	96.904	Q8TCT9-5;Q8TCT9;Q8TCT9-2;Q8TCT9-4	Minor histocompatibility antigen H13
27	25	40.6	87.343	96.388	Q9NR30;Q9NR30-2	Nucleolar RNA helicase 2
14	14	20.9	89.834	96.187	Q96GQ7	Probable ATP-dependent RNA helicase DDX27
21	21	48.2	51.156	96.059	Q9Y230;Q9Y230-2	RuvB-like 2

10	9	41.5	39.594	95.719	Q9NYL9	Tropomodulin-3
21	21	33.7	101.11	95.448	P56192;P56192-2	Methionine--tRNA ligase, cytoplasmic
19	15	43.5	58.024	95.231	P40227;P40227-2	T-complex protein 1 subunit zeta
26	26	46.5	75.872	94.819	P08133;P08133-2	Annexin A6
13	13	40.3	49.973	94.681	P13489	Ribonuclease inhibitor
21	15	42.8	70.67	94.633	P11940;P11940-2	Polyadenylate-binding protein 1
18	18	37.1	90.067	94.616	Q86XP3-2;Q86XP3	ATP-dependent RNA helicase DDX42
8	8	38.4	31.089	94.575	Q9NTX5-6;Q9NTX5-2;Q9NTX5;Q9NTX5-3	Ethylmalonyl-CoA decarboxylase
7	7	21.4	56.806	94.405	P30419;P30419-2	Glycylpeptide N-tetradecanoyltransferase 1
32	32	35.1	120.84	93.948	P53396;P53396-2;P53396-3	ATP-citrate synthase
29	29	28.2	136.37	93.936	Q86VP6;Q86VP6-2	Cullin-associated NEDD8-dissociated protein 1
21	21	24.5	134.46	93.576	Q9P2J5;Q9P2J5-2	Leucine--tRNA ligase, cytoplasmic
14	14	32.7	69.15	92.95	P23588;P23588-2	Eukaryotic translation initiation factor 4B
10	10	28	71.423	92.853	Q9NZT2-2;Q9NZT2	Opioid growth factor receptor
8	8	30.2	37.893	92.745	O96008;O96008-2	Mitochondrial import receptor subunit TOM40 homolog
10	10	16.7	91.838	92.604	Q09161	Nuclear cap-binding protein subunit 1
16	16	58	35.503	92.597	P40926;P40926-2	Malate dehydrogenase, mitochondrial
18	18	27.8	93.307	92.581	P45974-2;P45974	Ubiquitin carboxyl-terminal hydrolase 5
13	13	40	47.366	92.424	P43686;P43686-2	26S protease regulatory subunit 6B
19	19	42.9	66.049	92.357	O00567	Nucleolar protein 56
9	9	26.1	44.76	91.569	P61160;P61160-2	Actin-related protein 2
11	7	52.4	24.763	91.473	P24534	Elongation factor 1-beta
20	20	46.5	63.541	90.777	P46060	Ran GTPase-activating protein 1
11	10	61.9	32.922	90.519	P52907	F-actin-capping protein subunit alpha-1
7	7	19.2	53.29	90.506	Q16204	Coiled-coil domain-containing protein 6
15	15	15	138.91	90.403	O60610-2;O60610-3;O60610	Protein diaphanous homolog 1
25	24	41.8	87.798	90.317	P47897;P47897-2	Glutamine--tRNA ligase
13	13	38.4	57.861	90.276	P52292	Importin subunit alpha-1
13	12	22.8	87.644	90.132	Q9H4G0-2;Q9H4G0;Q9H4G0-4;Q9H4G0-3	Band 4.1-like protein 1
10	10	48.8	24.279	89.931	Q9HAV7	GrpE protein homolog 1, mitochondrial
6	6	72.1	10.192	89.775	P14854	Cytochrome c oxidase subunit 6B1
6	6	49.5	11.74	89.564	P31949	Protein S100-A11;Protein S100-A11, N-terminally processed
47	47	21.4	358.69	89.552	P46013;P46013-2	Antigen KI-67
7	7	80.8	11.662	89.48	Q9HCY8	Protein S100-A14
5	5	19.5	36.648	89.252	O15127	Secretory carrier-associated membrane protein 2
27	27	22.7	184.82	89.072	P26358-2;P26358;P26358-3	DNA (cytosine-5)-methyltransferase 1
6	6	63.6	14.515	89.056	P25398	40S ribosomal protein S12
19	19	20	150.83	88.969	Q29RF7	Sister chromatid cohesion protein PDS5 homolog A
13	13	25.2	75.981	88.884	Q96TA2-3;Q96TA2-2;Q96TA2	ATP-dependent zinc metalloprotease YME1L1
3	3	20.7	19.291	88.799	Q9GZN8;Q9GZN8-2	UPF0687 protein C20orf27
18	18	39.8	51.242	88.692	P50995-2;P50995	Annexin A11
13	13	41.3	32.337	88.599	P07910-2;P07910-4;P07910	Heterogeneous nuclear ribonucleoproteins C1/C2
40	40	31.3	166.57	88.465	Q14152;Q14152-2	Eukaryotic translation initiation factor 3 subunit A
13	13	18.8	103.64	88.297	P55265-5;P55265;P55265-4;P55265-3;P55265-2	Double-stranded RNA-specific adenosine deaminase
10	7	28.9	41.389	88.234	P28482;P28482-2	Mitogen-activated protein kinase 1
7	7	12.6	103.7	88.205	Q8IWX8	Calcium homeostasis endoplasmic reticulum protein
15	15	50.3	42.945	87.804	Q9UNM6;Q9UNM6-2	26S proteasome non-ATPase regulatory subunit 13
16	16	53.4	29.945	87.55	P61247	40S ribosomal protein S3a
5	5	19.6	35.238	86.95	P31942-2;P31942;P31942-3	Heterogeneous nuclear ribonucleoprotein H3
20	20	50.2	64.615	86.169	P31939;P31939-2	Bifunctional purine biosynthesis protein PURH;Phosphoribosylaminoimidazolecarboxamide formyltransferase;IMP cyclohydrolase

11	11	35.1	45.199	86.136	P24752	Acetyl-CoA acetyltransferase, mitochondrial
6	6	20.2	44.243	86.081	Q15007;Q15007-2	Pre-mRNA-splicing regulator WTAP
19	19	55	48.633	86.05	P35998;P35998-2	26S protease regulatory subunit 7
6	6	14.2	80.64	85.997	P06396-2;P06396-4;P06396-3;P06396;sp|Q3SX14|;CON__Q3SX14	Gelsolin
8	2	30	33.488	85.354	Q01105	Protein SET
21	20	38.5	76.149	84.827	P23246;P23246-2	Splicing factor, proline- and glutamine-rich
10	10	30	43.476	84.703	Q6NZI2;Q6NZI2-2	Polymerase I and transcript release factor
11	6	33.3	40.361	84.603	P63096;P63096-2	Guanine nucleotide-binding protein G(i) subunit alpha-1
29	29	15.2	274.37	84.337	P11717	Cation-independent mannose-6-phosphate receptor
9	9	29.1	49.512	84.135	O14929;O14929-2	Histone acetyltransferase type B catalytic subunit
13	13	23.3	82.845	84.003	P49589-2;P49589;P49589-3	Cysteine--tRNA ligase, cytoplasmic
19	19	51.1	51.028	83.552	P61978-2;P61978;P61978-3	Heterogeneous nuclear ribonucleoprotein K
9	9	35.1	40.282	83.437	Q12792;Q12792-3;Q12792-4	Twinfilin-1
29	29	21.1	218	83.311	Q14839;Q14839-2	Chromodomain-helicase-DNA-binding protein 4
13	11	24.1	86.16	83.178	Q15436;Q15436-2	Protein transport protein Sec23A
9	9	22.5	63.472	83.108	Q8N1G4	Leucine-rich repeat-containing protein 47
6	6	30.5	23.671	83.064	P82979	SAP domain-containing ribonucleoprotein
15	15	58.5	29.804	82.912	P35232	Prohibitin
5	5	55.8	13.291	82.224	P62318-2;P62318	Small nuclear ribonucleoprotein Sm D3
10	10	33.5	42.449	82.098	Q9HB07	UPF0160 protein MYG1, mitochondrial
19	19	61	50.227	81.932	Q9Y265;Q9Y265-2	RuvB-like 1
23	23	30.6	88.414	81.841	P05556;P05556-2;P05556-5;P05556-4;P05556-3	Integrin beta-1
6	6	30.6	37.157	81.611	O75781-2;O75781	Paralemmin-1
17	17	35.8	61.397	81.591	P00367;P00367-3;P00367-2;P49448	Glutamate dehydrogenase 1, mitochondrial;Glutamate dehydrogenase 2, mitochondrial
15	15	55.4	37.489	81.478	Q9UHD1;Q9UHD1-2	Cysteine and histidine-rich domain-containing protein 1
12	12	39	46.247	81.293	P17174;P17174-2	Aspartate aminotransferase, cytoplasmic
12	12	23.5	75.775	81.223	Q99805	Transmembrane 9 superfamily member 2
18	18	34.2	79.685	81.003	P51659;P51659-3;P51659-2	Peroxisomal multifunctional enzyme type 2;(3R)-hydroxyacyl-CoA dehydrogenase;Enoyl-CoA hydratase 2
3	1	9.8	48.872	80.849	Q14141-2;Q14141-4;Q14141	Septin-6
7	7	41.4	29.483	80.767	P25789;P25789-2	Proteasome subunit alpha type-4
7	7	22.2	61.89	80.475	P40222	Alpha-taxilin
34	34	33.9	140.47	80.321	P26640	Valine--tRNA ligase
7	7	35.8	26.888	80.298	Q86V81	THO complex subunit 4
14	14	28	82.588	80.174	Q9NY33;Q9NY33-4	Dipeptidyl peptidase 3
22	22	31.8	101.27	79.885	Q9BXJ9	N-alpha-acetyltransferase 15, NatA auxiliary subunit
18	18	33.2	85.862	79.651	Q12797;Q12797-10	Aspartyl/asparaginyl beta-hydroxylase
18	16	32.2	82.262	79.644	Q9ULC5-3;Q9ULC5;Q9ULC5-4	Long-chain-fatty-acid--CoA ligase 5
27	26	41.3	69.948	79.641	Q03252	Lamin-B2
20	20	16.1	187.37	79.638	CON__Q2UVX4;sp|Q2UVX4|	
11	11	42.9	55.18	79.596	Q9UMS4	Pre-mRNA-processing factor 19
29	14	36.7	121	79.506	P27816;P27816-6;P27816-2	Microtubule-associated protein 4
9	9	42.5	26.788	79.379	P51858;P51858-2;P51858-3	Hepatoma-derived growth factor
12	9	56.7	37.497	79.189	Q15365	Poly(rC)-binding protein 1
3	3	44.8	6.4575	79.137	Q96IX5	Up-regulated during skeletal muscle growth protein 5

24	24	35.7	108.17	79.075	O60716;O60716-9;O60716-11;O60716-18;O60716-10;O60716-19;O60716-21;O60716-13;O60716-17;O60716-5;O60716-3;O60716-2;O60716-7;O60716-6;O60716-12;O60716-14;O60716-4;O60716-8;O60716-15;O60716-16;O60716-20;O60716-22;O60716-23;O60716-24;O60716-25;O60716-26;O60716-27;O60716-29;O60716-31;O60716-28;O60716-30;O60716-32	Catenin delta-1
6	6	60.4	17.328	79.061	Q99471;Q99471-2;Q99471-3	Prefoldin subunit 5
19	19	48.5	68.587	78.632	CON__Q3SZ57;sp|Q3SZ57|	
6	6	50.4	14.665	78.338	Q9NX55	Huntingtin-interacting protein K
31	31	34.2	136.11	78.321	Q7L2E3-2;Q7L2E3-3;Q7L2E3	Putative ATP-dependent RNA helicase DHX30
10	10	20.6	81.224	78.257	P13798	Acylamino-acid-releasing enzyme
22	22	28.6	105.79	78.166	Q6UB35	Monofunctional C1-tetrahydrofolate synthase, mitochondrial
22	20	30.3	101.08	77.995	P19367-4;P19367-2;P19367;P19367-3	Hexokinase-1
1	1	20.9	9.9374	77.779	Q9Y4Y9	U6 snRNA-associated Sm-like protein LSm5
10	10	18.9	76.546	77.526	Q6P996-3;Q6P996-4;Q6P996-5;Q6P996;Q6P996-2	Pyridoxal-dependent decarboxylase domain-containing protein 1
17	14	27.3	102.35	77.446	Q92973;Q92973-2;Q92973-3	Transportin-1
10	10	51.9	26.411	77.429	P28066;P28066-2	Proteasome subunit alpha type-5
21	21	46.2	65.401	77.223	Q9UHD8;Q9UHD8-7;Q9UHD8-2;Q9UHD8-5;Q9UHD8-3;Q9UHD8-4;Q9UHD8-9;Q9UHD8-8	Septin-9
14	14	38.8	52.164	77.162	Q13283	Ras GTPase-activating protein-binding protein 1
21	13	42.2	50.184	76.976	Q5VTE0;P68104;sp|118764358|;P68104-2	Putative elongation factor 1-alpha-like 3;Elongation factor 1-alpha 1
14	14	26.2	91.417	76.862	P22223;P22223-2	Cadherin-3
13	13	42.9	50.227	76.849	P80303-2;P80303	Nucleobindin-2;Nesfatin-1
9	9	14.3	93.955	76.667	Q9P246-2;Q9P246;Q9P246-3	Stromal interaction molecule 2
17	17	32.4	62.846	76.564	Q969V3-2;Q969V3	Nicalin
18	11	51.9	50.582	76.54	P31150	Rab GDP dissociation inhibitor alpha
15	15	40.2	46.108	76.49	P39023	60S ribosomal protein L3
7	7	38	28.415	76.337	P09661	U2 small nuclear ribonucleoprotein A
19	19	35.5	76.689	76.279	P16435	NADPH--cytochrome P450 reductase
29	29	38.4	101.56	76.08	P11586	C-1-tetrahydrofolate synthase, cytoplasmic;Methylenetetrahydrofolate dehydrogenase;Methenyltetrahydrofolate cyclohydrolase;Formyltetrahydrofolate synthetase;C-1-tetrahydrofolate synthase, cytoplasmic, N-terminally processed
18	18	25.2	112.59	76.037	O43491;O43491-4;O43491-3;O43491-2	Band 4.1-like protein 2
21	21	25.8	114.71	75.794	O14617-4;O14617;O14617-5;O14617-2;O14617-3	AP-3 complex subunit delta-1
17	17	19	138.83	75.791	O60841	Eukaryotic translation initiation factor 5B
11	11	41.2	33.969	75.572	P23193;P23193-2	Transcription elongation factor A protein 1
4	4	53	11.665	75.496	P05387	60S acidic ribosomal protein P2
6	4	22.1	33.784	75.305	P22087	rRNA 2-O-methyltransferase fibrillarin
26	26	43.4	83.434	75.245	P26639;P26639-2	Threonine--tRNA ligase, cytoplasmic
11	11	52.9	24.68	75.109	Q92520	Protein FAM3C
17	17	42.3	67.454	75.101	O94826	Mitochondrial import receptor subunit TOM70
27	18	44.2	67.819	74.8	P26038	Moesin
16	16	30.3	90.069	74.646	P23921	Ribonucleoside-diphosphate reductase large subunit
16	16	32.2	68.047	74.62	Q15046;Q15046-2	Lysine--tRNA ligase
13	0	30.3	75.278	74.087	sp|GST-p62|	
7	6	30.8	43.171	73.935	P54727;P54727-2	UV excision repair protein RAD23 homolog B
15	15	37.1	56.65	73.688	O43175	D-3-phosphoglycerate dehydrogenase

8	8	13.9	84.468	73.289	Q5QJE6	Deoxynucleotidyltransferase terminal-interacting protein 2
14	13	42.3	39.594	73.086	P51991;P51991-2	Heterogeneous nuclear ribonucleoprotein A3
16	16	25.4	88.367	72.828	P50416;P50416-2	Carnitine O-palmitoyltransferase 1, liver isoform
13	13	32.7	56.598	72.818	Q15758;Q15758-3;Q15758-2	Neutral amino acid transporter B(0)
12	12	44.3	38.418	72.657	CON__P12763;sp|P12763|	
7	4	23.1	49.398	72.085	Q9NVA2;Q9NVA2-2	Septin-11
22	22	37.6	81.307	72.017	P33993;P33993-3	DNA replication licensing factor MCM7
8	8	39.9	31.284	71.99	Q96FW1	Ubiquitin thioesterase OTUB1
25	13	33.5	105.69	71.907	P63010-2;P63010;P63010-3	AP-2 complex subunit beta
14	14	27.2	62.942	71.88	O43776	Asparagine--tRNA ligase, cytoplasmic
5	4	47.6	21.418	71.817	P83916	Chromobox protein homolog 1
16	16	44.5	58.14	71.672	O15371-2;O15371-3;O15371	Eukaryotic translation initiation factor 3 subunit D
16	16	16.4	131.98	71.668	O43795;O43795-2	Unconventional myosin-Ib
9	9	15.1	98.767	71.569	Q92888-2;Q92888;Q92888-3;Q92888-4	Rho guanine nucleotide exchange factor 1
17	17	41.6	47.517	71.5	P00505;P00505-2	Aspartate aminotransferase, mitochondrial
9	9	33.6	42.1	71.445	P36952	Serpin B5
27	27	28	148.85	71.363	Q9BQG0;Q9BQG0-2	Myb-binding protein 1A
8	8	18	56.92	71.288	Q96JB5;Q96JB5-4;Q96JB5-2	CDK5 regulatory subunit-associated protein 3
8	8	38.6	24.593	70.847	O75396	Vesicle-trafficking protein SEC22b
11	11	46.1	28.994	70.829	P50402	Emerin
39	39	12	506.27	70.798	Q14517	Protocadherin Fat 1;Protocadherin Fat 1, nuclear form
8	8	16.1	82.004	70.781	Q9BVJ6-3;Q9BVJ6;Q9BVJ6-2	U3 small nucleolar RNA-associated protein 14 homolog A
7	7	26.7	30.588	70.685	Q99729-3;Q99729-2;Q99729-4;Q99729	Heterogeneous nuclear ribonucleoprotein A/B
16	16	44.2	48.442	70.651	P22695	Cytochrome b-c1 complex subunit 2, mitochondrial
10	10	45	34.559	70.608	Q15785	Mitochondrial import receptor subunit TOM34
24	20	43.8	81.744	70.471	P14923	Junction plakoglobin
26	26	38.2	98.149	70.418	P78344-2;P78344	Eukaryotic translation initiation factor 4 gamma 2
3	3	29.1	15.314	70.324	Q9NQP4	Prefoldin subunit 4
7	7	37	23.772	70.265	O95816;O95816-2	BAG family molecular chaperone regulator 2
8	8	37.9	31.387	70.214	P30084	Enoyl-CoA hydratase, mitochondrial
14	14	37.3	46.44	70.099	P50454	Serpin H1
14	14	25.5	96.557	69.984	Q8IY81	pre-rRNA processing protein FTSJ3
18	18	34.5	72.926	69.847	P49748-3;P49748;P49748-2	Very long-chain specific acyl-CoA dehydrogenase, mitochondrial
16	16	27.2	89.034	69.709	Q12788	Transducin beta-like protein 3
38	0	10.1	614.15	69.655	Q9UPN3-3;Q9UPN3-2	Microtubule-actin cross-linking factor 1, isoforms 1/2/3/5
13	13	26.3	68.119	69.531	Q8TAT6;Q8TAT6-2	Nuclear protein localization protein 4 homolog
23	23	25.5	119.91	69.521	Q9Y5B9	FACT complex subunit SPT16
10	7	36.1	28.585	69.163	P39687	Acidic leucine-rich nuclear phosphoprotein 32 family member A
9	8	30.7	32.834	68.982	Q14103-3;Q14103;Q14103-4;Q14103-2	Heterogeneous nuclear ribonucleoprotein D0
16	16	17.9	117.97	68.911	P14735;P14735-2	Insulin-degrading enzyme
12	12	42.6	43.062	68.862	Q12905	Interleukin enhancer-binding factor 2
6	6	15.7	62.507	68.812	Q01844-6;Q01844-3;Q01844;Q01844-5;Q01844-2	RNA-binding protein EWS
14	14	40.2	46.836	68.73	P05455	Lupus La protein
12	12	37	59.271	68.276	O15355	Protein phosphatase 1G
9	9	35.5	41.268	68.251	P52788;P52788-2	Spermine synthase
18	18	36.5	58.397	68.212	O43278;O43278-2	Kunitz-type protease inhibitor 1
33	33	35.6	138.34	67.876	P53621;P53621-2	Coatomer subunit alpha;Xenin;Proxenin
9	9	78.6	16.014	67.863	Q9UK76;Q9UK76-2;Q9UK76-3	Hematological and neurological expressed 1 protein;Hematological and neurological expressed 1 protein, N-terminally processed
11	11	32.8	44.468	67.728	Q16543	Hsp90 co-chaperone Cdc37;Hsp90 co-chaperone Cdc37, N-terminally processed

8	8	34.6	32.233	67.607	Q14165	Malectin
6	6	41.8	20.198	67.523	P61923;P61923-4;P61923-5;P61923-3	Coatomer subunit zeta-1
8	8	31.6	41.92	67.489	Q99536;Q99536-3;Q99536-2	Synaptic vesicle membrane protein VAT-1 homolog
29	29	25.9	165.57	67.382	P33527-3;P33527;P33527-9;P33527-7;P33527-5;P33527-4;P33527-2;P33527-8;P33527-6	Multidrug resistance-associated protein 1
14	14	26.2	92.25	67.105	Q99459	Cell division cycle 5-like protein
17	17	16.6	150.56	67.071	Q9UKV3-5;Q9UKV3;Q9UKV3-3;Q9UKV3-2	Apoptotic chromatin condensation inducer in the nucleus
11	11	41.3	36.701	67.033	O75381-2;O75381	Peroxisomal membrane protein PEX14
14	14	71.8	22.237	66.835	O43399;O43399-5;O43399-7;O43399-2;O43399-4;O43399-3;O43399-6	Tumor protein D54
15	15	20.5	119.52	66.815	O95373	Importin-7
4	4	25.4	23.754	66.605	Q9Y3B8-3;Q9Y3B8-2;Q9Y3B8	Oligoribonuclease, mitochondrial
11	11	69.3	22.782	66.544	P04792	Heat shock protein beta-1
7	3	48.7	17.965	66.5	P62979	Ubiquitin-40S ribosomal protein S27a;Ubiquitin;40S ribosomal protein S27a
11	11	55.8	25.035	66.484	P30041	Peroxiredoxin-6
7	7	27.8	29.995	66.384	P62424	60S ribosomal protein L7a
16	16	34.2	72.911	66.293	O00116	Alkyldihydroxyacetonephosphate synthase, peroxisomal
13	13	26.3	75.406	66.185	Q9NVP1	ATP-dependent RNA helicase DDX18
5	5	68.7	19.529	66.01	P49006	MARCKS-related protein
19	19	29.1	85.424	65.937	Q99798	Aconitate hydratase, mitochondrial
12	12	50.2	32.118	65.916	P00491	Purine nucleoside phosphorylase
19	19	43.1	71.428	65.826	Q16643;Q16643-3;Q16643-2	Drebrin
8	8	25	39.234	65.716	CON__P00978;sp|P00978|	
12	12	26.9	78.365	65.615	Q14444;Q14444-2	Caprin-1
13	13	58.6	38.89	65.387	Q15293;Q15293-2	Reticulocalbin-1
13	2	21.7	81.537	65.127	P16070;P16070-17;P16070-5;P16070-3;P16070-4;P16070-6;P16070-10;P16070-16;P16070-8;P16070-13;P16070-11;P16070-14;P16070-7;P16070-12;P16070-9	CD44 antigen
12	12	36.6	47.837	64.735	Q15084-3;Q15084;Q15084-4;Q15084-5;Q15084-2	Protein disulfide-isomerase A6
5	5	15	55.385	64.703	O00592-2;O00592	Podocalyxin
4	4	48.6	15.521	64.535	Q9NS69	Mitochondrial import receptor subunit TOM22 homolog
18	18	26.5	117.8	64.463	P42285	Superkiller viralicidic activity 2-like 2
14	14	32.8	59.143	64.445	P54577	Tyrosine--tRNA ligase, cytoplasmic;Tyrosine--tRNA ligase, cytoplasmic, N-terminally processed
12	12	26.9	48.755	64.398	P36957;P36957-2	Dihydrolipoyllysine-residue succinyltransferase component of 2-oxoglutarate dehydrogenase complex, mitochondrial
10	10	74.1	17.031	64.364	P30044-2;P30044;P30044-3;P30044-4	Peroxiredoxin-5, mitochondrial
25	25	38	80.852	64.252	P43304;P43304-2	Glycerol-3-phosphate dehydrogenase, mitochondrial
15	15	36.5	46.103	64.231	sp|P34955|;CON__P34955	
2	2	34.1	8.9687	64.073	Q9Y5U9	Immediate early response 3-interacting protein 1
17	17	36.7	70.729	63.977	Q9UHB9;Q9UHB9-4;Q9UHB9-2	Signal recognition particle subunit SRP68
2	2	23.1	16.859	63.8	Q9NRF9	DNA polymerase epsilon subunit 3
20	20	20.2	148.66	63.528	Q9UM54-6;Q9UM54-5;Q9UM54-2;Q9UM54-4;Q9UM54-1;Q9UM54	Unconventional myosin-VI
18	18	35.1	66.294	63.443	O95831-3;O95831;O95831-2;O95831-5	Apoptosis-inducing factor 1, mitochondrial
21	21	27.1	122.85	63.137	Q9BSJ8;Q9BSJ8-2	Extended synaptotagmin-1
16	1	25.9	98.9	63.037	A0FGR8-2;A0FGR8;A0FGR8-4	Extended synaptotagmin-2
12	12	63	32.66	62.995	Q15181	Inorganic pyrophosphatase
22	16	36.9	80.272	62.962	Q92841;Q92841-1;Q92841-3;Q92841-2	Probable ATP-dependent RNA helicase DDX17
10	10	35.1	48.207	62.803	Q9UJU6;Q9UJU6-2;Q9UJU6-3;Q9UJU6-5;Q9UJU6-6;Q9UJU6-4	Drebrin-like protein

7	7	22.3	29.668	62.598	P47985;P0C7P4	Cytochrome b-c1 complex subunit Rieske, mitochondrial;Cytochrome b-c1 complex subunit 11;Putative cytochrome b-c1 complex subunit Rieske-like protein 1
15	15	42.5	44.272	62.291	Q8NBJ4-2;Q8NBJ4	Golgi membrane protein 1
29	29	18.2	208.7	62.156	Q14690	Protein RRP5 homolog
10	10	33.4	37.895	62.126	P13995;P13995-2	Bifunctional methylenetetrahydrofolate dehydrogenase/cyclohydrolase, mitochondrial;NAD-dependent methylenetetrahydrofolate dehydrogenase;Methenyltetrahydrofolate cyclohydrolase
9	9	40.1	34.273	62.081	P05388;P05388-2;Q8NHW5	60S acidic ribosomal protein P0;60S acidic ribosomal protein P0-like
15	11	25	94.511	61.94	O95757	Heat shock 70 kDa protein 4L
26	26	15.6	218.52	61.805	Q14008-2;Q14008;Q14008-3	Cytoskeleton-associated protein 5
18	18	43	50.151	61.772	P36551	Oxygen-dependent coproporphyrinogen-III oxidase, mitochondrial
12	12	27	72.691	61.7	P31040;P31040-2;P31040-3	Succinate dehydrogenase [ubiquinone] flavoprotein subunit, mitochondrial
13	13	24.6	82.688	61.574	Q32MZ4-3;Q32MZ4-2;Q32MZ4	Leucine-rich repeat flightless-interacting protein 1
16	14	37	65.308	61.026	P30153	Serine/threonine-protein phosphatase 2A 65 kDa regulatory subunit A alpha isoform
25	25	16.9	205.11	60.848	Q8TEM1	Nuclear pore membrane glycoprotein 210
24	24	13.7	299.61	60.822	Q9UQ35;Q9UQ35-2	Serine/arginine repetitive matrix protein 2
7	7	30.9	35.979	60.791	Q9P287;Q9P287-3;Q9P287-4;Q9P287-2	BRCA2 and CDKN1A-interacting protein
13	13	49.7	38.438	60.515	Q9Y3F4;Q9Y3F4-2	Serine-threonine kinase receptor-associated protein
12	12	57.1	37.106	60.451	O43852;O43852-3;O43852-5;O43852-6;O43852-2;O43852-4;O43852-10;O43852-9;O43852-13;O43852-14;O43852-15;O43852-11	Calumenin
24	24	36.1	100.23	60.419	Q13435	Splicing factor 3B subunit 2
8	3	22	59.251	60.415	P33240-2;P33240	Cleavage stimulation factor subunit 2
12	12	23.2	75.718	60.28	Q9H6T3;Q9H6T3-2;Q9H6T3-3	RNA polymerase II-associated protein 3
6	6	27.4	33.526	60.279	Q99614	Tetratricopeptide repeat protein 1
11	9	27.5	57.206	60.07	P30837	Aldehyde dehydrogenase X, mitochondrial
12	12	36	52.623	60.033	Q96CS3	FAS-associated factor 2
11	1	51.5	35.594	59.896	P67775;P67775-2	Serine/threonine-protein phosphatase 2A catalytic subunit alpha isoform
10	10	43.2	35.079	59.786	P13804;P13804-2	Electron transfer flavoprotein subunit alpha, mitochondrial
5	5	19.8	40.529	59.742	Q96EP5-2;Q96EP5	DAZ-associated protein 1
22	22	17.1	206.01	59.722	Q92538-3;Q92538-2;Q92538	Golgi-specific brefeldin A-resistance guanine nucleotide exchange factor 1
23	23	32.8	100.2	59.668	Q13200;Q13200-3;Q13200-2	26S proteasome non-ATPase regulatory subunit 2
18	18	22.1	126.73	59.653	Q14203-5;Q14203-2;Q14203-3;Q14203-4;Q14203-6;Q14203	Dynactin subunit 1
5	5	8.4	92.547	59.644	Q8WTT2	Nucleolar complex protein 3 homolog
23	23	40.4	68.569	59.561	P04843	Dolichyl-diphosphooligosaccharide--protein glycosyltransferase subunit 1
12	12	38	38.226	59.536	P00387-3;P00387-2;P00387	NADH-cytochrome b5 reductase 3;NADH-cytochrome b5 reductase 3 membrane-bound form;NADH-cytochrome b5 reductase 3 soluble form
32	30	27.4	177.69	59.529	Q14160-3;Q14160;Q14160-2	Protein scribble homolog
13	13	38.2	54.392	59.319	Q96HE7	ERO1-like protein alpha
17	17	51.6	40.548	59.299	Q99873-3;Q99873-2;Q99873;Q99873-5	Protein arginine N-methyltransferase 1
11	11	32.3	50.212	59.255	P07954-2;P07954	Fumarate hydratase, mitochondrial
21	21	23.9	146.67	59.029	O75153	Clustered mitochondria protein homolog
8	8	70.9	16.961	58.756	P60660-2;P60660	Myosin light polypeptide 6
2	2	9.1	38.926	58.666	P61962	DDB1- and CUL4-associated factor 7
6	6	10.9	82.682	58.62	Q96TA1-2;Q96TA1	Niban-like protein 1
2	2	31.9	13.196	58.558	Q96HQ2;Q96HQ2-2	CDKN2AIP N-terminal-like protein
20	20	33.2	85.64	58.501	P36776-3;P36776-2;P36776	Lon protease homolog, mitochondrial
15	15	19.5	117.97	58.492	A0AVT1;A0AVT1-2	Ubiquitin-like modifier-activating enzyme 6
18	17	41.3	60.13	58.388	O75131	Copine-3
24	5	38.1	68.563	58.209	P35241;P35241-5;P35241-4	Radixin
10	2	35.1	28.521	58.139	P67936	Tropomyosin alpha-4 chain

7	7	40.6	28.068	58.12	Q9Y224	UPF0568 protein C14orf166
14	14	24.4	84.427	58.013	Q14684;Q14684-2	Ribosomal RNA processing protein 1 homolog B
25	19	44.9	60.563	57.996	P17844-2;P17844	Probable ATP-dependent RNA helicase DDX5
17	12	52	29.032	57.988	P06753-2;sp|Tpm3.1rat-6|;sp|Tpm3.1rat-2|;sp|Tpm3.1rat-4|;sp|Tpm3.1rat-3|;sp|Tpm3.1rat-5|;sp|Tpm3.1rat|;sp|Tpm3.1rat-7|;sp|Tpm3.1rat-1|;P06753-5;P06753-3;P06753-6;P06753-4	Tropomyosin alpha-3 chain
10	10	27.1	54.024	57.937	Q9UHX1-4;Q9UHX1-6;Q9UHX1-3;Q9UHX1-5;Q9UHX1-2;Q9UHX1	Poly(U)-binding-splicing factor PUF60
9	9	64.3	16.832	57.936	P63241;P63241-2;Q6IS14	Eukaryotic translation initiation factor 5A-1;Eukaryotic translation initiation factor 5A-1-like
14	14	36.5	64.369	57.8	P08243;P08243-2;P08243-3	Asparagine synthetase [glutamine-hydrolyzing]
6	6	42.1	22.456	57.793	Q99653	Calcineurin B homologous protein 1
11	11	42.4	40.572	57.736	Q9UNZ2;Q9UNZ2-5;Q9UNZ2-4;Q9UNZ2-6	NSFL1 cofactor p47
3	3	30.5	14.787	57.696	P35268	60S ribosomal protein L22
26	26	37.5	87.088	57.655	P54886-2;P54886	Delta-1-pyrroline-5-carboxylate synthase;Glutamate 5-kinase;Gamma-glutamyl phosphate reductase
7	6	13	77.436	57.489	Q9BQL6;Q9BQL6-4;Q9BQL6-2	Fermitin family homolog 1
20	20	26.6	115.73	57.347	Q9H0A0;Q9H0A0-2	N-acetyltransferase 10
9	9	28.2	54.972	57.339	O76021	Ribosomal L1 domain-containing protein 1
11	11	48.9	24.347	57.218	Q9UL25	Ras-related protein Rab-21
4	4	14.9	38.281	57.06	Q14376;Q14376-2	UDP-glucose 4-epimerase
11	11	41.2	50.496	56.76	P00390-4;P00390-2;P00390;P00390-5;P00390-3	Glutathione reductase, mitochondrial
18	18	18.4	149.52	56.752	O15439;O15439-2;O15439-4;O15439-3	Multidrug resistance-associated protein 4
6	6	53.2	16.476	56.72	Q15185-4;Q15185;Q15185-3;Q15185-2	Prostaglandin E synthase 3
9	9	47.3	29.062	56.521	O75822;O75822-3;O75822-2	Eukaryotic translation initiation factor 3 subunit J
10	2	25.8	65.459	56.474	O94925-3	Glutaminase kidney isoform, mitochondrial
8	8	40.1	35.54	56.464	P55735;P55735-2;P55735-4;P55735-3	Protein SEC13 homolog
10	10	45.5	28.772	56.4	Q9Y696	Chloride intracellular channel protein 4
22	22	5.6	632.81	56.355	Q9NU22	Midasin
16	16	15.3	116.61	56.163	P26006;P26006-1	Integrin alpha-3;Integrin alpha-3 heavy chain;Integrin alpha-3 light chain
4	4	26.8	21.943	55.978	O14925;Q5SRD1	Mitochondrial import inner membrane translocase subunit Tim23;Putative mitochondrial import inner membrane translocase subunit Tim23B
9	9	17.6	74.518	55.976	Q92544	Transmembrane 9 superfamily member 4
6	6	33.2	23.182	55.794	sp|Q3SZR3|;CON__Q3SZR3	
12	12	33.3	58.777	55.756	P49591	Serine--tRNA ligase, cytoplasmic
8	6	36.2	47.268	55.659	P09104;P09104-2	Gamma-enolase
23	23	44.2	76.758	55.641	Q06210-2;Q06210	Glutamine--fructose-6-phosphate aminotransferase [isomerizing] 1
7	7	10.6	106.05	55.587	Q99700-2;Q99700-5;Q99700-4;Q99700	Ataxin-2
9	6	44.7	23.567	55.495	P11233	Ras-related protein Ral-A
8	8	30.9	46.939	55.436	Q9Y2T2	AP-3 complex subunit mu-1
16	16	31.3	74.605	55.301	O76094;O76094-2	Signal recognition particle subunit SRP72
9	1	58	22.764	55.288	P35613-4;P35613-2;P35613	Basigin
9	7	23.4	61.557	55.235	Q92804-2;Q92804	TATA-binding protein-associated factor 2N
15	11	63	31.121	55.169	P29692;P29692-3;P29692-2;P29692-4	Elongation factor 1-delta
3	3	24.2	24.57	55.136	Q9NZZ3;Q9NZZ3-2	Charged multivesicular body protein 5
1	1	8.6	19.47	55.026	Q15800-2;Q15800	Methylsterol monooxygenase 1
6	3	53	11.309	54.987	Q15836	Vesicle-associated membrane protein 3
9	9	28.3	45.834	54.903	P82933	28S ribosomal protein S9, mitochondrial
6	6	6.2	149.39	54.803	P49790-2;P49790;P49790-3	Nuclear pore complex protein Nup153
14	14	39.6	53.126	54.789	Q12849;Q12849-5	G-rich sequence factor 1

21	19	30.2	90.725	54.764	P11387	DNA topoisomerase 1
13	13	24.4	96.959	54.667	P98194-2;P98194-4;P98194;P98194-3;P98194-5;P98194-9;P98194-6;P98194-7;P98194-8	Calcium-transporting ATPase type 2C member 1
4	4	23.4	25.789	54.641	P09417;P09417-2	Dihydropteridine reductase
21	20	31.3	90.932	54.512	O43143	Pre-mRNA-splicing factor ATP-dependent RNA helicase DHX15
8	8	20.9	65.33	54.373	Q08380	Galectin-3-binding protein
6	6	39.7	27.4	54.307	O15400-2;O15400	Syntaxin-7
9	9	57.8	22.977	54.079	Q9NP72;Q9NP72-2;Q9NP72-3	Ras-related protein Rab-18
6	6	27.2	28.315	54.055	P04632	Calpain small subunit 1
13	11	33	56.033	53.985	P07948-2;P07948	Tyrosine-protein kinase Lyn
15	15	43.3	47.716	53.94	P23526;P23526-2	Adenosylhomocysteinase
13	13	21.6	82.431	53.828	Q92499;Q92499-3;Q92499-2	ATP-dependent RNA helicase DDX1
3	3	19	16.402	53.69	P17568	NADH dehydrogenase [ubiquinone] 1 beta subcomplex subunit 7
3	1	18.4	20.12	53.466	P51809-3;P51809	Vesicle-associated membrane protein 7
17	17	51.9	47.463	53.349	O00231;O00231-2	26S proteasome non-ATPase regulatory subunit 11
24	24	19.2	204.29	53.105	Q5VYK3	Proteasome-associated protein ECM29 homolog
7	7	27.2	53.12	53.017	P26368-2;P26368	Splicing factor U2AF 65 kDa subunit
7	7	44.8	22.127	52.954	P62081	40S ribosomal protein S7
14	14	23	81.074	52.935	Q08945	FACT complex subunit SSRP1
23	23	38.8	81.889	52.887	P07384	Calpain-1 catalytic subunit
24	22	27.4	134.28	52.87	P00533;P00533-4;P00533-3	Epidermal growth factor receptor
16	16	34	47.697	52.75	P36578	60S ribosomal protein L4
8	8	14.7	85.852	52.75	O43719	HIV Tat-specific factor 1
9	9	52.9	26.923	52.693	Q99714;Q99714-2	3-hydroxyacyl-CoA dehydrogenase type-2
12	11	50	21.892	52.647	P32119	Peroxiredoxin-2
10	10	38.1	45.143	52.59	P68400;Q8NEV1;P68400-2	Casein kinase II subunit alpha;Casein kinase II subunit alpha 3
9	9	27.5	51.466	52.563	O95232	Luc7-like protein 3
10	10	26.8	51.556	52.55	P08621;P08621-2;P08621-3	U1 small nuclear ribonucleoprotein 70 kDa
15	15	67.1	26.688	52.523	P23396;P23396-2	40S ribosomal protein S3
5	4	22.9	25.943	52.402	Q7Z7H5;Q7Z7H5-2;Q7Z7H5-3	Transmembrane emp24 domain-containing protein 4
15	15	50.7	41.024	52.375	Q9Y2Z0;Q9Y2Z0-2	Suppressor of G2 allele of SKP1 homolog
6	6	22.7	41.193	52.365	Q15050	Ribosome biogenesis regulatory protein homolog
14	13	42.2	36.638	52.25	P07195	L-lactate dehydrogenase B chain
9	9	31.6	42.315	52.209	Q9Y570;Q9Y570-4;Q9Y570-2	Protein phosphatase methylesterase 1
8	8	30.3	41.564	52.207	Q15435;Q15435-2;Q15435-3	Protein phosphatase 1 regulatory subunit 7
7	7	41.2	22.876	52.197	P46782	40S ribosomal protein S5;40S ribosomal protein S5, N-terminally processed
8	8	37.5	26.697	52.193	Q96C19	EF-hand domain-containing protein D2
12	12	30.3	49.867	52.192	P49821-2;P49821	NADH dehydrogenase [ubiquinone] flavoprotein 1, mitochondrial
13	13	74.4	23.897	52.101	P61106	Ras-related protein Rab-14
21	21	27.1	103.08	52.05	Q9H3U1;Q9H3U1-2	Protein unc-45 homolog A
3	3	26.7	16.762	51.937	P20674	Cytochrome c oxidase subunit 5A, mitochondrial
10	10	48.3	29.702	51.862	Q9Y5M8	Signal recognition particle receptor subunit beta
10	10	41.6	39.681	51.827	P35249;P35249-2	Replication factor C subunit 4
4	1	50.6	10.366	51.707	P63167	Dynein light chain 1, cytoplasmic
4	4	37.3	12.895	51.422	P58546	Myotrophin
14	14	32.7	67.314	51.349	P61221	ATP-binding cassette sub-family E member 1
3	3	9.4	40.235	51.346	Q9H6F5	Coiled-coil domain-containing protein 86
8	8	33.5	37.578	51.18	Q92905	COP9 signalosome complex subunit 5
13	13	47.5	36.091	51.046	Q15717;Q15717-2	ELAV-like protein 1
10	10	27	58.282	51.02	Q9NP81;Q9NP81-2	Serine--tRNA ligase, mitochondrial
11	11	21.6	75.388	50.794	Q86SF2	N-acetylgalactosaminyltransferase 7

6	6	17.1	66.137	50.739	Q15833-2;Q15833;Q15833-3	Syntaxin-binding protein 2
6	6	8.2	91.746	50.715	Q9Y4R8	Telomere length regulation protein TEL2 homolog
7	7	31.3	38.252	50.7	sp|P17690|;CON__P17690	
11	6	33.3	40.532	50.679	P08754	Guanine nucleotide-binding protein G(k) subunit alpha
17	17	28.9	99.326	50.595	Q14764	Major vault protein
14	14	38.3	52.385	50.548	P54578-2;P54578;P54578-3	Ubiquitin carboxyl-terminal hydrolase 14
17	17	39	60.977	50.491	O43242;O43242-2	26S proteasome non-ATPase regulatory subunit 3
6	6	54.5	17.818	50.416	P30050;P30050-2	60S ribosomal protein L12
9	9	25	57.563	50.29	Q9Y285;Q9Y285-2	Phenylalanine--tRNA ligase alpha subunit
11	11	26.9	58.848	50.072	Q12874	Splicing factor 3A subunit 3
13	13	22.9	84.793	50.071	Q9BUJ2-4;Q9BUJ2-2;Q9BUJ2;Q9BUJ2-3	Heterogeneous nuclear ribonucleoprotein U-like protein 1
10	10	43.4	37.92	50.052	Q9H2U2;Q9H2U2-2;Q9H2U2-6;Q9H2U2-3	Inorganic pyrophosphatase 2, mitochondrial
4	4	33.8	15.512	49.974	Q9Y6H1;Q5T1J5	Coiled-coil-helix-coiled-coil-helix domain-containing protein 2;Putative coiled-coil-helix-coiled-coil-helix domain-containing protein CHCHD2P9, mitochondrial
18	17	41.8	55.804	49.926	P12268	Inosine-5-monophosphate dehydrogenase 2
16	16	19.1	118.71	49.748	Q8TEX9;Q8TEX9-2	Importin-4
15	14	26.2	81.775	49.693	P08237-2;P08237;P08237-3	ATP-dependent 6-phosphofructokinase, muscle type
5	5	20.1	40.834	49.652	Q6UN15-4;Q6UN15-3;Q6UN15-5;Q6UN15	Pre-mRNA 3-end-processing factor FIP1
19	19	36.6	82.285	49.638	P33992	DNA replication licensing factor MCM5
12	12	38.9	40.422	49.608	Q9Y617;Q9Y617-2	Phosphoserine aminotransferase
10	10	48.8	34.222	49.395	Q9BYG3	MKI67 FHA domain-interacting nucleolar phosphoprotein
5	5	12.2	57.274	49.383	Q9NY93-2;Q9NY93	Probable ATP-dependent RNA helicase DDX56
21	2	23.6	134.32	49.259	Q15155	Nodal modulator 1
25	25	28.7	121.68	49.181	O00159;O00159-3;O00159-2	Unconventional myosin-Ic
9	9	11.8	114.33	49.177	Q9BPX3	Condensin complex subunit 3
5	5	41.1	14.582	49.167	O15212	Prefoldin subunit 6
7	7	9.2	117.01	49.126	Q96KR1	Zinc finger RNA-binding protein
9	6	32.9	41.489	49.116	Q99961;Q99961-2;Q99961-3	Endophilin-A2
7	7	44.3	29.176	49.1	O43752	Syntaxin-6
3	3	8.7	56.914	49.088	Q6NXR4	TELO2-interacting protein 2
12	11	41.6	36.688	49.067	P00338;P00338-3;P00338-4;P00338-5;P00338-2	L-lactate dehydrogenase A chain
16	16	19	136.31	48.968	Q9HAV4	Exportin-5
6	5	26.6	28.228	48.956	O43291;O43291-2	Kunitz-type protease inhibitor 2
1	1	4	48.6	48.906	Q14849-2;Q14849-3;Q14849	StAR-related lipid transfer protein 3
7	3	20	46.203	48.686	sp|Q9TTE1|;CON__Q9TTE1	
15	15	48.3	38.242	48.658	Q9Y266	Nuclear migration protein nudC
20	20	22.2	129.68	48.622	P55011-3;P55011	Solute carrier family 12 member 2
6	6	42.9	22.704	48.6	Q9BRP8-2;Q9BRP8	Partner of Y14 and mago
12	11	35.7	61.64	48.564	Q96I24	Far upstream element-binding protein 3
6	4	17.2	39.171	48.519	O75477	Erlin-1
22	22	26.1	113.79	48.488	Q9NSE4	Isoleucine--tRNA ligase, mitochondrial
7	7	9.1	115.28	48.487	O14974;O14974-5;O14974-4;O14974-3;O14974-2	Protein phosphatase 1 regulatory subunit 12A
17	16	33.8	69.72	48.477	P51114;P51114-2;P51114-3	Fragile X mental retardation syndrome-related protein 1
10	7	29.7	57.937	48.295	Q9UEW8-2;Q9UEW8	STE20/SPS1-related proline-alanine-rich protein kinase
24	24	13	289.38	48.141	Q9ULT8	E3 ubiquitin-protein ligase HECTD1
13	13	25.5	72.748	48.066	Q9BTE3-2;Q9BTE3;Q9BTE3-3	Mini-chromosome maintenance complex-binding protein
16	16	53.2	38.388	48.019	P20042	Eukaryotic translation initiation factor 2 subunit 2
20	20	13.4	213.57	47.987	Q12888;Q12888-3;Q12888-2	Tumor suppressor p53-binding protein 1
8	8	17.6	61.448	47.934	P36871;P36871-2;P36871-3	Phosphoglucomutase-1
14	11	30.7	58.042	47.915	Q13177	Serine/threonine-protein kinase PAK 2;PAK-2p27;PAK-2p34

6	6	31.5	23.435	47.823	Q96AJ9-1;Q96AJ9	Vesicle transport through interaction with t-SNAREs homolog 1A
14	14	25.6	90.254	47.79	O43290	U4/U6.U5 tri-snRNP-associated protein 1
12	12	42.6	29.597	47.664	P62701	40S ribosomal protein S4, X isoform
12	12	26.8	80.264	47.625	P20810-9;P20810-10;P20810-7;P20810-6;P20810-5;P20810-4;P20810-8;P20810;P20810-3;P20810-2	Calpastatin
13	13	28.1	60.54	47.57	Q9BVP2-2;Q9BVP2	Guanine nucleotide-binding protein-like 3
10	10	32.4	46.659	47.463	O75874	Isocitrate dehydrogenase [NADP] cytoplasmic
4	4	24.5	22.958	47.425	O15258	Protein RER1
8	8	31.3	36.104	47.412	P40937-2;P40937	Replication factor C subunit 5
19	8	48.7	32.852	47.385	P05141	ADP/ATP translocase 2;ADP/ATP translocase 2, N-terminally processed
18	18	47.9	52.22	46.877	P60228	Eukaryotic translation initiation factor 3 subunit E
6	6	14.2	79.611	46.841	Q9UH99-3;Q9UH99;Q9UH99-2	SUN domain-containing protein 2
12	12	50.6	36.032	46.796	P30519;P30519-2	Heme oxygenase 2
9	9	23.1	61.212	46.763	Q05682-5;Q05682-4;Q05682-3;Q05682-6;Q05682-2;Q05682	Caldesmon
11	5	36.4	48.534	46.616	P04181	Ornithine aminotransferase, mitochondrial;Ornithine aminotransferase, hepatic form;Ornithine aminotransferase, renal form
9	3	64.2	18.012	46.555	P62937	Peptidyl-prolyl cis-trans isomerase A;Peptidyl-prolyl cis-trans isomerase A, N-terminally processed
5	5	54.3	11.749	46.48	P99999	Cytochrome c
19	18	41.1	70.81	46.439	P13797;P13797-2;P13797-3	Plastin-3
15	15	17.5	133.9	46.355	P30876	DNA-directed RNA polymerase II subunit RPB2
15	15	52.5	30.276	46.2	sp|P15497|;CON__P15497	
11	11	47.3	38.274	46.081	O95433;O95433-2	Activator of 90 kDa heat shock protein ATPase homolog 1
12	12	15.6	115.96	46.015	Q96P70	Importin-9
10	10	15	110.06	45.951	Q02241;Q02241-2;Q02241-3	Kinesin-like protein KIF23
8	8	21.1	62.442	45.87	O95671-3;O95671-2;O95671	N-acetylserotonin O-methyltransferase-like protein
23	23	21.5	152.29	45.796	Q96RT1-5;Q96RT1-3;Q96RT1-9;Q96RT1-2;Q96RT1;Q96RT1-8;Q96RT1-7;Q96RT1-6;Q96RT1-4	Protein LAP2
10	5	59.7	20.697	45.671	P84077;P61204;P61204-2	ADP-ribosylation factor 1;ADP-ribosylation factor 3
9	9	32.1	44.739	45.603	Q13148;Q13148-4	TAR DNA-binding protein 43
20	20	24.5	117.36	45.506	Q7L014	Probable ATP-dependent RNA helicase DDX46
8	6	34.7	37.23	45.41	Q9UBU8-2;Q9UBU8-3;Q9UBU8	Mortality factor 4-like protein 1
5	5	62.2	13.331	45.298	O76070	Gamma-synuclein
12	12	36	53.488	45.271	Q9UBB4;Q9UBB4-2	Ataxin-10
17	17	18.3	128.79	45.268	Q9Y2A7;Q9Y2A7-2	Nck-associated protein 1
3	3	23.4	16.648	45.042	Q9UHV9	Prefoldin subunit 2
12	12	30	55.023	44.986	O60701;O60701-2;O60701-3	UDP-glucose 6-dehydrogenase
10	10	41.2	34.482	44.924	O14579;O14579-2;O14579-3	Coatomer subunit epsilon
11	11	31.2	55.704	44.794	P61011;P61011-2	Signal recognition particle 54 kDa protein
15	15	35.7	54.933	44.769	O95793-2;O95793;O95793-3	Double-stranded RNA-binding protein Staufen homolog 1
3	3	4.8	105.47	44.748	Q9HCG8	Pre-mRNA-splicing factor CWC22 homolog
21	21	27.2	111.63	44.726	O60313;O60313-2;O60313-13;O60313-9;O60313-11;O60313-10	Dynamin-like 120 kDa protein, mitochondrial;Dynamin-like 120 kDa protein, form S1
4	4	16.6	38.963	44.666	Q8NBM4;Q8NBM4-4;Q8NBM4-3;Q8NBM4-2	Ubiquitin-associated domain-containing protein 2
7	7	30.5	36.521	44.652	O00273;O00273-2	DNA fragmentation factor subunit alpha
15	13	54.5	34.095	44.651	P06493;P06493-2	Cyclin-dependent kinase 1
20	20	26.3	109.93	44.614	Q15020;Q15020-4	Squamous cell carcinoma antigen recognized by T-cells 3
24	24	29.6	128.3	44.561	Q93009;Q93009-3	Ubiquitin carboxyl-terminal hydrolase 7
7	7	22.6	34.47	44.508	Q9H936	Mitochondrial glutamate carrier 1

11	11	18	129.93	44.437	Q9NQC3;Q9NQC3-2;Q9NQC3-5;Q9NQC3-4;Q9NQC3-6	Reticulon-4
21	21	14.6	227.92	44.433	Q92621	Nuclear pore complex protein Nup205
14	14	35.5	59.578	44.377	Q9Y2X3	Nucleolar protein 58
10	10	35.9	46.637	44.368	P43034	Platelet-activating factor acetylhydrolase IB subunit alpha
12	12	29.8	60.541	44.367	Q6DD88	Atlastin-3
12	2	38.1	52.891	44.276	P50579;P50579-3	Methionine aminopeptidase 2
5	5	40.8	19.863	44.241	P55327-2;P55327-4;P55327;P55327-7;P55327-5;P55327-6;P55327-3	Tumor protein D52
10	10	18.2	99.996	44.106	Q9Y2U8	Inner nuclear membrane protein Man1
12	12	33.2	51.311	43.973	Q16401-2;Q16401	26S proteasome non-ATPase regulatory subunit 5
16	3	42.7	50.432	43.96	Q13509;Q13509-2	Tubulin beta-3 chain
15	15	43.9	49.966	43.956	P18031	Tyrosine-protein phosphatase non-receptor type 1
13	13	23.7	87.563	43.932	Q9BQ52-4;Q9BQ52;Q9BQ52-3	Zinc phosphodiesterase ELAC protein 2
6	6	32.4	23.495	43.911	P57735	Ras-related protein Rab-25
5	2	58.4	10.35	43.837	Q96FJ2	Dynein light chain 2, cytoplasmic
10	10	35.9	39.157	43.821	P35250;P35250-2	Replication factor C subunit 2
9	9	37.5	30.658	43.724	Q9Y277;Q9Y277-2	Voltage-dependent anion-selective channel protein 3
15	15	34.1	70.973	43.586	Q14258	E3 ubiquitin/ISG15 ligase TRIM25
2	2	18.8	29.34	43.564	Q8IUW5	RELT-like protein 1
8	8	33.5	29.506	43.494	P61289;P61289-3;P61289-2	Proteasome activator complex subunit 3
8	8	22.1	58.24	43.475	P35269	General transcription factor IIF subunit 1
8	8	39.5	26.183	43.406	Q15631	Translin
25	25	23.2	177.19	43.396	Q9NYU2;Q9NYU2-2	UDP-glucose:glycoprotein glucosyltransferase 1
16	16	24	106.19	43.388	CON__Q9TRI1;sp|Q9TRI1|	
5	5	22	23.662	43.263	P09496-2;P09496-4;P09496-3;P09496	Clathrin light chain A
9	7	41	22.541	43.148	P61026	Ras-related protein Rab-10
6	6	23.6	37.821	43.122	P07858	Cathepsin B;Cathepsin B light chain;Cathepsin B heavy chain
18	18	23.7	106.92	43.119	O94906;O94906-2	Pre-mRNA-processing factor 6
15	15	41	50.118	43.011	P26641;P26641-2	Elongation factor 1-gamma
11	11	36.2	42.621	42.959	P35237	Serpin B6
8	4	25.8	49.263	42.891	P55795	Heterogeneous nuclear ribonucleoprotein H2
8	8	39.8	32.193	42.861	Q14192	Four and a half LIM domains protein 2
14	13	18.3	108.66	42.684	Q9Y2W1	Thyroid hormone receptor-associated protein 3
15	15	29.5	57.21	42.683	P48444;P48444-2	Coatomer subunit delta
6	6	26.5	23.466	42.6	P84098	60S ribosomal protein L19
10	5	28.7	62.518	42.466	Q9UMX0;Q9UMX0-2;Q9UMX0-3	Ubiquilin-1
13	13	41	47.628	42.442	Q8NBS9;Q8NBS9-2	Thioredoxin domain-containing protein 5
22	22	31.4	106.1	42.355	Q9UNX4	WD repeat-containing protein 3
21	21	27.3	92.931	42.296	Q9H5V8;Q9H5V8-2	CUB domain-containing protein 1
5	5	43.6	14.865	42.28	P62829	60S ribosomal protein L23
13	13	47.1	32.854	42.271	P08865	40S ribosomal protein SA
17	17	35.1	80.699	42.235	P48147	Prolyl endopeptidase
8	7	15.8	69.491	42.047	Q96PK6	RNA-binding protein 14
5	5	20.4	36.844	41.959	P82673	28S ribosomal protein S35, mitochondrial
13	13	15.6	128.18	41.897	P35251-2;P35251	Replication factor C subunit 1
27	11	33.3	111.75	41.775	P13637;P13637-2;P13637-3	Sodium/potassium-transporting ATPase subunit alpha-3
14	14	30.2	83.629	41.727	Q14137;Q14137-2	Ribosome biogenesis protein BOP1
12	12	22.7	93.52	41.666	Q86Y56;Q86Y56-2	Dynein assembly factor 5, axonemal
11	11	17.9	87.996	41.65	Q9UGP8	Translocation protein SEC63 homolog
11	11	61.6	23.354	41.625	O00161;O00161-2	Synaptosomal-associated protein 23

5	5	59.3	16.694	41.431	O43169	Cytochrome b5 type B
11	11	31.6	53.553	41.409	sp|Q2KJF1|;CON__Q2KJF1	
4	4	8.6	90.207	41.373	Q9UGT4	Sushi domain-containing protein 2
5	5	33.2	26.667	41.369	P21583-3;P21583-2;P21583	Kit ligand;Soluble KIT ligand
9	8	32.4	38.044	41.358	P25685;P25685-2	DnaJ homolog subfamily B member 1
10	10	42.6	32.251	41.328	O43396	Thioredoxin-like protein 1
8	8	44.6	31.554	41.238	P29966	Myristoylated alanine-rich C-kinase substrate
6	6	24.5	43.02	41.201	Q92734-2;Q92734;Q92734-4;Q92734-3	Protein TFG
2	2	14.3	27.835	41.131	Q8WY22	BRI3-binding protein
7	6	30	37.344	41.097	P55209-3;P55209-2;P55209	Nucleosome assembly protein 1-like 1
15	11	56.8	22.11	41.021	Q06830	Peroxiredoxin-1
14	14	49.6	44.172	40.936	P62333	26S protease regulatory subunit 10B
10	10	34.7	48.486	40.853	Q9UKS6	Protein kinase C and casein kinase substrate in neurons protein 3
8	8	24.9	41.806	40.807	Q9BRK5;Q9BRK5-6;Q9BRK5-4;Q9BRK5-3;Q9BRK5-2	45 kDa calcium-binding protein
10	10	40.4	44.969	40.688	P18754;P18754-2	Regulator of chromosome condensation
5	5	16.9	60.593	40.659	Q9H3P7	Golgi resident protein GCP60
3	3	30.8	13.734	40.345	Q9NWU5-2;Q9NWU5-3;Q9NWU5	39S ribosomal protein L22, mitochondrial
13	13	47.3	42.502	40.227	Q7L2H7;Q7L2H7-2	Eukaryotic translation initiation factor 3 subunit M
11	11	39.2	46.971	40.174	Q9BS26	Endoplasmic reticulum resident protein 44
18	18	31.4	83.165	40.033	P41250	Glycine--tRNA ligase
8	8	5.8	201.05	40.015	Q69YN4-3;Q69YN4;Q69YN4-2;Q69YN4-4	Protein virilizer homolog
10	10	60	26.599	39.983	P56537;P56537-2	Eukaryotic translation initiation factor 6
15	11	13.3	174.38	39.88	P11388;P11388-2;P11388-3;P11388-4	DNA topoisomerase 2-alpha
12	12	24.2	58.112	39.862	P07602;P07602-2;P07602-3	Prosaposin;Saposin-A;Saposin-B-Val;Saposin-B;Saposin-C;Saposin-D
16	13	38.2	46.871	39.544	P38919	Eukaryotic initiation factor 4A-III;Eukaryotic initiation factor 4A-III, N-terminally processed
4	4	20.4	33.412	39.487	P53384-2;P53384	Cytosolic Fe-S cluster assembly factor NUBP1
14	14	16.5	138.8	39.345	Q01970;Q01970-2	1-phosphatidylinositol 4,5-bisphosphate phosphodiesterase beta-3
23	23	13.8	265.55	39.173	Q13085;Q13085-3;Q13085-2;Q13085-4	Acetyl-CoA carboxylase 1;Biotin carboxylase
12	12	9.2	181.26	39.167	P51532-5;P51532-2;P51532-3;P51532-4;P51532;P51531-2;P51531	Transcription activator BRG1;Probable global transcription activator SNF2L2
11	11	43.1	36.573	39.073	P14550	Alcohol dehydrogenase [NADP(+)]
19	19	26.3	98.555	39.048	Q9P2B2	Prostaglandin F2 receptor negative regulator
9	9	22.7	56.805	38.997	Q16850;Q16850-2	Lanosterol 14-alpha demethylase
7	7	34.6	26.659	38.939	Q9ULC3	Ras-related protein Rab-23
4	4	31.2	21.474	38.908	O60232	Sjoegren syndrome/scleroderma autoantigen 1
7	6	40.6	32.949	38.887	P47755;P47755-2	F-actin-capping protein subunit alpha-2
19	19	31.6	66.115	38.886	Q9NSD9;Q9NSD9-2	Phenylalanine--tRNA ligase beta subunit
15	15	51.1	44.868	38.862	P31689;P31689-2	DnaJ homolog subfamily A member 1
8	8	74.5	10.932	38.852	P61604	10 kDa heat shock protein, mitochondrial
14	14	15.1	143.7	38.749	Q5JTH9;Q5JTH9-2;Q5JTH9-3	RRP12-like protein
13	13	19.3	84.141	38.637	O14672;O14672-2	Disintegrin and metalloproteinase domain-containing protein 10
10	10	28.8	50.646	38.544	Q9H0S4;Q9H0S4-2	Probable ATP-dependent RNA helicase DDX47
4	4	11.7	56.252	38.543	Q9BVG9	Phosphatidylserine synthase 2
13	13	44.2	46.91	38.522	Q9UGI8-2;Q9UGI8	Testin
14	14	37.4	51.355	38.406	O43615	Mitochondrial import inner membrane translocase subunit TIM44
8	8	31.4	31.282	38.303	Q9UBX3;Q9UBX3-2	Mitochondrial dicarboxylate carrier
5	5	6.7	141.32	38.067	Q8N3U4;Q8N3U4-2	Cohesin subunit SA-2
14	14	23.6	96.331	38.058	Q9GZR7;Q9GZR7-2	ATP-dependent RNA helicase DDX24
9	5	43.5	23.668	38.019	P61006;P61006-2	Ras-related protein Rab-8A
9	9	38	36.954	38.005	O43684-2;O43684	Mitotic checkpoint protein BUB3

10	9	20.9	62.333	37.998	Q9Y5A9;Q9Y5A9-2	YTH domain-containing family protein 2
6	6	18.7	38.002	37.927	P10644-2;P10644	cAMP-dependent protein kinase type I-alpha regulatory subunit;cAMP-dependent protein kinase type I-alpha regulatory subunit, N-terminally processed
3	3	16.7	31.642	37.911	Q86Y82	Syntaxin-12
11	11	20.1	101.43	37.824	Q7Z2W4;Q7Z2W4-2;Q7Z2W4-3	Zinc finger CCCH-type antiviral protein 1
3	1	65.6	6.0422	37.822	P02795;P13640-2;P13640	Metallothionein-2;Metallothionein-1G
10	9	37.3	42.823	37.718	Q99733;Q99733-2	Nucleosome assembly protein 1-like 4
14	11	30.3	60.626	37.673	Q9H4M9	EH domain-containing protein 1
17	6	49.3	50.67	37.636	P42167	Lamina-associated polypeptide 2, isoforms beta/gamma;Thymopoietin;Thymopentin
10	10	17.4	83.247	37.602	Q9UBC2-3;Q9UBC2;Q9UBC2-2;Q9UBC2-4	Epidermal growth factor receptor substrate 15-like 1
16	16	19.4	117.51	37.575	Q5JRX3-2;Q5JRX3;Q5JRX3-3	Presequence protease, mitochondrial
8	8	15.9	74.535	37.341	Q9NYY8-2;Q9NYY8	FAST kinase domain-containing protein 2
7	2	24.6	32.989	37.336	P07951-2;P07951-3;sp|Q3SX28|;CON__Q3SX28	
27	27	34.3	115.93	37.327	Q02218;Q02218-2	2-oxoglutarate dehydrogenase, mitochondrial
9	5	35.4	39.455	37.26	P09972	Fructose-bisphosphate aldolase C
6	0	46	13.92	37.255	Q16778;P33778;P23527;P06899	Histone H2B type 2-E;Histone H2B type 1-B;Histone H2B type 1-O;Histone H2B type 1-J
8	8	16.7	65.601	37.236	Q13123	Protein Red
12	12	31.1	50.8	37.175	P39656;P39656-3;P39656-2	Dolichyl-diphosphooligosaccharide--protein glycosyltransferase 48 kDa subunit
10	10	11.6	132.95	37.151	Q9HD20;Q9HD20-2	Manganese-transporting ATPase 13A1
19	4	54.3	49.67	37.064	P07437	Tubulin beta chain
17	17	16.8	157.18	37.055	Q15021	Condensin complex subunit 1
15	15	40.1	56.44	37.042	Q99615;Q99615-2	DnaJ homolog subfamily C member 7
13	13	18.4	85.237	37.009	P49321;P49321-2;P49321-3;P49321-4	Nuclear autoantigenic sperm protein
16	1	24.2	101.19	36.845	Q9BZF1;Q9BZF1-3	Oxysterol-binding protein-related protein 8
14	14	11.6	174.21	36.779	Q92887	Canalicular multispecific organic anion transporter 1
13	13	31	61.68	36.77	P35052	Glypican-1;Secreted glypican-1
14	8	43.3	47.82	36.757	Q16576;Q16576-2	Histone-binding protein RBBP7
19	19	28.8	101.34	36.752	Q6ZNB6;Q6ZNB6-2	NF-X1-type zinc finger protein NFXL1
5	5	20.5	41.881	36.742	Q7LGA3;Q7LGA3-3;Q7LGA3-2	Heparan sulfate 2-O-sulfotransferase 1
5	5	70.4	11.139	36.618	P04080	Cystatin-B
8	8	24.2	51.844	36.615	Q5TFE4;Q5TFE4-2	5-nucleotidase domain-containing protein 1
19	19	17.3	186.96	36.582	Q07157-2;Q07157	Tight junction protein ZO-1
3	3	27.4	15.86	36.485	P62910	60S ribosomal protein L32
9	9	18.9	70.182	36.471	Q9UBU9;Q9UBU9-2	Nuclear RNA export factor 1
12	11	30.9	51.812	36.455	Q13596-2;Q13596;Q13596-3	Sorting nexin-1
6	6	14.8	63.157	36.44	Q9BTX1-6;Q9BTX1-5;Q9BTX1-2;Q9BTX1;Q9BTX1-4	Nucleoporin NDC1
9	8	33.1	40.949	36.439	O15143	Actin-related protein 2/3 complex subunit 1B
14	9	18.2	103.98	36.387	Q13620;Q13620-1;Q13620-3	Cullin-4B
6	6	13	66.574	36.387	O95453-2;O95453;O95453-3;O95453-4	Poly(A)-specific ribonuclease PARN
7	7	28.1	40.763	36.379	Q08752	Peptidyl-prolyl cis-trans isomerase D
20	1	8.9	319.1	36.339	P50851	Lipopolysaccharide-responsive and beige-like anchor protein
12	12	21.5	78.839	36.25	P52888	Thimet oligopeptidase
14	14	42.1	56.084	36.162	Q9P258	Protein RCC2
19	13	24.6	105.36	36.157	O95782-2;O95782	AP-2 complex subunit alpha-1
7	5	19.5	55.238	36.12	Q92925-3;Q92925-2;Q92925	SWI/SNF-related matrix-associated actin-dependent regulator of chromatin subfamily D member 2
3	3	74.6	11.514	36.097	P05386	60S acidic ribosomal protein P1
12	11	30.4	66.248	36.097	Q5JTV8;Q5JTV8-3	Torsin-1A-interacting protein 1
6	6	25.9	35.777	36.055	Q13505-3;Q13505;Q13505-2	Metaxin-1
24	12	33.7	101.35	36.008	Q10567-4;Q10567-3;Q10567-2;Q10567	AP-1 complex subunit beta-1
8	8	24.5	54.548	35.983	P12955;P12955-3;P12955-2	Xaa-Pro dipeptidase

3	3	13	43.158	35.956	Q12824-2;Q12824	SWI/SNF-related matrix-associated actin-dependent regulator of chromatin subfamily B member 1
6	6	12.7	88.754	35.932	Q9UBV2;Q9UBV2-2	Protein sel-1 homolog 1
16	16	16.9	149.01	35.885	O75694-2;O75694	Nuclear pore complex protein Nup155
6	6	6.4	138.99	35.855	Q9H2G2-2;Q9H2G2	STE20-like serine/threonine-protein kinase
13	13	35.2	51.976	35.839	Q9Y512	Sorting and assembly machinery component 50 homolog
17	17	39.1	74.403	35.717	Q14166	Tubulin--tyrosine ligase-like protein 12
14	14	31.2	53.354	35.645	Q6NUK1;Q6NUK1-2	Calcium-binding mitochondrial carrier protein SCaMC-1
10	10	37.5	35.422	35.586	P08574	Cytochrome c1, heme protein, mitochondrial
12	12	25	77.187	35.549	O15228;O15228-2	Dihydroxyacetone phosphate acyltransferase
9	9	40.9	39.31	35.435	Q96DI7;Q96DI7-2	U5 small nuclear ribonucleoprotein 40 kDa protein
10	10	40.7	36.748	35.298	Q9NUQ9;Q9NUQ9-2	Protein FAM49B
14	14	42.8	45.462	35.242	P62495-2;P62495	Eukaryotic peptide chain release factor subunit 1
19	1	16.3	144.31	35.226	Q13428-2;Q13428-8;Q13428;Q13428-3;Q13428-4;Q13428-5	Treacle protein
3	3	14.3	26.671	35.212	Q9UNN8	Endothelial protein C receptor
9	9	30.2	42.663	35.112	sp|Q58D62|;CON__Q58D62	
7	7	40	24.783	35.079	P41227-2;P41227	N-alpha-acetyltransferase 10
15	15	30.2	75.356	35.068	Q8IVT2	Mitotic interactor and substrate of PLK1
10	10	20.3	69.557	35.018	Q86X29-4;Q86X29;Q86X29-6;Q86X29-5;Q86X29-3;Q86X29-2	Lipolysis-stimulated lipoprotein receptor
7	7	22.8	58.889	34.947	Q6L8Q7-2;Q6L8Q7	2,5-phosphodiesterase 12
6	6	14.8	54.71	34.91	CON__P28800	
13	13	23.1	85.95	34.865	Q8TCS8	Polyribonucleotide nucleotidyltransferase 1, mitochondrial
4	4	18.8	39.705	34.859	Q8N6T3-5;Q8N6T3;Q8N6T3-2;Q8N6T3-4;Q8N6T3-3	ADP-ribosylation factor GTPase-activating protein 1
7	6	41.3	24.389	34.842	P20338	Ras-related protein Rab-4A
3	3	15.2	32.461	34.828	P18827	Syndecan-1
19	19	20	141.29	34.811	Q9C0C9	E2/E3 hybrid ubiquitin-protein ligase UBE2O
13	13	25.6	75.475	34.778	P28288;P28288-2	ATP-binding cassette sub-family D member 3
7	7	32.9	24.423	34.726	P62826	GTP-binding nuclear protein Ran
16	14	35.9	61.174	34.702	Q9H223	EH domain-containing protein 4
7	7	36.8	28.048	34.674	P35270	Sepiapterin reductase
11	11	48.7	39.548	34.621	Q6IBS0	Twinfilin-2
1	1	5	31.524	34.619	P82970	High mobility group nucleosome-binding domain-containing protein 5
12	12	42.3	39.93	34.604	O15372	Eukaryotic translation initiation factor 3 subunit H
12	11	44.6	38.169	34.55	O75436;O75436-2	Vacuolar protein sorting-associated protein 26A
8	8	42.9	25.003	34.533	Q15005	Signal peptidase complex subunit 2
7	5	9.1	109.87	34.468	P29323-2;P29323-3;P29323	Ephrin type-B receptor 2
6	6	30.5	27.401	34.433	Q9UL46	Proteasome activator complex subunit 2
8	8	26.7	72.995	34.396	Q9UKV5	E3 ubiquitin-protein ligase AMFR
15	14	41.9	60.419	34.326	Q16881-4;Q16881-3;Q16881;Q16881-5;Q16881-2;Q16881-6;Q16881-7	Thioredoxin reductase 1, cytoplasmic
10	10	19	82.915	34.32	P05067-7;P05067-11;P05067-8;P05067-9;P05067;P05067-10;P05067-3;P05067-4;P05067-5;P05067-6	Amyloid beta A4 protein;N-APP;Soluble APP-alpha;Soluble APP-beta;C99;Beta-amyloid protein 42;Beta-amyloid protein 40;C83;P3(42);P3(40);C80;Gamma-secretase C-terminal fragment 59;Gamma-secretase C-terminal fragment 57;Gamma-secretase C-terminal fragment 50;C31
6	6	42.8	18.091	34.299	Q07020-2;Q07020	60S ribosomal protein L18
9	9	39.3	35.554	34.281	P27695	DNA-(apurinic or apyrimidinic site) lyase;DNA-(apurinic or apyrimidinic site) lyase, mitochondrial
8	8	22.7	43.159	34.267	Q9P035;Q9P035-2	Very-long-chain (3R)-3-hydroxyacyl-CoA dehydratase 3
7	7	41.6	22.626	34.266	P61758;P61758-2	Prefoldin subunit 3
14	14	20.4	104.2	34.256	Q9Y5L0;Q9Y5L0-3;Q9Y5L0-1;Q9Y5L0-5	Transportin-3
16	16	19.7	138.49	34.222	Q13045-2;Q13045;Q13045-3	Protein flightless-1 homolog

9	9	21.4	64.519	34.125	A0MZ66-5;A0MZ66-6;A0MZ66;A0MZ66-3;A0MZ66-8;A0MZ66-4;A0MZ66-2	Shootin-1
12	12	28.6	63.664	34.036	Q13740-2;Q13740	CD166 antigen
9	9	39.8	30.241	34.021	O75489	NADH dehydrogenase [ubiquinone] iron-sulfur protein 3, mitochondrial
7	5	42.2	23.399	33.943	P62070;P62070-4;P62070-3;P62070-2	Ras-related protein R-Ras2
16	16	43.8	46.48	33.91	Q9HDC9;Q9HDC9-2	Adipocyte plasma membrane-associated protein
14	14	21	105.32	33.827	Q8NI36	WD repeat-containing protein 36
21	21	10.7	266.94	33.772	A5YKK6;A5YKK6-2;A5YKK6-3	CCR4-NOT transcription complex subunit 1
9	9	11.4	125.82	33.762	Q9Y2H6-2;Q9Y2H6	Fibronectin type-III domain-containing protein 3A
15	15	11.1	232.3	33.72	P18583-2;P18583-10;P18583-3;P18583-4;P18583-7;P18583;P18583-5;P18583-9;P18583-6;P18583-8	Protein SON
5	5	20.5	40.556	33.645	P40938;P40938-2	Replication factor C subunit 3
9	9	38.9	34.583	33.627	Q9NZL9-4;Q9NZL9-2;Q9NZL9;Q9NZL9-3	Methionine adenosyltransferase 2 subunit beta
13	13	34.6	61.053	33.552	Q9P0J1;Q9P0J1-2	[Pyruvate dehydrogenase [acetyl-transferring]]-phosphatase 1, mitochondrial
6	6	13.4	73.457	33.547	P46063	ATP-dependent DNA helicase Q1
12	12	23.5	69.223	33.51	O76031	ATP-dependent Clp protease ATP-binding subunit clpX-like, mitochondrial
10	6	25.2	60.029	33.464	O60684;O15131	Importin subunit alpha-7;Importin subunit alpha-6
13	13	26.1	66.966	33.462	Q9NTJ5;Q9NTJ5-2	Phosphatidylinositide phosphatase SAC1
6	6	23.3	40.683	33.437	Q9NV96;Q9NV96-2;Q9NV96-3	Cell cycle control protein 50A
3	3	17.8	30.124	33.289	Q96GG9	DCN1-like protein 1
4	4	50.4	14.523	33.201	P36954	DNA-directed RNA polymerase II subunit RPB9
16	16	15.9	152.93	33.2	O75976;O75976-2	Carboxypeptidase D
7	7	31.5	36.876	33.181	Q14257;Q14257-2	Reticulocalbin-2
4	4	30.6	19.3	33.153	P63098	Calcineurin subunit B type 1
14	14	9.1	249.4	33.145	P02751-14;P02751-17;P02751-1;P02751-10;P02751-6;P02751-5;P02751-9;P02751-3;P02751-8;P02751-7;P02751-11;P02751;P02751-13;P02751-12;P02751-4	Fibronectin;Anastellin;Ugl-Y1;Ugl-Y2;Ugl-Y3
5	3	32.3	14.107	33.101	P20671;Q96KK5;Q16777;Q9BTM1;Q93077;Q7L7L0;Q6FI13;Q99878;P04908;P0C0S8;Q96QV6;P16104;Q9BTM1-2	Histone H2A type 1-D;Histone H2A type 1-H;Histone H2A type 2-C;Histone H2A.J;Histone H2A type 1-C;Histone H2A type 3;Histone H2A type 2-A;Histone H2A type 1-J;Histone H2A type 1-B/E;Histone H2A type 1;Histone H2A type 1-A;Histone H2AX
5	5	6.8	110.11	32.989	Q24JP5;Q24JP5-2;Q24JP5-4	Transmembrane protein 132A
9	9	15.4	97.455	32.981	P12830;P12830-2	Cadherin-1;E-Cad/CTF1;E-Cad/CTF2;E-Cad/CTF3
17	17	26	97.651	32.874	P50570-2;P50570-5;P50570;P50570-3;P50570-4	Dynamin-2
16	14	24.2	102.38	32.871	P52789	Hexokinase-2
11	11	16.2	113.67	32.845	Q9HCE1;Q9HCE1-2	Putative helicase MOV-10
6	6	30.3	25.855	32.817	P30085-3;P30085;P30085-2	UMP-CMP kinase
5	5	48.3	19.343	32.786	O75607	Nucleoplasmin-3
13	13	25.5	69.917	32.758	Q9NQW7;Q9NQW7-3;Q9NQW7-2;Q9NQW7-4	Xaa-Pro aminopeptidase 1
18	18	43.6	53.879	32.753	Q02818	Nucleobindin-1
14	14	26.3	83.023	32.744	Q8TBA6;Q8TBA6-2	Golgin subfamily A member 5
14	14	12.7	170.45	32.739	Q9UIG0-2;Q9UIG0	Tyrosine-protein kinase BAZ1B
11	4	66.2	22.171	32.675	Q9H0U4	Ras-related protein Rab-1B
13	13	24.1	87.334	32.649	P42224;P42224-2	Signal transducer and activator of transcription 1-alpha/beta
13	11	22.2	85.018	32.594	P17858;P17858-2	ATP-dependent 6-phosphofructokinase, liver type
11	11	55.3	27.399	32.549	P60900;P60900-2	Proteasome subunit alpha type-6
12	12	24.9	62.094	32.513	P19525;P19525-2	Interferon-induced, double-stranded RNA-activated protein kinase
13	13	10.8	199.07	32.449	Q7KZ85	Transcription elongation factor SPT6
16	16	21.2	107.97	32.363	P78347-2;P78347-4;P78347-3;P78347	General transcription factor II-I
16	16	13.1	192.07	32.344	Q14669-4;Q14669-2;Q14669;Q14669-3	E3 ubiquitin-protein ligase TRIP12

8	8	34.8	24.146	32.297	P61313	60S ribosomal protein L15
12	12	43.5	27.744	32.229	Q07955;Q07955-3;Q07955-2	Serine/arginine-rich splicing factor 1
15	15	17.7	116.19	32.223	O00203-3;O00203	AP-3 complex subunit beta-1
15	11	14.8	145.18	32.184	Q7L576;Q7L576-2	Cytoplasmic FMR1-interacting protein 1
6	5	19.1	58.136	32.183	Q9P0V9-2;Q9P0V9;Q9P0V9-3	Septin-10
9	8	50.9	18.506	32.136	P60981;P60981-2	Destrin
18	18	17.2	138.62	31.972	O00411	DNA-directed RNA polymerase, mitochondrial
7	7	45.5	23.277	31.942	P48047	ATP synthase subunit O, mitochondrial
8	8	17.7	71.223	31.94	Q9UBT2;Q9UBT2-2	SUMO-activating enzyme subunit 2
8	8	29.3	47.146	31.936	Q9NPH2-2;Q9NPH2-3;Q9NPH2	Inositol-3-phosphate synthase 1
7	7	37	22.406	31.873	P61086;P61086-2;P61086-3	Ubiquitin-conjugating enzyme E2 K
11	11	45.6	28.48	31.87	P28074;P28074-3	Proteasome subunit beta type-5
9	9	22.8	59.747	31.83	Q8TAA9-2;Q8TAA9	Vang-like protein 1
10	10	33.3	44.743	31.76	Q9NTK5;Q9NTK5-2;Q9NTK5-3	Obg-like ATPase 1
2	2	7.6	46.411	31.703	Q8ND56-3;Q8ND56-2;Q8ND56	Protein LSM14 homolog A
4	4	27.2	24.843	31.646	Q6P587;Q6P587-2;Q6P587-3	Acylpyruvase FAHD1, mitochondrial
4	4	16.9	46.744	31.608	P49841;P49841-2	Glycogen synthase kinase-3 beta
5	5	33	28.932	31.57	Q9UHY7	Enolase-phosphatase E1
13	13	49.1	32.004	31.529	Q96HS1;Q96HS1-2	Serine/threonine-protein phosphatase PGAM5, mitochondrial
12	12	19.3	91.215	31.528	sp|P06868|;CON__P06868	
16	16	19.5	132.6	31.519	Q9BTW9;Q9BTW9-4;Q9BTW9-5	Tubulin-specific chaperone D
4	4	6	97.667	31.493	P78316;P78316-2	Nucleolar protein 14
11	11	62.1	11.367	31.471	P62805	Histone H4
9	4	53.2	21.416	31.359	Q96BM9	ADP-ribosylation factor-like protein 8A
14	4	41.1	48.991	31.342	Q13838;Q13838-2	Spliceosome RNA helicase DDX39B
10	10	42	43.614	31.314	Q9NX58	Cell growth-regulating nucleolar protein
8	8	20.3	48.043	31.299	Q7L1Q6;Q7L1Q6-4;Q7L1Q6-3;Q7L1Q6-2	Basic leucine zipper and W2 domain-containing protein 1
8	8	15.3	76.889	31.267	Q969X6;Q969X6-2;Q969X6-3	Cirhin
1	1	8.5	23.132	31.267	O76080	AN1-type zinc finger protein 5
6	6	25.5	43.109	31.23	Q6NYC1-2;Q6NYC1;Q6NYC1-3	Bifunctional arginine demethylase and lysyl-hydroxylase JMJD6
4	4	10.9	56.109	31.205	Q96ST2-2;Q96ST2-3;Q96ST2	Protein IWS1 homolog
3	3	32	14.585	31.083	Q9Y3B4	Splicing factor 3B subunit 6
3	2	65.6	6.0142	31.051	P04732;sp|P67983|;CON__P67983	Metallothionein-1E
8	8	47.1	19.891	31.006	Q99497	Protein deglycase DJ-1
5	5	17.5	45.456	30.987	CON__Q3SZH5	
5	5	65.3	10.5	30.981	Q9Y5L4	Mitochondrial import inner membrane translocase subunit Tim13
7	7	48.6	25.059	30.953	Q9UBQ5;Q9UBQ5-2	Eukaryotic translation initiation factor 3 subunit K
7	7	14.9	70.193	30.943	Q9NYH9	U3 small nucleolar RNA-associated protein 6 homolog
16	16	22.1	108.58	30.886	Q9H0D6;Q9H0D6-2	5-3 exoribonuclease 2
15	15	42.5	52.878	30.881	Q96KP4;Q96KP4-2	Cytosolic non-specific dipeptidase
13	13	36.4	59.755	30.868	P04040	Catalase
11	11	14.2	95.755	30.779	Q93050-1;Q93050;Q93050-3	V-type proton ATPase 116 kDa subunit a isoform 1
12	12	51	27.843	30.744	P38117;P38117-2	Electron transfer flavoprotein subunit beta
13	13	16.8	110.3	30.74	Q86W92-4;Q86W92;Q86W92-3;Q86W92-2	Liprin-beta-1
10	10	37.9	31.324	30.736	P15880	40S ribosomal protein S2
3	3	5.3	106.16	30.706	P06400	Retinoblastoma-associated protein
14	14	18.2	121.89	30.685	Q9NZB2;Q9NZB2-6;Q9NZB2-4	Constitutive coactivator of PPAR-gamma-like protein 1
9	8	20.1	61.932	30.671	Q9UBL6-2;Q9UBL6	Copine-7
11	11	34.4	39.591	30.666	P50213;P50213-2	Isocitrate dehydrogenase [NAD] subunit alpha, mitochondrial
11	11	19.9	84.936	30.647	P17480-2;P17480	Nucleolar transcription factor 1
7	7	27.8	56.527	30.588	Q7Z434;Q7Z434-4	Mitochondrial antiviral-signaling protein

3	3	8.7	50.854	30.586	Q9NS86	LanC-like protein 2
10	10	32	50.097	30.562	P30520	Adenylosuccinate synthetase isozyme 2
11	4	41	40.648	30.544	P10321;P10321-2	HLA class I histocompatibility antigen, Cw-7 alpha chain
10	10	22.4	52.743	30.516	O15269	Serine palmitoyltransferase 1
10	10	17.8	83.471	30.469	Q96RP9;Q96RP9-2	Elongation factor G, mitochondrial
9	9	43.3	17.779	30.365	P83731	60S ribosomal protein L24
4	4	22.3	26.056	30.362	Q9BVG4	Protein PBDC1
7	6	21.4	46.648	30.263	Q9UNH7;Q9UNH7-2	Sorting nexin-6;Sorting nexin-6, N-terminally processed
4	4	75	7.2053	30.21	P50151;Q9H1X3-3	Guanine nucleotide-binding protein G(I)/G(S)/G(O) subunit gamma-10
2	2	13.4	23.485	30.198	Q96FZ7	Charged multivesicular body protein 6
13	13	35	51.109	30.163	P41091;Q2VIR3;Q2VIR3-2	Eukaryotic translation initiation factor 2 subunit 3;Putative eukaryotic translation initiation factor 2 subunit 3-like protein
7	7	27.8	39.315	30.135	Q9NXG2	THUMP domain-containing protein 1
10	10	16.7	96.748	30.12	Q00653;Q00653-4;Q00653-3	Nuclear factor NF-kappa-B p100 subunit;Nuclear factor NF-kappa-B p52 subunit
15	15	16	152.78	30.042	P52701;P52701-4;P52701-2;P52701-3	DNA mismatch repair protein Msh6
9	9	12.4	120.5	30.007	O00267-2;O00267	Transcription elongation factor SPT5
8	8	43.3	25.838	29.996	P30048-2;P30048	Thioredoxin-dependent peroxide reductase, mitochondrial
20	20	14.4	195.82	29.935	P52948-5;P52948;P52948-6;P52948-2	Nuclear pore complex protein Nup98-Nup96;Nuclear pore complex protein Nup98;Nuclear pore complex protein Nup96
8	3	59.8	21.298	29.907	P01112;P01112-2	GTPase HRas;GTPase HRas, N-terminally processed
8	6	49.3	22.06	29.899	sp|Q3SX09|;CON__Q3SX09	
9	9	27.2	45.098	29.848	P09543-2;P09543	2,3-cyclic-nucleotide 3-phosphodiesterase
7	7	11	119.1	29.841	Q9Y666	Solute carrier family 12 member 7
11	11	35.5	39.829	29.758	P50552	Vasodilator-stimulated phosphoprotein
9	9	42.7	26.152	29.635	Q9NX63	MICOS complex subunit MIC19
6	6	10.6	97.967	29.631	Q05086-2;Q05086-3;Q05086	Ubiquitin-protein ligase E3A
9	9	9.3	148.63	29.571	Q6Y7W6-4;Q6Y7W6-5;Q6Y7W6;Q6Y7W6-3	PERQ amino acid-rich with GYF domain-containing protein 2
11	11	21.1	87.133	29.516	Q14694;Q14694-3;Q14694-2	Ubiquitin carboxyl-terminal hydrolase 10
7	7	23.7	28.68	29.513	P62753	40S ribosomal protein S6
8	7	21.8	63.704	29.513	O00425	Insulin-like growth factor 2 mRNA-binding protein 3
10	10	20.9	81.123	29.505	Q9H8H0	Nucleolar protein 11
9	9	24.6	49.797	29.493	Q9Y4P3	Transducin beta-like protein 2
15	15	23.6	96.221	29.485	Q9BXP5-5;Q9BXP5-4;Q9BXP5-2;Q9BXP5-3;Q9BXP5	Serrate RNA effector molecule homolog
13	13	50	29.225	29.477	P18124	60S ribosomal protein L7
8	8	64.4	14.716	29.473	P09382	Galectin-1
3	3	69.7	7.8547	29.469	Q15847	Adipogenesis regulatory factor
10	10	14.8	99.961	29.457	Q02487;Q02487-2	Desmocollin-2
10	10	31.4	46.862	29.419	Q9UKX7-2;Q9UKX7	Nuclear pore complex protein Nup50
10	10	52.9	29.717	29.41	Q14847;Q14847-2;Q14847-3	LIM and SH3 domain protein 1
12	12	21.1	88.908	29.407	Q5H9R7-3;Q5H9R7-4;Q5H9R7-6;Q5H9R7-2;Q5H9R7;Q5H9R7-5	Serine/threonine-protein phosphatase 6 regulatory subunit 3
13	2	28	64.149	29.399	P48163	NADP-dependent malic enzyme
11	11	30.4	58.486	29.397	P49419;P49419-2;P49419-4	Alpha-aminoadipic semialdehyde dehydrogenase
7	7	40.2	23.207	29.387	P52565;P52565-2	Rho GDP-dissociation inhibitor 1
9	9	18.9	49.469	29.379	O15427	Monocarboxylate transporter 4
14	14	22.6	81.627	29.373	Q9H307	Pinin
6	6	22.6	42.621	29.333	Q9Y4P1-6;Q9Y4P1;Q9Y4P1-2;Q9Y4P1-4	Cysteine protease ATG4B
13	4	39.7	49.857	29.324	Q9BUF5	Tubulin beta-6 chain
12	12	47.7	36.501	29.309	Q13347	Eukaryotic translation initiation factor 3 subunit I
11	11	29.7	60.67	29.303	P10155;P10155-3;P10155-5;P10155-4	60 kDa SS-A/Ro ribonucleoprotein
3	3	9.4	80.692	29.284	P08047;P08047-3;P08047-2	Transcription factor Sp1

18	18	11.4	252.5	29.24	Q8WYP5;Q8WYP5-3;Q8WYP5-2	Protein ELYS
6	5	27.6	25.476	29.182	Q01130;Q01130-2	Serine/arginine-rich splicing factor 2
12	12	38.2	48.392	29.129	Q6DKJ4;Q6DKJ4-3	Nucleoredoxin
10	1	40	38.58	29.065	Q15366;Q15366-2;Q15366-8;Q15366-5	Poly(rC)-binding protein 2
4	1	62.8	12.732	29.048	P41567	Eukaryotic translation initiation factor 1
9	8	13	94.34	29.02	Q9H8M5-2;Q9H8M5;Q9H8M5-3	Metal transporter CNNM2
3	3	14	36.071	29.006	O00151	PDZ and LIM domain protein 1
13	13	31.5	54.366	28.965	O75439	Mitochondrial-processing peptidase subunit beta
2	2	16.9	21.452	28.921	Q9Y3B2	Exosome complex component CSL4
5	5	11.6	71.813	28.887	Q96EK5	KIF1-binding protein
19	19	10.1	290.46	28.868	Q93008-1;Q93008	Probable ubiquitin carboxyl-terminal hydrolase FAF-X
3	3	40.2	12.473	28.83	Q15369;Q15369-2	Transcription elongation factor B polypeptide 1
4	1	20.4	34.769	28.793	P11908;P11908-2	Ribose-phosphate pyrophosphokinase 2
9	1	26.5	46.513	28.781	Q9Y383;Q96HJ9-2;Q9Y383-3	Putative RNA-binding protein Luc7-like 2
2	2	18.2	25.809	28.679	P60033	CD81 antigen
11	11	16.6	89.813	28.677	Q96T88;Q96T88-2	E3 ubiquitin-protein ligase UHRF1
11	9	14.4	113.66	28.66	Q5VTR2	E3 ubiquitin-protein ligase BRE1A
7	5	12	95.367	28.653	Q6IN85;Q6IN85-2;Q6IN85-5;Q6IN85-4	Serine/threonine-protein phosphatase 4 regulatory subunit 3A
5	5	32.6	23.384	28.629	Q13765;E9PAV3-2;E9PAV3	Nascent polypeptide-associated complex subunit alpha;Nascent polypeptide-associated complex subunit alpha, muscle-specific form
14	6	37.4	50.47	28.599	Q05639	Elongation factor 1-alpha 2
10	10	34.7	34.362	28.578	P46777	60S ribosomal protein L5
17	1	65.3	34.196	28.558	P09651-2	Heterogeneous nuclear ribonucleoprotein A1;Heterogeneous nuclear ribonucleoprotein A1, N-terminally processed
11	11	49.4	28.723	28.532	Q06323;Q06323-3;Q06323-2	Proteasome activator complex subunit 1
6	6	26.8	35.716	28.507	P04818;P04818-2	Thymidylate synthase
12	11	28.1	70.253	28.488	Q14651	Plastin-1
2	2	26	14.984	28.48	Q9BPZ3	Polyadenylate-binding protein-interacting protein 2
15	15	30.5	61.585	28.452	Q14247;Q14247-3;Q14247-2	Src substrate cortactin
10	10	35	28.494	28.444	Q02978-2;Q02978	Mitochondrial 2-oxoglutarate/malate carrier protein
12	12	14	131.7	28.437	O43847;O43847-2	Nardilysin
14	12	25	74.175	28.36	Q9UJS0;Q9UJS0-2	Calcium-binding mitochondrial carrier protein Aralar2
6	6	25.3	34.905	28.355	Q9HC07;Q9HC07-2	Transmembrane protein 165
10	10	27.7	57.293	28.336	Q01581	Hydroxymethylglutaryl-CoA synthase, cytoplasmic
8	8	28.7	52.723	28.313	Q9BXK5;Q9BXK5-2;Q9BXK5-4	Bcl-2-like protein 13
12	11	37.5	44.877	28.236	P31350;P31350-2	Ribonucleoside-diphosphate reductase subunit M2
11	11	25.2	70.698	28.201	Q16822;Q16822-3	Phosphoenolpyruvate carboxykinase [GTP], mitochondrial
15	9	34.6	60.801	28.176	P07947	Tyrosine-protein kinase Yes
3	3	14	28.763	28.167	Q9Y3A5	Ribosome maturation protein SBDS
4	4	15.3	42.243	28.161	Q9NVD7	Alpha-parvin
11	11	13.4	128.56	28.099	Q8NEN9	PDZ domain-containing protein 8
5	5	26.9	32.235	28.097	P43307;P43307-2	Translocon-associated protein subunit alpha
8	8	30	47.873	28.085	Q9UNS2;Q9UNS2-2	COP9 signalosome complex subunit 3
11	11	19.2	96.931	28.078	O60502-4;O60502;O60502-2;O60502-3	Protein O-GlcNAcase
9	9	50	33.824	28.056	P19623	Spermidine synthase
6	6	37.7	24.942	28.053	P67870	Casein kinase II subunit beta
8	8	19.7	57.398	27.958	Q06203	Amidophosphoribosyltransferase
8	7	29.3	45.547	27.954	Q52LJ0;Q52LJ0-1	Protein FAM98B
9	9	16.3	72.765	27.937	P48506	Glutamate--cysteine ligase catalytic subunit
17	8	20.3	137.92	27.926	P23634;P23634-8;P23634-6;P23634-7;P23634-4;P23634-2;P23634-5;P23634-3	Plasma membrane calcium-transporting ATPase 4

6	6	11.5	80.02	27.737	Q6PJT7-5;Q6PJT7-9;Q6PJT7-2;Q6PJT7;Q6PJT7-10;Q6PJT7-4;Q6PJT7-3;Q6PJT7-11	Zinc finger CCCH domain-containing protein 14
13	7	37.6	46.158	27.706	Q09028-3;Q09028;Q09028-4;Q09028-2	Histone-binding protein RBBP4
6	6	49.5	21.863	27.672	P32969	60S ribosomal protein L9
14	14	40.7	47.346	27.632	Q7L0Y3	Mitochondrial ribonuclease P protein 1
8	7	23.5	58.762	27.584	P27338;P27338-2	Amine oxidase [flavin-containing] B
12	9	26	62.825	27.579	Q07866-8;Q07866-2;Q07866-3;Q07866;Q07866-7;Q07866-5;Q07866-10;Q07866-6;Q07866-4;Q07866-9	Kinesin light chain 1
13	13	28.6	72.595	27.575	Q9H4A4	Aminopeptidase B
11	11	35.9	43.941	27.57	P21283	V-type proton ATPase subunit C 1
10	10	27.4	50.909	27.513	P48735;P48735-2	Isocitrate dehydrogenase [NADP], mitochondrial
7	1	29.2	32.103	27.495	Q01105-2;Q01105-3;Q01105-4;P0DME0	Protein SET;Protein SETSIP
10	10	56.4	20.776	27.469	O96000;O96000-2	NADH dehydrogenase [ubiquinone] 1 beta subcomplex subunit 10
12	12	23.8	89.677	27.438	Q13616	Cullin-1
17	17	6	480.19	27.433	Q7Z6Z7-2;Q7Z6Z7-3;Q7Z6Z7	E3 ubiquitin-protein ligase HUWE1
15	15	21.3	109	27.42	Q9Y2L1;Q9Y2L1-2	Exosome complex exonuclease RRP44
12	12	30	50.549	27.365	sp|FA49-20|;Q16181-2;Q16181	Septin-7
10	10	16.7	90.555	27.305	Q13443;Q13443-2	Disintegrin and metalloproteinase domain-containing protein 9
8	8	25.5	41.331	27.303	P50502;Q8NFI4;Q8IZP2	Hsc70-interacting protein;Putative protein FAM10A5;Putative protein FAM10A4
10	4	56.5	20.825	27.285	P61224;P61224-3;P61224-2;P61224-4;A6NIZ1	Ras-related protein Rap-1b;Ras-related protein Rap-1b-like protein
9	9	34.2	35.619	27.268	Q9H9B4	Sideroflexin-1
7	7	26.2	34.893	27.226	P05026-2;P05026	Sodium/potassium-transporting ATPase subunit beta-1
11	11	40.6	46.268	27.21	Q9BT78;Q9BT78-2	COP9 signalosome complex subunit 4
9	9	41.6	27.025	27.167	P40925-2;P40925;P40925-3	Malate dehydrogenase, cytoplasmic
9	8	27.2	55.102	27.139	Q13547	Histone deacetylase 1
9	9	58.7	24.579	27.041	P00492	Hypoxanthine-guanine phosphoribosyltransferase
8	8	56.8	13.53	26.985	P14927;P14927-2	Cytochrome b-c1 complex subunit 7
6	6	26.7	35.386	26.907	Q8TC12;Q8TC12-2;Q8TC12-3	Retinol dehydrogenase 11
9	9	15.5	87.217	26.905	Q03518	Antigen peptide transporter 1
4	4	12.6	56.025	26.89	Q96QD8;Q96QD8-2	Sodium-coupled neutral amino acid transporter 2
19	4	39	51.561	26.864	P02533;CON__P02533;sp|P02533|;sp|Q6IFX2|;CON__Q6IFX2	Keratin, type I cytoskeletal 14
16	15	15.8	161.85	26.834	Q8N3D4	EH domain-binding protein 1-like protein 1
7	7	23.9	47.611	26.829	Q92552;Q92552-2	28S ribosomal protein S27, mitochondrial
6	6	12.2	77.744	26.828	Q13033-2;Q13033	Striatin-3
9	9	50	31.462	26.826	P10768	S-formylglutathione hydrolase
14	14	34.4	53.836	26.807	P22570;P22570-7;P22570-4;P22570-5;P22570-2;P22570-3;P22570-6	NADPH:adrenodoxin oxidoreductase, mitochondrial
9	9	35.7	44.23	26.798	Q13561;Q13561-3;Q13561-2	Dynactin subunit 2
9	5	17.8	56.678	26.797	Q08170	Serine/arginine-rich splicing factor 4
11	7	13.7	145	26.783	O95819-6;O95819-4;O95819-2;O95819;O95819-5;O95819-3	Mitogen-activated protein kinase kinase kinase kinase 4
4	4	16.1	38.704	26.754	P35659-2;P35659	Protein DEK
4	4	11.5	77.456	26.718	sp|E16046|;CON__ENSEMBL:ENSBTAP00000016046	
9	9	21	69.305	26.687	Q9NXH9-2;Q9NXH9	tRNA (guanine(26)-N(2))-dimethyltransferase
12	12	28.2	58.252	26.659	Q10713;Q10713-2	Mitochondrial-processing peptidase subunit alpha
10	10	39.1	38.868	26.568	O14745	Na(+)/H(+) exchange regulatory cofactor NHE-RF1
9	9	15.7	73.602	26.519	Q14978;Q14978-2;Q14978-3	Nucleolar and coiled-body phosphoprotein 1
14	14	26.6	86.47	26.517	Q08J23;Q08J23-2;Q08J23-3	tRNA (cytosine(34)-C(5))-methyltransferase

11	11	24.6	66.558	26.504	P08240-2;P08240	Signal recognition particle receptor subunit alpha
7	7	44.2	22.119	26.5	P30043	Flavin reductase (NADPH)
11	11	29.8	54.785	26.471	Q5T8D3-2;Q5T8D3-3;Q5T8D3;Q5T8D3-4	Acyl-CoA-binding domain-containing protein 5
9	9	31.6	28.908	26.451	P24539	ATP synthase F(0) complex subunit B1, mitochondrial
10	10	21.6	72.063	26.435	Q6P1M0;Q6P1M0-2	Long-chain fatty acid transport protein 4
7	7	37.9	20.7	26.417	P55145	Mesencephalic astrocyte-derived neurotrophic factor
10	10	16.5	91.35	26.373	O43747;O43747-2	AP-1 complex subunit gamma-1
11	11	17.5	108.38	26.372	Q96T76-9;Q96T76;Q96T76-8;Q96T76-5	MMS19 nucleotide excision repair protein homolog
9	9	33	31.512	26.341	P54709;P54709-2	Sodium/potassium-transporting ATPase subunit beta-3
8	8	33.7	34.994	26.334	Q16698-2;Q16698	2,4-dienoyl-CoA reductase, mitochondrial
6	1	41.8	18.742	26.332	P62633-2;P62633;P62633-7;P62633-3;P62633-5;P62633-6	Cellular nucleic acid-binding protein
7	7	30.6	35.808	26.3	Q96DB5;Q96DB5-2;Q96DB5-3	Regulator of microtubule dynamics protein 1
4	4	18.9	27.325	26.21	Q99426;Q99426-2	Tubulin-folding cofactor B
10	10	24.8	53.41	26.2	Q9H6Z4-3;Q9H6Z4;Q9H6Z4-2	Ran-binding protein 3
11	11	17.7	98.088	26.184	Q01780-2;Q01780	Exosome component 10
9	9	49.7	21.228	26.161	Q9HB71-3;Q9HB71	Calcyclin-binding protein
13	13	10.9	159.83	26.12	Q96PC5;Q96PC5-14;Q96PC5-11;Q96PC5-5;Q96PC5-8;Q96PC5-10;Q96PC5-9;Q96PC5-7;Q96PC5-13;Q96PC5-12;Q96PC5-6	Melanoma inhibitory activity protein 2
6	6	16.5	53.254	26.106	P37198	Nuclear pore glycoprotein p62
7	6	24.5	46.897	26.103	sp|X52647|;CON__REFSEQ:XP_001252647	
14	14	17.2	118.69	26.029	P46379-2;P46379;P46379-3;P46379-5;P46379-4	Large proline-rich protein BAG6
7	3	47.6	18.736	25.973	Q9Y281;Q9Y281-3	Cofilin-2
11	11	47.1	38.449	25.928	Q9UBE0;Q9UBE0-2;Q9UBE0-3	SUMO-activating enzyme subunit 1;SUMO-activating enzyme subunit 1, N-terminally processed
9	9	12.2	106.37	25.908	P57740;P57740-2;P57740-3	Nuclear pore complex protein Nup107
10	10	69.6	18.491	25.894	O75947;O75947-2	ATP synthase subunit d, mitochondrial
8	8	16.5	76.482	25.884	P13807-2;P13807	Glycogen [starch] synthase, muscle
8	8	39.4	23.742	25.872	P23284	Peptidyl-prolyl cis-trans isomerase B
14	9	16.3	122.87	25.79	Q92922	SWI/SNF complex subunit SMARCC1
9	7	16.8	97.621	25.774	Q9UBF2;Q9UBF2-2	Coatomer subunit gamma-2
10	10	48.4	36.079	25.757	Q9Y5K5-2;Q9Y5K5-4;Q9Y5K5-3;Q9Y5K5	Ubiquitin carboxyl-terminal hydrolase isozyme L5
3	3	7.8	57.258	25.751	Q96RE7	Nucleus accumbens-associated protein 1
7	7	34	23.577	25.737	P40429	60S ribosomal protein L13a
7	7	8.4	135.62	25.697	Q27J81;Q27J81-2	Inverted formin-2
10	9	23.1	54.847	25.687	P51648;P51648-2	Fatty aldehyde dehydrogenase
10	10	16.5	80.529	25.656	P46977;P46977-2	Dolichyl-diphosphooligosaccharide--protein glycosyltransferase subunit STT3A
3	3	32.4	19.76	25.655	Q9Y5S9-2;Q9Y5S9	RNA-binding protein 8A
4	4	13.6	52.197	25.645	P49768-2;P49768;P49768-3;P49768-5;P49768-6;P49768-7;P49768-4	Presenilin-1;Presenilin-1 NTF subunit;Presenilin-1 CTF subunit;Presenilin-1 CTF12
12	9	49.8	30.54	25.536	Q13162	Peroxiredoxin-4
5	5	31.3	24.604	25.523	P27635	60S ribosomal protein L10
9	9	29.9	47.371	25.489	P61158	Actin-related protein 3
15	9	43.5	42.123	25.321	P29992	Guanine nucleotide-binding protein subunit alpha-11
12	12	17.8	102.89	25.319	Q8WWM7-6;Q8WWM7-8;Q8WWM7-5;Q8WWM7-4;Q8WWM7-9;Q8WWM7-2;Q8WWM7;Q8WWM7-3;Q8WWM7-7	Ataxin-2-like protein
4	4	37.3	20.43	25.296	Q8NCW5-2;Q8NCW5	NAD(P)H-hydrate epimerase
8	7	12.6	78.832	25.283	Q9NVV4-2;Q9NVV4	Poly(A) RNA polymerase, mitochondrial
12	12	15.9	111.08	25.245	Q9Y6Y8;Q9Y6Y8-2	SEC23-interacting protein
7	7	34.5	33.83	25.235	P46108;P46108-2	Adapter molecule crk

8	8	34.6	37.419	25.23	O00154-4;O00154-6;O00154-7;O00154;O00154-2;O00154-3;O00154-5	Cytosolic acyl coenzyme A thioester hydrolase
12	12	25.1	72.683	25.222	O14744;O14744-5;O14744-2;O14744-3;O14744-4	Protein arginine N-methyltransferase 5;Protein arginine N-methyltransferase 5, N-terminally processed
6	6	36.1	33.58	25.174	Q9GZS3	WD repeat-containing protein 61;WD repeat-containing protein 61, N-terminally processed
12	12	37.9	61.277	25.079	Q15942;Q15942-2	Zyxin
23	3	63	49.895	25.073	Q9BQE3	Tubulin alpha-1C chain
8	8	23.1	55.299	25.035	Q9Y6M5	Zinc transporter 1
2	2	16.5	20.48	25.007	Q96B54	Zinc finger protein 428
8	7	27.9	24.893	24.99	P09429;B2RPK0	High mobility group protein B1;Putative high mobility group protein B1-like 1
13	12	13.8	137.17	24.955	Q6WKZ4;Q6WKZ4-3;Q6WKZ4-2	Rab11 family-interacting protein 1
8	8	26.2	51.596	24.914	P61201;P61201-2	COP9 signalosome complex subunit 2
5	3	18.7	40.057	24.894	P13747	HLA class I histocompatibility antigen, alpha chain E
8	8	21.2	58.946	24.88	Q3LXA3;Q3LXA3-2	Bifunctional ATP-dependent dihydroxyacetone kinase/FAD-AMP lyase (cyclizing);ATP-dependent dihydroxyacetone kinase;FAD-AMP lyase (cyclizing)
10	10	33.1	37.514	24.853	P11177-3;P11177-2;P11177	Pyruvate dehydrogenase E1 component subunit beta, mitochondrial
7	7	35.4	29.634	24.851	O60762	Dolichol-phosphate mannosyltransferase subunit 1
17	17	17.6	147.18	24.82	Q9NTJ3;Q9NTJ3-2	Structural maintenance of chromosomes protein 4
5	5	50	16.62	24.814	Q4VC31	Coiled-coil domain-containing protein 58
10	10	22.4	68.436	24.778	Q06124-1;Q06124;Q06124-3	Tyrosine-protein phosphatase non-receptor type 11
5	5	7.2	114.54	24.766	P08648	Integrin alpha-5;Integrin alpha-5 heavy chain;Integrin alpha-5 light chain
10	10	73.2	17.748	24.739	P33316-2;P33316	Deoxyuridine 5-triphosphate nucleotidohydrolase, mitochondrial
9	9	20.8	54.177	24.721	P09622;P09622-2;P09622-3	Dihydrolipoyl dehydrogenase, mitochondrial
10	10	9.3	162.12	24.72	Q12769	Nuclear pore complex protein Nup160
3	3	10.2	54.557	24.715	Q9NQZ2	Something about silencing protein 10
12	12	29	52.347	24.713	CON__P41361;sp|P41361|	
10	10	43.8	30.628	24.71	P47756-2;P47756	F-actin-capping protein subunit beta
11	9	24.3	54.846	24.703	P12081-4;P12081;P12081-3;P12081-2	Histidine--tRNA ligase, cytoplasmic
9	9	6.6	250.53	24.692	Q6UVK1	Chondroitin sulfate proteoglycan 4
2	2	32.9	9.4275	24.604	Q5JTJ3-3;Q5JTJ3;Q5JTJ3-2	Cytochrome c oxidase assembly factor 6 homolog
3	3	27.9	17.218	24.569	Q9H4G4	Golgi-associated plant pathogenesis-related protein 1
8	8	40.2	28.993	24.554	P30040	Endoplasmic reticulum resident protein 29
3	3	40.7	11.951	24.533	P62942	Peptidyl-prolyl cis-trans isomerase FKBP1A
32	1	75.5	44.091	24.519	CON__P08727;sp|P08727|	
4	4	29.4	17.493	24.442	P84157-2;P84157-3;P84157	Matrix-remodeling-associated protein 7
5	5	26.5	14.57	24.416	P37108	Signal recognition particle 14 kDa protein
9	9	32.2	32.996	24.401	P36542;P36542-2	ATP synthase subunit gamma, mitochondrial
15	15	41.8	55.52	24.369	P43490	Nicotinamide phosphoribosyltransferase
10	10	27.1	48.115	24.363	P37268;P37268-2;P37268-4;P37268-3;P37268-5	Squalene synthase
11	11	12.6	145.81	24.339	Q14692	Ribosome biogenesis protein BMS1 homolog
18	1	26.4	62.378	24.327	sp|P13647|;CON__P13647;P13647	Keratin, type II cytoskeletal 5
11	11	15.5	98.398	24.295	P21399	Cytoplasmic aconitate hydratase
7	5	15.4	53.354	24.276	P35637-2;P35637	RNA-binding protein FUS
12	12	32.5	47.064	24.206	Q14254	Flotillin-2
6	6	20	39.383	24.188	P46976;P46976-2;P46976-3	Glycogenin-1
4	4	16.9	41.096	24.187	Q5W0Z9-3;Q5W0Z9;Q5W0Z9-4	Probable palmitoyltransferase ZDHHC20
6	6	34.6	28.024	24.152	P62917	60S ribosomal protein L8
6	6	19.7	46.609	24.146	Q96B97-3;Q96B97-2;Q96B97	SH3 domain-containing kinase-binding protein 1
9	9	22.6	75.019	24.127	Q9BW27;Q9BW27-3;Q9BW27-2	Nuclear pore complex protein Nup85
11	11	58	24.648	24.125	P54819-5;P54819-2;P54819;P54819-3;P54819-6;P54819-4	Adenylate kinase 2, mitochondrial;Adenylate kinase 2, mitochondrial, N-terminally processed

5	5	40	22.826	24.101	Q9NRW3	DNA dC->dU-editing enzyme APOBEC-3C
6	6	24.8	43.66	24.047	P31153;P31153-2	S-adenosylmethionine synthase isoform type-2
9	9	15.1	91.08	24.015	Q9UDY8-2;Q9UDY8	Mucosa-associated lymphoid tissue lymphoma translocation protein 1
5	5	40.8	18.416	24.006	Q9H910-2;Q9H910;Q9H910-3	Hematological and neurological expressed 1-like protein
7	7	23.7	45.468	23.971	Q9HCU5	Prolactin regulatory element-binding protein
11	11	32	40.188	23.948	P08559-3;P08559;P08559-2;P08559-4	Pyruvate dehydrogenase E1 component subunit alpha, somatic form, mitochondrial
8	8	54.5	21.896	23.883	Q14116-2;Q14116	Interleukin-18
8	8	53.5	20.105	23.795	P51970	NADH dehydrogenase [ubiquinone] 1 alpha subcomplex subunit 8
12	12	12.2	162.23	23.771	Q14C86-5;Q14C86;Q14C86-3;Q14C86-4;Q14C86-2;Q14C86-6	GTPase-activating protein and VPS9 domain-containing protein 1
7	7	16.5	62.924	23.683	O15270	Serine palmitoyltransferase 2
7	7	23.7	48.227	23.682	Q07666;Q07666-2;Q07666-3	KH domain-containing, RNA-binding, signal transduction-associated protein 1
10	10	19.5	78.457	23.65	Q8NC60	Nitric oxide-associated protein 1
8	7	17.8	80.785	23.638	Q13330;Q13330-3;Q13330-2	Metastasis-associated protein MTA1
11	10	52	31.54	23.616	P48739;P48739-2;P48739-3	Phosphatidylinositol transfer protein beta isoform
13	13	35.1	56.613	23.581	Q96RS6-3;Q96RS6-2;Q96RS6	NudC domain-containing protein 1
6	6	22.5	43.215	23.573	P53582	Methionine aminopeptidase 1
8	8	28.6	37.563	23.567	O00303	Eukaryotic translation initiation factor 3 subunit F
12	12	34.6	61.594	23.525	O95817	BAG family molecular chaperone regulator 3
6	6	17.6	51.545	23.481	Q92879-2;Q92879-3;Q92879-6;Q92879;Q92879-4;Q92879-5	CUGBP Elav-like family member 1
9	9	25.5	39.958	23.474	Q00325-2;Q00325	Phosphate carrier protein, mitochondrial
10	10	9.7	160.88	23.446	Q10570	Cleavage and polyadenylation specificity factor subunit 1
7	7	26	41.213	23.444	P19784	Casein kinase II subunit alpha
13	13	40.7	52.73	23.417	Q6ZMU5;Q6ZMU5-2	Tripartite motif-containing protein 72
9	9	29.8	40.228	23.397	Q12907	Vesicular integral-membrane protein VIP36
12	3	56.6	30.137	23.352	P22392-2;P22392	Nucleoside diphosphate kinase B
8	8	34.6	37.92	23.282	O15126;O15126-2	Secretory carrier-associated membrane protein 1
10	10	7.6	217.17	23.27	P24928	DNA-directed RNA polymerase II subunit RPB1
7	7	26.5	34.063	23.235	O43765	Small glutamine-rich tetratricopeptide repeat-containing protein alpha
9	8	11.8	111.02	23.204	Q5T8P6-2;Q5T8P6;Q5T8P6-3;Q5T8P6-5;Q5T8P6-4	RNA-binding protein 26
9	9	10.3	114.74	23.187	Q08174;Q08174-2	Protocadherin-1
10	10	25.7	61.888	23.186	Q15637-6;Q15637-4;Q15637-3;Q15637-2;Q15637;Q15637-5;Q15637-7	Splicing factor 1
5	5	16	55.278	23.17	O75907	Diacylglycerol O-acyltransferase 1
12	12	32.7	62.288	23.126	Q9BY44-3;Q9BY44;Q9BY44-4	Eukaryotic translation initiation factor 2A;Eukaryotic translation initiation factor 2A, N-terminally processed
9	9	11.7	144.68	23.097	O60271-4;O60271-2;O60271;O60271-9;O60271-5;O60271-3	C-Jun-amino-terminal kinase-interacting protein 4
3	3	25	29.204	23.081	P28070	Proteasome subunit beta type-4
8	8	33.6	24.261	23.07	P26373;P26373-2	60S ribosomal protein L13
7	7	21.8	44.648	23.029	Q14318-2;Q14318;Q14318-3	Peptidyl-prolyl cis-trans isomerase FKBP8
16	16	16.8	143.23	23.008	Q14683	Structural maintenance of chromosomes protein 1A
7	7	19.8	52.838	23.004	Q15043-2;Q15043-3;Q15043	Zinc transporter ZIP14
8	8	21.6	54.122	22.931	O00330;O00330-3	Pyruvate dehydrogenase protein X component, mitochondrial
3	3	29.7	18.325	22.901	Q8N5N7	39S ribosomal protein L50, mitochondrial
6	6	24.5	57.193	22.862	O43660;O43660-2	Pleiotropic regulator 1
13	13	16	118.29	22.855	O15042;O15042-2;O15042-3	U2 snRNP-associated SURP motif-containing protein
9	9	13.7	97.173	22.839	Q08345-2;Q08345-6;Q08345;Q08345-5;Q08345-4	Epithelial discoidin domain-containing receptor 1
8	8	39.3	24.327	22.838	Q9UKK9	ADP-sugar pyrophosphatase

8	8	32.9	39.935	22.821	Q6P6B1	Glutamate-rich protein 5
14	14	40.5	46.51	22.781	Q96I99;Q96I99-2	Succinyl-CoA ligase [GDP-forming] subunit beta, mitochondrial
9	9	17.8	68.996	22.781	P10515	Dihydrolipoyllysine-residue acetyltransferase component of pyruvate dehydrogenase complex, mitochondrial
8	8	48	17.718	22.777	P62269	40S ribosomal protein S18
12	12	19.8	88.929	22.758	Q13618;Q13618-2;Q13618-3	Cullin-3
12	12	42.6	47.707	22.732	Q9GZL7	Ribosome biogenesis protein WDR12
13	13	28.4	73.964	22.713	Q9BZE4;Q9BZE4-2;Q9BZE4-3	Nucleolar GTP-binding protein 1
5	5	38.1	25.65	22.688	P82930	28S ribosomal protein S34, mitochondrial
10	7	42.2	40.089	22.628	P16989;P16989-2;P16989-3	Y-box-binding protein 3
9	9	7	211.68	22.618	P10586-2;P10586	Receptor-type tyrosine-protein phosphatase F
4	4	34.8	21.348	22.615	P52815	39S ribosomal protein L12, mitochondrial
8	8	29.1	46.51	22.594	Q9NR12-2;Q9NR12	PDZ and LIM domain protein 7
11	10	26.4	61.842	22.576	Q9Y6M1-1;Q9Y6M1;Q9Y6M1-5;Q9Y6M1-6;Q9Y6M1-3;Q9Y6M1-4	Insulin-like growth factor 2 mRNA-binding protein 2
6	4	20.8	56.369	22.566	Q13557;Q13557-6;Q13557-12;Q13557-8;Q13557-11;Q13557-10;Q13557-9;Q13557-4;Q13557-5;Q13557-3	Calcium/calmodulin-dependent protein kinase type II subunit delta
12	12	32.7	51.691	22.561	P63151;P63151-2;Q66LE6	Serine/threonine-protein phosphatase 2A 55 kDa regulatory subunit B alpha isoform;Serine/threonine-protein phosphatase 2A 55 kDa regulatory subunit B delta isoform
3	3	20.2	28.275	22.533	Q8NBZ7-3;Q8NBZ7;Q8NBZ7-2	UDP-glucuronic acid decarboxylase 1
8	5	56.7	22.178	22.516	P20339-2;P20339	Ras-related protein Rab-5A
10	10	18.5	89.42	22.513	P22059	Oxysterol-binding protein 1
9	9	25	41.28	22.485	P82650	28S ribosomal protein S22, mitochondrial
11	10	31.2	46.816	22.465	Q9Y5X3	Sorting nexin-5
7	7	22.5	34.012	22.404	P53007	Tricarboxylate transport protein, mitochondrial
6	6	31.1	33.327	22.384	Q9UK22	F-box only protein 2
9	9	10.9	123.91	22.34	Q9UIA9	Exportin-7
12	11	41	27.893	22.32	Q9P0L0;Q9P0L0-2	Vesicle-associated membrane protein-associated protein A
12	12	24.4	68.6	22.293	P15170-2;P15170-3;P15170;Q8IYD1	Eukaryotic peptide chain release factor GTP-binding subunit ERF3A;Eukaryotic peptide chain release factor GTP-binding subunit ERF3B
11	11	34.7	47.355	22.251	O75955;O75955-2	Flotillin-1
5	5	34.3	19.198	22.248	Q13405	39S ribosomal protein L49, mitochondrial
10	10	21.7	57.089	22.232	Q14498-3;Q14498-2;Q14498	RNA-binding protein 39
8	8	21.5	77.859	22.192	Q8IY67-2;Q8IY67	Ribonucleoprotein PTB-binding 1
8	8	32.6	35.39	22.158	P43897;P43897-2	Elongation factor Ts, mitochondrial
6	6	54.4	14.285	22.147	O14737;O14737-2	Programmed cell death protein 5
9	9	22.8	48.879	22.085	P55084-2;P55084	Trifunctional enzyme subunit beta, mitochondrial;3-ketoacyl-CoA thiolase
9	9	31	39.724	22.075	P11766	Alcohol dehydrogenase class-3
4	3	19.8	33.433	22.019	P54725-2;P54725-3;P54725	UV excision repair protein RAD23 homolog A
3	3	8.6	58.135	21.936	O95251-2;O95251-4;O95251;O95251-3;O95251-5	Histone acetyltransferase KAT7
15	15	13.6	157.71	21.935	P08581-2;P08581	Hepatocyte growth factor receptor
13	13	9.9	208.73	21.899	P51610;P51610-4;P51610-2;P51610-3	Host cell factor 1;HCF N-terminal chain 1;HCF N-terminal chain 2;HCF N-terminal chain 3;HCF N-terminal chain 4;HCF N-terminal chain 5;HCF N-terminal chain 6;HCF C-terminal chain 1;HCF C-terminal chain 2;HCF C-terminal chain 3;HCF C-terminal chain 4;HCF C-terminal chain 5;HCF C-terminal chain 6
9	9	24.3	50.464	21.896	Q3ZCQ8-2;Q3ZCQ8;Q3ZCQ8-3	Mitochondrial import inner membrane translocase subunit TIM50
12	12	21.4	94.498	21.888	Q8IWA0	WD repeat-containing protein 75
14	14	14.4	151.66	21.874	Q6P2E9;Q6P2E9-2	Enhancer of mRNA-decapping protein 4
7	7	25.8	44.971	21.861	Q92783-2;Q92783	Signal transducing adapter molecule 1
9	9	28.3	40.744	21.856	Q8NBU5;Q8NBU5-2	ATPase family AAA domain-containing protein 1
6	6	27.4	35.329	21.828	P31937	3-hydroxyisobutyrate dehydrogenase, mitochondrial

12	11	22.9	64.732	21.823	Q10471	Polypeptide N-acetylgalactosaminyltransferase 2;Polypeptide N-acetylgalactosaminyltransferase 2 soluble form
3	3	7.6	47.405	21.788	Q9BV38	WD repeat-containing protein 18
2	2	2.3	136.68	21.786	Q9BZH6	WD repeat-containing protein 11
2	2	9.5	38.143	21.784	O00180	Potassium channel subfamily K member 1
6	6	27.1	40.822	21.74	Q8IVD9	NudC domain-containing protein 3
7	7	28.7	48.112	21.739	Q9NQH7-2;Q9NQH7-4;Q9NQH7	Probable Xaa-Pro aminopeptidase 3
4	4	7.7	112.7	21.72	O75717-2;O75717	WD repeat and HMG-box DNA-binding protein 1
8	8	53.4	18.658	21.705	P63208;P63208-2	S-phase kinase-associated protein 1
6	6	33.7	30.608	21.695	Q9NQR4	Omega-amidase NIT2
13	13	20.9	104.11	21.683	Q86XL3;Q86XL3-2	Ankyrin repeat and LEM domain-containing protein 2
7	1	22.3	49.775	21.674	Q3ZCM7	Tubulin beta-8 chain
5	5	17.6	48.349	21.651	O75616	GTPase Era, mitochondrial
12	10	32.1	59.241	21.646	Q9BTT6;Q9BTT6-2	Leucine-rich repeat-containing protein 1
15	15	8.5	271.69	21.631	Q5UIP0-2;Q5UIP0	Telomere-associated protein RIF1
9	9	30.4	55.092	21.611	Q13098-5;Q13098;Q13098-7;Q13098-6	COP9 signalosome complex subunit 1
6	6	22.2	44.292	21.585	P09110;P09110-2	3-ketoacyl-CoA thiolase, peroxisomal
12	12	14.8	150.25	21.562	O95163	Elongator complex protein 1
8	8	25.4	50.983	21.556	P56181-2	
3	3	21.3	20.016	21.543	P32321;P32321-2	Deoxycytidylate deaminase
9	8	42.6	27.191	21.53	O14979-3;O14979-2;O14979	Heterogeneous nuclear ribonucleoprotein D-like
5	5	18.9	56.777	21.497	Q92575	UBX domain-containing protein 4
8	8	23.5	56.157	21.49	P55809	Succinyl-CoA:3-ketoacid coenzyme A transferase 1, mitochondrial
8	8	21.4	48.602	21.476	Q8WU90	Zinc finger CCCH domain-containing protein 15
13	13	36.2	45.531	21.451	Q15008;Q15008-4;Q15008-3;Q15008-2	26S proteasome non-ATPase regulatory subunit 6
9	2	18.3	68.709	21.448	sp|P01045-1|;CON__P01045-1;sp|P01044-1|;CON__P01044-1	
7	7	24.2	40.349	21.434	P48637-2;P48637	Glutathione synthetase
8	8	39.5	22.949	21.424	P49720	Proteasome subunit beta type-3
11	11	25.8	74.423	21.409	Q15067;Q15067-2;Q15067-3	Peroxisomal acyl-coenzyme A oxidase 1
6	6	12.4	66.69	21.403	P17812;P17812-2	CTP synthase 1
6	6	25.7	38.946	21.397	O00743-3;O00743;O00743-2	Serine/threonine-protein phosphatase 6 catalytic subunit;Serine/threonine-protein phosphatase 6 catalytic subunit, N-terminally processed
9	9	16.3	77.528	21.395	Q6ZRP7	Sulfhydryl oxidase 2
3	3	16.4	33.1	21.364	Q9UNW1-4;Q9UNW1	Multiple inositol polyphosphate phosphatase 1
11	11	26.1	55.21	21.343	Q9Y3I0	tRNA-splicing ligase RtcB homolog
8	8	33.1	38.324	21.335	Q00796;Q00796-2	Sorbitol dehydrogenase
6	3	52.4	16.363	21.315	Q15819;Q13404-8	Ubiquitin-conjugating enzyme E2 variant 2;Ubiquitin-conjugating enzyme E2 variant 1
8	8	21.5	42.331	21.239	P38159;P38159-2;Q96E39;P38159-3	RNA-binding motif protein, X chromosome;RNA-binding motif protein, X chromosome, N-terminally processed;RNA binding motif protein, X-linked-like-1
8	7	27	45.57	21.227	Q8WXF1-2;Q8WXF1	Paraspeckle component 1
5	5	42	15.641	21.196	Q96C01	Protein FAM136A
6	6	17.8	44.508	21.151	P32929;P32929-2;P32929-3	Cystathionine gamma-lyase
4	4	8.8	63.514	21.129	Q6PML9	Zinc transporter 9
7	7	35.5	19.595	21.126	P13693;P13693-2	Translationally-controlled tumor protein
11	9	20.7	86.478	21.086	Q15437	Protein transport protein Sec23B
5	5	36.3	15.278	21.052	Q8N5K1	CDGSH iron-sulfur domain-containing protein 2
6	6	16.7	44.302	21.01	Q9NZL4-3;Q9NZL4;Q9NZL4-2	Hsp70-binding protein 1
8	8	49.7	22.313	20.995	P37235;P84074	Hippocalcin-like protein 1;Neuron-specific calcium-binding protein hippocalcin
8	7	20.1	68.117	20.991	Q8IXI1;Q8IXI1-2	Mitochondrial Rho GTPase 2
2	2	24.1	12.274	20.921	P62877	E3 ubiquitin-protein ligase RBX1;E3 ubiquitin-protein ligase RBX1, N-terminally processed
5	5	44.1	19.193	20.873	Q9Y3E5	Peptidyl-tRNA hydrolase 2, mitochondrial

7	7	50.7	15.079	20.85	P22307-6;P22307-2;P22307-4;P22307-7;P22307-8;P22307	Non-specific lipid-transfer protein
10	1	13.3	105.93	20.843	O75400-2;O75400	Pre-mRNA-processing factor 40 homolog A
8	6	47.7	17.302	20.828	P16949;P16949-2	Stathmin
6	6	13.8	57.196	20.821	Q9NVH1-3;Q9NVH1;Q9NVH1-2	DnaJ homolog subfamily C member 11
2	2	20.2	24.377	20.796	O00559;O00559-2	Receptor-binding cancer antigen expressed on SiSo cells
7	7	42.6	16.561	20.752	P46776	60S ribosomal protein L27a
20	9	10.6	287.28	20.73	Q14315-2;Q14315	Filamin-C
8	8	29.2	38.298	20.729	Q9Y394;Q9Y394-2	Dehydrogenase/reductase SDR family member 7
12	12	17.7	94.584	20.687	Q9NXE4-2;Q9NXE4;Q9NXE4-4;Q9NXE4-3;Q9NXE4-6;Q9NXE4-8;Q9NXE4-9;Q9NXE4-10;Q9NXE4-7;Q9NXE4-5	Sphingomyelin phosphodiesterase 4
11	11	26.2	57.658	20.667	Q08209-2;Q08209;Q08209-5;Q08209-3	Serine/threonine-protein phosphatase 2B catalytic subunit alpha isoform
9	6	23.1	58.022	20.621	O95747	Serine/threonine-protein kinase OSR1
2	2	13.6	21.739	20.568	Q96AT9-4;Q96AT9-2;Q96AT9;Q96AT9-5;Q96AT9-3;Q2QD12	Ribulose-phosphate 3-epimerase;Ribulose-phosphate 3-epimerase-like protein 1
5	5	14.9	49.96	20.556	Q9Y6N5	Sulfide:quinone oxidoreductase, mitochondrial
4	4	41.9	22.149	20.549	Q9H3Z4;Q9H3Z4-2	DnaJ homolog subfamily C member 5
6	6	19.6	50.925	20.528	O75312	Zinc finger protein ZPR1
7	7	29.8	36.413	20.455	Q15417;Q15417-2;Q15417-3	Calponin-3
7	4	21.3	47.913	20.454	Q9Y6E0-2;Q9Y6E0	Serine/threonine-protein kinase 24;Serine/threonine-protein kinase 24 36 kDa subunit;Serine/threonine-protein kinase 24 12 kDa subunit
8	8	32.2	33.232	20.441	Q9HC38-2;Q9HC38;Q9HC38-3	Glyoxalase domain-containing protein 4
11	5	43.5	37.331	20.383	P62879	Guanine nucleotide-binding protein G(I)/G(S)/G(T) subunit beta-2
13	7	27.4	69.578	20.368	Q13310-2;Q13310;Q13310-3	Polyadenylate-binding protein 4
8	8	31.1	30.84	20.357	Q13151	Heterogeneous nuclear ribonucleoprotein A0
6	6	31.2	25.734	20.336	Q15102	Platelet-activating factor acetylhydrolase IB subunit gamma
6	6	29.2	27.872	20.328	P0DN76;Q01081;Q01081-2;Q01081-4;Q8WU68-3;Q8WU68	Splicing factor U2AF 35 kDa subunit;Splicing factor U2AF 26 kDa subunit
3	3	10.2	55.455	20.298	Q8WWY3	U4/U6 small nuclear ribonucleoprotein Prp31
6	6	21.6	46.674	20.265	Q9ULX3	RNA-binding protein NOB1
11	11	17.4	77.15	20.225	Q6NUQ4;Q6NUQ4-2	Transmembrane protein 214
7	7	44.5	22.142	20.219	P62760	Visinin-like protein 1
6	6	9.1	135.6	20.205	P04920-2;P04920-3;P04920	Anion exchange protein 2
7	7	39.4	21.732	20.198	Q92572	AP-3 complex subunit sigma-1
10	6	64.4	20.511	20.197	P18085	ADP-ribosylation factor 4
8	8	16.2	52.264	20.183	P61619;P61619-3	Protein transport protein Sec61 subunit alpha isoform 1
7	7	16.9	64.868	20.18	P41440;P41440-2;P41440-3	Folate transporter 1
14	14	14	142.06	20.179	Q52LW3	Rho GTPase-activating protein 29
8	8	25.1	59.544	20.102	Q86XZ4	Spermatogenesis-associated serine-rich protein 2
9	9	13.9	100.18	20.092	P49756	RNA-binding protein 25
7	6	50.8	21.671	20.037	O00264;O00264-2	Membrane-associated progesterone receptor component 1
7	7	16.3	65.653	20.02	Q5BKZ1	DBIRD complex subunit ZNF326
9	9	16.5	88.053	19.965	P18084	Integrin beta-5
7	7	15.2	64.615	19.954	O14975-2;O14975	Very long-chain acyl-CoA synthetase
5	4	21.1	37.806	19.945	Q9UDY4	DnaJ homolog subfamily B member 4
9	9	47.1	25.913	19.944	Q7Z4W1	L-xylulose reductase
8	8	17.7	66.855	19.936	Q9UJX3;Q9UJX3-2	Anaphase-promoting complex subunit 7
9	9	45.5	33.172	19.93	Q9Y314	Nitric oxide synthase-interacting protein
8	8	23.5	45.745	19.897	O60884	DnaJ homolog subfamily A member 2

12	10	18.3	95.165	19.895	Q12959-5;Q12959-3;Q12959-6;Q12959-4;Q12959;Q12959-7;Q12959-2;Q12959-8;Q12959-9	Disks large homolog 1
13	10	30.3	74.435	19.874	O60488-2;O60488	Long-chain-fatty-acid--CoA ligase 4
12	12	43.1	41.401	19.862	Q8TDN6	Ribosome biogenesis protein BRX1 homolog
9	9	11	119.76	19.837	Q9HBR0	Putative sodium-coupled neutral amino acid transporter 10
13	13	26.5	83.549	19.813	Q02809;Q02809-2	Procollagen-lysine,2-oxoglutarate 5-dioxygenase 1
3	1	23.8	22.168	19.805	P20290	Transcription factor BTF3
6	6	35.8	28.17	19.796	P0DPI2;A0A0B4J2D5;P0DPI2-2;A0A0B4J2D5-2	
6	3	23.7	42.613	19.765	P61163	Alpha-centractin
5	5	22.7	29.965	19.754	Q99436	Proteasome subunit beta type-7
4	4	6.3	101.27	19.731	Q9Y5Q9;Q9Y5Q9-2	General transcription factor 3C polypeptide 3
5	5	36.1	18.205	19.72	Q9Y3B7-2;Q9Y3B7;Q9Y3B7-3	39S ribosomal protein L11, mitochondrial
8	5	59.6	20.745	19.695	Q9Y3L5	Ras-related protein Rap-2c
4	3	43.6	12.975	19.688	O43768-2;O43768;O43768-9;O43768-3;O43768-4;O43768-6;O43768-5;O43768-7;O43768-8	Alpha-endosulfine
4	4	45.4	18.848	19.672	P14209;P14209-3;P14209-2	CD99 antigen
5	5	31.2	31.705	19.658	Q9H2W6	39S ribosomal protein L46, mitochondrial
9	9	35.8	32.583	19.645	Q9Y624;Q9Y624-2	Junctional adhesion molecule A
10	10	23.3	65.443	19.627	P23368;P23368-2	NAD-dependent malic enzyme, mitochondrial
6	6	20.1	42.153	19.611	Q16186	Proteasomal ubiquitin receptor ADRM1
3	3	20.9	25.249	19.61	Q9NQT4	Exosome complex component RRP46
6	5	29.4	31.279	19.592	P09012	U1 small nuclear ribonucleoprotein A
7	7	14.2	59.151	19.591	Q8NF37	Lysophosphatidylcholine acyltransferase 1
6	6	64.9	15.936	19.57	P00441	Superoxide dismutase [Cu-Zn]
7	7	27.1	48.357	19.564	Q05048	Cleavage stimulation factor subunit 1
4	4	11	59.844	19.496	Q9UHY1	Nuclear receptor-binding protein
7	7	15.7	70.294	19.476	Q14677-3;Q14677-2;Q14677	Clathrin interactor 1
15	15	27.4	68.76	19.475	Q9H845	Acyl-CoA dehydrogenase family member 9, mitochondrial
4	4	30.2	22.814	19.442	Q7Z2W9;Q7Z2W9-2	39S ribosomal protein L21, mitochondrial
5	4	40.3	14.203	19.44	P84103-2;P84103	Serine/arginine-rich splicing factor 3
6	6	11.7	83.641	19.425	Q9UHD2	Serine/threonine-protein kinase TBK1
9	9	25.7	53.974	19.421	Q9NUU7;Q9UMR2-2;Q9UMR2-4;Q9UMR2;Q9UMR2-3;Q9NUU7-2	ATP-dependent RNA helicase DDX19A;ATP-dependent RNA helicase DDX19B
8	8	29.7	41.044	19.415	P51398-2;P51398-3;P51398	28S ribosomal protein S29, mitochondrial
1	1	4.3	40.063	19.405	Q8IV48	3-5 exoribonuclease 1
7	7	11.1	105.32	19.399	P10253	Lysosomal alpha-glucosidase;76 kDa lysosomal alpha-glucosidase;70 kDa lysosomal alpha-glucosidase
14	14	24.6	82.842	19.377	P51003;P51003-2	Poly(A) polymerase alpha
5	5	14.6	52.764	19.332	Q96N66;Q96N66-3;Q96N66-2	Lysophospholipid acyltransferase 7
5	5	28.9	25.2	19.276	Q15056-2;Q15056	Eukaryotic translation initiation factor 4H
11	11	22	67.455	19.275	O00541-2;O00541	Pescadillo homolog
7	7	60.7	15.85	19.262	P09455;P09455-2;P09455-3	Retinol-binding protein 1
5	5	13.3	46.588	19.241	P11310;P11310-2	Medium-chain specific acyl-CoA dehydrogenase, mitochondrial
6	6	31.7	38.287	19.235	O14828;O14828-2	Secretory carrier-associated membrane protein 3
8	8	20.5	54.099	19.226	CON__Q3ZBS7;sp|Q3ZBS7|	
7	7	30	43.588	19.182	Q9BXW7-2;Q9BXW7	Cat eye syndrome critical region protein 5
1	1	3.8	56.476	19.106	Q9UBL3-2;Q9UBL3-3;Q9UBL3	Set1/Ash2 histone methyltransferase complex subunit ASH2
9	9	29	43.482	19.09	Q6NVY1;Q6NVY1-2	3-hydroxyisobutyryl-CoA hydrolase, mitochondrial
4	4	24.1	26.825	18.958	O15162-2;O15162	Phospholipid scramblase 1
10	10	10.8	141.15	18.958	Q92797;Q92797-2	Symplekin

20	1	82.2	41.251	18.957	sp|FA84-17-ACTB-Variante-2|;sp|P60712|;sp|P60710|;sp|P60709|;sp|FA84-17-ACTB-Native|;sp|FA05-19c|;sp|FA05-19b|;P60709;sp|FA84-17-ACTB-Variante-1|;sp|FA16-19|	Actin, cytoplasmic 1;Actin, cytoplasmic 1, N-terminally processed
9	9	14.2	100.29	18.94	O76024	Wolframin
5	5	17.3	39.304	18.901	P15151-3;P15151-2;P15151;P15151-4	Poliovirus receptor
8	8	59.3	20.652	18.885	Q9Y2B0	Protein canopy homolog 2
7	7	40.2	31.602	18.862	Q16625-4;Q16625;Q16625-2;Q16625-3;Q16625-5	Occludin
3	3	16.5	35.371	18.854	Q13426-3;Q13426-2;Q13426	DNA repair protein XRCC4
9	9	25.5	37.54	18.85	P37837	Transaldolase
11	11	18.6	87.743	18.84	Q13444-2;Q13444-12;Q13444-5;Q13444-4;Q13444-3;Q13444;Q13444-10;Q13444-9;Q13444-8;Q13444-7;Q13444-6;Q13444-11;Q13444-13	Disintegrin and metalloproteinase domain-containing protein 15
7	7	15	76.12	18.833	Q7Z417	Nuclear fragile X mental retardation-interacting protein 2
7	7	32.2	39.648	18.83	Q96EE3;Q96EE3-1	Nucleoporin SEH1
7	7	12	70.702	18.822	Q14739	Lamin-B receptor
3	3	18.4	22.761	18.801	Q15363	Transmembrane emp24 domain-containing protein 2
6	6	55.8	17.861	18.795	P68036;P68036-3;P68036-2;A0A1B0GUS4	Ubiquitin-conjugating enzyme E2 L3
12	12	13.6	111.13	18.793	P06756-3;P06756-2;P06756	Integrin alpha-V;Integrin alpha-V heavy chain;Integrin alpha-V light chain
6	6	6	169.01	18.782	Q9Y4B6;Q9Y4B6-2;Q9Y4B6-3	Protein VPRBP
7	7	8.6	130.44	18.769	Q9UDY2-6;Q9UDY2-3;Q9UDY2;Q9UDY2-7;Q9UDY2-5;Q9UDY2-4;Q9UDY2-2	Tight junction protein ZO-2
8	8	61.9	13.527	18.717	P62316;P62316-2	Small nuclear ribonucleoprotein Sm D2
5	5	20.1	44.552	18.698	P07339	Cathepsin D;Cathepsin D light chain;Cathepsin D heavy chain
8	8	29	37.432	18.661	O76003	Glutaredoxin-3
6	6	14.5	55.816	18.645	Q96SQ9;Q96SQ9-2	Cytochrome P450 2S1
4	4	14.1	57.948	18.584	P21589-2;P21589	5-nucleotidase
7	7	13.5	78.549	18.567	Q96EY7	Pentatricopeptide repeat domain-containing protein 3, mitochondrial
6	6	43.3	19.608	18.555	P07741;P07741-2	Adenine phosphoribosyltransferase
8	8	37.9	32.789	18.553	Q13868;Q13868-2;Q13868-3	Exosome complex component RRP4
6	6	58.3	12.784	18.541	P62888	60S ribosomal protein L30
9	9	22.4	57.548	18.541	P49257	Protein ERGIC-53
9	9	27.9	56.44	18.533	sp|P02676|;CON__P02676	
6	4	17	63.852	18.532	Q9NRR5	Ubiquilin-4
9	9	26.6	42.867	18.497	Q02127	Dihydroorotate dehydrogenase (quinone), mitochondrial
11	11	25.5	63.944	18.43	Q96HC4;Q96HC4-7;Q96HC4-4;Q96HC4-6	PDZ and LIM domain protein 5
9	9	44.4	30.601	18.398	P53701	Cytochrome c-type heme lyase
21	3	58.7	48.328	18.378	P68366-2;P68366	Tubulin alpha-4A chain
8	8	33.5	35.816	18.358	Q13011	Delta(3,5)-Delta(2,4)-dienoyl-CoA isomerase, mitochondrial
4	4	17.8	48.274	18.348	P46937-5;P46937-3;P46937-6;P46937-7;P46937-2;P46937-8;P46937;P46937-9;P46937-4	Transcriptional coactivator YAP1
11	11	13.2	119.39	18.315	O75146;O75146-2	Huntingtin-interacting protein 1-related protein
5	5	20.8	57.165	18.305	Q5ZPR3-4;Q5ZPR3;Q5ZPR3-3;Q5ZPR3-2	CD276 antigen
7	7	38.6	20.762	18.288	Q02543	60S ribosomal protein L18a
4	4	14.9	51.091	18.277	Q9NRH3;P23258	Tubulin gamma-2 chain;Tubulin gamma-1 chain
7	7	17.3	54.812	18.26	O75844	CAAX prenyl protease 1 homolog
5	5	10.5	69.531	18.254	Q96LD4;Q96LD4-2	Tripartite motif-containing protein 47
5	5	21.9	38.646	18.253	O15160-2;O15160	DNA-directed RNA polymerases I and III subunit RPAC1

6	6	29.8	27.647	18.241	P25788-2;P25788	Proteasome subunit alpha type-3
8	8	25.4	52.699	18.234	Q15642-4;Q15642-2;Q15642-3;Q15642;Q15642-5	Cdc42-interacting protein 4
7	7	42.8	22.837	18.187	P51151	Ras-related protein Rab-9A
3	3	6.9	78.159	18.179	O75027-3;O75027;O75027-2	ATP-binding cassette sub-family B member 7, mitochondrial
3	3	10.5	62.667	18.159	Q9BST9;Q9BST9-3;Q9BST9-2	Rhotekin
5	5	21.4	33.697	18.155	Q99439;Q99439-2	Calponin-2
5	5	46.3	12.587	18.135	P18859;P18859-2	ATP synthase-coupling factor 6, mitochondrial
4	4	18.7	40.623	18.106	O94992	Protein HEXIM1
9	9	24.6	52.221	18.1	P11172;P11172-3;P11172-2	Uridine 5-monophosphate synthase;Orotate phosphoribosyltransferase;Orotidine 5-phosphate decarboxylase
5	5	11.8	59.918	18.077	Q13492-4;Q13492-3;Q13492-2;Q13492-5;Q13492	Phosphatidylinositol-binding clathrin assembly protein
9	9	26.6	52.325	18.064	Q16630-3;Q16630;Q16630-2	Cleavage and polyadenylation specificity factor subunit 6
8	7	23.4	46.097	18.061	O60749-2;O60749	Sorting nexin-2
5	5	13.7	67.497	18.06	Q9Y2R4	Probable ATP-dependent RNA helicase DDX52
6	6	19.5	42.5	18.003	Q9UH62	Armadillo repeat-containing X-linked protein 3
6	1	34.1	27.389	17.971	Q8N2F6-4;Q8N2F6-2;Q8N2F6-6;Q8N2F6-5	Armadillo repeat-containing protein 10
10	10	24.9	48.039	17.966	Q9P2R7-2;Q9P2R7	Succinyl-CoA ligase [ADP-forming] subunit beta, mitochondrial
6	6	22.8	45.318	17.93	Q92665	28S ribosomal protein S31, mitochondrial
12	11	16.6	105.95	17.891	Q9NYF8-2;Q9NYF8;Q9NYF8-3;Q9NYF8-4	Bcl-2-associated transcription factor 1
5	5	16.6	42.006	17.885	Q9H074-3;Q9H074-2;Q9H074	Polyadenylate-binding protein-interacting protein 1
5	5	56.7	14.478	17.872	P61970	Nuclear transport factor 2
7	7	23.7	44.712	17.87	P25490	Transcriptional repressor protein YY1
12	12	11.3	143.35	17.867	Q8IY17-5;Q8IY17-2;Q8IY17-3;Q8IY17	Neuropathy target esterase
8	8	22.6	61.494	17.866	Q13573	SNW domain-containing protein 1
4	4	21.8	27.614	17.861	Q9H2J4	Phosducin-like protein 3
12	12	16.2	99.363	17.815	Q8NCN5	Pyruvate dehydrogenase phosphatase regulatory subunit, mitochondrial
8	8	27.1	33.331	17.796	Q9Y6C9	Mitochondrial carrier homolog 2
4	4	21.2	30.748	17.769	Q9BT09	Protein canopy homolog 3
11	11	9	191.58	17.754	Q7LBC6;Q7LBC6-2	Lysine-specific demethylase 3B
8	8	36.9	24.831	17.752	P62906	60S ribosomal protein L10a
5	5	18.5	31.625	17.737	Q96P16-3;Q96P16	Regulation of nuclear pre-mRNA domain-containing protein 1A
11	11	12.5	117.88	17.735	Q9NX02-5;Q9NX02-2;Q9NX02;Q9NX02-4;Q9NX02-3	NACHT, LRR and PYD domains-containing protein 2
6	6	25.1	38.006	17.712	P98172	Ephrin-B1
4	4	14.3	39.768	17.702	Q9H8Y8-2;Q9H8Y8;Q9H8Y8-3	Golgi reassembly-stacking protein 2
7	7	41.8	26.688	17.684	Q9UEU0;Q9UEU0-2	Vesicle transport through interaction with t-SNAREs homolog 1B
4	4	12	70.624	17.678	Q9Y450-4;Q9Y450	HBS1-like protein
7	7	20.4	56.5	17.652	P21281	V-type proton ATPase subunit B, brain isoform
4	4	27.5	19.266	17.64	Q8WW33	Gametocyte-specific factor 1
3	3	14.8	33.652	17.608	Q9BSV6	tRNA-splicing endonuclease subunit Sen34
6	6	7.7	118.32	17.56	P53992;P53992-2	Protein transport protein Sec24C
12	2	10.6	192.99	17.535	sp|E07350|;CON__ENSEMBL:ENSBTAP00000007350	
7	7	24.4	52.228	17.479	Q92917	G patch domain and KOW motifs-containing protein
7	7	15.8	71.689	17.478	O60216	Double-strand-break repair protein rad21 homolog
6	6	17.8	51.871	17.463	Q96JJ7	Protein disulfide-isomerase TMX3
6	6	36.1	20.006	17.412	P30626-3;P30626-2;P30626	Sorcin
8	8	10.9	123.51	17.407	Q6PKG0;Q6PKG0-3	La-related protein 1
9	9	10.6	120.54	17.398	Q9UPN9-2;Q9UPN9	E3 ubiquitin-protein ligase TRIM33
8	8	23.8	50.817	17.364	Q9UN86-2;Q9UN86	Ras GTPase-activating protein-binding protein 2

8	8	8.4	144.73	17.355	O15067	Phosphoribosylformylglycinamidine synthase
6	6	13.6	72.2	17.332	Q9Y3Z3;Q9Y3Z3-4;Q9Y3Z3-3	Deoxynucleoside triphosphate triphosphohydrolase SAMHD1
6	6	28.2	33.392	17.326	O95861;O95861-2;O95861-3;O95861-4	3(2),5-bisphosphate nucleotidase 1
3	3	36.2	14.649	17.28	Q9Y605	MORF4 family-associated protein 1
6	6	20.1	41.779	17.273	Q969G3-2;Q969G3;Q969G3-6;Q969G3-5;Q969G3-3;Q969G3-4	SWI/SNF-related matrix-associated actin-dependent regulator of chromatin subfamily E member 1
4	4	29.3	25.569	17.26	P68402;P68402-3;P68402-2;P68402-4	Platelet-activating factor acetylhydrolase IB subunit beta
4	4	33.1	18.648	17.249	Q14011;Q14011-2	Cold-inducible RNA-binding protein
8	8	13.1	102.45	17.235	Q15269	Periodic tryptophan protein 2 homolog
5	5	25.2	35.386	17.186	P25445-6;P25445	Tumor necrosis factor receptor superfamily member 6
4	4	2.3	243.07	17.184	Q9H2D6-3;Q9H2D6-2;Q9H2D6;Q9H2D6-5;Q9H2D6-7	TRIO and F-actin-binding protein
9	2	35.7	21.364	17.183	P16403;P16402	Histone H1.2;Histone H1.3
7	7	42.2	26.182	17.17	P15374	Ubiquitin carboxyl-terminal hydrolase isozyme L3
11	9	17.1	108.27	17.158	P54760;P54760-2;P54760-4	Ephrin type-B receptor 4
6	6	26	36.978	17.152	Q03405;Q03405-3;Q03405-2	Urokinase plasminogen activator surface receptor
9	9	9.5	131.14	17.151	Q16706	Alpha-mannosidase 2
11	11	28	44.875	17.136	Q9BTV4	Transmembrane protein 43
6	6	10.8	80.64	17.132	Q99797	Mitochondrial intermediate peptidase
4	4	20.9	35.348	17.088	Q13155;Q13155-2	Aminoacyl tRNA synthase complex-interacting multifunctional protein 2
9	9	41	30.364	17.082	Q9UKM9-2;Q9UKM9	RNA-binding protein Raly
8	8	19.3	65.38	17.019	Q53GS9;Q53GS9-2;Q53GS9-3	U4/U6.U5 tri-snRNP-associated protein 2
6	6	14	40.251	17.015	Q8NFJ5	Retinoic acid-induced protein 3
4	4	30.3	23.598	16.99	Q9HCN8	Stromal cell-derived factor 2-like protein 1
7	7	22.7	40.029	16.981	P78310;P78310-7;P78310-2;P78310-6;P78310-5	Coxsackievirus and adenovirus receptor
7	5	31.8	32.668	16.976	P46926;P46926-2	Glucosamine-6-phosphate isomerase 1
5	5	42.5	21.831	16.962	Q9Y6M9	NADH dehydrogenase [ubiquinone] 1 beta subcomplex subunit 9
12	12	10.8	185.54	16.95	Q9ULH0-2;Q9ULH0-4;Q9ULH0	Kinase D-interacting substrate of 220 kDa
6	6	7.3	138.04	16.95	Q9H7F0;Q9H7F0-2	Probable cation-transporting ATPase 13A3
7	7	48.3	16.06	16.921	P39019	40S ribosomal protein S19
6	6	25.4	36.724	16.906	Q9BQA1;Q9BQA1-2	Methylosome protein 50
6	6	40	15.55	16.903	P08708	40S ribosomal protein S17
10	10	24.1	56.974	16.899	Q8NC56;Q8NC56-2	LEM domain-containing protein 2
7	6	11.9	94.198	16.899	Q8IWT6	Volume-regulated anion channel subunit LRRC8A
6	6	27.7	35.368	16.896	Q9BXY0	Protein MAK16 homolog
6	6	21.2	27.626	16.893	Q9NX40;Q9NX40-3;Q9NX40-2;Q9NX40-4	OCIA domain-containing protein 1
7	7	39.4	26.489	16.892	P20618	Proteasome subunit beta type-1
5	5	23.3	25.565	16.885	P00403	Cytochrome c oxidase subunit 2
6	6	8.8	122.11	16.861	Q9Y4F1-2;Q9Y4F1	FERM, RhoGEF and pleckstrin domain-containing protein 1
7	7	19.4	48.506	16.86	Q9UHG3-2;Q9UHG3	Prenylcysteine oxidase 1
5	5	24.7	23.432	16.859	P50914	60S ribosomal protein L14
5	5	38.2	21.232	16.858	Q9NR28-2;Q9NR28;Q9NR28-3	Diablo homolog, mitochondrial
10	10	12	101.51	16.831	sp|Q3T052|;CON__Q3T052	
4	4	34.2	18.007	16.815	P63279	SUMO-conjugating enzyme UBC9
7	6	21.3	50.989	16.802	P05091-2;P05091	Aldehyde dehydrogenase, mitochondrial
7	7	29.3	35.892	16.782	Q13630	GDP-L-fucose synthase
9	9	20.2	63.523	16.777	O95470	Sphingosine-1-phosphate lyase 1
13	11	26.5	73.91	16.77	Q14195-2;Q14195	Dihydropyrimidinase-related protein 3
11	11	26.6	56.625	16.758	Q9UQB8-3;Q9UQB8-5;Q9UQB8-4;Q9UQB8-6;Q9UQB8-2;Q9UQB8	Brain-specific angiogenesis inhibitor 1-associated protein 2
6	6	15.9	54.29	16.746	Q14108;Q14108-2	Lysosome membrane protein 2

11	11	5.8	295.73	16.717	Q9Y520-3;Q9Y520-6;Q9Y520-4;Q9Y520-5;Q9Y520;Q9Y520-7;Q9Y520-2	Protein PRRC2C
10	10	58.2	16.445	16.706	P62249	40S ribosomal protein S16
6	6	28.2	35.102	16.667	O00764;O00764-3;O00764-2	Pyridoxal kinase
10	10	18.9	77.91	16.663	O94901-5;O94901-8;O94901;O94901-9;O94901-6	SUN domain-containing protein 1
6	6	33.8	26.372	16.629	P18621-3;P18621;P18621-2	60S ribosomal protein L17
7	7	28.9	36.908	16.626	Q9BYD6	39S ribosomal protein L1, mitochondrial
8	8	16.5	82.785	16.625	P49711;P49711-2	Transcriptional repressor CTCF
10	10	16.9	87.156	16.61	Q9Y263	Phospholipase A-2-activating protein
6	6	42	19.043	16.603	Q04760-2;Q04760	Lactoylglutathione lyase
10	10	33.2	44.596	16.6	Q96DV4	39S ribosomal protein L38, mitochondrial
5	5	5.9	142.17	16.598	O15118;O15118-2	Niemann-Pick C1 protein
2	2	39.5	9.2733	16.596	P52926-3;P52926-6;P52926-4;P52926-5;P52926-2;P52926	High mobility group protein HMGI-C
6	6	60.2	9.1113	16.541	P63220	40S ribosomal protein S21
6	6	22.4	38.498	16.528	P40121;P40121-2	Macrophage-capping protein
5	5	27.4	30.18	16.493	Q16740	ATP-dependent Clp protease proteolytic subunit, mitochondrial
5	5	6.4	140.5	16.448	Q92620	Pre-mRNA-splicing factor ATP-dependent RNA helicase PRP16
4	4	21.9	33.178	16.437	P25325;P25325-2	3-mercaptopyruvate sulfurtransferase
30	1	50.8	95.807	16.372	Q12906-7;Q12906-4;Q12906-6	Interleukin enhancer-binding factor 3
8	8	35.7	29.555	16.359	P25786;P25786-2	Proteasome subunit alpha type-1
5	5	15.7	43.772	16.357	Q9Y282-3;Q9Y282;Q9Y282-2	Endoplasmic reticulum-Golgi intermediate compartment protein 3
6	6	18.2	62.259	16.35	Q8WVM8-2;Q8WVM8-3;Q8WVM8	Sec1 family domain-containing protein 1
6	6	16	48.55	16.332	Q15645	Pachytene checkpoint protein 2 homolog
10	10	15.2	89.257	16.331	Q8N3E9	1-phosphatidylinositol 4,5-bisphosphate phosphodiesterase delta-3
6	6	15.9	59.356	16.306	Q8WVX9	Fatty acyl-CoA reductase 1
4	4	12.4	54.854	16.263	Q96GA3	Protein LTV1 homolog
6	6	18.7	46.75	16.249	Q13685	Angio-associated migratory cell protein
6	3	30.8	34.834	16.237	P60891;P60891-2	Ribose-phosphate pyrophosphokinase 1
6	6	28.9	31.821	16.227	Q15024	Exosome complex component RRP42
5	5	6.6	126.71	16.224	Q8NB49-2;Q8NB49-3;Q8NB49-4;Q8NB49	Phospholipid-transporting ATPase IG
5	5	39.9	17.316	16.18	Q9NX14;Q9NX14-2	NADH dehydrogenase [ubiquinone] 1 beta subcomplex subunit 11, mitochondrial
10	10	21	81.742	16.175	P48960-2;P48960-3;P48960	CD97 antigen;CD97 antigen subunit alpha;CD97 antigen subunit beta
9	9	27.3	49.389	16.162	Q96CW1-2;Q96CW1	AP-2 complex subunit mu
8	8	26.7	38.914	16.162	P48729;P48729-2;P48729-3	Casein kinase I isoform alpha
9	9	24.8	45.811	16.149	Q96ER9;Q96ER9-2	Coiled-coil domain-containing protein 51
15	14	2.7	860.65	16.141	Q03001;Q03001-8;Q03001-14	Dystonin
9	9	21.5	54.75	16.135	Q15392-2;Q15392	Delta(24)-sterol reductase
7	7	16.4	60.207	16.125	Q7L5N7	Lysophosphatidylcholine acyltransferase 2
6	6	38.2	26.711	16.097	O75940	Survival of motor neuron-related-splicing factor 30
8	7	13.2	98.675	16.079	Q96J02-2;Q96J02;Q96J02-3	E3 ubiquitin-protein ligase Itchy homolog
8	7	34.7	33.637	16.063	Q96C36	Pyrroline-5-carboxylate reductase 2
8	8	14.4	67.887	16.058	Q9HD45	Transmembrane 9 superfamily member 3
3	3	10.5	59.862	16.015	Q9Y5Y0	Feline leukemia virus subgroup C receptor-related protein 1
3	3	14.5	31.528	16.012	Q96IZ7-2;Q96IZ7	Serine/Arginine-related protein 53
7	7	16.9	67.119	16.008	Q9UHB6-2;Q9UHB6-5;Q9UHB6;Q9UHB6-4;Q9UHB6-3	LIM domain and actin-binding protein 1
5	5	25.7	32.485	16.004	Q13907-2;Q13907	Isopentenyl-diphosphate Delta-isomerase 1
6	6	11.1	88.118	16.002	Q6PJG6;Q6PJG6-3	BRCA1-associated ATM activator 1
4	4	41.5	12.517	15.99	O43920	NADH dehydrogenase [ubiquinone] iron-sulfur protein 5

5	5	14.2	41.522	15.98	O00767	Acyl-CoA desaturase
6	6	12.6	63.429	15.959	Q8NBN3;Q8NBN3-3	Transmembrane protein 87A
10	10	18.9	91.809	15.956	Q2NL82	Pre-rRNA-processing protein TSR1 homolog
5	5	19.8	42.646	15.943	Q29980;Q29980-2	MHC class I polypeptide-related sequence B
6	6	20	47.998	15.931	Q8N806	Putative E3 ubiquitin-protein ligase UBR7
8	8	13.4	82.658	15.925	Q9H1B7	Interferon regulatory factor 2-binding protein-like
9	8	20.9	66.54	15.915	Q8IXI2-4;Q8IXI2;Q8IXI2-5;Q8IXI2-2;Q8IXI2-7;Q8IXI2-3;Q8IXI2-6	Mitochondrial Rho GTPase 1
4	4	57.1	11.471	15.91	Q99584	Protein S100-A13
8	8	53.5	18.681	15.909	Q9H773	dCTP pyrophosphatase 1
11	11	16	98.594	15.895	Q8TDD1;Q8TDD1-2	ATP-dependent RNA helicase DDX54
7	7	21.1	57.13	15.885	O00515	Ladinin-1
9	9	19	66.819	15.865	Q6NUM9;Q6NUM9-2	All-trans-retinol 13,14-reductase
8	8	52.8	16.698	15.859	Q9P0J0;Q9P0J0-2	NADH dehydrogenase [ubiquinone] 1 alpha subcomplex subunit 13
7	7	36	33.777	15.854	P46109	Crk-like protein
6	6	29.6	33.429	15.808	Q16762	Thiosulfate sulfurtransferase
7	7	17.1	70.352	15.803	Q9NVH0;Q9NVH0-2	Exonuclease 3-5 domain-containing protein 2
4	4	33.6	14.173	15.787	P55769	NHP2-like protein 1;NHP2-like protein 1, N-terminally processed
5	5	15.4	47.205	15.765	Q9H6R6-2;Q9H6R6	Palmitoyltransferase ZDHHC6
5	5	32.3	31.307	15.757	Q9H9H4	Vacuolar protein sorting-associated protein 37B
5	5	22.7	34.188	15.755	Q9Y6I9	Testis-expressed sequence 264 protein
4	4	20.1	41.211	15.725	Q8IXU6;Q8IXU6-3;Q8IXU6-2	Solute carrier family 35 member F2
9	9	9.1	156.33	15.724	P06213;P06213-2	Insulin receptor;Insulin receptor subunit alpha;Insulin receptor subunit beta
5	4	6.4	104.67	15.723	Q16512-2;Q16512;Q16512-3	Serine/threonine-protein kinase N1
4	4	10.5	49.481	15.714	Q8NDZ4;Q8NDZ4-2	Deleted in autism protein 1
4	4	7.5	76.107	15.69	O43823	A-kinase anchor protein 8
9	1	28.5	40.338	15.638	sp	
10	10	45.6	25.206	15.623	P62993;P62993-2	Growth factor receptor-bound protein 2
1	1	11.5	21.711	15.608	Q12962	Transcription initiation factor TFIID subunit 10
5	5	32	25.694	15.585	Q9Y508;Q9Y508-2	E3 ubiquitin-protein ligase RNF114
8	1	25.2	47.46	15.585	O96019	Actin-like protein 6A
11	11	29.5	49.222	15.582	P55010	Eukaryotic translation initiation factor 5
6	6	23.9	41.219	15.577	Q8IWS0-3;Q8IWS0;Q8IWS0-5;Q8IWS0-4;Q8IWS0-2	PHD finger protein 6
6	6	30.7	27.248	15.576	Q6IQ22	Ras-related protein Rab-12
5	5	14.7	49.692	15.562	O43670-2;O43670;O43670-4;O43670-3	BUB3-interacting and GLEBS motif-containing protein ZNF207
8	8	35.4	27.873	15.558	O95571	Persulfide dioxygenase ETHE1, mitochondrial
7	7	32.1	25.177	15.549	Q00688	Peptidyl-prolyl cis-trans isomerase FKBP3
11	11	19.6	75.579	15.543	O60353-2;O60353	Frizzled-6
10	10	9.8	197.6	15.488	Q14676-2;Q14676;Q14676-3;Q14676-4	Mediator of DNA damage checkpoint protein 1
8	7	20.1	56.578	15.468	Q9Y6G9	Cytoplasmic dynein 1 light intermediate chain 1
17	2	46.1	49.906	15.459	Q13885;Q9BVA1	Tubulin beta-2A chain;Tubulin beta-2B chain
9	9	9.7	145.17	15.414	Q96ST3	Paired amphipathic helix protein Sin3a
10	10	28.2	47.137	15.394	Q53H12	Acylglycerol kinase, mitochondrial
5	5	20.8	42.456	15.388	Q6DKK2	Tetratricopeptide repeat protein 19, mitochondrial
16	3	45.3	32.866	15.377	P12236	ADP/ATP translocase 3;ADP/ATP translocase 3, N-terminally processed
5	5	32.7	16.545	15.342	P31431-2;P31431	Syndecan-4
7	7	26.7	39.42	15.331	Q9BQP7	Mitochondrial genome maintenance exonuclease 1
4	4	20	33.3	15.322	Q5T653	39S ribosomal protein L2, mitochondrial
6	6	22.3	44.259	15.278	Q9NYB0	Telomeric repeat-binding factor 2-interacting protein 1
11	11	15.8	92.755	15.277	Q99959-2;Q99959	Plakophilin-2

10	9	31.6	44.049	15.272	Q14344;Q14344-2	Guanine nucleotide-binding protein subunit alpha-13
10	10	18.3	74.89	15.268	Q15061	WD repeat-containing protein 43
10	10	12.5	116.92	15.265	O15294;O15294-3;O15294-2	UDP-N-acetylglucosamine--peptide N-acetylglucosaminyltransferase 110 kDa subunit
7	7	7.8	133.5	15.254	Q6PD62	RNA polymerase-associated protein CTR9 homolog
4	4	57	10.859	15.246	Q9BXV9	Uncharacterized protein C14orf142
11	11	16	109.3	15.237	Q9Y4E8-2;Q9Y4E8-3;Q9Y4E8	Ubiquitin carboxyl-terminal hydrolase 15
8	8	10.6	112.29	15.23	Q14CX7;Q14CX7-2	N-alpha-acetyltransferase 25, NatB auxiliary subunit
8	8	10.8	114.25	15.209	Q05397-7;Q05397;Q05397-5;Q05397-2;Q05397-6	Focal adhesion kinase 1
10	10	30.6	32.728	15.194	Q02878	60S ribosomal protein L6
2	2	22	23.489	15.129	O95197-6;O95197-3;O95197-5;O95197-4;O95197-7;O95197-2;O95197	Reticulon-3
5	5	12.8	52.839	15.123	P56182	Ribosomal RNA processing protein 1 homolog A
9	9	14.4	97.685	15.087	Q9H4L5-2;Q9H4L5;Q9H4L5-4;Q9H4L5-3;Q9H4L5-6;Q9H4L5-5	Oxysterol-binding protein-related protein 3
5	4	35.6	20.589	15.08	Q13595-4;Q13595-3;Q13595;Q13595-2	Transformer-2 protein homolog alpha
6	6	15.7	68.297	15.075	Q13409-6;Q13409-3;Q13409-7;Q13409-2;Q13409-5;Q13409	Cytoplasmic dynein 1 intermediate chain 2
7	6	35	27.228	15.072	O95292	Vesicle-associated membrane protein-associated protein B/C
8	7	25.6	44.179	15.069	P63092-3;P63092-2;P63092;P63092-4;Q5JWF2-2;Q5JWF2	Guanine nucleotide-binding protein G(s) subunit alpha isoforms short;Guanine nucleotide-binding protein G(s) subunit alpha isoforms XLas
3	3	3.9	119.75	15.059	O95486	Protein transport protein Sec24A
6	6	12.1	71.639	14.961	Q9ULX6;Q9ULX6-2	A-kinase anchor protein 8-like
9	9	18.7	67.011	14.958	sp|P02672|;CON__P02672	
6	6	24.7	40.968	14.923	P78406	mRNA export factor
5	5	6.7	140.37	14.911	sp|Q28085|;CON__Q28085	
4	4	23.7	27.391	14.895	P19404	NADH dehydrogenase [ubiquinone] flavoprotein 2, mitochondrial
7	7	19.1	52.562	14.879	Q13867	Bleomycin hydrolase
6	6	52.8	15.945	14.874	Q14019	Coactosin-like protein
10	8	14	113.68	14.867	O75150;O75150-4;O75150-3	E3 ubiquitin-protein ligase BRE1B
8	8	20.1	57.543	14.841	Q2TAY7;Q2TAY7-2	WD40 repeat-containing protein SMU1;WD40 repeat-containing protein SMU1, N-terminally processed
5	5	15.2	57.224	14.838	Q8WUA2	Peptidyl-prolyl cis-trans isomerase-like 4
6	6	35.3	29.572	14.832	Q9NQT5;Q9NQT5-2	Exosome complex component RRP40
6	6	18.9	48.713	14.816	Q12765-2;Q12765;Q12765-3	Secernin-1
6	6	33.3	12.254	14.814	Q9Y3U8	60S ribosomal protein L36
9	8	12.7	109.82	14.813	P51784	Ubiquitin carboxyl-terminal hydrolase 11
10	5	60.6	20.529	14.809	P84085	ADP-ribosylation factor 5
6	6	34.9	28.114	14.763	O60499;O60499-2	Syntaxin-10
3	3	29.8	17.745	14.763	Q6IAA8	Ragulator complex protein LAMTOR1
6	6	9.4	94.131	14.759	Q9ULF5;Q9ULF5-2	Zinc transporter ZIP10
7	7	18.9	63.132	14.746	Q9NY61	Protein AATF
9	9	13.3	109.96	14.746	O43592	Exportin-T
6	6	20.2	41.943	14.73	Q9H7Z7	Prostaglandin E synthase 2;Prostaglandin E synthase 2 truncated form
7	7	14.7	69.922	14.729	Q9NW13-2;Q9NW13	RNA-binding protein 28
4	4	11.5	42.906	14.714	Q9H0C8	Integrin-linked kinase-associated serine/threonine phosphatase 2C
5	5	16.6	58.281	14.698	Q9C0B1	Alpha-ketoglutarate-dependent dioxygenase FTO
4	4	20.5	27.567	14.686	Q9GZP9	Derlin-2
19	2	55.4	49.585	14.643	P04350	Tubulin beta-4A chain
9	9	14.8	91.88	14.612	Q12929	Epidermal growth factor receptor kinase substrate 8
10	10	29.8	41.9	14.609	Q15738	Sterol-4-alpha-carboxylate 3-dehydrogenase, decarboxylating
8	8	55	23.239	14.594	P43487-2;P43487	Ran-specific GTPase-activating protein

8	8	20	53.801	14.591	P49189;P49189-3;P49189-2	4-trimethylaminobutyraldehyde dehydrogenase
4	4	2.4	294.36	14.584	Q9UPU5	Ubiquitin carboxyl-terminal hydrolase 24
7	7	19.3	57.673	14.56	O60832;O60832-2	H/ACA ribonucleoprotein complex subunit 4
4	4	17.2	37.413	14.548	Q15599;Q15599-3;Q15599-2	Na(+)/H(+) exchange regulatory cofactor NHE-RF2
5	5	17	53.198	14.538	Q9Y2Z4	Tyrosine--tRNA ligase, mitochondrial
5	5	11.2	54.083	14.536	P11166	Solute carrier family 2, facilitated glucose transporter member 1
10	10	13.8	113.45	14.526	Q6P1M3;Q6P1M3-2	Lethal(2) giant larvae protein homolog 2
3	3	28.5	16.156	14.515	P09132	Signal recognition particle 19 kDa protein
6	6	27.4	35.196	14.509	Q96DH6;Q96DH6-2;Q96DH6-3	RNA-binding protein Musashi homolog 2
6	5	10.8	72.383	14.487	Q96SB4-4;Q96SB4;Q96SB4-3	SRSF protein kinase 1
3	3	12.2	36.163	14.477	Q7L5N1	COP9 signalosome complex subunit 6
5	5	43.4	13.696	14.472	P10606	Cytochrome c oxidase subunit 5B, mitochondrial
6	6	34.1	29.395	14.471	Q9Y676	28S ribosomal protein S18b, mitochondrial
8	8	32.4	37.84	14.455	O76071	Probable cytosolic iron-sulfur protein assembly protein CIAO1
5	5	28.2	25.898	14.452	P25787	Proteasome subunit alpha type-2
5	5	5	147.81	14.437	Q9HAU5	Regulator of nonsense transcripts 2
5	5	46.7	11.737	14.412	P10599;P10599-2	Thioredoxin
4	4	44.8	13.832	14.4	Q9NRX4;Q9NRX4-2	14 kDa phosphohistidine phosphatase
5	3	52.1	15.184	14.399	sp|P01966|;CON__P01966	
7	7	24.2	40.75	14.398	O95299;O95299-2	NADH dehydrogenase [ubiquinone] 1 alpha subcomplex subunit 10, mitochondrial
2	2	17.4	19.738	14.366	P41208	Centrin-2
8	8	9.6	128.98	14.354	Q8WUM0	Nuclear pore complex protein Nup133
5	5	16	51.402	14.338	Q9NV06	DDB1- and CUL4-associated factor 13
5	5	15.4	42.127	14.323	Q8TB61-3;Q8TB61-2;Q8TB61;Q8TB61-5;Q8TB61-4	Adenosine 3-phospho 5-phosphosulfate transporter 1
9	9	18.2	94.769	14.31	Q9Y5Y6	Suppressor of tumorigenicity 14 protein
8	8	25.2	44.839	14.304	Q8WVQ1;Q8WVQ1-3;Q8WVQ1-2	Soluble calcium-activated nucleotidase 1
6	6	23.7	50.287	14.303	Q15654	Thyroid receptor-interacting protein 6
8	8	27.1	46.898	14.302	Q9NY27;Q9NY27-2;Q9NY27-3	Serine/threonine-protein phosphatase 4 regulatory subunit 2
6	6	13	65.523	14.29	Q5T2T1;Q5T2T1-2	MAGUK p55 subfamily member 7
7	7	23.6	43.083	14.289	Q70UQ0-4	Inhibitor of nuclear factor kappa-B kinase-interacting protein
5	5	10.2	79.579	14.288	Q9UKM7	Endoplasmic reticulum mannosyl-oligosaccharide 1,2-alpha-mannosidase
9	9	38.7	22.591	14.272	P46781	40S ribosomal protein S9
7	7	32.7	38.725	14.258	Q92890-1;Q92890;Q92890-3	Ubiquitin fusion degradation protein 1 homolog
8	8	15.3	71.996	14.258	Q99543;Q99543-2	DnaJ homolog subfamily C member 2;DnaJ homolog subfamily C member 2, N-terminally processed
5	5	16.1	55.594	14.251	Q9BZK7	F-box-like/WD repeat-containing protein TBL1XR1
10	10	11.3	123.56	14.216	Q9H2P0	Activity-dependent neuroprotector homeobox protein
2	2	13.1	21.125	14.203	Q8N5M9	Protein jagunal homolog 1
17	11	24.3	104.09	14.202	O94973-2;O94973;O94973-3	AP-2 complex subunit alpha-2
2	2	56.8	8.0019	14.183	Q96B49	Mitochondrial import receptor subunit TOM6 homolog
5	5	20.6	40.307	14.18	Q9NR45	Sialic acid synthase
6	6	28.3	34.291	14.175	Q8IWE4	DCN1-like protein 3
7	5	3.9	269.45	14.175	Q8IWZ3;Q8IWZ3-4;Q8IWZ3-6	Ankyrin repeat and KH domain-containing protein 1
7	7	32.1	34.352	14.156	Q12904;Q12904-2	Aminoacyl tRNA synthase complex-interacting multifunctional protein 1;Endothelial monocyte-activating polypeptide 2
5	5	20.6	37.32	14.151	Q6YN16-2;Q6YN16	Hydroxysteroid dehydrogenase-like protein 2
7	7	20.5	57.46	14.094	Q9H0R6;Q9H0R6-2	Glutamyl-tRNA(Gln) amidotransferase subunit A, mitochondrial
8	8	28	34.333	14.089	O15144	Actin-related protein 2/3 complex subunit 2
4	4	34.6	14.395	14.081	P53999	Activated RNA polymerase II transcriptional coactivator p15
7	7	18	58.32	14.071	O43172-2;O43172	U4/U6 small nuclear ribonucleoprotein Prp4
5	5	20	32.935	14.064	Q8NBQ5	Estradiol 17-beta-dehydrogenase 11

6	6	14.9	49.601	14.036	Q13895	Bystin
6	3	30	22.774	14.027	P51153	Ras-related protein Rab-13
7	7	13.2	76.855	14.006	P01130-3;P01130-4;P01130-5;P01130;P01130-2;P01130-6	Low-density lipoprotein receptor
5	5	10.2	58.355	14.001	Q96KA5-2;Q96KA5	Cleft lip and palate transmembrane protein 1-like protein
2	2	16.2	16.786	13.992	P83876	Thioredoxin-like protein 4A
5	5	18.9	39.221	13.991	P78318	Immunoglobulin-binding protein 1
6	6	12.3	62.317	13.969	Q9ULP9-2;Q9ULP9	TBC1 domain family member 24
9	9	4	318.38	13.968	O75691	Small subunit processome component 20 homolog
4	4	14.6	46.701	13.965	Q96M27;Q96M27-3;Q96M27-2;Q96M27-5;Q96M27-4	Protein PRRC1
5	5	16	43.835	13.953	Q7KZN9-2;Q7KZN9	Cytochrome c oxidase assembly protein COX15 homolog
6	6	18.8	51.852	13.95	Q8TBC4;Q8TBC4-2	NEDD8-activating enzyme E1 catalytic subunit
4	4	36.4	16.273	13.947	P62263	40S ribosomal protein S14
2	2	27.1	18.541	13.936	Q71RG4-3;Q71RG4-2;Q71RG4	Transmembrane and ubiquitin-like domain-containing protein 2
7	7	28	27.991	13.884	P51572;P51572-2	B-cell receptor-associated protein 31
6	6	9.9	97.44	13.835	Q14118	Dystroglycan;Alpha-dystroglycan;Beta-dystroglycan
7	6	13.8	70.86	13.787	Q8IUH4;Q8IUH4-2;Q8IUH4-3	Palmitoyltransferase ZDHHC13
8	8	28.2	33.582	13.785	Q6FI81;Q6FI81-3	Anamorsin
4	4	15.2	37.646	13.776	O95400	CD2 antigen cytoplasmic tail-binding protein 2
1	1	7.6	21.08	13.775	Q9UNL2;Q9UNL2-2	Translocon-associated protein subunit gamma
2	2	5.8	48.705	13.771	Q53EU6	Glycerol-3-phosphate acyltransferase 3
5	5	38.4	15.693	13.769	P29373	Cellular retinoic acid-binding protein 2
7	7	21.4	38.681	13.759	Q9NX62	Inositol monophosphatase 3
8	8	11.9	101.73	13.758	P18858;P18858-3;P18858-2	DNA ligase 1
7	7	14.2	83.654	13.721	Q13823	Nucleolar GTP-binding protein 2
5	5	15.5	38.036	13.693	Q9H0U3	Magnesium transporter protein 1
7	7	34.4	34.14	13.681	Q99988	Growth/differentiation factor 15
9	9	37.9	30.188	13.667	P29218;P29218-3	Inositol monophosphatase 1
4	4	23.5	35.668	13.657	Q9UBQ7	Glyoxylate reductase/hydroxypyruvate reductase
4	4	9.7	68.07	13.641	O15213	WD repeat-containing protein 46
7	7	10.6	104.74	13.631	P43246;P43246-2	DNA mismatch repair protein Msh2
5	5	32.2	26.906	13.599	Q9UNK0	Syntaxin-8
3	3	16.6	32.363	13.597	Q9NWS0;Q9NWS0-3	PIH1 domain-containing protein 1
8	8	20.8	47.094	13.572	Q9C0E8-2;Q9C0E8;Q9C0E8-4;Q9C0E8-3	Protein lunapark
7	7	44.2	21.878	13.562	P62166;P62166-2	Neuronal calcium sensor 1
3	3	14	32.662	13.553	Q9BW60;Q9BW60-2	Elongation of very long chain fatty acids protein 1
7	7	35.2	33.112	13.546	Q99598	Translin-associated protein X
8	8	18.2	54.022	13.534	Q9BTU6	Phosphatidylinositol 4-kinase type 2-alpha
14	4	57.2	37.186	13.523	P62140	Serine/threonine-protein phosphatase PP1-beta catalytic subunit
7	7	16.7	63.111	13.51	P07686	Beta-hexosaminidase subunit beta;Beta-hexosaminidase subunit beta chain B;Beta-hexosaminidase subunit beta chain A
7	7	39.4	22.417	13.49	O15498;O15498-2	Synaptobrevin homolog YKT6
6	6	23.2	34.69	13.489	Q8NFV4;Q8NFV4-4;Q8NFV4-6	Alpha/beta hydrolase domain-containing protein 11
3	3	52.1	10.403	13.478	O95777	U6 snRNA-associated Sm-like protein LSm8
8	8	17.4	56.535	13.476	P52306-6;P52306-2;P52306-3;P52306-4;P52306;P52306-5	Rap1 GTPase-GDP dissociation stimulator 1
7	7	57	17.887	13.464	P05161	Ubiquitin-like protein ISG15
2	2	23.3	26.318	13.46	Q14353;Q14353-2	Guanidinoacetate N-methyltransferase
6	6	22.7	37.636	13.445	O00170	AH receptor-interacting protein
10	10	7.6	182.29	13.444	Q5SRE5-2;Q5SRE5	Nucleoporin NUP188 homolog

6	6	14.8	71.951	13.439	Q9C0B5-2;Q9C0B5	Palmitoyltransferase ZDHHC5
6	6	19.7	37.001	13.406	Q3SXM5;Q3SXM5-2	Inactive hydroxysteroid dehydrogenase-like protein 1
7	7	46.8	25.384	13.371	Q8TAE8	Growth arrest and DNA damage-inducible proteins-interacting protein 1
8	8	23.8	53.248	13.361	Q9ULV4;Q9ULV4-2;Q9ULV4-3	Coronin-1C
3	3	6.6	67.664	13.36	Q6NXG1-2;Q6NXG1-5;Q6NXG1-4;Q6NXG1-3;Q6NXG1	Epithelial splicing regulatory protein 1
5	5	17.9	31.791	13.357	Q9H3N1	Thioredoxin-related transmembrane protein 1
5	5	14.5	50.219	13.347	Q99808;Q99808-2	Equilibrative nucleoside transporter 1
5	3	21.1	37.655	13.342	Q99496;Q99496-2	E3 ubiquitin-protein ligase RING2
2	2	41.2	10.998	13.304	O60220	Mitochondrial import inner membrane translocase subunit Tim8 A
8	6	10.7	106.19	13.282	P56545-2;P56545;P56545-3	C-terminal-binding protein 2
3	3	30.8	15.585	13.27	P21741;P21741-2	Midkine
10	10	12.7	93.673	13.249	Q8TCJ2	Dolichyl-diphosphooligosaccharide--protein glycosyltransferase subunit STT3B
6	6	11.8	61.206	13.241	Q5SSJ5;Q5SSJ5-3;Q5SSJ5-2	Heterochromatin protein 1-binding protein 3
4	4	10.1	72.609	13.227	Q14435	Polypeptide N-acetylgalactosaminyltransferase 3
4	4	18.5	36.864	13.225	Q9HBH5	Retinol dehydrogenase 14
6	6	31.4	25.497	13.22	Q9Y2Q3;Q9Y2Q3-3;Q9Y2Q3-2;Q9Y2Q3-4	Glutathione S-transferase kappa 1
7	7	12.6	80.651	13.196	Q9BYT8	Neurolysin, mitochondrial
4	4	50	10.275	13.178	P61513	60S ribosomal protein L37a
3	3	13.5	26.005	13.172	Q9Y3A6	Transmembrane emp24 domain-containing protein 5
5	4	24.8	35.08	13.17	P60510	Serine/threonine-protein phosphatase 4 catalytic subunit
4	4	10.1	57.218	13.163	Q643R3	Lysophospholipid acyltransferase LPCAT4
3	3	32.1	13.81	13.13	Q9Y237;Q9Y237-2	Peptidyl-prolyl cis-trans isomerase NIMA-interacting 4
5	5	19.7	33.84	13.118	Q12846-2;Q12846	Syntaxin-4
9	9	21.1	52.948	13.109	P07099	Epoxide hydrolase 1
5	4	37.2	21.935	13.108	P62995-3;P62995	Transformer-2 protein homolog beta
7	6	24.4	55.364	13.085	Q92769;Q92769-3	Histone deacetylase 2
8	8	17.5	68.283	13.071	Q96G03	Phosphoglucomutase-2
5	4	26.5	23.818	13.058	O15173;O15173-2	Membrane-associated progesterone receptor component 2
7	7	21.2	47.308	13.053	Q6P4E1-5;Q6P4E1;Q6P4E1-2	Protein CASC4
6	6	18.7	42.741	13.049	P30740;P30740-2	Leukocyte elastase inhibitor
7	4	17.3	57.81	13.036	O00505	Importin subunit alpha-4
9	9	17.5	71.289	13.028	Q9UG63;Q9UG63-2	ATP-binding cassette sub-family F member 2
5	5	12.8	54.489	13.016	Q9UBM7	7-dehydrocholesterol reductase
5	5	10.1	54.047	13.015	Q9H2H9	Sodium-coupled neutral amino acid transporter 1
5	5	17	49.419	12.974	Q9BQ67	Glutamate-rich WD repeat-containing protein 1
11	11	48	25.542	12.959	Q13242	Serine/arginine-rich splicing factor 9
17	11	10	227.87	12.956	Q7Z406;Q7Z406-6;Q7Z406-2;Q7Z406-5;Q7Z406-4	Myosin-14
4	4	15.3	39.562	12.947	O43493-4;O43493;O43493-5;O43493-3;O43493-7;O43493-6	Trans-Golgi network integral membrane protein 2
7	7	19.2	52.488	12.932	Q96EY1;Q96EY1-2	DnaJ homolog subfamily A member 3, mitochondrial
9	9	11.1	100.23	12.917	Q7Z2K6	Endoplasmic reticulum metallopeptidase 1
7	7	16	67.867	12.889	A1L0T0	Acetolactate synthase-like protein
5	5	20.1	34.96	12.887	Q8IZ81	ELMO domain-containing protein 2
5	5	33.7	20.471	12.884	Q03135;Q03135-2	Caveolin-1
4	4	44	14.199	12.866	Q9UI30;Q9UI30-2	Multifunctional methyltransferase subunit TRM112-like protein
4	4	33.3	18.281	12.864	sp|P02754|;CON__P02754	
5	5	69.3	12.774	12.837	O75368	SH3 domain-binding glutamic acid-rich-like protein
5	5	19.5	42.15	12.831	Q8NFH3	Nucleoporin Nup43
4	4	36	15.798	12.824	P61353	60S ribosomal protein L27

7	7	23.5	40.532	12.822	P14324-2;P14324	Farnesyl pyrophosphate synthase
6	6	14.1	53.579	12.793	Q8WTV0-4;Q8WTV0-2;sp|pGC1|;Q8WTV0-5;Q8WTV0;Q8WTV0-3	Scavenger receptor class B member 1
5	2	59.5	12.663	12.787	P63027	Vesicle-associated membrane protein 2
4	4	6.4	106.91	12.739	Q13111;Q13111-2;Q13111-3	Chromatin assembly factor 1 subunit A
4	3	13.9	55.272	12.721	Q8NCA5	Protein FAM98A
5	5	28.5	32.904	12.717	Q9BU89	Deoxyhypusine hydroxylase
6	6	24.2	32.749	12.708	Q86U42;Q86U42-2;Q92843-2	Polyadenylate-binding protein 2
5	5	15.6	48.803	12.695	P10909-4;P10909-6;P10909;P10909-5;P10909-2;P10909-3	Clusterin;Clusterin beta chain;Clusterin alpha chain
6	6	29.8	18.925	12.687	Q8WW12;Q8WW12-2	PEST proteolytic signal-containing nuclear protein
6	6	7.8	109.42	12.633	Q8N766-4;Q8N766-3;Q8N766-2;Q8N766	ER membrane protein complex subunit 1
6	5	21.2	42.777	12.619	P50750;P50750-2	Cyclin-dependent kinase 9
4	4	23.4	27.067	12.618	Q9UNE7-2;Q9UNE7	E3 ubiquitin-protein ligase CHIP
7	3	16.5	65.695	12.616	Q9UHD9	Ubiquilin-2
1	1	9.4	12.249	12.616	Q9UII2	ATPase inhibitor, mitochondrial
5	5	7	85.046	12.611	Q13433;Q13433-2	Zinc transporter ZIP6
7	7	24.3	43.206	12.61	Q9Y305-3;Q9Y305-2;Q9Y305;Q9Y305-4	Acyl-coenzyme A thioesterase 9, mitochondrial
10	10	21.9	70.737	12.602	Q969Z0;Q969Z0-2	Protein TBRG4
4	4	5	141.1	12.595	Q2NKX8	DNA excision repair protein ERCC-6-like
9	9	23.5	59.975	12.591	Q8N7H5;Q8N7H5-3;Q8N7H5-2	RNA polymerase II-associated factor 1 homolog
5	5	13.9	51.103	12.589	Q5SWX8;Q5SWX8-3;Q5SWX8-4;Q5SWX8-2	Protein odr-4 homolog
3	3	12	44.385	12.589	Q15427	Splicing factor 3B subunit 4
2	2	25.3	15.892	12.582	Q9NZT1	Calmodulin-like protein 5
7	7	14.9	82.322	12.58	Q9H6S3-3;Q9H6S3;Q9H6S3-2	Epidermal growth factor receptor kinase substrate 8-like protein 2
7	7	49.1	19.398	12.561	Q9GZZ1;Q9GZZ1-2	N-alpha-acetyltransferase 50
8	8	8	156.78	12.555	P30622-2;P30622-1;P30622	CAP-Gly domain-containing linker protein 1
6	0	6.1	140.6	12.538	sp|FA50-20b|	
3	3	46.4	7.8409	12.534	P62857	40S ribosomal protein S28
5	5	18.6	54.181	12.529	P52594-2;P52594-3;P52594;P52594-4	Arf-GAP domain and FG repeat-containing protein 1
8	8	23.6	48.117	12.528	Q9BZE1	39S ribosomal protein L37, mitochondrial
6	6	33.9	24.636	12.487	P22061;P22061-2	Protein-L-isoaspartate(D-aspartate) O-methyltransferase
7	6	16.1	61.189	12.464	Q96FN4;Q96FN4-2	Copine-2
4	3	24.7	24.758	12.461	P20337	Ras-related protein Rab-3B
8	8	32.8	38.792	12.445	O43681	ATPase ASNA1
4	4	48.3	15.911	12.442	Q96C90;P53671-3	Protein phosphatase 1 regulatory subunit 14B
7	7	25.7	38.673	12.436	Q9P2W9	Syntaxin-18
4	4	10.6	56.3	12.408	Q13188;Q13188-2	Serine/threonine-protein kinase 3;Serine/threonine-protein kinase 3 36kDa subunit;Serine/threonine-protein kinase 3 20kDa subunit
8	8	18.8	76.666	12.407	Q9BQ70	Transcription factor 25
6	6	22.3	42.183	12.392	O43837;O43837-2	Isocitrate dehydrogenase [NAD] subunit beta, mitochondrial
5	5	8.1	67.638	12.391	P30825	High affinity cationic amino acid transporter 1
6	6	8.5	81.636	12.381	Q9NRZ9-6;Q9NRZ9-5;Q9NRZ9-3;Q9NRZ9-2;Q9NRZ9;Q9NRZ9-4	Lymphoid-specific helicase
3	3	19	23.51	12.363	Q96E11-8;Q96E11-3;Q96E11	Ribosome-recycling factor, mitochondrial
5	5	23.5	42.192	12.36	Q86WV6	Stimulator of interferon genes protein
8	8	14.3	93.653	12.345	O43490-4;O43490-7;O43490-2;O43490;O43490-5;O43490-3;O43490-6	Prominin-1
20	1	41.4	79.441	12.315	O00429-3;O00429;O00429-5;O00429-6;O00429-7	Dynamin-1-like protein
6	6	12.4	77.422	12.313	Q13586;Q13586-2	Stromal interaction molecule 1

3	3	12.4	39.542	12.308	Q8WVM0	Dimethyladenosine transferase 1, mitochondrial
7	7	46.3	12.855	12.294	O75347;O75347-2	Tubulin-specific chaperone A
5	5	14.1	53.413	12.294	Q05519-2;Q05519	Serine/arginine-rich splicing factor 11
7	7	6.8	128.46	12.292	O95239-2;O95239	Chromosome-associated kinesin KIF4A
7	7	7	158.77	12.289	P23470-2;P23470	Receptor-type tyrosine-protein phosphatase gamma
4	2	18.1	39.821	12.27	P17612-2;P17612	cAMP-dependent protein kinase catalytic subunit alpha
7	4	43.7	23.707	12.259	P61020;P61020-2	Ras-related protein Rab-5B
9	5	16.4	87.652	12.253	sp|QFA12-17|GST-p65_HUMAN;sp|Q04206|TF65_HUMAN;Q04206-2;Q04206-3;Q04206	Transcription factor p65
6	6	22.5	30.895	12.225	P42126-2;P42126	Enoyl-CoA delta isomerase 1, mitochondrial
8	8	24.1	48.948	12.216	Q06265;Q06265-2;Q06265-3;Q06265-4	Exosome complex component RRP45
4	4	12.1	46.53	12.2	P00966	Argininosuccinate synthase
7	7	16.8	70.505	12.195	CON__P00735;sp|P00735|	
5	5	20.9	23.025	12.194	Q15286;Q15286-2	Ras-related protein Rab-35
1	1	15.2	21.522	12.193	P02686-2;P02686	Myelin basic protein
5	5	18.4	31.484	12.184	Q9Y3B9	RRP15-like protein
5	5	48.7	13.015	12.182	P62854;Q5JNZ5	40S ribosomal protein S26;Putative 40S ribosomal protein S26-like 1
6	6	16.7	60.618	12.182	Q69YN2;Q69YN2-3	CWF19-like protein 1
6	6	12.6	60.348	12.171	Q8N8S7-3;Q8N8S7-2;Q8N8S7	Protein enabled homolog
8	8	17.3	73.776	12.136	P23786	Carnitine O-palmitoyltransferase 2, mitochondrial
5	5	37.4	20.55	12.133	Q9ULC4-3;Q9ULC4;Q9ULC4-2	Malignant T-cell-amplified sequence 1
5	5	26.4	30.993	12.118	P20645	Cation-dependent mannose-6-phosphate receptor
5	5	17.3	53.327	12.114	Q9UKL0	REST corepressor 1
2	2	4.8	96.149	12.101	Q92854	Semaphorin-4D
5	5	22.8	34.833	12.094	Q00403	Transcription initiation factor IIB
8	8	11.8	104.76	12.092	Q01974	Tyrosine-protein kinase transmembrane receptor ROR2
2	2	25.5	14.852	12.079	Q86Y39;Q86Y39-2	NADH dehydrogenase [ubiquinone] 1 alpha subcomplex subunit 11
4	4	30.6	17.676	12.061	Q8WVJ2	NudC domain-containing protein 2
5	5	12	78.863	12.057	O00566	U3 small nucleolar ribonucleoprotein protein MPP10
2	2	22.8	16.298	12.054	Q15388	Mitochondrial import receptor subunit TOM20 homolog
3	3	9.4	49.666	12.052	Q8IYU8	Calcium uptake protein 2, mitochondrial
5	5	9.1	82.921	12.031	Q12996	Cleavage stimulation factor subunit 3
7	7	34.5	28.38	12.021	P13984	General transcription factor IIF subunit 2
5	5	14.9	54.271	12.017	Q969S3	Zinc finger protein 622
6	6	14.5	58.805	12.008	P49643	DNA primase large subunit
5	5	36.4	18.998	12.007	P51571	Translocon-associated protein subunit delta
6	6	5.7	140.76	12.006	Q9Y6M7-7;Q9Y6M7-8;Q9Y6M7-13;Q9Y6M7-6;Q9Y6M7-9;Q9Y6M7-12;Q9Y6M7;Q9Y6M7-2;Q9Y6M7-14;Q9Y6M7-3;Q9Y6M7-4;Q9Y6M7-5;Q9Y6M7-10;Q9Y6M7-11	Sodium bicarbonate cotransporter 3
3	3	8.5	51.927	11.994	P48723	Heat shock 70 kDa protein 13
6	6	6.2	155.98	11.986	Q9H2M9	Rab3 GTPase-activating protein non-catalytic subunit
8	8	18.2	64.218	11.978	Q10472	Polypeptide N-acetylgalactosaminyltransferase 1;Polypeptide N-acetylgalactosaminyltransferase 1 soluble form
4	4	12.5	55.676	11.974	Q16851-2;Q16851	UTP--glucose-1-phosphate uridylyltransferase
3	3	21.3	22.04	11.918	P30405;P30405-2	Peptidyl-prolyl cis-trans isomerase F, mitochondrial
3	3	34	10.659	11.898	P05114	Non-histone chromosomal protein HMG-14
7	7	12.1	85.486	11.89	Q06481-3;Q06481-6;Q06481;Q06481-4;Q06481-2;Q06481-5	Amyloid-like protein 2
4	4	23.7	31.648	11.889	Q9NRY6	Phospholipid scramblase 3
8	8	6.5	194.81	11.886	O95602	DNA-directed RNA polymerase I subunit RPA1

3	3	18.7	22.774	11.874	Q9Y6A4	Cilia- and flagella-associated protein 20
6	6	41.7	17.695	11.853	P62750	60S ribosomal protein L23a
4	4	18	26.724	11.848	O96011-2;O96011	Peroxisomal membrane protein 11B
5	5	23.4	40.329	11.846	P61421	V-type proton ATPase subunit d 1
5	5	51	11.438	11.818	Q9BV40	Vesicle-associated membrane protein 8
5	5	17.8	51.958	11.786	P55081	Microfibrillar-associated protein 1
9	9	17.1	89.333	11.776	Q96SB3	Neurabin-2
6	6	15.5	47.534	11.773	Q9Y276	Mitochondrial chaperone BCS1
5	5	24.7	27.978	11.759	P57088	Transmembrane protein 33
6	6	22.4	46.086	11.753	O14639-4;O14639-3;O14639-5;O14639-2;O14639-6;O14639	Actin-binding LIM protein 1
7	7	30.1	31.291	11.75	Q9Y639-1;Q9Y639;Q9Y639-3;Q9Y639-4;Q9Y639-5	Neuroplastin
4	4	10.7	56.486	11.731	O15091-4;O15091;O15091-2	Mitochondrial ribonuclease P protein 3
4	4	15.9	35.934	11.722	Q9NS00-2;Q9NS00	Glycoprotein-N-acetylgalactosamine 3-beta-galactosyltransferase 1
7	7	11.1	86.982	11.698	Q13617;Q13617-2	Cullin-2
6	6	15	55.01	11.69	Q01650	Large neutral amino acids transporter small subunit 1
4	4	30.8	18.856	11.675	Q9UMX5	Neudesin
8	8	43.2	19.576	11.672	P13073	Cytochrome c oxidase subunit 4 isoform 1, mitochondrial
7	6	20.1	56.466	11.672	Q8WVV9-5;Q8WVV9-4;Q8WVV9	Heterogeneous nuclear ribonucleoprotein L-like
7	7	3.7	265.4	11.669	Q04721	Neurogenic locus notch homolog protein 2;Notch 2 extracellular truncation;Notch 2 intracellular domain
9	9	9.2	162.46	11.664	Q15075	Early endosome antigen 1
5	3	14.8	51.388	11.65	Q9Y6M4;Q9Y6M4-3;Q9Y6M4-4;Q9Y6M4-2;Q9Y6M4-6;Q9Y6M4-5	Casein kinase I isoform gamma-3
4	4	21.7	29.642	11.648	Q9Y320-2;Q9Y320	Thioredoxin-related transmembrane protein 2
4	4	20	22.98	11.645	O15347	High mobility group protein B3
4	4	35.4	12.497	11.634	P61803	Dolichyl-diphosphooligosaccharide--protein glycosyltransferase subunit DAD1
3	3	3.7	135.76	11.632	P39060-2;P39060-1;P39060	Collagen alpha-1(XVIII) chain;Endostatin
5	5	2.9	286.79	11.626	Q92508	Piezo-type mechanosensitive ion channel component 1
2	2	6	57.504	11.625	Q9Y6I3-3;Q9Y6I3;Q9Y6I3-1	Epsin-1
9	9	9.7	117.67	11.607	O94979-6;O94979-3;O94979-10;O94979-4;O94979-9;O94979-2;O94979;O94979-8;O94979-7;O94979-5	Protein transport protein Sec31A
6	6	31.2	35.61	11.595	Q96HY6;Q96HY6-2	DDRGK domain-containing protein 1
8	8	13.1	93.02	11.589	P78536;P78536-2	Disintegrin and metalloproteinase domain-containing protein 17
8	7	8.6	134.85	11.586	P04626-5;P04626-4;P04626;P04626-3;P04626-2;P04626-6	Receptor tyrosine-protein kinase erbB-2
3	3	22.5	11.53	11.579	P20962	Parathymosin
7	7	7	143.19	11.576	O14976;O14976-2	Cyclin-G-associated kinase
8	8	9.9	120.97	11.551	Q03701	CCAAT/enhancer-binding protein zeta
8	8	29	38.703	11.549	P55263-2;P55263;P55263-3;P55263-4	Adenosine kinase
5	5	32.3	10.438	11.548	Q9H299	SH3 domain-binding glutamic acid-rich-like protein 3
7	7	11.4	81.879	11.546	O00461	Golgi integral membrane protein 4
6	6	35.3	24.449	11.539	P21964-2;P21964	Catechol O-methyltransferase
3	3	12.7	26.256	11.537	Q96EC8	Protein YIPF6
7	4	13.8	72.697	11.533	Q15418-3;Q15418-4;Q15418;Q15418-2;Q15349;Q15349-3;Q15349-2	Ribosomal protein S6 kinase alpha-1;Ribosomal protein S6 kinase alpha-2
6	6	13.7	58.414	11.514	Q8TED0;Q8TED0-3	U3 small nucleolar RNA-associated protein 15 homolog
8	7	5.8	238.27	11.507	Q96N67-4;Q96N67-3;Q96N67-5;Q96N67-2;Q96N67 6;Q96N67	Dedicator of cytokinesis protein 7
4	4	45.3	10.112	11.503	P49458;P49458-2	Signal recognition particle 9 kDa protein

5	5	16.4	47.485	11.469	P45954;P45954-2	Short/branched chain specific acyl-CoA dehydrogenase, mitochondrial
7	4	54.1	20.504	11.462	P61225	Ras-related protein Rap-2b
7	7	32.7	25.835	11.458	Q96CT7	Coiled-coil domain-containing protein 124
5	5	34.1	18.565	11.408	P40616-2;P40616	ADP-ribosylation factor-like protein 1
5	5	20.5	33.812	11.408	Q8N6M0	OTU domain-containing protein 6B
6	6	24.5	42.785	11.389	Q8NFZ8	Cell adhesion molecule 4
8	8	14.3	79.748	11.375	Q9NQX3;Q9NQX3-2	Gephyrin;Molybdopterin adenylyltransferase;Molybdopterin molybdenumtransferase
6	6	11.6	72.023	11.366	P39880-9;Q13948-10;Q13948;Q13948-9;Q13948-2;P39880-6;P39880-4;P39880-5;P39880-2;P39880;P39880-3	Homeobox protein cut-like 1;Protein CASP
9	9	24.8	56.878	11.361	P53041	Serine/threonine-protein phosphatase 5
7	7	5.1	236.28	11.357	Q12789-3;Q12789	General transcription factor 3C polypeptide 1
7	3	39.1	23.584	11.354	Q92930	Ras-related protein Rab-8B
5	4	32.5	22.225	11.35	P45973	Chromobox protein homolog 5
6	6	10.2	53.944	11.347	P53985	Monocarboxylate transporter 1
13	13	11.5	174.76	11.332	P35573;P35573-2;P35573-3	Glycogen debranching enzyme;4-alpha-glucanotransferase;Amylo-alpha-1,6-glucosidase
4	4	8.6	44.882	11.328	P11279;P11279-2	Lysosome-associated membrane glycoprotein 1
6	5	13.7	49.301	11.316	O75351	Vacuolar protein sorting-associated protein 4B
8	8	25.8	45.083	11.31	O14763-2;O14763	Tumor necrosis factor receptor superfamily member 10B
6	6	13.7	61.327	11.306	Q9Y692-2;Q9Y692	Glucocorticoid modulatory element-binding protein 1
4	4	22.7	21.749	11.276	Q96GC5-3;Q96GC5	39S ribosomal protein L48, mitochondrial
5	5	11.3	61.728	11.273	Q9NUQ6;Q9NUQ6-4;Q9NUQ6-3;Q9NUQ6-2	SPATS2-like protein
7	7	25.2	35.503	11.268	Q9BWM7	Sideroflexin-3
21	1	47	61.815	11.249	P08195-3	4F2 cell-surface antigen heavy chain
8	8	17.8	51.712	11.247	O75390	Citrate synthase, mitochondrial
6	6	4.8	210.11	11.216	Q15154-4;Q15154-2;Q15154-5;Q15154	Pericentriolar material 1 protein
4	4	43	9.7251	11.213	P62306	Small nuclear ribonucleoprotein F
5	5	18.9	47.862	11.206	P22830;P22830-2	Ferrochelatase, mitochondrial
6	6	9.6	83.069	11.195	Q8IYQ7	Threonine synthase-like 1
5	5	17	35.808	11.194	O00214;O00214-2	Galectin-8
3	3	21.2	23.864	11.177	Q96EY4	Translation machinery-associated protein 16
4	4	23.2	22.574	11.164	Q9NP77	RNA polymerase II subunit A C-terminal domain phosphatase SSU72
9	9	14.7	92.902	11.164	O60341;O60341-2	Lysine-specific histone demethylase 1A
4	4	16	23.656	11.153	P14678-2;P63162;P14678;P63162-2;P14678-3	Small nuclear ribonucleoprotein-associated proteins B and B;Small nuclear ribonucleoprotein-associated protein N
5	5	3.9	204.74	11.143	O60437	Periplakin
4	4	6.8	97.031	11.103	P52735-3;P52735-2;P52735	Guanine nucleotide exchange factor VAV2
5	5	17.8	38.711	11.091	Q9NYK5;Q9NYK5-2	39S ribosomal protein L39, mitochondrial
5	5	8.7	92.239	11.087	Q9UHI6	Probable ATP-dependent RNA helicase DDX20
5	5	21.2	34.852	11.08	Q99848	Probable rRNA-processing protein EBP2
4	4	21.2	31.73	11.075	P27105;P27105-2	Erythrocyte band 7 integral membrane protein
8	8	16.1	71.635	11.067	Q8NBJ5	Procollagen galactosyltransferase 1
6	6	14.9	48.006	11.053	P82675;P82675-2	28S ribosomal protein S5, mitochondrial
4	4	16.6	38.247	11.042	Q9GZM5	Protein YIPF3;Protein YIPF3, 36 kDa form III
3	3	30.5	12.712	11.039	P30046;P30046-2;A6NHG4	D-dopachrome decarboxylase;D-dopachrome decarboxylase-like protein
9	3	56.5	20.987	11.036	P62834	Ras-related protein Rap-1A
6	6	31.6	26.72	11.024	Q92979	Ribosomal RNA small subunit methyltransferase NEP1
8	8	27.2	41.859	11.013	Q9NXW2;Q9NXW2-2	DnaJ homolog subfamily B member 12
4	4	18.9	36.492	11.01	P49354-2;P49354	Protein farnesyltransferase/geranylgeranyltransferase type-1 subunit alpha
4	4	18	20.863	10.996	P07305;P07305-2	Histone H1.0;Histone H1.0, N-terminally processed
2	2	11.2	29.014	10.95	O95983-2;O95983	Methyl-CpG-binding domain protein 3
8	8	40.5	18.431	10.945	P62280	40S ribosomal protein S11

4	4	8.2	75.035	10.943	Q96PZ0;Q96PZ0-2	Pseudouridylate synthase 7 homolog
5	4	28	25.486	10.929	P08579	U2 small nuclear ribonucleoprotein B
8	5	16.4	68.934	10.927	Q9H0B6;Q9H0B6-2	Kinesin light chain 2
5	5	10.6	84.639	10.923	O15111	Inhibitor of nuclear factor kappa-B kinase subunit alpha
7	7	13.2	70.631	10.921	Q8NE01-2;Q8NE01-3;Q8NE01	Metal transporter CNNM3
5	4	25.1	27.277	10.914	Q9BVK6	Transmembrane emp24 domain-containing protein 9
5	4	23.9	24.033	10.914	P26583	High mobility group protein B2
6	6	26	34.324	10.912	Q53GQ0	Very-long-chain 3-oxoacyl-CoA reductase
5	5	54	13.711	10.894	O75380	NADH dehydrogenase [ubiquinone] iron-sulfur protein 6, mitochondrial
4	4	43.1	13.459	10.885	Q16718;Q16718-2	NADH dehydrogenase [ubiquinone] 1 alpha subcomplex subunit 5
7	7	4.7	226.37	10.877	A6NHR9;A6NHR9-2;A6NHR9-3	Structural maintenance of chromosomes flexible hinge domain-containing protein 1
5	5	9.2	83.147	10.875	Q9BZJ0-2;Q9BZJ0-3;Q9BZJ0	Crooked neck-like protein 1
9	4	10.3	124.84	10.869	Q8TAQ2-2;Q8TAQ2-3;Q8TAQ2	SWI/SNF complex subunit SMARCC2
4	4	41.7	17.162	10.867	Q9BX68	Histidine triad nucleotide-binding protein 2, mitochondrial
10	10	24.2	60.585	10.853	Q9NUQ3;Q9NUQ3-2	Gamma-taxilin
3	3	19.3	26.748	10.841	Q9NWU2	Glucose-induced degradation protein 8 homolog
1	1	4.7	40.907	10.83	O95684-2;O95684	FGFR1 oncogene partner
4	4	32.9	19.608	10.814	O43665-2;O43665-1;O43665	Regulator of G-protein signaling 10
5	5	23.9	34.773	10.781	Q8NFH5;Q8NFH5-2	Nucleoporin NUP53
7	7	71	12.122	10.764	Q9GZT3-2;Q9GZT3	SRA stem-loop-interacting RNA-binding protein, mitochondrial
4	4	16.5	38.989	10.764	P49770	Translation initiation factor eIF-2B subunit beta
10	10	16	95.581	10.75	Q9BUQ8	Probable ATP-dependent RNA helicase DDX23
6	6	29.8	28.134	10.738	Q9Y2R9	28S ribosomal protein S7, mitochondrial
5	5	6.7	116.99	10.732	Q13523	Serine/threonine-protein kinase PRP4 homolog
4	4	8	105.43	10.706	P19838-2;P19838;P19838-3	Nuclear factor NF-kappa-B p105 subunit;Nuclear factor NF-kappa-B p50 subunit
3	3	31.2	17.909	10.69	Q99627-2;Q99627	COP9 signalosome complex subunit 8
3	3	24.5	16.57	10.69	P61916;P61916-2	Epididymal secretory protein E1
7	7	15.9	58.957	10.689	P13674-3;P13674-2;P13674	Prolyl 4-hydroxylase subunit alpha-1
3	3	5	86.401	10.682	O95140	Mitofusin-2
5	5	8.9	77.485	10.68	Q9UKF6	Cleavage and polyadenylation specificity factor subunit 3
5	5	29.3	20.35	10.675	Q9BU61	NADH dehydrogenase [ubiquinone] 1 alpha subcomplex assembly factor 3
4	4	6.9	91.271	10.674	Q5JSH3-4;Q5JSH3-2;Q5JSH3	WD repeat-containing protein 44
7	7	20.2	54.794	10.664	Q0VDF9	Heat shock 70 kDa protein 14
3	3	21.8	26.131	10.657	O75934	Pre-mRNA-splicing factor SPF27
8	8	9.9	119.94	10.603	O15397;O15397-2	Importin-8
7	7	14.3	65.853	10.569	Q86X55;Q86X55-1;Q86X55-2	Histone-arginine methyltransferase CARM1
5	5	36.1	21.868	10.564	O75340;O75340-2	Programmed cell death protein 6
4	4	27	23.39	10.562	Q7Z4H3;Q7Z4H3-2	HD domain-containing protein 2
7	6	40.3	21.258	10.556	P60953;P60953-1	Cell division control protein 42 homolog
5	5	13.4	50.315	10.555	Q9H9P8;Q9H9P8-2	L-2-hydroxyglutarate dehydrogenase, mitochondrial
6	6	10.7	77.318	10.547	Q2PZI1	Probable C-mannosyltransferase DPY19L1
7	7	41.2	18.565	10.541	P46778	60S ribosomal protein L21
8	7	6.8	165.65	10.529	P31327-3;P31327;P31327-2	Carbamoyl-phosphate synthase [ammonia], mitochondrial
4	4	14.6	48.05	10.506	Q96RD7;Q96RD7-2	Pannexin-1
10	4	33.1	42.142	10.488	P50148	Guanine nucleotide-binding protein G(q) subunit alpha
3	3	6	79.845	10.475	Q8IWA5-3;Q8IWA5;Q8IWA5-2	Choline transporter-like protein 2
4	4	13.8	39.393	10.446	Q14558;Q14558-2	Phosphoribosyl pyrophosphate synthase-associated protein 1
3	3	12.3	30.712	10.419	Q6UXH1-3;Q6UXH1-2;Q6UXH1-4;Q6UXH1;Q6UXH1-6;Q6UXH1-5	Cysteine-rich with EGF-like domain protein 2
3	3	20	29.909	10.413	Q8WXX5	DnaJ homolog subfamily C member 9
5	5	17.9	44.017	10.409	Q9Y697-2;Q9Y697;Q9Y697-3	Cysteine desulfurase, mitochondrial

6	6	10.3	85.411	10.4	sp|Q3KUS7|;CON__Q3KUS7	
4	4	15.7	35.277	10.385	O15479	Melanoma-associated antigen B2
4	4	10.8	51.419	10.385	Q13418;Q13418-2;Q13418-3	Integrin-linked protein kinase
7	7	25.1	39.609	10.383	O75521-2;O75521	Enoyl-CoA delta isomerase 2, mitochondrial
3	3	10.4	34.392	10.367	O43688-2;O43688;O43688-3	Lipid phosphate phosphohydrolase 2
5	5	5.9	118.02	10.363	O60524-4;O60524-5;O60524-3;O60524	Nuclear export mediator factor NEMF
8	6	9	124.42	10.361	Q14671-2;Q14671;Q14671-3;Q14671-4	Pumilio homolog 1
5	5	8.1	82.324	10.36	Q9UBD5-2;Q9UBD5;Q9UBD5-3	Origin recognition complex subunit 3
4	4	43.5	16.829	10.359	P50583	Bis(5-nucleosyl)-tetraphosphatase [asymmetrical]
6	6	13.3	45.861	10.354	Q99442	Translocation protein SEC62
1	1	5.9	44.772	10.349	Q96K37	Solute carrier family 35 member E1
4	4	21.3	32.895	10.346	Q14914-2;Q14914	Prostaglandin reductase 1
6	6	13.4	76.749	10.342	P17252	Protein kinase C alpha type
6	6	9.9	102.4	10.336	Q7Z2K8;Q7Z2K8-2	G protein-regulated inducer of neurite outgrowth 1
7	7	14.6	60.718	10.322	Q9H857;Q9H857-3;Q9H857-2;Q9H857-4	5-nucleotidase domain-containing protein 2
4	4	11.3	53.743	10.32	Q9Y6I4-2;Q9Y6I4	Ubiquitin carboxyl-terminal hydrolase 3
6	6	29.9	29.299	10.317	Q9BZX2	Uridine-cytidine kinase 2
2	2	18.8	25.827	10.317	Q8IZR5;Q8IZR5-2;Q8IZR5-3	CKLF-like MARVEL transmembrane domain-containing protein 4
3	3	20.9	26.076	10.313	Q14696	LDLR chaperone MESD
4	4	12.7	46.258	10.308	P30989	Neurotensin receptor type 1
4	4	25.1	24.409	10.298	sp|P00761|;CON__P00761	
6	6	7	117.11	10.286	Q5T6F2	Ubiquitin-associated protein 2
3	3	34.5	12.405	10.278	Q7RTV0	PHD finger-like domain-containing protein 5A
4	4	10.1	68.254	10.273	P53350	Serine/threonine-protein kinase PLK1
9	9	12.9	102.11	10.256	O14936-3;O14936-4;O14936-6;O14936-2;O14936;O14936-5	Peripheral plasma membrane protein CASK
7	7	50.3	19.856	10.251	Q8N183	Mimitin, mitochondrial
2	2	35.8	9.0564	10.251	Q9P1F3	Costars family protein ABRACL
8	7	23.6	51.263	10.247	Q8NFQ8	Torsin-1A-interacting protein 2
1	1	4.3	52.417	10.241	Q92733	Proline-rich protein PRCC
7	7	44.7	21.77	10.233	Q9Y3D9	28S ribosomal protein S23, mitochondrial
3	3	10.2	46.017	10.232	CON__Q9TT36;sp|Q9TT36|	
6	6	6.2	142.44	10.221	Q9NVI1-2;Q9NVI1-1;Q9NVI1;Q9NVI1-4	Fanconi anemia group I protein
5	4	17.5	48.678	10.209	Q8IZP0-2;Q8IZP0-4;Q8IZP0-3;Q8IZP0-5;Q8IZP0-6;Q8IZP0-9;Q8IZP0;Q8IZP0-10;Q8IZP0-8;Q8IZP0-7;Q8IZP0-12;Q8IZP0-11	Abl interactor 1
10	10	10.2	135.65	10.204	O95347;O95347-2	Structural maintenance of chromosomes protein 2
4	4	16	29.044	10.203	P53365-2;P53365-3;P53365	Arfaptin-2
4	4	16.7	39.134	10.197	O75794	Cell division cycle protein 123 homolog
2	2	38.4	11.25	10.194	Q9C005	Protein dpy-30 homolog
2	2	5.3	51.723	10.192	sp|P50448|;CON__P50448	
32	0	48.2	83.677	10.171	Q16891;Q16891-3	MICOS complex subunit MIC60
5	5	16.8	42.033	10.168	Q9Y2S7	Polymerase delta-interacting protein 2
5	3	10.1	74.222	10.162	P51116	Fragile X mental retardation syndrome-related protein 2
5	5	20.7	36.588	10.159	P61964	WD repeat-containing protein 5
5	5	9.1	88.828	10.156	O43264;O43264-2	Centromere/kinetochore protein zw10 homolog
4	4	35.6	11.999	10.147	P61457	Pterin-4-alpha-carbinolamine dehydratase
8	8	9.9	120.04	10.135	P57678	Gem-associated protein 4
6	6	25.2	33.843	10.127	Q8NBJ7;Q8NBJ7-2;Q8NBJ7-5;Q8NBJ7-3	Sulfatase-modifying factor 2
16	6	38.8	46.402	10.125	Q14240;Q14240-2	Eukaryotic initiation factor 4A-II;Eukaryotic initiation factor 4A-II, N-terminally processed
3	3	16.2	24.915	10.124	Q96A35	39S ribosomal protein L24, mitochondrial

4	4	45.5	13.569	10.108	P35244	Replication protein A 14 kDa subunit
5	5	7.8	117.35	10.105	Q9UIQ6;Q9UIQ6-3;Q9UIQ6-2	Leucyl-cystinyl aminopeptidase;Leucyl-cystinyl aminopeptidase, pregnancy serum form
5	5	9.5	82.597	10.104	Q8N9T8	Protein KRI1 homolog
6	6	4.8	220.62	10.1	Q14966;Q14966-5;Q14966-3	Zinc finger protein 638
4	4	11.7	52.025	10.097	Q15904	V-type proton ATPase subunit S1
3	3	14.6	47.025	10.095	Q9BTD8-4;Q9BTD8-2;Q9BTD8-3;Q9BTD8	RNA-binding protein 42
5	5	14.8	42.072	10.079	Q9NUQ2	1-acyl-sn-glycerol-3-phosphate acyltransferase epsilon
7	7	21.4	42.139	10.062	P56589	Peroxisomal biogenesis factor 3
6	6	15.5	65.522	10.054	Q13131-2;Q13131	5-AMP-activated protein kinase catalytic subunit alpha-1
4	4	17.7	18.988	10.038	P67812-4;P67812;P67812-3;P67812-2	Signal peptidase complex catalytic subunit SEC11A
3	3	12	35.119	10.037	Q6IAN0	Dehydrogenase/reductase SDR family member 7B
7	7	23.1	37.998	10.028	Q6P4A7;Q6P4A7-2	Sideroflexin-4
8	6	13	74.761	10.023	O75746;O75746-2	Calcium-binding mitochondrial carrier protein Aralar1
6	6	30.1	28.075	9.9939	Q9H832-2;Q9H832	Ubiquitin-conjugating enzyme E2 Z
4	4	16.7	54.985	9.9919	O15446;O15446-2	DNA-directed RNA polymerase I subunit RPA34
5	5	46.2	14.839	9.9796	P62244	40S ribosomal protein S15a
4	4	16.9	28.368	9.9765	Q6Y1H2	Very-long-chain (3R)-3-hydroxyacyl-CoA dehydratase 2
6	6	18.6	51.55	9.9748	Q10469	Alpha-1,6-mannosyl-glycoprotein 2-beta-N-acetylglucosaminyltransferase
6	6	11.3	68.641	9.9689	Q9Y289	Sodium-dependent multivitamin transporter
7	7	25.1	27.56	9.9674	Q9UKD2	mRNA turnover protein 4 homolog
4	4	16	36.924	9.9392	P53611	Geranylgeranyl transferase type-2 subunit beta
3	3	25.5	12.538	9.9327	P18077	60S ribosomal protein L35a
3	2	4.6	104.22	9.9295	P46934-4	E3 ubiquitin-protein ligase NEDD4
5	5	22.6	36.172	9.9219	P46734-2;P46734;P46734-3	Dual specificity mitogen-activated protein kinase kinase 3
6	6	11.2	72.399	9.9209	Q71RC2-5;Q71RC2;Q71RC2-4;Q71RC2-7;Q71RC2-3;Q71RC2-6;Q71RC2-2	La-related protein 4
4	4	25.3	17.342	9.9178	Q96A26	Protein FAM162A
3	3	28.6	14.875	9.9171	Q9BRT2	Ubiquinol-cytochrome-c reductase complex assembly factor 2
12	7	17.4	87.679	9.9107	Q13619;Q13619-2	Cullin-4A
4	4	13.6	46.172	9.8807	Q86XR7-2;Q9Y3B3-2;Q9Y3B3	Transmembrane emp24 domain-containing protein 7
2	2	10.2	30.727	9.879	P48507;P48507-2	Glutamate--cysteine ligase regulatory subunit
4	4	9.9	53.557	9.8763	Q96ER3	Protein SAAL1
8	8	7.4	142.16	9.8755	Q86SQ0;Q86SQ0-2;Q86SQ0-3	Pleckstrin homology-like domain family B member 2
6	6	25.9	35.979	9.8697	CON__Q03247;sp|Q03247|	
6	6	15.1	62.411	9.8658	O75487;O75487-2	Glypican-4;Secreted glypican-4
4	4	49.2	13.802	9.8479	P49773	Histidine triad nucleotide-binding protein 1
4	4	11.4	49.452	9.8363	P29083	General transcription factor IIE subunit 1
7	7	30.3	27.566	9.8256	P78417;P78417-3;P78417-2	Glutathione S-transferase omega-1
7	7	10.7	87.99	9.815	Q8IYB8	ATP-dependent RNA helicase SUPV3L1, mitochondrial
1	1	9.2	35.151	9.8045	Q9BWQ6	Protein YIPF2
4	4	21.3	28.462	9.8043	Q9UMS0;Q9UMS0-3;Q9UMS0-2	NFU1 iron-sulfur cluster scaffold homolog, mitochondrial
2	2	30.2	11.776	9.8023	P35754	Glutaredoxin-1
4	4	6	83.125	9.7893	P40763-3;P40763-2;P40763	Signal transducer and activator of transcription 3
5	5	9.4	78.855	9.7803	O75127	Pentatricopeptide repeat-containing protein 1, mitochondrial
5	5	10.8	69.437	9.766	Q86U38;Q86U38-2	Nucleolar protein 9
5	5	20.4	37.025	9.7577	P51665	26S proteasome non-ATPase regulatory subunit 7
8	8	36	31.231	9.7542	Q9NX47	E3 ubiquitin-protein ligase MARCH5
4	4	14.7	40.786	9.7357	P06132	Uroporphyrinogen decarboxylase
4	4	4.2	162.17	9.7302	Q15262-2;Q15262-3;Q15262-4;Q15262	Receptor-type tyrosine-protein phosphatase kappa
3	3	13.7	49.148	9.7274	Q9BQ95;Q9BQ95-4;Q9BQ95-2	Evolutionarily conserved signaling intermediate in Toll pathway, mitochondrial
4	4	17.2	38.608	9.7197	Q96C86	m7GpppX diphosphatase

7	7	6.7	167.35	9.7193	Q08378;Q08378-2;Q08378-4	Golgin subfamily A member 3
2	2	20	15.069	9.7103	P62847-2;P62847-3;P62847;P62847-4	40S ribosomal protein S24
5	5	8.4	86.323	9.7094	Q969S9-3;Q969S9;Q969S9-5;Q969S9-4;Q969S9-2	Ribosome-releasing factor 2, mitochondrial
4	4	67	10.739	9.7064	P07919;A0A096LP55	Cytochrome b-c1 complex subunit 6, mitochondrial;Cytochrome b-c1 complex subunit 6
3	3	6.4	84.046	9.6877	Q8TAD4;Q8TAD4-3	Zinc transporter 5
5	5	41	17	9.686	P41223;P41223-2	Protein BUD31 homolog
3	3	8.4	57.578	9.6774	Q6XQN6;Q6XQN6-3;Q6XQN6-2	Nicotinate phosphoribosyltransferase
6	6	21	43.664	9.6756	Q13601;Q13601-2	KRR1 small subunit processome component homolog
7	7	36.4	32.973	9.661	Q9GZY8-2;Q9GZY8;Q9GZY8-5;Q9GZY8-4;Q9GZY8-3	Mitochondrial fission factor
7	7	26.2	37.535	9.6572	Q9H9J2	39S ribosomal protein L44, mitochondrial
6	6	10.4	83.134	9.6391	P22033	Methylmalonyl-CoA mutase, mitochondrial
5	5	18.2	36.622	9.6347	P53990-2;P53990;P53990-4;P53990-5;P53990-3	IST1 homolog
6	6	14.7	51.851	9.6334	O75306-2;O75306	NADH dehydrogenase [ubiquinone] iron-sulfur protein 2, mitochondrial
2	2	22.9	17.781	9.6297	Q16540	39S ribosomal protein L23, mitochondrial
3	3	5	69.997	9.6244	Q9Y2W2	WW domain-binding protein 11
5	5	9.8	73.562	9.6202	Q6PI48	Aspartate--tRNA ligase, mitochondrial
5	5	12.5	62.608	9.6172	P38432	Coilin
3	3	23.2	18.479	9.6082	O95169-3;O95169-2;O95169	NADH dehydrogenase [ubiquinone] 1 beta subcomplex subunit 8, mitochondrial
5	5	18.1	39.589	9.6012	O43488	Aflatoxin B1 aldehyde reductase member 2
4	4	8.8	80.473	9.5997	Q04446	1,4-alpha-glucan-branching enzyme
4	4	49.1	12.107	9.5978	Q96EL3	39S ribosomal protein L53, mitochondrial
3	3	17.9	22.151	9.5948	Q9UBI1	COMM domain-containing protein 3
7	7	30.8	32.541	9.5878	Q9C004	Protein sprouty homolog 4
7	7	19.7	47.573	9.5819	P21579	Synaptotagmin-1
3	3	1.8	260.32	9.57	Q13439-3;Q13439-4;Q13439;Q13439-5	Golgin subfamily A member 4
7	7	15.2	61.116	9.5689	Q9NZW5	MAGUK p55 subfamily member 6
5	5	21.5	39.007	9.5632	O95210	Starch-binding domain-containing protein 1
6	6	42.4	17.222	9.559	P62277	40S ribosomal protein S13
3	3	18	21.163	9.5564	Q9BQE4	Selenoprotein S
5	5	35	15.747	9.5378	P46779;P46779-2;P46779-3;P46779-4;P46779-5	60S ribosomal protein L28
7	7	11.8	79.147	9.5273	Q9NRK6	ATP-binding cassette sub-family B member 10, mitochondrial
7	7	5.8	205.6	9.5265	P55196-1;P55196;P55196-5;P55196-2;P55196-6;P55196-3	Afadin
4	4	14.5	49.901	9.5238	P49642	DNA primase small subunit
5	5	14.5	59.382	9.5224	O95870-2;O95870	Abhydrolase domain-containing protein 16A
5	5	31.4	21.007	9.5139	O75223	Gamma-glutamylcyclotransferase
6	6	13.7	62.921	9.5118	Q92667-2;Q92667	A-kinase anchor protein 1, mitochondrial
6	6	48.4	20.455	9.5094	P36405	ADP-ribosylation factor-like protein 3
5	5	7.3	133.27	9.5093	O95155-2;O95155;O95155-4;O95155-3	Ubiquitin conjugation factor E4 B
3	3	18.9	33.325	9.5023	Q04323;Q04323-2	UBX domain-containing protein 1
6	1	13.2	57.741	9.5012	Q92692	Nectin-2
4	4	10.3	46.765	9.4982	Q6P1A2-2;Q6P1A2	Lysophospholipid acyltransferase 5
4	4	27.3	18.898	9.4908	P46783;Q9NQ39	40S ribosomal protein S10;Putative 40S ribosomal protein S10-like
2	2	20.1	19.108	9.4831	O43598;O43598-2	2-deoxynucleoside 5-phosphate N-hydrolase 1
9	3	52.3	24.214	9.4826	Q8WUD1;Q8WUD1-2	Ras-related protein Rab-2B
3	3	35	11.202	9.481	sp|P81644|;CON__P81644	
6	1	47.3	21.424	9.4729	P01116-2	GTPase KRas;GTPase KRas, N-terminally processed
5	5	16	28.612	9.4558	O95249	Golgi SNAP receptor complex member 1
3	3	9.8	55.016	9.4466	O94919	Endonuclease domain-containing 1 protein

4	4	8.3	77.504	9.4432	Q05655;Q05655-2	Protein kinase C delta type;Protein kinase C delta type regulatory subunit;Protein kinase C delta type catalytic subunit
4	4	14.3	39.641	9.4425	O75695	Protein XRP2
5	5	47.6	15.807	9.4353	P62266	40S ribosomal protein S23
8	8	32.5	23.819	9.429	P23919;P23919-2	Thymidylate kinase
3	3	56.9	8.0061	9.4282	Q9UBI6	Guanine nucleotide-binding protein G(I)/G(S)/G(O) subunit gamma-12
4	4	5.5	117.15	9.4229	Q9NWH9	SAFB-like transcription modulator
4	4	7.1	79.693	9.4228	P53794	Sodium/myo-inositol cotransporter
5	5	8.1	104.5	9.4211	Q9H9Y6-4;Q9H9Y6-2;Q9H9Y6;Q9H9Y6-3	DNA-directed RNA polymerase I subunit RPA2
3	3	21.6	26.836	9.4173	P55957-2;P55957	BH3-interacting domain death agonist;BH3-interacting domain death agonist p15;BH3-interacting domain death agonist p13;BH3-interacting domain death agonist p11
3	3	25	14.177	9.3802	P13987;P13987-2	CD59 glycoprotein
8	8	4.7	288.89	9.3778	P42345	Serine/threonine-protein kinase mTOR
5	5	22.8	33.467	9.3726	Q15257-3;Q15257-2;Q15257;Q15257-4	Serine/threonine-protein phosphatase 2A activator
6	6	51	21.445	9.3687	Q9BY32;Q9BY32-3;Q9BY32-2	Inosine triphosphate pyrophosphatase
4	4	14.2	43.9	9.3506	sp|E24466|;CON__ENSEMBL:ENSBTAP00000024466;sp|E24462|;CON__ENSEMBL:ENSBTAP00000024462	
5	5	13.6	51.603	9.349	Q9NWS8;Q9NWS8-2	Required for meiotic nuclear division protein 1 homolog
4	4	29.5	15.548	9.3409	O43324-2;O43324	Eukaryotic translation elongation factor 1 epsilon-1
2	2	10.9	36.742	9.3348	O60504-2;O60504	Vinexin
5	5	18.9	33.419	9.3129	Q9P015	39S ribosomal protein L15, mitochondrial
5	5	8.3	101.58	9.2957	A1X283	SH3 and PX domain-containing protein 2B
5	5	26	26.034	9.2915	O75391	Sperm-associated antigen 7
6	6	11.5	87.081	9.2888	Q9Y446;Q9Y446-2	Plakophilin-3
9	2	23.6	44.348	9.2733	Q58FF8	Putative heat shock protein HSP 90-beta 2
4	4	6.4	107.11	9.2725	Q14149	MORC family CW-type zinc finger protein 3
3	3	3.7	123.63	9.2724	P28340	DNA polymerase delta catalytic subunit
3	3	21.5	20.63	9.2637	Q13442	28 kDa heat- and acid-stable phosphoprotein
5	5	11.4	69.458	9.2473	Q96S55-2;Q96S55;Q96S55-3	ATPase WRNIP1
3	3	17.2	31.307	9.2455	Q9Y316-2;Q9Y316;Q9Y316-3	Protein MEMO1
2	2	10.8	29.371	9.2273	Q9NZ43;Q9NZ43-2	Vesicle transport protein USE1
3	3	10.5	29.469	9.2258	Q86T03;Q86T03-2	Type 1 phosphatidylinositol 4,5-bisphosphate 4-phosphatase
6	6	6.9	127.06	9.2187	Q12965	Unconventional myosin-Ie
5	5	21	34.094	9.2048	Q9UHQ9	NADH-cytochrome b5 reductase 1
6	6	38.3	18.762	9.1866	O60493;O60493-4;O60493-2	Sorting nexin-3
4	4	9.6	67.56	9.184	P29350;P29350-3;P29350-4;P29350-2	Tyrosine-protein phosphatase non-receptor type 6
8	8	10.4	102.44	9.1782	Q9HC35-2;Q9HC35	Echinoderm microtubule-associated protein-like 4
5	5	30	25.097	9.1737	P06730;P06730-2;P06730-3	Eukaryotic translation initiation factor 4E
3	3	4.2	134.19	9.172	Q9Y2G3	Probable phospholipid-transporting ATPase IF
3	3	34.3	11.721	9.1649	Q16890-4;Q16890-3;Q16890-2;Q16890-5;Q16890	Tumor protein D53
5	5	7.1	97.394	9.1584	Q9NTZ6	RNA-binding protein 12
4	4	18.5	36.167	9.1568	Q8N9F7;Q8N9F7-3;Q8N9F7-2	Glycerophosphodiester phosphodiesterase domain-containing protein 1
7	7	39.2	29.246	9.1171	P00918	Carbonic anhydrase 2
4	4	48.2	12.615	9.1168	O14548	Cytochrome c oxidase subunit 7A-related protein, mitochondrial
1	1	14.5	18.373	9.1053	Q9NVM1	Protein eva-1 homolog B
5	5	35.6	13.133	9.1014	Q15370;Q15370-2	Transcription elongation factor B polypeptide 2
4	4	23.2	29.057	9.0995	A1L170;A1L170-2	Uncharacterized protein C1orf226
5	5	27.8	24.682	9.0925	O00233;O00233-2	26S proteasome non-ATPase regulatory subunit 9
7	7	3.8	251.46	9.0891	Q8N3C0	Activating signal cointegrator 1 complex subunit 3
5	5	24.6	27.924	9.0785	Q9NRX1	RNA-binding protein PNO1

2	2	10.2	33.067	9.0729	P13726;P13726-2	Tissue factor
4	4	26	29.644	9.0571	O95865	N(G),N(G)-dimethylarginine dimethylaminohydrolase 2
3	3	18.5	23.845	9.0557	Q16763	Ubiquitin-conjugating enzyme E2 S
5	5	30.6	20.567	9.0555	P21291	Cysteine and glycine-rich protein 1
3	2	30.3	15.396	9.0395	Q8N490-2	Probable hydrolase PNKD
5	5	3.1	254.41	9.0356	O75165	DnaJ homolog subfamily C member 13
6	6	10.1	88.486	9.035	Q9P2I0	Cleavage and polyadenylation specificity factor subunit 2
4	4	8.9	66.63	9.0284	Q9H1C4	Protein unc-93 homolog B1
3	3	20.7	26.261	9.0212	Q9BVT8	Transmembrane and ubiquitin-like domain-containing protein 1
4	4	36.5	12.476	9.0109	P14174	Macrophage migration inhibitory factor
14	4	41.2	49.129	9.0029	O00148;O00148-2;O00148-3	ATP-dependent RNA helicase DDX39A
3	2	25.9	18.042	9.0022	P24666;P24666-4	Low molecular weight phosphotyrosine protein phosphatase
2	2	19.5	18.119	9.0015	P38936	Cyclin-dependent kinase inhibitor 1
7	3	18.6	60.221	9.0001	P52294	Importin subunit alpha-5;Importin subunit alpha-5, N-terminally processed
3	3	36.1	12.199	8.99	Q9NZ45	CDGSH iron-sulfur domain-containing protein 1
6	6	25.8	34.577	8.9883	O00487	26S proteasome non-ATPase regulatory subunit 14
4	4	12.7	55.527	8.9747	P48651;P48651-3;P48651-2	Phosphatidylserine synthase 1
6	6	11.8	67.256	8.9661	Q86TU7;Q86TU7-3;Q86TU7-2	Histone-lysine N-methyltransferase setd3
4	4	18.5	30.69	8.9652	Q8NG11;Q8NG11-2;Q8NG11-3	Tetraspanin-14
3	3	26.5	18.262	8.9583	P04179-3;P04179-4;P04179;P04179-2	Superoxide dismutase [Mn], mitochondrial
3	3	10.9	33.868	8.9562	Q9UBR2	Cathepsin Z
3	3	22.1	17.04	8.9518	P62841	40S ribosomal protein S15
4	4	21	23.51	8.9349	Q13257	Mitotic spindle assembly checkpoint protein MAD2A
5	3	17.3	43.448	8.9344	Q01085-2;Q01085;P31483-2	Nucleolysin TIAR;Nucleolysin TIA-1 isoform p40
6	6	10.5	92.273	8.9313	CON__ENSEMBL:ENSBTAP00000032840;sp|E32840|	
4	4	33.5	19.443	8.93	Q01658	Protein Dr1
2	2	15.8	20.419	8.9284	Q9NX76	CKLF-like MARVEL transmembrane domain-containing protein 6
2	2	5	50.126	8.9171	Q92685;Q92685-2	Dol-P-Man:Man(5)GlcNAc(2)-PP-Dol alpha-1,3-mannosyltransferase
9	9	30.6	42.403	8.9091	P50453	Serpin B9
6	6	12.4	81.242	8.8884	Q9Y4W2-2;Q9Y4W2;Q9Y4W2-3;Q9Y4W2-4	Ribosomal biogenesis protein LAS1L
6	6	13.9	64.293	8.8799	P43250-3;P43250;P43250-2	G protein-coupled receptor kinase 6
5	5	33.6	17.138	8.8736	P61088;Q5JXB2	Ubiquitin-conjugating enzyme E2 N;Putative ubiquitin-conjugating enzyme E2 N-like
2	2	10.4	21.482	8.865	Q99720-3;Q99720;Q99720-4;Q99720-5;Q99720-2	Sigma non-opioid intracellular receptor 1
5	5	54.6	13.281	8.8583	P62314	Small nuclear ribonucleoprotein Sm D1
5	5	13.3	57.579	8.8577	Q13217	DnaJ homolog subfamily C member 3
6	6	16.8	54.862	8.854	O94888	UBX domain-containing protein 7
3	3	14.8	37.413	8.8511	O43913-2;O43913	Origin recognition complex subunit 5
5	2	7	100.41	8.8489	O14787-2;O14787	Transportin-2
6	6	12.6	54.889	8.8385	P30566;P30566-2	Adenylosuccinate lyase
4	4	7.9	82.936	8.8312	P33897	ATP-binding cassette sub-family D member 1
4	4	5.1	138.35	8.8278	P29144	Tripeptidyl-peptidase 2
4	4	14	46.089	8.8241	Q9BY77;Q9BY77-2	Polymerase delta-interacting protein 3
5	5	33.3	17.853	8.8234	Q8N983-4;Q8N983-3;Q8N983-2;Q8N983;Q8N983-6;Q8N983-7	39S ribosomal protein L43, mitochondrial
4	4	18.4	54.389	8.8215	Q9NZM5	Glioma tumor suppressor candidate region gene 2 protein
6	6	4.7	196.44	8.8196	Q92614-2;Q92614-3;Q92614-4;Q92614;Q92614-5	Unconventional myosin-XVIIIa
5	5	11	71.607	8.8172	P43121	Cell surface glycoprotein MUC18
3	3	9	55.722	8.8005	P43007;P43007-2	Neutral amino acid transporter A

3	3	8	46.65	8.8004	P26440;P26440-2	Isovaleryl-CoA dehydrogenase, mitochondrial
4	4	12.2	55.446	8.7941	Q86Y07-3;Q86Y07;Q86Y07-4;Q86Y07-5;Q86Y07-2	Serine/threonine-protein kinase VRK2
3	3	25.6	16.832	8.794	O60888-3;O60888;O60888-2	Protein CutA
4	4	19.6	21.493	8.7925	Q00765	Receptor expression-enhancing protein 5
4	4	10.7	54.851	8.784	P78324-4;P78324;P78324-2;Q5TFQ8	Tyrosine-protein phosphatase non-receptor type substrate 1;Signal-regulatory protein beta-1 isoform 3
6	6	23	35.236	8.7529	Q9UNQ2	Probable dimethyladenosine transferase
3	3	12.9	45.168	8.7452	P17706-2;P17706-4;P17706;P17706-3	Tyrosine-protein phosphatase non-receptor type 2
1	1	2.6	68.932	8.7416	sp|Q32PI4|;CON__Q32PI4	
3	3	37.5	9.9743	8.7271	P60468	Protein transport protein Sec61 subunit beta
6	6	1.9	530.25	8.7225	Q9NR09	Baculoviral IAP repeat-containing protein 6
4	4	30	30.619	8.7031	P25942;P25942-2	Tumor necrosis factor receptor superfamily member 5
4	4	6.9	72.002	8.6848	Q03519-2;Q03519	Antigen peptide transporter 2
6	6	32.9	25.468	8.6796	P04183	Thymidine kinase, cytosolic
16	5	48.7	33.064	8.6749	P12235	ADP/ATP translocase 1
2	2	12.6	27.279	8.6654	O15120-2;O15120	1-acyl-sn-glycerol-3-phosphate acyltransferase beta
3	3	60.3	6.9151	8.6481	Q14061	Cytochrome c oxidase copper chaperone
6	0	13.2	67.355	8.648	sp|FA04-21b|;sp|FA04-21a|	
3	3	9.7	44.508	8.6422	Q8NEZ5;Q8NEZ5-3	F-box only protein 22
3	3	35.1	11.203	8.6245	P60903	Protein S100-A10
3	3	13.2	44.809	8.5897	P36941-2;P36941	Tumor necrosis factor receptor superfamily member 3
4	4	16.8	26.145	8.5845	P36543;P36543-2;P36543-3	V-type proton ATPase subunit E 1
2	2	32	8.7448	8.5784	Q8WUW1;Q8WUW1-2	Protein BRICK1
4	4	22.4	25.465	8.5682	Q00059-2;Q00059	Transcription factor A, mitochondrial
3	3	8.4	64.734	8.5613	P35610;P35610-3;P35610-2	Sterol O-acyltransferase 1
6	6	16.3	44.377	8.5376	Q9Y606-2;Q9Y606	tRNA pseudouridine synthase A, mitochondrial
2	2	9.1	35.198	8.5364	Q9Y385	Ubiquitin-conjugating enzyme E2 J1
4	3	17.6	31.263	8.5348	Q13243;Q13243-3;Q13243-2	Serine/arginine-rich splicing factor 5
7	7	21.1	52.295	8.5296	sp|Q3SZV7|;CON__Q3SZV7	
3	3	48.1	8.5328	8.4908	P50238	Cysteine-rich protein 1
5	5	11.2	52.672	8.4852	Q8IUF8-4;Q8IUF8;Q8IUF8-2	Bifunctional lysine-specific demethylase and histidyl-hydroxylase MINA
5	5	9.3	75.225	8.4701	Q9H089	Large subunit GTPase 1 homolog
5	5	39.1	21.85	8.4618	Q9NV31	U3 small nucleolar ribonucleoprotein protein IMP3
3	3	19.5	23.015	8.4493	P41236;Q6NXS1	Protein phosphatase inhibitor 2;Protein phosphatase inhibitor 2-like protein 3
8	8	18.2	64.521	8.4488	Q8NHH9-5;Q8NHH9-4;Q8NHH9-2;Q8NHH9;Q8NHH9-3	Atlastin-2
5	5	22.1	38.638	8.4442	Q6IN84	rRNA methyltransferase 1, mitochondrial
3	3	33.6	14.494	8.4375	P52758	Ribonuclease UK114
4	4	20.8	31.127	8.4329	Q9NUM4	Transmembrane protein 106B
5	5	52.7	17.143	8.4213	P52434;P52434-3;P52434-4	DNA-directed RNA polymerases I, II, and III subunit RPABC3
2	2	26.7	14.765	8.4135	Q96AB3-3;Q96AB3;Q96AB3-2	Isochorismatase domain-containing protein 2, mitochondrial
6	6	7.6	132.89	8.4135	Q53EP0;Q53EP0-2	Fibronectin type III domain-containing protein 3B
4	4	14.1	42.509	8.4128	Q16795	NADH dehydrogenase [ubiquinone] 1 alpha subcomplex subunit 9, mitochondrial
4	4	21.5	20.124	8.4049	P62913-2;P62913	60S ribosomal protein L11
3	3	15.3	29.952	8.3908	Q9P0I2;Q9P0I2-2	ER membrane protein complex subunit 3
4	4	26.9	22.629	8.3898	O60936;O60936-3;O60936-1	Nucleolar protein 3
7	6	16.5	66.398	8.3853	P11171-4;P11171-3;P11171-2;P11171;P11171-6;P11171-7;P11171-5	Protein 4.1
4	4	25.8	22.487	8.3708	Q92522	Histone H1x
5	5	13.8	56.077	8.3522	Q96F86	Enhancer of mRNA-decapping protein 3
4	4	8.9	53.486	8.3496	P11182	Lipoamide acyltransferase component of branched-chain alpha-keto acid dehydrogenase complex, mitochondrial

4	4	29	19.015	8.3413	Q13232	Nucleoside diphosphate kinase 3
4	4	13.7	42.835	8.3341	Q92597;Q92597-3;Q92597-2	Protein NDRG1
7	7	11.7	76.375	8.3282	O14964-2;O14964	Hepatocyte growth factor-regulated tyrosine kinase substrate
6	6	18.7	50.704	8.3107	P41240	Tyrosine-protein kinase CSK
4	1	23.3	31.761	8.3095	O00560-3;O00560	Syntenin-1
4	4	6	76.743	8.3044	Q92542-2;Q92542	Nicastrin
4	4	4.6	122.56	8.2815	Q14139;Q14139-2	Ubiquitin conjugation factor E4 A
5	5	9.8	73.011	8.2795	Q8WWI5-3;Q8WWI5-2;Q8WWI5	Choline transporter-like protein 1
9	9	9.4	114.08	8.2698	Q5XXA6;Q5XXA6-2;Q5XXA6-3	Anoctamin-1
8	8	25.5	42.996	8.2553	Q8NCH0	Carbohydrate sulfotransferase 14
5	2	11.3	60.873	8.2529	Q9BYJ9	YTH domain-containing family protein 1
4	4	20.8	26.383	8.2515	Q9NPD3	Exosome complex component RRP41
6	6	12.6	69.991	8.2479	Q14738;Q14738-3;Q14738-2	Serine/threonine-protein phosphatase 2A 56 kDa regulatory subunit delta isoform
5	5	5.3	152.66	8.2478	Q6YHK3-2;Q6YHK3-4;Q6YHK3	CD109 antigen
3	3	17.2	29.6	8.2441	Q9Y657;Q5JUX0	Spindlin-1;Spindlin-3
6	6	7.7	97.525	8.2346	Q7L7X3-3;Q7L7X3;Q7L7X3-2	Serine/threonine-protein kinase TAO1
2	2	15.2	18.092	8.2342	O60613;O60613-2	15 kDa selenoprotein
2	2	4.6	51.295	8.2316	Q06546	GA-binding protein alpha chain
2	2	19.4	15.68	8.2304	Q15714-2;Q15714-3;Q15714	TSC22 domain family protein 1
3	3	6.5	81.316	8.2276	P46199	Translation initiation factor IF-2, mitochondrial
7	7	12.4	79.443	8.2213	Q8NBF2;Q8NBF2-2	NHL repeat-containing protein 2
4	4	9.2	56.882	8.2206	Q9UHR4	Brain-specific angiogenesis inhibitor 1-associated protein 2-like protein 1
2	2	6	47.617	8.2145	Q6NXT4-4;Q6NXT4;Q6NXT4-2;Q6NXT4-3	Zinc transporter 6
5	5	28.1	20.834	8.2014	Q9NY12-2;Q9NY12	H/ACA ribonucleoprotein complex subunit 1
4	4	14.1	46.503	8.184	Q13510-2;Q13510;Q13510-3	Acid ceramidase;Acid ceramidase subunit alpha;Acid ceramidase subunit beta
5	3	30.7	23.48	8.1831	P10301	Ras-related protein R-Ras
5	5	5.3	149.78	8.1826	Q9NS87-2;Q9NS87;Q9NS87-4	Kinesin-like protein KIF15
6	3	6.7	144.25	8.1644	Q9UKE5-8;Q9UKE5-5;Q9UKE5-7;Q9UKE5-3;Q9UKE5-6;Q9UKE5-2;Q9UKE5-4;Q9UKE5	TRAF2 and NCK-interacting protein kinase
5	5	2.8	182.77	8.1598	Q8NI27	THO complex subunit 2
6	6	28.7	30.344	8.1483	O94903	Proline synthase co-transcribed bacterial homolog protein
3	3	23.3	22.345	8.1469	Q96IU4	Alpha/beta hydrolase domain-containing protein 14B
5	5	33.2	20.878	8.1465	P36404	ADP-ribosylation factor-like protein 2
4	4	9.6	75.275	8.1407	Q9UNK9	Protein angel homolog 1
2	2	12.1	23.598	8.1372	Q92785-2;Q92785	Zinc finger protein ubi-d4
4	4	15.5	33.479	8.1301	Q8TB36-2;Q8TB36	Ganglioside-induced differentiation-associated protein 1
5	5	9.9	72.275	8.124	Q9H078-3;Q9H078-2;Q9H078;Q9H078-5;Q9H078-4	Caseinolytic peptidase B protein homolog
2	2	27.7	9.3959	8.1182	P14406	Cytochrome c oxidase subunit 7A2, mitochondrial
7	7	20.5	57.603	8.1147	Q96D46	60S ribosomal export protein NMD3
2	2	18.6	21.405	8.1041	Q9BYC8	39S ribosomal protein L32, mitochondrial
5	4	5.9	116.58	8.094	P78504-2;P78504	Protein jagged-1
5	5	28.7	27.547	8.0919	O95336	6-phosphogluconolactonase
3	3	39.1	15.179	8.0797	O15116	U6 snRNA-associated Sm-like protein LSm1
20	1	22.6	134.19	8.0711	Q5JPE7-2;P69849;Q5JPE7;Q5JPE7-3	Nodal modulator 2;Nodal modulator 3
6	6	12.2	90.359	8.0693	Q9Y6A5	Transforming acidic coiled-coil-containing protein 3
6	6	25.9	34.596	8.0656	Q9P2T1-3;Q9P2T1;Q9P2T1-2	GMP reductase 2
5	5	26.2	32.113	8.0583	O00625	Pirin
8	8	12.9	80.702	8.058	Q13724-2;Q13724	Mannosyl-oligosaccharide glucosidase
2	2	29.9	10.688	8.057	P82921	28S ribosomal protein S21, mitochondrial
3	3	12.5	28.982	8.0524	Q86SK9-2;Q86SK9	Stearoyl-CoA desaturase 5

7	7	9.9	112.3	8.0482	Q9H6R4-2;Q9H6R4-4;Q9H6R4;Q9H6R4-3	Nucleolar protein 6
5	5	38	15.824	8.0466	Q7Z5G4;Q7Z5G4-3	Golgin subfamily A member 7
1	1	3.8	51.547	8.0449	Q6NXE6-2;Q6NXE6	Armadillo repeat-containing protein 6
6	5	35.2	28.466	8.0423	Q9UFN0	Protein NipSnap homolog 3A
3	3	22.2	21.198	8.037	Q6UW68	Transmembrane protein 205
4	4	16.4	42.961	8.0257	Q8IVS2	Malonyl-CoA-acyl carrier protein transacylase, mitochondrial
5	4	6.1	119.26	8.016	O60231	Putative pre-mRNA-splicing factor ATP-dependent RNA helicase DHX16
1	1	14.5	16.669	8.0131	Q6RW13-2;Q6RW13	Type-1 angiotensin II receptor-associated protein
2	2	31.3	10.921	8.0023	O43678;O43678-2	NADH dehydrogenase [ubiquinone] 1 alpha subcomplex subunit 2
6	6	26.2	42.794	7.9969	P51553;P51553-2	Isocitrate dehydrogenase [NAD] subunit gamma, mitochondrial
4	4	8.6	65.697	7.9806	Q8WX92;Q8WX92-2	Negative elongation factor B
6	6	28.1	35.351	7.9696	Q9BRJ2	39S ribosomal protein L45, mitochondrial
3	3	13.4	27.661	7.9649	P04156	Major prion protein
1	1	13	15.805	7.9601	Q9Y3C7	Mediator of RNA polymerase II transcription subunit 31
3	3	23.4	15.667	7.957	O43674-2;O43674	NADH dehydrogenase [ubiquinone] 1 beta subcomplex subunit 5, mitochondrial
11	5	22.8	59.834	7.9513	P12931;P12931-2	Proto-oncogene tyrosine-protein kinase Src
3	3	16.2	25.079	7.9507	Q9H8S9;Q7L9L4;Q7L9L4-2;Q9H8S9-2	MOB kinase activator 1A;MOB kinase activator 1B
3	3	14.8	35.25	7.9504	Q8NEJ9-2;Q8NEJ9	Neuroguidin
3	3	17.4	26.818	7.94	P27701-2;P27701	CD82 antigen
4	4	24.8	29.81	7.9397	O43819	Protein SCO2 homolog, mitochondrial
5	5	19.4	35.864	7.9259	Q9NT62;Q9NT62-2	Ubiquitin-like-conjugating enzyme ATG3
6	6	12.5	69.666	7.9239	Q14145	Kelch-like ECH-associated protein 1
7	6	19.9	54.099	7.9238	O43237;O43237-2	Cytoplasmic dynein 1 light intermediate chain 2
6	6	10.1	80.642	7.9168	Q96AQ6;Q96AQ6-2;Q96AQ6-3	Pre-B-cell leukemia transcription factor-interacting protein 1
2	2	4.4	81.871	7.915	O75175	CCR4-NOT transcription complex subunit 3
4	4	37.1	10.058	7.9086	O75531	Barrier-to-autointegration factor;Barrier-to-autointegration factor, N-terminally processed
3	3	21.4	24.95	7.8943	Q9H444	Charged multivesicular body protein 4b
3	3	12.4	29.45	7.8892	Q9HD33;Q9HD33-2	39S ribosomal protein L47, mitochondrial
6	6	10.5	93.378	7.8853	Q6PJF5-2;Q6PJF5	Inactive rhomboid protein 2
3	3	10.1	51.199	7.884	Q9UL15;Q9UL15-2	BAG family molecular chaperone regulator 5
6	4	17.7	56.501	7.8705	O95758-1;O95758-6;O95758-2;O95758;O95758-5;O95758-4;O95758-7	Polypyrimidine tract-binding protein 3
6	4	46.2	22.276	7.862	Q92688-2;Q92688	Acidic leucine-rich nuclear phosphoprotein 32 family member B
4	4	16.2	34.006	7.8565	A6NDG6	Phosphoglycolate phosphatase
5	5	16.9	42.441	7.8561	Q6RFH5;Q6RFH5-2	WD repeat-containing protein 74
3	3	7.3	80.684	7.8555	O60563	Cyclin-T1
7	7	25.5	35.206	7.8547	Q08257;Q08257-3;Q08257-2	Quinone oxidoreductase
5	5	30.9	15.48	7.8531	O60869-2;O60869-3;O60869	Endothelial differentiation-related factor 1
5	5	7.9	92.619	7.8045	P21127;Q9UQ88-5;Q9UQ88;P21127-2;P21127-3;Q9UQ88-2;Q9UQ88-3;P21127-8;Q9UQ88-4;P21127-9;P21127-6;P21127-10;P21127-5;P21127-4;Q9UQ88-10;P21127-12;Q9UQ88-9	Cyclin-dependent kinase 11B;Cyclin-dependent kinase 11A
1	1	13.7	14.71	7.8003	Q9NRG0	Chromatin accessibility complex protein 1
3	3	6.9	72.078	7.7988	Q8IW92	Beta-galactosidase-1-like protein 2
4	4	11.2	63.167	7.7867	Q9UNF1-2;Q9UNF1	Melanoma-associated antigen D2
5	5	13.6	52.118	7.7785	Q96TC7;Q96TC7-2	Regulator of microtubule dynamics protein 3
3	3	7.8	62.374	7.7763	Q6UXD5-4;Q6UXD5-6;Q6UXD5-2;Q6UXD5-3;Q6UXD5-5;Q6UXD5	Seizure 6-like protein 2
2	2	14.6	16.46	7.7758	P47813;O14602	Eukaryotic translation initiation factor 1A, X-chromosomal;Eukaryotic translation initiation factor 1A, Y-chromosomal
5	5	14.1	54.226	7.7579	Q9Y6V7	Probable ATP-dependent RNA helicase DDX49
4	4	8.5	71.685	7.7572	Q9HDC5	Junctophilin-1

6	6	17.2	42.515	7.7561	Q8TBM8;Q8TBM8-2	DnaJ homolog subfamily B member 14
3	3	6.4	86.573	7.7475	Q7L8L6	FAST kinase domain-containing protein 5
2	2	33.6	12.452	7.733	P63172	Dynein light chain Tctex-type 1
10	5	11.1	555.65	7.7307	P58107	Epiplakin
4	4	16.8	33.382	7.7156	O14562	Ubiquitin domain-containing protein UBFD1
6	4	17.7	46.404	7.7048	Q13363-2;Q13363	C-terminal-binding protein 1
1	1	15.6	19.642	7.6982	Q9BTX3	Transmembrane protein 208
6	6	26	32.558	7.6845	Q9H0P0-3;Q9H0P0-2;Q9H0P0-1;Q9H0P0	Cytosolic 5-nucleotidase 3A
3	3	16.1	26.071	7.6809	O14908-2;O14908	PDZ domain-containing protein GIPC1
3	3	23.2	13.742	7.6585	P62851	40S ribosomal protein S25
4	4	11.8	40.842	7.647	Q9Y2P8	RNA 3-terminal phosphate cyclase-like protein
4	4	9.4	80.379	7.6271	Q13144	Translation initiation factor eIF-2B subunit epsilon
2	1	27.5	8.5787	7.6228	sp|E38329|;CON__ENSEMBL:ENSBTAP00000038329	
3	3	9.1	53.846	7.6185	Q6UX04	Peptidyl-prolyl cis-trans isomerase CWC27 homolog
3	2	9.3	58.813	7.6162	Q9BTC8-2;Q9BTC8	Metastasis-associated protein MTA3
4	4	13.4	41.465	7.6159	P30533	Alpha-2-macroglobulin receptor-associated protein
7	5	3.7	246.75	7.6074	O75179-6;O75179-7;O75179-2;O75179	Ankyrin repeat domain-containing protein 17
4	4	18.7	36.836	7.6057	Q92600-2;Q92600;Q92600-3	Cell differentiation protein RCD1 homolog
4	4	3.6	140.7	7.6026	Q04912-7;Q04912-2;Q04912	Macrophage-stimulating protein receptor;Macrophage-stimulating protein receptor alpha chain;Macrophage-stimulating protein receptor beta chain
2	2	25.4	15.04	7.5932	Q15121;Q15121-2	Astrocytic phosphoprotein PEA-15
8	8	19.9	59.851	7.5929	O95394;O95394-3;O95394-4	Phosphoacetylglucosamine mutase
4	4	17.2	37.251	7.591	P48059;P48059-4;P48059-2;P48059-5;P48059-3;P0CW19-2	LIM and senescent cell antigen-like-containing domain protein 1
3	3	45.3	10.135	7.5856	Q9BWJ5	Splicing factor 3B subunit 5
5	5	16.7	41.896	7.5657	Q14BN4-5;Q14BN4-4;Q14BN4-2;Q14BN4-3;Q14BN4	Sarcolemmal membrane-associated protein
4	4	26.1	13.373	7.5595	P60866;P60866-2	40S ribosomal protein S20
4	4	16.4	36.946	7.5583	Q9Y673;Q9Y673-2	Dolichyl-phosphate beta-glucosyltransferase
3	3	33.1	15.434	7.5571	Q86WX3	Active regulator of SIRT1
4	4	9.6	69.156	7.5555	Q8IYS2;Q8IYS2-2	Uncharacterized protein KIAA2013
5	4	8.5	90.246	7.5425	Q6NSJ5	Volume-regulated anion channel subunit LRRC8E
3	3	11.7	39.837	7.5358	Q96S66-4;Q96S66-3;Q96S66-2;Q96S66	Chloride channel CLIC-like protein 1
2	2	22.9	13.475	7.5349	Q96BP2	Coiled-coil-helix-coiled-coil-helix domain-containing protein 1
10	10	15.1	89.594	7.5298	O94874;O94874-2	E3 UFM1-protein ligase 1
7	7	22.4	44.287	7.5278	O15382;O15382-2	Branched-chain-amino-acid aminotransferase, mitochondrial
3	3	19.6	22.876	7.5277	Q9NXU5	ADP-ribosylation factor-like protein 15
2	2	13.7	17.561	7.5182	O00422;O00422-2	Histone deacetylase complex subunit SAP18
3	3	31.7	15.996	7.5125	Q8N4Q1;Q8N4Q1-2	Mitochondrial intermembrane space import and assembly protein 40
6	6	28	36.085	7.5084	Q96KB5;Q96KB5-2	Lymphokine-activated killer T-cell-originated protein kinase
6	6	8	100.89	7.486	Q13206	Probable ATP-dependent RNA helicase DDX10
6	2	5.7	145.67	7.4695	Q96F07-2;Q96F07	Cytoplasmic FMR1-interacting protein 2
6	6	15.1	61.161	7.4522	Q9NZN4;Q9NZN4-2	EH domain-containing protein 2
3	3	14.9	31.585	7.4444	Q9NZD8-2;Q9NZD8	Maspardin
2	2	9.9	40.746	7.4366	P55039	Developmentally-regulated GTP-binding protein 2
6	6	5	158.55	7.4274	Q6PL18;Q6PL18-2	ATPase family AAA domain-containing protein 2
1	1	3	43.998	7.4263	P49441	Inositol polyphosphate 1-phosphatase
4	4	2.8	202.04	7.4257	Q9Y6D5;Q9Y6D6	Brefeldin A-inhibited guanine nucleotide-exchange protein 2;Brefeldin A-inhibited guanine nucleotide-exchange protein 1
5	5	7	104.66	7.4255	O14939-4;O14939	Phospholipase D2

8	8	18.5	65.173	7.4198	Q8WYA6;Q8WYA6-4;Q8WYA6-3;Q8WYA6-2	Beta-catenin-like protein 1
6	6	23.9	31.629	7.4188	P21912	Succinate dehydrogenase [ubiquinone] iron-sulfur subunit, mitochondrial
3	3	28	16.628	7.4171	Q86SX6	Glutaredoxin-related protein 5, mitochondrial
4	4	20.5	33.471	7.4063	Q9H974-2;Q9H974-3;Q9H974;Q9H974-4	Queuine tRNA-ribosyltransferase subunit QTRTD1
3	3	7.5	47.61	7.4	Q9H490-2;Q9H490	Phosphatidylinositol glycan anchor biosynthesis class U protein
2	2	11.6	32.369	7.3861	Q8NFF5-4;Q8NFF5-5;Q8NFF5-3;Q8NFF5-2;Q8NFF5	FAD synthase;Molybdenum cofactor biosynthesis protein-like region;FAD synthase region
3	3	28.9	15.139	7.3691	O60783	28S ribosomal protein S14, mitochondrial
2	2	14.6	20.546	7.3686	O15145	Actin-related protein 2/3 complex subunit 3
9	9	8.2	155.23	7.368	Q7Z478	ATP-dependent RNA helicase DHX29
6	6	17.2	54.151	7.36	Q9UI12-2;Q9UI12	V-type proton ATPase subunit H
5	5	7.2	84.918	7.3513	Q9Y3T9	Nucleolar complex protein 2 homolog
5	5	4.3	177.6	7.3492	P11047	Laminin subunit gamma-1
5	5	7.8	91.981	7.3392	Q9UKN8	General transcription factor 3C polypeptide 4
4	4	2.8	242.04	7.3326	O14497;O14497-2;O14497-3	AT-rich interactive domain-containing protein 1A
4	4	10.9	50.089	7.3266	O00220	Tumor necrosis factor receptor superfamily member 10A
5	5	6.7	107.17	7.3265	Q8TD19	Serine/threonine-protein kinase Nek9
4	4	45.8	15.649	7.321	P26885	Peptidyl-prolyl cis-trans isomerase FKBP2
4	4	15.5	46.237	7.3169	Q96GC9;Q96GC9-2	Vacuole membrane protein 1
5	5	12.4	68.66	7.306	P36915	Guanine nucleotide-binding protein-like 1
6	6	15.2	44.424	7.3047	P36507	Dual specificity mitogen-activated protein kinase kinase 2
4	4	19.4	28.97	7.3038	O95721	Synaptosomal-associated protein 29
6	6	9.6	88.234	7.3034	Q9UQ90;Q9UQ90-2	Paraplegin
3	3	34.9	12.49	7.3005	Q7Z7K0	COX assembly mitochondrial protein homolog
5	5	18.8	41.738	7.2887	P53367;P53367-2	Arfaptin-1
1	1	6	30.337	7.2885	P62079	Tetraspanin-5
3	3	4.1	114.05	7.2863	Q6WCQ1-3;Q6WCQ1;Q6WCQ1-2	Myosin phosphatase Rho-interacting protein
3	3	13.7	25.206	7.2857	Q13445	Transmembrane emp24 domain-containing protein 1
2	2	12.5	20.733	7.2825	Q96HR9-2;Q96HR9	Receptor expression-enhancing protein 6
2	2	23.7	16.516	7.2797	O14880	Microsomal glutathione S-transferase 3
4	4	9.2	55.25	7.2795	O15321-2;O15321	Transmembrane 9 superfamily member 1
1	1	2.6	61.317	7.2715	Q8ND24-2;Q8ND24	RING finger protein 214
3	3	9.2	48.868	7.2611	Q6NT16	MFS-type transporter SLC18B1
5	5	10	54.283	7.2428	Q9Y6W5;sp|WAVE2mur|	Wiskott-Aldrich syndrome protein family member 2
4	4	17.4	32.805	7.2402	Q53H82	Beta-lactamase-like protein 2
3	3	34.2	14.026	7.2371	O95168-2;O95168	NADH dehydrogenase [ubiquinone] 1 beta subcomplex subunit 4
2	2	23.5	17.201	7.228	Q9NX24	H/ACA ribonucleoprotein complex subunit 2
3	3	13.5	35.612	7.2254	O75787-2;O75787	Renin receptor
2	2	9.1	46.306	7.2174	Q9NZ32	Actin-related protein 10
5	5	17.1	45.558	7.2155	Q8N2K0-2;Q8N2K0;Q8N2K0-3	Monoacylglycerol lipase ABHD12
4	4	18	35.023	7.2056	Q96BJ3;Q96BJ3-3	Axin interactor, dorsalization-associated protein
2	2	16.6	19.621	7.2044	Q9UHA3	Probable ribosome biogenesis protein RLP24
7	4	13	83.735	7.1987	P51812	Ribosomal protein S6 kinase alpha-3
4	4	13.9	50.435	7.195	Q07960	Rho GTPase-activating protein 1
2	2	5.6	85.652	7.1885	Q9ULW0;Q9ULW0-2	Targeting protein for Xklp2
2	2	5.3	57.276	7.1845	Q9H3P2	Negative elongation factor A
5	5	14.2	50.927	7.1761	Q5T3I0-3;Q5T3I0	G patch domain-containing protein 4
2	2	16.9	18.048	7.1694	Q9NWV4	UPF0587 protein C1orf123
4	4	38.2	13.941	7.1678	Q9BRA2	Thioredoxin domain-containing protein 17
7	7	17.7	43.944	7.1673	Q99816	Tumor susceptibility gene 101 protein
4	4	8.8	56.72	7.1665	Q8N6H7;Q8N6H7-2	ADP-ribosylation factor GTPase-activating protein 2

7	7	7.1	108.63	7.1658	Q8WX93-3;Q8WX93;Q8WX93-4;Q8WX93-8;Q8WX93-5;Q8WX93-2;Q8WX93-9;Q8WX93-7	Palladin
4	4	29.4	21.635	7.164	P00568	Adenylate kinase isoenzyme 1
3	3	19.9	31.576	7.1636	Q29983-2;Q29983	MHC class I polypeptide-related sequence A
2	2	3.1	97.356	7.1631	Q9H330;Q9H330-3;Q9H330-4	Transmembrane protein 245
5	5	23.2	33.269	7.1602	P49247	Ribose-5-phosphate isomerase
4	4	8.7	79.322	7.1581	Q5SY16	Polynucleotide 5-hydroxyl-kinase NOL9
7	1	14.6	47.754	7.1406	sp|P08730-1|;CON__P08730-1	
3	3	26.2	15.947	7.14	Q6P1L8	39S ribosomal protein L14, mitochondrial
3	1	3.7	125.09	7.137	A8CG34;Q96HA1-2;A8CG34-2;Q96HA1-3	Nuclear envelope pore membrane protein POM 121C;Nuclear envelope pore membrane protein POM 121
3	3	17.6	20.505	7.1296	Q9UBQ0;Q9UBQ0-2	Vacuolar protein sorting-associated protein 29
3	3	17	30.039	7.1271	Q96B26	Exosome complex component RRP43
1	1	5.9	49.485	7.1152	Q676U5-5;Q676U5-3;Q676U5-2;Q676U5	Autophagy-related protein 16-1
2	2	7	40.214	7.0991	Q8N357	Solute carrier family 35 member F6
3	1	7.5	51.724	7.0981	P36896-5;P36896-2;P36896-3;P36896;P36896-4;Q8NER5-3;P36897-3;Q8NER5-4;Q8NER5	Activin receptor type-1B;Activin receptor type-1C;TGF-beta receptor type-1
1	1	5.2	26.352	7.0809	Q15125	3-beta-hydroxysteroid-Delta(8),Delta(7)-isomerase
3	2	11.3	39.453	7.079	Q5VT66-2;Q5VT66;Q5VT66-3	Mitochondrial amidoxime-reducing component 1
2	2	8.6	36.504	7.0788	Q7L5D6;Q7L5D6-2	Golgi to ER traffic protein 4 homolog
4	4	20.1	28.228	7.0776	Q9UIV1-2;Q9UIV1	CCR4-NOT transcription complex subunit 7
6	6	6.9	129.54	7.0743	Q8IY37	Probable ATP-dependent RNA helicase DHX37
5	5	7.6	98.795	7.0653	Q9H501	ESF1 homolog
5	5	29.2	29.816	7.0638	Q92530	Proteasome inhibitor PI31 subunit
3	3	10.3	45.78	7.0576	Q9Y3I1-3;Q9Y3I1-2;Q9Y3I1	F-box only protein 7
5	5	13.7	60.246	7.0537	Q13564;Q13564-4;Q13564-3;Q13564-2	NEDD8-activating enzyme E1 regulatory subunit
7	7	11.7	77.394	7.0515	Q8TC07-2;Q8TC07-3;Q8TC07	TBC1 domain family member 15
7	5	17	54.114	7.0503	P49590-2;P49590	Probable histidine--tRNA ligase, mitochondrial
4	4	13.1	43.064	7.0436	Q96S59-2;Q96S59-3;Q96S59	Ran-binding protein 9
6	6	14.9	50.519	7.0433	Q9HD26;Q9HD26-2;Q9HD26-3	Golgi-associated PDZ and coiled-coil motif-containing protein
5	5	28	31.441	7.0382	P19387	DNA-directed RNA polymerase II subunit RPB3
6	6	19.2	48.166	7.038	Q92990-2;Q92990	Glomulin
5	5	24.6	26.365	7.0347	P15559-3;P15559-2;P15559	NAD(P)H dehydrogenase [quinone] 1
4	4	23.9	24.211	7.0323	Q9BYN8	28S ribosomal protein S26, mitochondrial
1	1	25.8	10.679	7.0282	Q0VAQ4	Small cell adhesion glycoprotein
3	3	6.6	96.723	7.0226	Q9UPN7	Serine/threonine-protein phosphatase 6 regulatory subunit 1
3	3	14.8	27.775	7.0193	Q6UX53	Methyltransferase-like protein 7B
1	1	13	21.386	7.0135	Q96FX8	p53 apoptosis effector related to PMP-22
6	3	22.4	37.77	7.0062	Q9P289-2;Q9P289;O00506-3;O00506-2;Q9P289-3;O00506	Serine/threonine-protein kinase 26;Serine/threonine-protein kinase 25
3	3	6.7	69.574	7.0018	O15254-2;O15254	Peroxisomal acyl-coenzyme A oxidase 3
3	3	2.2	200.21	6.9973	Q8IWV7	E3 ubiquitin-protein ligase UBR1
3	3	9.8	46.63	6.9934	sp|FA74-18a|;Q86VE9-3;Q86VE9-2;Q86VE9;Q86VE9-4;sp|FA74-18b|AAI01284.1	Serine incorporator 5
4	4	17	24.81	6.9593	O43760;O43760-2	Synaptogyrin-2
4	4	6.7	78.983	6.9514	Q9NUQ8-2;Q9NUQ8	ATP-binding cassette sub-family F member 3
7	7	7.2	131.04	6.9441	Q8IX12-2;Q8IX12	Cell division cycle and apoptosis regulator protein 1
3	3	14.8	23.881	6.9395	Q8TED1	Probable glutathione peroxidase 8
3	3	6.2	67.703	6.9343	sp|E05568|;CON__ENSEMBL:ENSBTAP00000023055;Q04756	Hepatocyte growth factor activator;Hepatocyte growth factor activator short chain;Hepatocyte growth factor activator long chain
5	5	6.7	119.16	6.934	P52732	Kinesin-like protein KIF11
2	2	10.5	26.66	6.933	P19256-2;P19256-3;P19256	Lymphocyte function-associated antigen 3

4	4	6	92.203	6.9278	Q53R41-2;Q53R41	FAST kinase domain-containing protein 1
4	4	25.7	26.411	6.927	Q16563-2;Q16563	Synaptophysin-like protein 1
1	1	2.7	50.364	6.9253	Q9NP92	28S ribosomal protein S30, mitochondrial
4	4	6.8	76.837	6.9201	Q96D71-2;Q96D71-4;Q96D71-3;Q96D71	RalBP1-associated Eps domain-containing protein 1
7	7	11.5	90.59	6.9173	Q9Y2G8;Q9Y2G8-2	DnaJ homolog subfamily C member 16
3	3	13.2	40.816	6.9166	Q9UJA5-3;Q9UJA5-2;Q9UJA5	tRNA (adenine(58)-N(1))-methyltransferase non-catalytic subunit TRM6
33	2	61.8	58.061	6.9081	P14618-2	Pyruvate kinase PKM
2	2	14.9	18.154	6.8972	Q9H2H8;Q9H2H8-2	Peptidyl-prolyl cis-trans isomerase-like 3
4	4	32.4	16.713	6.8829	P60983	Glia maturation factor beta
4	4	37.7	11.731	6.8696	Q9Y2R0	Cytochrome c oxidase assembly factor 3 homolog, mitochondrial
3	3	19.5	22.875	6.8683	Q9Y5Z4;Q9Y5Z4-2	Heme-binding protein 2
2	2	14.5	18.01	6.8619	Q9UDX5;Q9UDX5-2	Mitochondrial fission process protein 1
4	4	10.3	55.435	6.8425	Q7Z3B4;Q7Z3B4-3;Q7Z3B4-2	Nucleoporin p54
5	5	17.5	35.158	6.8118	Q8NE86-3;Q8NE86;Q8NE86-2	Calcium uniporter protein, mitochondrial
5	5	19.8	54.234	6.8101	Q9BR76	Coronin-1B
3	3	10.7	42.272	6.8076	P51570;P51570-2	Galactokinase
3	3	10.6	49.917	6.8013	Q15750-2;Q15750	TGF-beta-activated kinase 1 and MAP3K7-binding protein 1
4	4	6	92.067	6.791	Q99523;Q99523-2	Sortilin
7	1	33.6	32.853	6.7898	P04181-2	Ornithine aminotransferase, mitochondrial;Ornithine aminotransferase, hepatic form;Ornithine aminotransferase, renal form
6	6	12.7	59.058	6.7878	Q99829	Copine-1
3	3	9.7	55.148	6.781	P43003-2;P43003	Excitatory amino acid transporter 1
3	3	17.8	20.313	6.7777	P61009	Signal peptidase complex subunit 3
3	3	30.7	19.181	6.775	Q8WUD4	Coiled-coil domain-containing protein 12
2	2	32.4	11.845	6.7696	P62310	U6 snRNA-associated Sm-like protein LSm3
2	2	19.7	17.174	6.7573	P62256-2;P62256	Ubiquitin-conjugating enzyme E2 H
5	5	16.7	41.968	6.7435	Q9GZT8;Q9GZT8-2;Q9GZT8-3	NIF3-like protein 1
4	4	20.1	25.357	6.7378	P28072	Proteasome subunit beta type-6
2	2	6.4	49.395	6.731	Q86V85	Integral membrane protein GPR180
4	4	20.1	32.818	6.7128	P09601	Heme oxygenase 1
2	2	6.3	54.508	6.7121	Q9BVS4-2;Q9BVS4	Serine/threonine-protein kinase RIO2
2	2	32.5	8.4426	6.7017	Q8NHG7	Small VCP/p97-interacting protein
2	2	17.4	21.494	6.694	Q8WZA0;Q8WZA0-2	Protein LZIC
3	3	17.5	25.627	6.6935	Q53RY4;Q53RY4-2	Keratinocyte-associated protein 3
2	2	7.3	41.945	6.6911	Q9P0J7	E3 ubiquitin-protein ligase KCMF1
4	4	7.1	89.702	6.69	P18433-6;P18433	Receptor-type tyrosine-protein phosphatase alpha
2	2	3.1	108.53	6.6886	P12109	Collagen alpha-1(VI) chain
2	2	5.3	55.699	6.688	Q96BP3-2;Q96BP3	Peptidylprolyl isomerase domain and WD repeat-containing protein 1
2	2	22.7	11.471	6.686	Q6ZSJ8	Uncharacterized protein C1orf122
3	1	15.7	22.367	6.6726	Q9NR31	GTP-binding protein SAR1a
2	2	11.4	20.266	6.6462	Q9P032	NADH dehydrogenase [ubiquinone] 1 alpha subcomplex assembly factor 4
5	5	11.3	55.207	6.6462	sp|E18574|;CON__ENSEMBL:ENSBTAP00000018574	
3	3	8.6	59.33	6.639	Q9NPI6-2;Q9NPI6	mRNA-decapping enzyme 1A
4	4	15.5	40.574	6.6354	Q96FZ2	Embryonic stem cell-specific 5-hydroxymethylcytosine-binding protein
3	3	20.1	30.354	6.6335	Q15287-3;Q15287-2;Q15287	RNA-binding protein with serine-rich domain 1
3	3	8.7	63.245	6.6148	Q8N543;Q8N543-2	Prolyl 3-hydroxylase OGFOD1
5	5	8.9	73.918	6.6082	Q9UBH6-2;Q9UBH6	Xenotropic and polytropic retrovirus receptor 1
2	2	40	10.834	6.6025	Q9Y333	U6 snRNA-associated Sm-like protein LSm2
5	5	41.6	11.728	6.6013	P26447	Protein S100-A4
7	7	9	112.53	6.6011	Q9UI26;Q9UI26-2	Importin-11

3	3	18.4	22.999	6.5986	P82664	28S ribosomal protein S10, mitochondrial
5	5	9.2	78.695	6.5925	Q96KG9-3;Q96KG9-4;Q96KG9-2;Q96KG9;Q96KG9-5;Q96KG9-6	N-terminal kinase-like protein
2	2	10.2	29.622	6.5806	Q9H9Q2;Q9H9Q2-2;Q9H9Q2-3	COP9 signalosome complex subunit 7b
3	1	18.4	22.255	6.5797	Q96LR5;Q969T4	Ubiquitin-conjugating enzyme E2 E2;Ubiquitin-conjugating enzyme E2 E3
4	4	18.4	36.707	6.5654	Q8NFH4	Nucleoporin Nup37
4	4	7	80.551	6.5631	Q92615	La-related protein 4B
3	3	1.4	527.22	6.56	O95714	E3 ubiquitin-protein ligase HERC2
3	3	9.6	37.205	6.5529	Q9H3K2	Growth hormone-inducible transmembrane protein
6	6	6.9	129.75	6.5513	P98196	Probable phospholipid-transporting ATPase IH
5	5	14.4	47.776	6.5471	Q8IYS1	Peptidase M20 domain-containing protein 2
4	4	11.1	39.682	6.5432	Q15629-2;Q15629	Translocating chain-associated membrane protein 1
3	3	19.4	21.65	6.5423	Q8WUY8	N-acetyltransferase 14
3	3	20	20.199	6.5404	Q9BUR5-2;Q9BUR5	Apolipoprotein O
3	3	13.9	31.978	6.5381	Q15526-2;Q15526	Surfeit locus protein 1
4	4	12.4	53.32	6.5371	Q9NVX2;Q9NVX2-2	Notchless protein homolog 1
3	3	5.8	86.359	6.5222	Q9H1I8;Q9H1I8-3	Activating signal cointegrator 1 complex subunit 2
2	2	26.6	16.758	6.5167	Q96MW1-2;Q96MW1	Coiled-coil domain-containing protein 43
9	5	22.4	39.586	6.5163	Q13247;Q13247-3	Serine/arginine-rich splicing factor 6
5	5	4.1	177.35	6.4873	Q6XZF7	Dynamin-binding protein
4	4	9.3	71.026	6.4858	Q9H0H5	Rac GTPase-activating protein 1
2	2	7	35.83	6.4791	Q6PCB6;Q6PCB6-2	Alpha/beta hydrolase domain-containing protein 17C
4	4	8	71.695	6.4622	A6NDB9	Paralemmin-3
4	4	5.4	103.88	6.4618	Q6ZXV5-2;Q6ZXV5	Transmembrane and TPR repeat-containing protein 3
3	3	26.1	16.941	6.4603	Q9BPX5	Actin-related protein 2/3 complex subunit 5-like protein
3	3	5.6	70.454	6.4596	Q96BD0-3;Q96BD0-4;Q96BD0	Solute carrier organic anion transporter family member 4A1
4	4	31	15.928	6.4541	P57105	Synaptojanin-2-binding protein
2	2	5.3	73.003	6.45	O60476	Mannosyl-oligosaccharide 1,2-alpha-mannosidase IB
3	3	5.3	88.028	6.4352	Q15057	Arf-GAP with coiled-coil, ANK repeat and PH domain-containing protein 2
4	4	19.9	30.707	6.4257	Q14790-8;Q14790-7;Q14790-2;Q14790;Q14790-4;Q14790-9;Q14790-6;Q14790-5;Q14790-3	Caspase-8;Caspase-8 subunit p18;Caspase-8 subunit p10
4	4	19.3	30.276	6.4251	Q9UBW8	COP9 signalosome complex subunit 7a
4	4	24.1	29.841	6.4184	O75937	DnaJ homolog subfamily C member 8
3	3	13.5	36.567	6.4175	Q96IZ0	PRKC apoptosis WT1 regulator protein
3	3	11.9	46.291	6.4061	Q96IJ6;Q96IJ6-2	Mannose-1-phosphate guanyltransferase alpha
2	2	25	10.803	6.405	P62304	Small nuclear ribonucleoprotein E
3	3	12.4	35.759	6.4007	Q8IXK0-2;Q8IXK0-4;Q8IXK0;Q8IXK0-5;Q8IXK0-3	Polyhomeotic-like protein 2
3	3	8.8	51.417	6.396	O95674	Phosphatidate cytidylyltransferase 2
7	1	27.4	38.405	6.3684	Q9NQ29-2;Q9NQ29	Putative RNA-binding protein Luc7-like 1
5	5	13.7	51.017	6.3617	Q96GQ5	RUS1 family protein C16orf58
5	5	33.7	20.214	6.3606	O75663-2;O75663	TIP41-like protein
6	1	8.9	58.264	6.3596	Q58FF6	Putative heat shock protein HSP 90-beta 4
4	4	10.9	57.028	6.355	Q16222-2;Q16222-3;Q16222	UDP-N-acetylhexosamine pyrophosphorylase;UDP-N-acetylgalactosamine pyrophosphorylase;UDP-N-acetylglucosamine pyrophosphorylase
4	4	36.2	6.9611	6.3541	O75438;O75438-2	NADH dehydrogenase [ubiquinone] 1 beta subcomplex subunit 1
5	4	40.2	15.257	6.3496	Q16629-3;Q16629-2;Q16629-4;Q16629	Serine/arginine-rich splicing factor 7
2	2	6.7	54.351	6.3447	Q9BPX6;Q9BPX6-3;Q9BPX6-5;Q9BPX6-4;Q9BPX6-2	Calcium uptake protein 1, mitochondrial
4	4	5.6	121.17	6.3402	Q9Y6K5	2-5-oligoadenylate synthase 3
4	4	14.2	46.43	6.3371	Q4ZIN3-2;Q4ZIN3	Membralin
3	3	30.5	16.32	6.32	O15511;O15511-2	Actin-related protein 2/3 complex subunit 5

6	6	23	33.249	6.3179	Q9Y399	28S ribosomal protein S2, mitochondrial
4	4	11.7	44.297	6.3078	P16219	Short-chain specific acyl-CoA dehydrogenase, mitochondrial
2	2	4.1	80.709	6.2853	Q9BVQ7;Q9BVQ7-2;Q9BVQ7-3	Spermatogenesis-associated protein 5-like protein 1
5	5	58.6	10.044	6.2707	P07108;P07108-3;P07108-2;P07108-4;P07108-5;P07108-6	Acyl-CoA-binding protein
3	1	8.8	50.82	6.265	Q9Y3L3-2;Q9Y3L3	SH3 domain-binding protein 1
3	3	44.3	10.771	6.2634	Q96GX2	Putative ataxin-7-like protein 3B
3	3	7.9	52.12	6.2591	Q8NHP6-2;Q8NHP6	Motile sperm domain-containing protein 2
2	2	13.7	17.49	6.2587	P30049	ATP synthase subunit delta, mitochondrial
3	3	9.3	49.255	6.244	Q15428	Splicing factor 3A subunit 2
2	2	5.9	61.719	6.2364	P30038;P30038-2;P30038-3	Delta-1-pyrroline-5-carboxylate dehydrogenase, mitochondrial
3	3	34.5	16.311	6.2253	O15514	DNA-directed RNA polymerase II subunit RPB4
3	3	18.9	22.836	6.2091	P49721	Proteasome subunit beta type-2
4	1	39.3	9.461	6.2051	P42677	40S ribosomal protein S27
2	2	9.5	33.428	6.2033	P53004	Biliverdin reductase A
5	5	16.3	47.036	6.2028	P12532;P12532-2;P17540	Creatine kinase U-type, mitochondrial;Creatine kinase S-type, mitochondrial
3	3	51.2	10.116	6.2022	Q9H3K6;Q9H3K6-2	BolA-like protein 2
4	4	10.4	39.803	6.1875	P15529-7;P15529-4;P15529-6;P15529-3;P15529-5;P15529-2;P15529-15;P15529-9;P15529-12;P15529-14;P15529-8;P15529-11;P15529-13;P15529-10;P15529;P15529-16	Membrane cofactor protein
2	2	18	23.449	6.1873	P55789	FAD-linked sulfhydryl oxidase ALR
5	3	19.5	37.839	6.186	O94905	Erlin-2
2	2	25.2	15.672	6.1859	P00374-2;P00374	Dihydrofolate reductase
2	2	28.6	13.428	6.1856	O15305-2;O15305	Phosphomannomutase 2
8	8	1.4	796.43	6.1744	Q8WXH0;Q8WXH0-2	Nesprin-2
4	4	11.6	51.459	6.1679	Q8N2G8-2;Q8N2G8-3;Q8N2G8	GH3 domain-containing protein
7	7	4.9	214.82	6.1586	A3KMH1;A3KMH1-3;A3KMH1-2	von Willebrand factor A domain-containing protein 8
5	3	26.5	24.267	6.1557	O95716	Ras-related protein Rab-3D
6	4	8.3	92.448	6.154	Q8TDW0	Volume-regulated anion channel subunit LRRC8C
5	5	6.4	145.29	6.1416	Q7Z3K3-5;Q7Z3K3-7;Q7Z3K3-2;Q7Z3K3-3;Q7Z3K3-6;Q7Z3K3	Pogo transposable element with ZNF domain
3	3	17.2	25.565	6.1393	Q9UIJ7;Q9UIJ7-3;Q9UIJ7-2	GTP:AMP phosphotransferase AK3, mitochondrial
6	6	36.4	16.953	6.132	Q56VL3	OCIA domain-containing protein 2
3	3	2.5	158.54	6.1212	O75882;O75882-3;O75882-2	Attractin
4	4	6.9	88.813	6.1097	Q9BWU0	Kanadaptin
2	2	6.8	41.597	6.092	Q8WW22-3;Q8WW22;Q8WW22-2	DnaJ homolog subfamily A member 4
1	1	4.8	29.73	6.0898	Q7Z7K6-3;Q7Z7K6	Centromere protein V
3	3	15.2	23.705	6.0842	O00217	NADH dehydrogenase [ubiquinone] iron-sulfur protein 8, mitochondrial
3	3	9.1	26.21	6.0675	Q9BVC6	Transmembrane protein 109
5	1	4.9	143.64	6.0631	Q8N4C8-5;Q8N4C8-2;Q8N4C8-3;Q8N4C8-4;Q8N4C8	Misshapen-like kinase 1
5	5	8.9	69.561	6.0546	sp|585019|;CON__REFSEQ:XP_585019	
3	3	31.9	18.243	6.0543	Q13526	Peptidyl-prolyl cis-trans isomerase NIMA-interacting 1
2	2	22	13.757	6.0515	O75348;O95670-2;O95670	V-type proton ATPase subunit G 1;V-type proton ATPase subunit G 2
2	2	12.8	27.125	6.0494	Q96GX9;Q96GX9-3	Methylthioribulose-1-phosphate dehydratase
2	2	4.7	59.988	6.0351	P15586-2;P15586	N-acetylglucosamine-6-sulfatase
3	3	8	65.009	6.0308	Q9H4L4	Sentrin-specific protease 3
2	2	8.3	44.287	6.0285	P45985;P45985-2	Dual specificity mitogen-activated protein kinase kinase 4
3	2	15.9	24.984	6.0269	P20336	Ras-related protein Rab-3A
3	3	36.9	11.466	6.024	P82909	28S ribosomal protein S36, mitochondrial

2	2	25.8	17.663	6.0228	Q9Y3D0	Mitotic spindle-associated MMXD complex subunit MIP18
2	2	15.6	30.062	6.0117	Q9NQ88	Fructose-2,6-bisphosphatase TIGAR
6	6	13.6	71.277	6.0089	Q9Y223-4;Q9Y223-3;Q9Y223;Q9Y223-2;Q9Y223-5	Bifunctional UDP-N-acetylglucosamine 2-epimerase/N-acetylmannosamine kinase;UDP-N-acetylglucosamine 2-epimerase (hydrolyzing);N-acetylmannosamine kinase
3	3	15.3	32.009	6.0055	Q9H467	CUE domain-containing protein 2
1	1	9.9	14.428	5.9952	O95214;O95214-2	Leptin receptor overlapping transcript-like 1
3	3	6	101.55	5.9937	Q8NEB9	Phosphatidylinositol 3-kinase catalytic subunit type 3
3	3	28.4	18.291	5.9887	P51636;P51636-2	Caveolin-2
3	3	9.9	50.813	5.9846	Q92485;Q92485-2	Acid sphingomyelinase-like phosphodiesterase 3b
1	1	9.9	12.746	5.9748	P61599-2;P61599	N-alpha-acetyltransferase 20
4	4	11.1	59.593	5.9737	Q15554;Q15554-4	Telomeric repeat-binding factor 2
6	6	5.8	149.04	5.9719	Q8N122;Q8N122-3	Regulatory-associated protein of mTOR
4	4	26	19.806	5.9713	Q9NZ72-2;Q9NZ72	Stathmin-3
4	4	10.3	69.984	5.9703	P35611-2;P35611-6;P35611-4;P35611;P35611-3;P35611-5	Alpha-adducin
1	1	15.4	14.874	5.9622	O95295	SNARE-associated protein Snapin
3	2	3.2	132.32	5.9612	Q8NDI1-3;Q8NDI1-2;Q8NDI1	EH domain-binding protein 1
5	5	9.7	108.13	5.9564	P21709;P21709-3;P21709-2	Ephrin type-A receptor 1
5	4	7.1	129.23	5.9551	Q9Y219-2;Q9Y219	Protein jagged-2
5	5	8.1	89.13	5.9503	O75152	Zinc finger CCCH domain-containing protein 11A
3	3	6	76.177	5.9497	Q8N5G2;Q8N5G2-2;Q8N5G2-3	Macoilin
4	3	3.1	162.11	5.9459	Q7Z460-2;Q7Z460-4;Q7Z460-5;Q7Z460-3;Q7Z460	CLIP-associating protein 1
3	3	17.2	29.988	5.945	Q9NVS9;Q9NVS9-3;Q9NVS9-4	Pyridoxine-5-phosphate oxidase
3	3	14.3	37.98	5.945	Q15165-3;Q15165-1;Q15165	Serum paraoxonase/arylesterase 2
4	4	17.5	36.899	5.944	Q9NQG5	Regulation of nuclear pre-mRNA domain-containing protein 1B
2	2	8.5	45.479	5.9409	Q9BTE6;Q9BTE6-2;Q9BTE6-3	Alanyl-tRNA editing protein Aarsd1
3	3	4.9	93.908	5.9405	Q658Y4	Protein FAM91A1
5	4	7.7	97.903	5.9399	Q8NB90;Q8NB90-2;Q8NB90-3	Spermatogenesis-associated protein 5
7	7	3.9	254.39	5.9297	O60287	Nucleolar pre-ribosomal-associated protein 1
10	0	14.4	76.24	5.9195	sp|FA23-17-Human-HspA6|;P17066;P48741	Heat shock 70 kDa protein 6;Putative heat shock 70 kDa protein 7
3	3	15.2	27.508	5.9172	Q9BTE7	DCN1-like protein 5
3	3	14.7	31.832	5.9167	Q9HCN4-3;Q9HCN4-2;Q9HCN4-4;Q9HCN4;Q9HCN4-5	GPN-loop GTPase 1
1	1	1.2	105.02	5.9165	P98171;P98171-2	Rho GTPase-activating protein 4
4	4	20.8	38.197	5.9158	Q12893	Transmembrane protein 115
4	4	8.1	67.152	5.9116	Q8WXI4-2;Q8WXI4	Acyl-coenzyme A thioesterase 11
4	4	7.6	76.967	5.9045	Q9H9A5-4;Q9H9A5-3;Q9H9A5;Q9H9A5-6;Q9H9A5-2	CCR4-NOT transcription complex subunit 10
5	5	5.8	129.39	5.901	sp|Q28194|;CON__Q28194;P07996-2;P07996	Thrombospondin-1
3	3	26.8	18.129	5.8986	Q07812-5;Q07812-7;Q07812-8;Q07812;Q07812-2	Apoptosis regulator BAX
2	2	8.2	36.382	5.8958	Q96EU7	C1GALT1-specific chaperone 1
4	4	6.9	87.198	5.8957	Q4KMP7	TBC1 domain family member 10B
2	2	17	17.394	5.8925	P09234	U1 small nuclear ribonucleoprotein C
4	4	2.1	309.22	5.886	O95071-2;O95071	E3 ubiquitin-protein ligase UBR5
7	7	15.6	67.653	5.8776	Q4G0J3-3;Q4G0J3	La-related protein 7
5	5	7.5	88.8	5.8776	Q14156-2;Q14156;Q14156-3	Protein EFR3 homolog A
1	1	12.6	20.625	5.8699	Q96LW7-2	
2	2	11.2	27.135	5.8686	Q16637-4;Q16637-2;Q16637-3;Q16637;sp|P97801|SMN_MOUSE	Survival motor neuron protein

1	1	14.4	10.333	5.8566	P62072	Mitochondrial import inner membrane translocase subunit Tim10
4	4	15.2	29.217	5.8512	Q9HC21-2;Q9HC21	Mitochondrial thiamine pyrophosphate carrier
2	2	6.7	43.261	5.8499	O60870-2;O60870	DNA/RNA-binding protein KIN17
8	5	19.6	48.586	5.8494	Q9BXS5;Q9BXS5-2	AP-1 complex subunit mu-1
5	5	16.2	28.32	5.8494	Q9UHQ4;Q9UHQ4-2	B-cell receptor-associated protein 29
3	3	15	26.227	5.8373	O43809	Cleavage and polyadenylation specificity factor subunit 5
3	3	26.3	16.937	5.8324	Q9Y3D6	Mitochondrial fission 1 protein
5	5	5.4	178.97	5.8319	Q9H3S7	Tyrosine-protein phosphatase non-receptor type 23
2	2	8.4	42.78	5.8275	Q53T59	HCLS1-binding protein 3
3	3	7.4	67.568	5.8249	P61764;P61764-2	Syntaxin-binding protein 1
2	2	21.7	15.225	5.8207	Q9BRT6	Protein LLP homolog
3	3	42.5	10.134	5.8191	Q9NYJ1;Q9NYJ1-2	Cytochrome c oxidase assembly factor 4 homolog, mitochondrial
3	3	22.2	24.701	5.813	Q9BUL8	Programmed cell death protein 10
6	6	24	42.91	5.8104	P49903;P49903-2;P49903-4;P49903-3	Selenide, water dikinase 1
5	5	23.9	35.932	5.8059	Q8NBN7;Q8NBN7-2	Retinol dehydrogenase 13
3	1	67.3	6.2953	5.8056	P56134-3;P56134	ATP synthase subunit f, mitochondrial
2	2	22.6	19.015	5.7987	Q96ET8-2;Q96ET8-3;Q9NYZ1;Q96ET8	Golgi apparatus membrane protein TVP23 homolog C;Golgi apparatus membrane protein TVP23 homolog B
3	3	12	36.665	5.7985	Q9HB09-3;Q9HB09	Bcl-2-like protein 12
2	2	2.6	99.536	5.7957	sp|Q0V8M9|;CON__Q0V8M9;Q06033-2;Q06033	Inter-alpha-trypsin inhibitor heavy chain H3
4	4	21.2	31.716	5.7948	Q99943	1-acyl-sn-glycerol-3-phosphate acyltransferase alpha
3	3	9.2	48.344	5.79	P11117;P11117-2	Lysosomal acid phosphatase
3	3	22.4	18.57	5.7889	Q9BUB7-2;Q9BUB7	Transmembrane protein 70, mitochondrial
4	4	55.3	11.967	5.7768	Q99417	C-Myc-binding protein
3	3	16.9	15.388	5.7741	Q71DI3;Q16695;P84243;P68431;Q6NXT2	Histone H3.2;Histone H3.1t;Histone H3.3;Histone H3.1;Histone H3.3C
4	4	10.7	52.428	5.7596	Q9ULA0	Aspartyl aminopeptidase
3	3	4.2	124.01	5.754	O95104-3;O95104;O95104-2	Splicing factor, arginine/serine-rich 15
2	2	8.8	30.518	5.7501	P27707	Deoxycytidine kinase
4	4	7.1	95.012	5.7497	Q86TI2-4;Q86TI2;Q86TI2-2	Dipeptidyl peptidase 9
3	3	19.3	34.91	5.7389	Q00577	Transcriptional activator protein Pur-alpha
3	3	9	46.832	5.7385	P48730-2;P48730;P49674	Casein kinase I isoform delta;Casein kinase I isoform epsilon
7	7	11.1	94.095	5.7355	Q96JB2	Conserved oligomeric Golgi complex subunit 3
5	5	13.7	46.626	5.7343	Q6PIU2-2;Q6PIU2;Q6PIU2-3	Neutral cholesterol ester hydrolase 1
2	2	10.4	31.716	5.7235	Q4G0N4-3;Q4G0N4-2;Q4G0N4	NAD kinase 2, mitochondrial
6	6	34.6	25.709	5.7228	Q96BR5	Cytochrome c oxidase assembly factor 7
4	4	30.3	14.21	5.7213	O60925	Prefoldin subunit 1
4	4	40.4	12.844	5.7165	P61326-2;P61326;Q96A72	Protein mago nashi homolog;Protein mago nashi homolog 2
1	1	4.5	27.368	5.7154	Q9BZM5	NKG2D ligand 2
1	1	1.7	132.94	5.707	Q7Z5K2;Q7Z5K2-2;Q7Z5K2-3	Wings apart-like protein homolog
5	5	2.9	272.5	5.7043	P46531	Neurogenic locus notch homolog protein 1;Notch 1 extracellular truncation;Notch 1 intracellular domain
2	2	17	24.49	5.7006	Q9NQ50	39S ribosomal protein L40, mitochondrial
2	2	21.8	15.511	5.6991	Q9NUG6	p53 and DNA damage-regulated protein 1
6	3	36.4	23.408	5.6973	P11234;P11234-3;P11234-2	Ras-related protein Ral-B
3	3	5.1	90.954	5.6954	Q93034	Cullin-5
1	1	5	53.715	5.6934	Q02447-3	
1	1	16.2	14.149	5.6933	Q8WYQ3	Coiled-coil-helix-coiled-coil-helix domain-containing protein 10, mitochondrial
7	3	22.1	58.82	5.6777	sp|FA46-21_d6|;sp|FA73-20|;sp|FA46-21_WT|;sp|FA36-21|	
3	3	2.6	197.29	5.672	Q7Z6E9-2;Q7Z6E9;Q7Z6E9-4	E3 ubiquitin-protein ligase RBBP6
5	5	9.5	79.87	5.6663	Q9NVU7;Q9NVU7-2	Protein SDA1 homolog
3	3	20	30.541	5.6624	O00124;O00124-3;O00124-2	UBX domain-containing protein 8

1	1	2.9	54.314	5.652	Q9ULK4-6;Q9ULK4-2;Q9ULK4-4;Q9ULK4-5;Q9ULK4-3;Q9ULK4	Mediator of RNA polymerase II transcription subunit 23
3	3	14	43.164	5.634	Q8NC42	E3 ubiquitin-protein ligase RNF149
4	4	20.1	33.81	5.6306	Q9H4A6;Q9H4A5	Golgi phosphoprotein 3;Golgi phosphoprotein 3-like
4	4	13.1	28.981	5.6269	O95297-2;O95297;O95297-4;O95297-3	Myelin protein zero-like protein 1
2	2	6.3	57.818	5.6197	O95376	E3 ubiquitin-protein ligase ARIH2
2	2	12.2	33.861	5.6177	Q9BV68	E3 ubiquitin-protein ligase RNF126
1	1	4.4	38.367	5.6147	Q9H8G2	Caspase activity and apoptosis inhibitor 1
3	3	15.4	26.624	5.6134	P41273	Tumor necrosis factor ligand superfamily member 9
1	1	3.2	34.293	5.6105	Q16836;Q16836-3;Q16836-2	Hydroxyacyl-coenzyme A dehydrogenase, mitochondrial
4	4	8.8	65.478	5.6077	Q8IXH7-4;Q8IXH7	Negative elongation factor C/D
3	3	11.3	41.385	5.6059	Q9Y679-3;Q9Y679	Ancient ubiquitous protein 1
3	3	5.4	111.65	5.6002	Q96JQ2	Calmin
4	4	24.1	21.838	5.5938	Q8TCC3-2;Q8TCC3;Q8TCC3-3	39S ribosomal protein L30, mitochondrial
3	3	4.9	82.562	5.5905	Q15003;Q15003-2	Condensin complex subunit 2
3	3	8.8	48.193	5.5866	Q9NQ84;Q9NQ84-2	G-protein coupled receptor family C group 5 member C
2	2	13.1	22.329	5.5847	O95057	GTP-binding protein Di-Ras1
2	2	12.6	33.939	5.577	P23511-2;P23511	Nuclear transcription factor Y subunit alpha
3	3	6.4	69.837	5.57	Q9UJV9	Probable ATP-dependent RNA helicase DDX41
1	1	3.6	61.002	5.5654	Q9NQS3	Nectin-3
2	2	5.9	45.409	5.5638	O95429-2;O95429	BAG family molecular chaperone regulator 4
4	4	20.1	29.328	5.5632	Q86U90	YrdC domain-containing protein, mitochondrial
5	5	35	13.293	5.561	P49207	60S ribosomal protein L34
3	3	19.6	21.052	5.554	Q9NY35-2;Q9NY35	Claudin domain-containing protein 1
3	3	4.6	96.109	5.5527	Q9BXP2;Q9BXP2-2;Q9BXP2-3;Q9BXP2-4	Solute carrier family 12 member 9
4	4	14.7	45.251	5.5515	Q92643;Q92643-2	GPI-anchor transamidase
3	3	36.5	13.178	5.5444	Q99622	Protein C10
1	1	4.3	34.516	5.5444	Q9NQX5	Neural proliferation differentiation and control protein 1
4	4	17.3	28.202	5.5424	Q9BVV7	Mitochondrial import inner membrane translocase subunit Tim21
2	2	14.7	20.497	5.5308	Q15382	GTP-binding protein Rheb
5	5	7.7	108.24	5.5285	Q92974-3;Q92974-2;Q92974	Rho guanine nucleotide exchange factor 2
3	3	22.2	17.27	5.5275	Q96K17;Q96K17-2	Transcription factor BTF3 homolog 4
4	4	26.8	26.132	5.521	Q12981;Q12981-1;Q12981-2;Q12981-3	Vesicle transport protein SEC20
5	5	34.2	21.666	5.5196	Q5W111;Q5W111-2	SPRY domain-containing protein 7
5	5	5.1	102.12	5.5132	Q8IYB3-2;Q8IYB3	Serine/arginine repetitive matrix protein 1
4	4	28	14.463	5.5085	P62899;P62899-3;P62899-2	60S ribosomal protein L31
5	5	2.7	229.14	5.5054	Q13459-2;Q13459	Unconventional myosin-IXb
2	2	5.3	60.535	5.49	P16278-2;P16278-3;P16278	Beta-galactosidase
2	2	6.3	48.672	5.4872	O60784-3;O60784;O60784-2;O60784-4	Target of Myb protein 1
4	4	35.9	13.667	5.487	P60520	Gamma-aminobutyric acid receptor-associated protein-like 2
5	5	28.2	19.208	5.4849	O43447;O43447-2	Peptidyl-prolyl cis-trans isomerase H
2	2	8.7	43.835	5.4813	Q99447;Q99447-3;Q99447-2;Q99447-4	Ethanolamine-phosphate cytidylyltransferase
5	4	10.6	63.107	5.4809	Q86YQ8;Q8IYJ1;Q9HCH3	Copine-8;Copine-9;Copine-5
3	3	19.9	33.098	5.4805	Q9H0W9-2;Q9H0W9;Q9H0W9-4;Q9H0W9-3	Ester hydrolase C11orf54
3	3	15.8	35.907	5.4773	Q96BW9-2;Q96BW9-3;Q96BW9	Phosphatidate cytidylyltransferase, mitochondrial
4	4	24.1	19.509	5.4665	P62253	Ubiquitin-conjugating enzyme E2 G1;Ubiquitin-conjugating enzyme E2 G1, N-terminally processed
2	2	5.6	65.746	5.4656	Q93052	Lipoma-preferred partner
3	3	5.3	98.615	5.4652	Q9H0X9;Q9H0X9-2;Q9H0X9-3	Oxysterol-binding protein-related protein 5
3	2	9.4	48.897	5.4617	Q9UN37	Vacuolar protein sorting-associated protein 4A
3	3	10.2	44.132	5.449	P28799-3;P28799;P28799-2	Granulins;Acrogranin;Paragranulin;Granulin-1;Granulin-2;Granulin-3;Granulin-4;Granulin-5;Granulin-6;Granulin-7
4	4	13.5	39.869	5.4478	Q9Y376	Calcium-binding protein 39

2	2	6.9	35.23	5.4403	Q9Y315	Deoxyribose-phosphate aldolase
4	1	1.7	327.82	5.435	Q8NFP9	Neurobeachin
6	6	23.3	28.048	5.4339	Q96DG6	Carboxymethylenebutenolidase homolog
4	4	7.4	73.139	5.4308	Q14155-1;Q14155-5;Q14155-6;Q14155-2;Q14155-3;Q14155	Rho guanine nucleotide exchange factor 7
2	2	12.4	32.824	5.4299	Q5BJH7-6;Q5BJH7-3;Q5BJH7;Q5BJH7-2;Q5BJH7-4;Q5BJH7-5	Protein YIF1B
3	3	32.2	13.291	5.424	Q5RI15;Q5RI15-2	Cytochrome c oxidase protein 20 homolog
2	2	11.6	19.1	5.4089	Q9UQN3-2;Q9UQN3	Charged multivesicular body protein 2b
2	2	6.4	41.894	5.3887	Q96HR8-2;Q96HR8	H/ACA ribonucleoprotein complex non-core subunit NAF1
2	2	16.1	20.462	5.3813	Q9Y221;Q9Y221-2	60S ribosome subunit biogenesis protein NIP7 homolog
2	2	5.2	28.86	5.3737	Q13277-2;Q13277-3;Q13277	Syntaxin-3
3	3	6.1	71.649	5.3703	Q8WXA9-2	Splicing regulatory glutamine/lysine-rich protein 1
4	4	7.9	60.576	5.3687	Q6P1J9	Parafibromin
2	2	9.8	41.293	5.3665	O75909-1;O75909;O75909-4;O75909-2	Cyclin-K
2	2	9.5	37.252	5.3596	Q8WWH5	Probable tRNA pseudouridine synthase 1
2	2	7.7	37.085	5.3588	Q9BW85	Coiled-coil domain-containing protein 94
4	4	8.6	60.12	5.3566	Q96E52;Q96E52-2	Metalloendopeptidase OMA1, mitochondrial
4	4	5.5	100.01	5.3553	Q9HCS7	Pre-mRNA-splicing factor SYF1
6	6	18.3	43.05	5.3528	Q07973-3;Q07973-2;Q07973	1,25-dihydroxyvitamin D(3) 24-hydroxylase, mitochondrial
2	2	11.2	22.202	5.3405	O75251-2;O75251	NADH dehydrogenase [ubiquinone] iron-sulfur protein 7, mitochondrial
4	4	14.2	28.295	5.3384	P48509	CD151 antigen
1	1	3.7	42.233	5.3377	A8MXV4	Nucleoside diphosphate-linked moiety X motif 19, mitochondrial
8	3	48.4	21.539	5.3375	Q9NVJ2;Q9NVJ2-2	ADP-ribosylation factor-like protein 8B
3	3	13.4	34.562	5.3343	Q7RTP0;Q7RTP0-2	Magnesium transporter NIPA1
3	3	9	50.47	5.3321	P12694;P12694-2	2-oxoisovalerate dehydrogenase subunit alpha, mitochondrial
2	2	8.6	33.043	5.3295	P29084	Transcription initiation factor IIE subunit beta
5	5	2.5	322.21	5.3259	Q12830-4;Q12830-2;Q12830	Nucleosome-remodeling factor subunit BPTF
3	3	5.9	71.949	5.3173	Q8NI60;Q8NI60-3	Atypical kinase ADCK3, mitochondrial
3	3	10.9	43.472	5.3084	Q9Y312	Protein AAR2 homolog
6	6	13.6	65.521	5.3068	O60508	Pre-mRNA-processing factor 17
4	4	12.1	36.249	5.3009	P53597	Succinyl-CoA ligase [ADP/GDP-forming] subunit alpha, mitochondrial
5	3	22.3	30.035	5.2918	P24941-2;P24941;Q00526	Cyclin-dependent kinase 2;Cyclin-dependent kinase 3
3	3	7.2	65.572	5.2809	Q9NVN8	Guanine nucleotide-binding protein-like 3-like protein
4	4	39.4	12.417	5.272	P53680-2;P53680	AP-2 complex subunit sigma
2	2	26.1	9.8745	5.2691	Q9BQB6-3;Q9BQB6-2;Q9BQB6	Vitamin K epoxide reductase complex subunit 1
4	4	13.6	45.756	5.2683	Q9UJW0-2;Q9UJW0;Q9UJW0-3	Dynactin subunit 4
1	1	31.8	5.0256	5.2622	P63313	Thymosin beta-10
4	4	18.2	24.737	5.258	O95372	Acyl-protein thioesterase 2
4	4	12.7	46.491	5.2547	Q9HD23-2;Q9HD23;Q9HD23-4	Magnesium transporter MRS2 homolog, mitochondrial
2	2	2.7	171.49	5.2527	Q9P265	Disco-interacting protein 2 homolog B
2	2	9.2	26.779	5.2501	O75954	Tetraspanin-9
3	3	22.2	20.747	5.2494	Q9BRG1	Vacuolar protein-sorting-associated protein 25
3	3	6.9	63.069	5.2428	Q9UQ53-2;Q9UQ53;Q9UQ53-3	Alpha-1,3-mannosyl-glycoprotein 4-beta-N-acetylglucosaminyltransferase B
5	5	13	57.518	5.2406	Q9HCC0-2;Q9HCC0	Methylcrotonoyl-CoA carboxylase beta chain, mitochondrial
2	2	2.6	134.32	5.239	Q96JH7	Deubiquitinating protein VCIP135
4	3	13.7	40.736	5.2368	Q7LG56;Q7LG56-6;Q7LG56-5;Q7LG56-2	Ribonucleoside-diphosphate reductase subunit M2 B
3	3	11.7	28.991	5.2293	Q9NPF0;Q9NPF0-2	CD320 antigen
3	3	11.5	44.91	5.2267	Q96A59	MARVEL domain-containing protein 3
3	3	5.7	96.256	5.2213	Q5C9Z4	Nucleolar MIF4G domain-containing protein 1
3	3	14.6	29.321	5.2143	P78346;P78346-2	Ribonuclease P protein subunit p30

4	4	10.3	52.07	5.2028	Q86UL3	Glycerol-3-phosphate acyltransferase 4
3	3	19.6	25.545	5.2023	Q15404-2;Q15404	Ras suppressor protein 1
1	1	2.8	44.621	5.1987	Q9HAS0	Protein Njmu-R1
4	4	13.5	44.961	5.1898	Q96I25	Splicing factor 45
2	2	11.3	21.701	5.1846	Q3MHD2;Q3MHD2-2	Protein LSM12 homolog
2	2	38.1	7.3084	5.1833	Q9UDW1;Q9UDW1-2	Cytochrome b-c1 complex subunit 9
1	1	7.4	17.442	5.1755	Q9BYC9	39S ribosomal protein L20, mitochondrial
1	1	5	29.918	5.1729	P78382-2;P78382	CMP-sialic acid transporter
1	1	2.3	68.423	5.167	Q96MW5	Conserved oligomeric Golgi complex subunit 8
2	2	8.4	34.607	5.1611	Q969X1	Protein lifeguard 3
3	3	16.2	32.761	5.13	Q9UHW5;Q9UHW5-2;Q9UHW5-3	GPN-loop GTPase 3
5	5	7.8	77.528	5.1299	O43395	U4/U6 small nuclear ribonucleoprotein Prp3
4	4	14.9	38.632	5.12	P09001	39S ribosomal protein L3, mitochondrial
5	3	16.2	39.851	5.1164	Q9UHE8	Metalloreductase STEAP1
4	4	10.8	55.65	5.1102	Q2TAA5	GDP-Man:Man(3)GlcNAc(2)-PP-Dol alpha-1,2-mannosyltransferase
4	4	16.9	33.085	5.107	Q9UNP9-2;Q9UNP9;Q9UNP9-3	Peptidyl-prolyl cis-trans isomerase E
4	4	21.1	38.771	5.1012	Q96J01;Q96J01-2	THO complex subunit 3
7	4	21.1	57.886	5.0992	O00629	Importin subunit alpha-3
5	4	4.7	160.9	5.0818	O75116	Rho-associated protein kinase 2
3	3	4.6	99.057	5.08	Q96QC0	Serine/threonine-protein phosphatase 1 regulatory subunit 10
4	4	4.8	116.2	5.0752	O94832	Unconventional myosin-Id
3	3	10.6	50.968	5.075	Q9HBU6	Ethanolamine kinase 1
2	2	10.5	34.596	5.0661	Q96GK7;Q6P2I3	Fumarylacetoacetate hydrolase domain-containing protein 2A;Fumarylacetoacetate hydrolase domain-containing protein 2B
6	4	7.9	107.65	5.0621	Q8TB72-4;Q8TB72-3;Q8TB72-2;Q8TB72	Pumilio homolog 2
3	3	13.5	37.765	5.0556	Q96C23	Aldose 1-epimerase
3	3	9.9	43.359	5.0551	Q9BRX2	Protein pelota homolog
2	2	5.6	54.664	5.0524	Q86UY8-2;Q86UY8	5-nucleotidase domain-containing protein 3
4	4	14.4	51.396	5.0513	Q86VR2	Protein FAM134C
4	4	5.3	118.01	5.0495	Q68E01-2;Q68E01;Q68E01-3;Q68E01-4	Integrator complex subunit 3
3	3	32.3	15.237	5.0494	P0DPB6	
2	2	7.4	50.576	5.048	Q53EL6-2;Q53EL6	Programmed cell death protein 4
3	3	6.5	81.448	5.0475	Q86UV5-3;Q86UV5-2;Q86UV5;Q86UV5-8;Q86UV5-7	Ubiquitin carboxyl-terminal hydrolase 48
2	2	11.5	24.061	5.0464	Q9UEE9-2;Q9UEE9	Craniofacial development protein 1
3	3	14.3	25.268	5.0272	P27144	Adenylate kinase 4, mitochondrial
4	4	10.9	54.699	5.0272	Q16537;Q16537-2;Q16537-3	Serine/threonine-protein phosphatase 2A 56 kDa regulatory subunit epsilon isoform
2	2	8.1	51.144	5.0271	Q9Y5Q0	Fatty acid desaturase 3
1	1	7.4	25.788	5.027	Q9BVX2-2;Q9BVX2	Transmembrane protein 106C
4	4	11.3	58.215	5.0179	P05166;P05166-2	Propionyl-CoA carboxylase beta chain, mitochondrial
2	2	20.7	15.032	5.017	sp|FA18-18-Q9JJV2|PROF2_MOUSE;P35080-2;P35080	Profilin-2
2	2	18.1	25.425	5.0157	Q9UK41;Q9UK41-2	Vacuolar protein sorting-associated protein 28 homolog
1	1	22.6	9.2234	5.0116	Q9NRR3	CDC42 small effector protein 2
3	3	15.5	32.424	4.9913	P32856-3;P32856-2;P32856	Syntaxin-2
4	4	36.7	15.35	4.9765	Q9Y4Z0	U6 snRNA-associated Sm-like protein LSm4
2	2	8.8	32.177	4.9695	Q9NPL8	Complex I assembly factor TIMMDC1, mitochondrial
2	2	4.4	60.908	4.9684	O00400	Acetyl-coenzyme A transporter 1
2	2	12.6	36.688	4.9679	P16220;P16220-2;P16220-3	Cyclic AMP-responsive element-binding protein 1
2	2	13.9	28.544	4.9671	Q68CQ7-2;Q68CQ7	Glycosyltransferase 8 domain-containing protein 1
3	1	18.6	21.569	4.9669	Q13636	Ras-related protein Rab-31

1	1	4.2	46.587	4.9598	Q96EB1;Q96EB1-3;Q96EB1-2	Elongator complex protein 4
5	5	18.4	36.318	4.9575	O95218-2;O95218	Zinc finger Ran-binding domain-containing protein 2
4	4	5.9	103.96	4.9514	Q4KMQ2-3;Q4KMQ2;Q4KMQ2-2;Q4KMQ2-4	Anoctamin-6
2	2	28.9	9.3435	4.9438	Q9Y5J9	Mitochondrial import inner membrane translocase subunit Tim8 B
2	2	8.1	44.301	4.9423	Q9NXX6;Q9NXX6-2	Non-structural maintenance of chromosomes element 4 homolog A
4	2	14	50.327	4.9397	sp|Q2YDI2|;CON__Q2YDI2	
2	2	25.2	14.686	4.9327	Q8N4V1;Q8N4V1-2	Membrane magnesium transporter 1
3	3	3.3	135.15	4.9247	O94854;O94854-2	Uncharacterized protein KIAA0754
4	4	8.3	81.852	4.9236	O00471	Exocyst complex component 5
3	3	10.8	54.705	4.9211	Q8IV08	Phospholipase D3
4	4	5.5	110.5	4.9185	Q96A65;Q96A65-2	Exocyst complex component 4
3	3	11.3	35.776	4.9171	Q9NRN7	L-aminoadipate-semialdehyde dehydrogenase-phosphopantetheinyl transferase
4	4	24.3	23.63	4.917	Q14197	Peptidyl-tRNA hydrolase ICT1, mitochondrial
6	2	20.5	42.003	4.9152	Q562R1	Beta-actin-like protein 2
3	3	14.8	27.013	4.8956	Q9HBH1	Peptide deformylase, mitochondrial
9	1	57.2	17.149	4.869	P15531;P15531-2	Nucleoside diphosphate kinase A
4	4	18.4	27.949	4.8675	Q9BZM4	NKG2D ligand 3
4	4	17.4	32.236	4.8591	Q96CN7	Isochorismatase domain-containing protein 1
3	3	13.7	29.982	4.8571	Q9BRP4-3;Q9BRP4-2;Q9BRP4	Proteasomal ATPase-associated factor 1
3	2	17.4	23.586	4.8555	P61018;P61018-2	Ras-related protein Rab-4B
2	2	7.8	45.476	4.8541	Q99986	Serine/threonine-protein kinase VRK1
7	7	19.8	36.034	4.8455	Q9NZ01	Very-long-chain enoyl-CoA reductase
2	2	8	34.36	4.8424	P35914	Hydroxymethylglutaryl-CoA lyase, mitochondrial
2	2	35.6	12.878	4.839	Q9UFG5;Q9UFG5-2	UPF0449 protein C19orf25
2	2	7.4	44.825	4.8364	Q9BVC3	Sister chromatid cohesion protein DCC1
4	4	3.1	198.04	4.8346	P07942	Laminin subunit beta-1
1	1	3.6	53.148	4.833	Q9H6A0-2;Q9H6A0	DENN domain-containing protein 2D
3	3	2.4	195.91	4.826	Q8IWJ2	GRIP and coiled-coil domain-containing protein 2
5	5	5.1	133.97	4.8243	Q13136-2;Q13136	Liprin-alpha-1
3	3	15.4	17.417	4.8196	O14561	Acyl carrier protein, mitochondrial
2	2	6.8	60.197	4.8053	P36894	Bone morphogenetic protein receptor type-1A
2	2	11.2	22.35	4.8036	Q14919;Q14919-2	Dr1-associated corepressor
5	5	9.5	99.7	4.8002	Q96T37-4;Q96T37-2;Q96T37-3;Q96T37	Putative RNA-binding protein 15
1	1	6.9	24.269	4.7921	Q8NDC0	MAPK-interacting and spindle-stabilizing protein-like
3	3	17.1	28.449	4.7903	Q9NX20	39S ribosomal protein L16, mitochondrial
2	2	9.7	21.527	4.7902	Q9H061	Transmembrane protein 126A
2	2	1.5	261.51	4.7837	Q07864	DNA polymerase epsilon catalytic subunit A
2	2	3	122.07	4.7826	O43156	TELO2-interacting protein 1 homolog
2	2	2.7	113.01	4.7789	O94855;O94855-2	Protein transport protein Sec24D
3	3	7.5	61.863	4.7783	O75879	Glutamyl-tRNA(Gln) amidotransferase subunit B, mitochondrial
3	3	9.9	46.914	4.7672	Q9NXH8	Torsin-4A
2	2	30.4	7.9331	4.7664	P56385	ATP synthase subunit e, mitochondrial
10	2	30.6	32.722	4.7616	P67936-2	Tropomyosin alpha-4 chain
2	2	7	51.246	4.7602	O94763-2;O94763-3;O94763;O94763-4	Unconventional prefoldin RPB5 interactor 1
2	2	15.3	21.789	4.758	Q587I9	Vesicle transport protein SFT2C
4	4	28.1	22.975	4.7572	sp|P02662|;CON__P02662	
2	2	18.7	18.237	4.7456	Q9Y3C6	Peptidyl-prolyl cis-trans isomerase-like 1
4	4	11.8	60.438	4.7434	Q13485	Mothers against decapentaplegic homolog 4
3	3	23.8	21.097	4.7431	Q9NRV9	Heme-binding protein 1
3	3	14.7	32.068	4.7429	Q9UHR5-2;Q9UHR5	SAP30-binding protein
3	3	1	434.41	4.7348	Q9Y4A5-2;Q9Y4A5	Transformation/transcription domain-associated protein

2	2	5.9	41.625	4.7303	Q8NEW0	Zinc transporter 7
3	3	11.9	43.214	4.7282	Q96FK6	WD repeat-containing protein 89
6	6	7.8	118.1	4.7248	Q8IXT5	RNA-binding protein 12B
2	2	10.3	23.239	4.7247	Q9BVM2	Protein DPCD
4	4	2.4	248.42	4.7242	Q92673	Sortilin-related receptor
3	3	8.5	41.631	4.7204	Q9NX61-2;Q9NX61	Transmembrane protein 161A
4	4	2.8	192.59	4.7186	Q9UKK3	Poly [ADP-ribose] polymerase 4
3	3	17.7	23.944	4.7097	Q9NSI2-2;Q9NSI2	Protein FAM207A
2	2	8.6	31.614	4.7063	P55210-4;P55210;P55210-3	Caspase-7;Caspase-7 subunit p20;Caspase-7 subunit p11
2	2	4.4	81.68	4.7055	Q96EB6	NAD-dependent protein deacetylase sirtuin-1;SirtT1 75 kDa fragment
4	4	6.6	282.39	4.7044	sp|Q86YZ3|;CON__Q86YZ3;Q86YZ3	Hornerin
5	5	7.2	92.02	4.7022	P04035-2;P04035;P04035-3	3-hydroxy-3-methylglutaryl-coenzyme A reductase
2	2	2.2	148.53	4.6966	P19174;P19174-2	1-phosphatidylinositol 4,5-bisphosphate phosphodiesterase gamma-1
4	4	26.1	20.576	4.6961	Q9H0U6	39S ribosomal protein L18, mitochondrial
4	4	9.9	64.129	4.694	Q4G176	Acyl-CoA synthetase family member 3, mitochondrial
3	3	4.8	92.153	4.6907	P52756;P52756-4;P52756-2;P52756-5	RNA-binding protein 5
2	2	4.3	88.463	4.6905	P51790-4;P51790-5;P51790;P51790-2	H(+)/Cl(-) exchange transporter 3
1	1	5.5	38.066	4.688	Q96J42-2;Q96J42	Thioredoxin domain-containing protein 15
2	2	12.6	23.369	4.6822	Q9Y324	rRNA-processing protein FCF1 homolog
2	2	7	25.416	4.6795	P21926	CD9 antigen
3	3	10.2	47.299	4.6782	Q8TBX8;Q8TBX8-3	Phosphatidylinositol 5-phosphate 4-kinase type-2 gamma
6	6	7.9	85.466	4.6708	Q8IZ52;Q8IZ52-4;Q8IZ52-2	Chondroitin sulfate synthase 2
4	4	21.5	29.075	4.6618	Q9GZU7-2;Q9GZU7-3;Q9GZU7	Carboxy-terminal domain RNA polymerase II polypeptide A small phosphatase 1
3	3	6.6	68.249	4.6595	Q9H9A6	Leucine-rich repeat-containing protein 40
2	2	10.9	30.243	4.6563	Q9BYD2	39S ribosomal protein L9, mitochondrial
3	3	18.5	27.419	4.6549	O95639-3;O95639-2;O95639	Cleavage and polyadenylation specificity factor subunit 4
8	3	12	69.366	4.6528	sp|P02768-1|;CON__P02768-1;P02768;P02768-2;P02768-3	Serum albumin
4	4	17.3	32.772	4.6469	Q8N5M1	ATP synthase mitochondrial F1 complex assembly factor 2
1	1	7.3	23.55	4.6423	Q7Z7N9	Transmembrane protein 179B
4	2	2.8	184.7	4.6383	Q86VI3	Ras GTPase-activating-like protein IQGAP3
2	2	11.1	29.745	4.637	Q9NPJ6;Q9NPJ6-2	Mediator of RNA polymerase II transcription subunit 4
4	4	4.7	150.66	4.6312	O94966-7;O94966-6;O94966-5;O94966-4;O94966-3;O94966;O94966-2	Ubiquitin carboxyl-terminal hydrolase 19
2	2	7.9	38.964	4.628	O14681;O14681-3	Etoposide-induced protein 2.4 homolog
2	2	3.9	79.158	4.6277	Q9BXS9-7;Q9BXS9-2;Q9BXS9-3;Q9BXS9;Q9BXS9-5;Q9BXS9-4	Solute carrier family 26 member 6
3	2	3.6	139.31	4.6106	Q14562	ATP-dependent RNA helicase DHX8
3	3	6.8	63.422	4.6105	Q9BSJ5-2;Q9BSJ5-3;Q9BSJ5	Uncharacterized protein C17orf80
2	2	17.1	8.496	4.6102	P62308;A8MWD9	Small nuclear ribonucleoprotein G;Putative small nuclear ribonucleoprotein G-like protein 15
4	2	31.2	13.509	4.6053	Q71UI9;P0C0S5;Q71UI9-3;Q71UI9-4;Q71UI9-2	Histone H2A.V;Histone H2A.Z
3	3	8.9	59.309	4.6042	Q9BUR4	Telomerase Cajal body protein 1
2	2	20.5	14.652	4.5986	Q8N8J7	Uncharacterized protein C4orf32
2	2	8.3	39.834	4.5944	Q9Y5P6;Q9Y5P6-2	Mannose-1-phosphate guanyltransferase beta
3	3	4.4	73.699	4.5936	Q8WUM9	Sodium-dependent phosphate transporter 1
4	4	8.8	72.259	4.5876	Q92581-3;Q92581-2;Q92581	Sodium/hydrogen exchanger 6
2	2	30.8	14.632	4.5796	Q01628;P13164;Q01629	Interferon-induced transmembrane protein 3;Interferon-induced transmembrane protein 1;Interferon-induced transmembrane protein 2
4	1	11.4	42.293	4.5595	P42025	Beta-centractin
3	3	2	248.98	4.5587	sp|Q28107|;CON__Q28107	
2	2	5.8	53.96	4.5552	Q9BRR6-2;Q9BRR6;Q9BRR6-4;Q9BRR6-3	ADP-dependent glucokinase

2	2	13	37.091	4.5489	Q9H4I3-2;Q9H4I3	TraB domain-containing protein
3	3	6.9	58.246	4.5439	Q32P41	tRNA (guanine(37)-N1)-methyltransferase
4	4	15	31.545	4.5432	Q9NVA1-2;Q9NVA1;Q9NVA1-4;Q9NVA1-5	Ubiquinol-cytochrome-c reductase complex assembly factor 1
1	1	16.2	10.852	4.5319	P02656	Apolipoprotein C-III
3	3	2.3	205.14	4.5286	Q92545	Transmembrane protein 131
8	8	17.7	71.45	4.5191	Q9Y5K6	CD2-associated protein
3	3	12.4	17.256	4.5165	Q9UNX3;P61254	60S ribosomal protein L26-like 1;60S ribosomal protein L26
3	3	15.5	20.843	4.5101	Q9Y2Q9	28S ribosomal protein S28, mitochondrial
5	5	7.3	95.801	4.5034	P49916-4;P49916-3;P49916-2;P49916	DNA ligase 3
1	1	19.4	10.143	4.5032	Q9Y241	HIG1 domain family member 1A, mitochondrial
2	2	23.2	10.685	4.5024	Q6EEV6;P61956-2;P61956;P55854;P55854-2	Small ubiquitin-related modifier 4;Small ubiquitin-related modifier 2;Small ubiquitin-related modifier 3
2	2	5.7	43.327	4.5002	Q15035	Translocating chain-associated membrane protein 2
1	1	2.7	50.21	4.4938	Q9BVL2-2;Q9BVL2-3;Q9BVL2	Nucleoporin p58/p45
15	0	20.9	60.066	4.4925	P04259	Keratin, type II cytoskeletal 6B
3	3	14.9	28.147	4.4924	Q15773	Myeloid leukemia factor 2
3	3	5.2	94.086	4.4919	Q9H8H2	Probable ATP-dependent RNA helicase DDX31
3	3	12.4	32.544	4.4851	Q8WWC4	Uncharacterized protein C2orf47, mitochondrial
11	1	51.5	35.575	4.474	P62714	Serine/threonine-protein phosphatase 2A catalytic subunit beta isoform
2	2	7.8	33.62	4.4729	A6NDU8	UPF0600 protein C5orf51
4	4	19	35.582	4.4725	Q9H7B2	Ribosome production factor 2 homolog
2	2	10.1	29.159	4.4662	Q6UXV4	MICOS complex subunit MIC27
3	3	15	30.281	4.4623	Q9H0E2;Q9H0E2-2	Toll-interacting protein
3	1	21.2	22.41	4.4537	Q9Y6B6	GTP-binding protein SAR1b
2	2	6.2	45.02	4.4525	Q8IUX4	DNA dC->dU-editing enzyme APOBEC-3F
3	3	20.8	25.348	4.4429	Q66PJ3-7;Q66PJ3-4;Q66PJ3-3;Q66PJ3-2;Q66PJ3	ADP-ribosylation factor-like protein 6-interacting protein 4
3	3	21.7	17.711	4.4394	Q9Y2Y0-2;Q9Y2Y0	ADP-ribosylation factor-like protein 2-binding protein
4	4	14.5	45.283	4.4388	O43813	LanC-like protein 1
5	5	18.1	42.932	4.4382	A4D1E9;A4D1E9-2	GTP-binding protein 10
2	2	9	18.383	4.435	Q99757	Thioredoxin, mitochondrial
3	3	5.7	93.484	4.4335	Q9Y5B0-4;Q9Y5B0	RNA polymerase II subunit A C-terminal domain phosphatase
4	4	14.1	52.432	4.4312	Q6UW02;Q6UW02-2	Cytochrome P450 20A1
4	4	14.2	43.404	4.4262	P53602	Diphosphomevalonate decarboxylase
3	3	20	20.117	4.4255	O75494-5;O75494-4;O75494-6;O75494-3;O75494-2;O75494;Q8WXF0	Serine/arginine-rich splicing factor 10;Serine/arginine-rich splicing factor 12
1	1	4.5	42.615	4.4242	Q96A54	Adiponectin receptor protein 1
6	3	14.2	48.108	4.4212	Q9Y6Q5;Q9Y6Q5-2	AP-1 complex subunit mu-2
2	2	5.1	69.863	4.4196	Q9H6U8;Q9H6U8-3;Q9H6U8-2;Q9H6U8-4	Alpha-1,2-mannosyltransferase ALG9
4	4	13.6	43.374	4.418	Q5JVF3-3;Q5JVF3-2;Q5JVF3;Q5JVF3-4	PCI domain-containing protein 2
1	1	6.5	24.402	4.4171	Q6PHR2-2;Q6PHR2-3;Q6PHR2;Q6PHR2-4	Serine/threonine-protein kinase ULK3
3	3	9.6	39.759	4.4159	P08174-4;P08174;P08174-3;P08174-5;P08174-2;P08174-6;P08174-7	Complement decay-accelerating factor
2	2	6.6	44.876	4.4158	Q96G23	Ceramide synthase 2
3	3	6.4	65.225	4.4139	Q86YP4-2;Q86YP4;Q86YP4-3	Transcriptional repressor p66-alpha
3	3	17.9	19.667	4.4023	P59998;P59998-3;P59998-2	Actin-related protein 2/3 complex subunit 4
3	3	8.4	55.786	4.4019	Q9Y6K9-2;Q9Y6K9;Q9Y6K9-3	NF-kappa-B essential modulator
1	1	4.9	32.992	4.4009	Q9ULR0;Q9ULR0-2;Q9ULR0-1	Pre-mRNA-splicing factor ISY1 homolog
2	2	6.8	49.298	4.3953	Q8IV38	Ankyrin repeat and MYND domain-containing protein 2
3	3	10.9	29.395	4.3946	Q9H6H4;Q9H6H4-2	Receptor expression-enhancing protein 4
6	6	21.3	41.151	4.39	Q9NW64-2;Q9NW64	Pre-mRNA-splicing factor RBM22
3	3	8.1	62.003	4.3874	Q9Y5J1	U3 small nucleolar RNA-associated protein 18 homolog
2	2	3.2	79.817	4.3825	Q96T51;Q96T51-2;Q96T51-3	RUN and FYVE domain-containing protein 1

4	4	19.7	26.321	4.3819	Q969U7-2;Q969U7	Proteasome assembly chaperone 2
4	4	3.1	206.89	4.3811	O14981	TATA-binding protein-associated factor 172
1	1	4.2	33.24	4.3792	Q96QR8	Transcriptional activator protein Pur-beta
4	3	6.8	86.606	4.3777	Q6P4Q7	Metal transporter CNNM4
2	2	10	27.559	4.3758	P49447	Cytochrome b561
3	3	8.3	51.9	4.3727	Q9NQ55-2;Q9NQ55;Q9NQ55-3	Suppressor of SWI4 1 homolog
5	4	6.4	118.72	4.3573	Q9P2N5	RNA-binding protein 27
5	2	41	20.615	4.3558	P10114	Ras-related protein Rap-2a
1	1	3.6	43.963	4.3527	Q9NWT1	p21-activated protein kinase-interacting protein 1
2	2	10.9	29.388	4.3514	Q16539-5;Q16539-3;Q16539-4;Q16539-2;Q16539	Mitogen-activated protein kinase 14
4	4	17.2	26.152	4.3487	P17931	Galectin-3
3	3	13	36.174	4.3479	Q9HA72;Q9HA72-3;Q9HA72-2	Calcium homeostasis modulator protein 2
1	1	0.9	162.47	4.3459	Q8NFD5-4;Q8NFD5;Q8NFD5-2;Q8NFD5-3	AT-rich interactive domain-containing protein 1B
2	2	11.7	38.441	4.3419	Q96I51-2;Q96I51-3;Q96I51	Williams-Beuren syndrome chromosomal region 16 protein
3	3	6.9	57.117	4.3362	Q9BXI6;Q9BXI6-2	TBC1 domain family member 10A
3	3	26.3	15.383	4.3301	Q8IXM3	39S ribosomal protein L41, mitochondrial
6	3	12.6	51.385	4.3219	sp|Q3KNV1|;sp|P08729|;CON__Q3KNV1;CON__P08729;P08729	Keratin, type II cytoskeletal 7
6	6	11.4	63.882	4.3163	Q96KC8	DnaJ homolog subfamily C member 1
3	3	34.4	11.25	4.3129	L0R819	
3	3	16.6	28.823	4.3116	O75431-2;O75431	Metaxin-2
2	2	8.5	40.034	4.3091	Q9NWU1-2;Q9NWU1	3-oxoacyl-[acyl-carrier-protein] synthase, mitochondrial
2	2	33.9	11.829	4.3026	Q96GE9-2;Q96GE9	Transmembrane protein 261
1	1	7.1	18.298	4.2964	Q9Y6A9	Signal peptidase complex subunit 1
2	2	9.2	37.395	4.2917	P49757-9;P49757-8;P49757-7;P49757-6;P49757-5;P49757-4;P49757-2;P49757-3;P49757	Protein numb homolog
4	4	5.3	105.29	4.2916	Q15042-4;Q15042;Q15042-3	Rab3 GTPase-activating protein catalytic subunit
4	4	10.5	47.695	4.2839	O15554	Intermediate conductance calcium-activated potassium channel protein 4
3	2	37.5	12.323	4.2795	P56211;P56211-2	cAMP-regulated phosphoprotein 19
2	2	21.1	16.093	4.2791	Q9Y3C4-2;Q9Y3C4;Q9Y3C4-3	EKC/KEOPS complex subunit TPRKB
1	1	5.8	36.633	4.2762	Q8WZ73-3;Q8WZ73-2;Q8WZ73	E3 ubiquitin-protein ligase rififylin
3	3	5.4	84.784	4.272	O60568	Procollagen-lysine,2-oxoglutarate 5-dioxygenase 3
1	1	8.3	19.881	4.2694	Q99942	E3 ubiquitin-protein ligase RNF5
5	5	24.4	14.187	4.2686	O95298;O95298-2	NADH dehydrogenase [ubiquinone] 1 subunit C2
4	1	12.8	57.557	4.2686	Q9UI10	Translation initiation factor eIF-2B subunit delta
2	2	7	44.598	4.2671	Q96Q45-2;Q96Q45;Q96Q45-5;Q96Q45-3	Transmembrane protein 237
2	2	9	30.503	4.2616	Q9Y580	RNA-binding protein 7
2	2	20.2	13.596	4.26	O43805	Sjoegren syndrome nuclear autoantigen 1
4	4	17.9	44.129	4.26	Q92968	Peroxisomal membrane protein PEX13
2	2	8.2	51.095	4.2582	Q8N684-2;Q8N684;Q8N684-3	Cleavage and polyadenylation specificity factor subunit 7
2	2	6.9	54.341	4.2563	Q9UHL4	Dipeptidyl peptidase 2
3	3	9.1	45.348	4.2561	Q9H5Q4	Dimethyladenosine transferase 2, mitochondrial
2	2	15.5	24.955	4.2557	Q9NQX7-2;Q9NQX7-3;Q9NQX7	Integral membrane protein 2C;CT-BRI3
2	2	3.4	71.381	4.2487	P23469-3;P23469-2;P23469	Receptor-type tyrosine-protein phosphatase epsilon
2	2	6.1	46.691	4.2484	Q96P11;Q96P11-2;Q96P11-4;Q96P11-5	Probable 28S rRNA (cytosine-C(5))-methyltransferase
4	4	8.5	65.536	4.2437	Q9HCD5	Nuclear receptor coactivator 5
3	3	3.6	128.78	4.2313	Q6AI08	HEAT repeat-containing protein 6
3	2	6.1	75.171	4.2123	Q16513-5;Q16513-4;Q16513-3;Q16513-2;Q16513	Serine/threonine-protein kinase N2
2	2	2.3	134.32	4.207	Q9H0H0	Integrator complex subunit 2

11	0	30.9	41.915	4.2064	sp|FA87-21_4|;sp|P68032|;sp|FA87-21_1|;P68133;P68032;sp|FA87-21_6|;sp|FA87-21_5|;sp|FA87-21_3|;sp|FA87-21_2|;P63267;P62736	Actin, alpha skeletal muscle;Actin, alpha cardiac muscle 1;Actin, gamma-enteric smooth muscle;Actin, aortic smooth muscle
3	3	6.3	57.949	4.2061	Q13425	Beta-2-syntrophin
5	5	6	130.14	4.2061	Q9C0E2	Exportin-4
3	3	7.1	72.189	4.2059	Q9UGP4	LIM domain-containing protein 1
3	3	5.5	72.655	4.2059	Q9UID3-2;Q9UID3	Vacuolar protein sorting-associated protein 51 homolog
5	5	12.8	51.84	4.2031	O43818	U3 small nucleolar RNA-interacting protein 2
2	2	5.2	55.827	4.1953	Q13610	Periodic tryptophan protein 1 homolog
3	3	11.2	45.439	4.1903	Q96HD1;Q96HD1-2	Cysteine-rich with EGF-like domain protein 1
1	1	12.7	11.667	4.1843	Q96DE5	Anaphase-promoting complex subunit 16
1	1	3.1	63.244	4.1817	Q7Z6J6-2;Q7Z6J6	FERM domain-containing protein 5
2	2	20	11.284	4.1758	P81605;P81605-2	Dermcidin;Survival-promoting peptide;DCD-1
2	2	4.4	95.575	4.1682	Q5VSL9;Q5VSL9-2;Q5VSL9-3	Striatin-interacting protein 1
5	4	26.1	23.155	4.1632	O14966	Ras-related protein Rab-7L1
2	2	13.3	29.502	4.161	Q9BYD3-2;Q9BYD3	39S ribosomal protein L4, mitochondrial
1	1	10.3	10.631	4.1585	Q9H1C7	Cysteine-rich and transmembrane domain-containing protein 1
2	2	2.6	111.26	4.1548	Q8IVF7-2;Q8IVF7-3;Q8IVF7;Q96PY5;Q96PY5-3	Formin-like protein 3;Formin-like protein 2
2	2	4.3	83.135	4.135	Q14746-2;Q14746	Conserved oligomeric Golgi complex subunit 2
3	3	19	18.624	4.1319	O14735-3;O14735	CDP-diacylglycerol--inositol 3-phosphatidyltransferase
4	4	30.2	21.498	4.1299	Q9BV57;Q9BV57-2	1,2-dihydroxy-3-keto-5-methylthiopentene dioxygenase
1	1	10.3	22.147	4.1297	O95857	Tetraspanin-13
2	2	6.5	46.36	4.1193	O43826;O43826-2	Glucose-6-phosphate translocase
2	2	8.7	31.571	4.1184	Q6PCB8-2;Q6PCB8	Embigin
4	4	8.3	78.767	4.1132	Q8N6R0;Q8N6R0-2;Q8N6R0-1;Q8N6R0-3;Q8N6R0-4	Methyltransferase-like protein 13
2	2	9.7	37.359	4.1096	Q8N0X4;Q8N0X4-2	Citrate lyase subunit beta-like protein, mitochondrial
2	2	6.2	41.823	4.1054	Q9UBN6	Tumor necrosis factor receptor superfamily member 10D
2	2	8.9	41.054	4.105	Q9NZJ6	Ubiquinone biosynthesis O-methyltransferase, mitochondrial
3	3	6.4	66.591	4.1028	Q9Y5X1	Sorting nexin-9
3	3	37	9.3697	4.0994	O00483	Cytochrome c oxidase subunit NDUFA4
2	2	11.5	24.428	4.0983	O75832;O75832-2	26S proteasome non-ATPase regulatory subunit 10
4	4	15.6	26.423	4.0982	Q9BUN8-2;Q9BUN8	Derlin-1
4	4	10.3	73.584	4.0959	Q15397	Pumilio domain-containing protein KIAA0020
2	2	5.6	67.026	4.0942	P03915	NADH-ubiquinone oxidoreductase chain 5
1	1	6.3	22.831	4.092	P25208	Nuclear transcription factor Y subunit beta
3	3	3.3	128.88	4.0891	Q96QU8;Q96QU8-2	Exportin-6
1	1	13	13.186	4.0882	P03897	NADH-ubiquinone oxidoreductase chain 3
1	1	5.4	26.301	4.0797	O60635	Tetraspanin-1
2	2	12.2	28.338	4.0763	Q9NVZ3;Q9NVZ3-3;Q9NVZ3-4;Q9NVZ3-2	Adaptin ear-binding coat-associated protein 2
3	3	4.1	130.96	4.0751	Q86XI2;Q86XI2-2	Condensin-2 complex subunit G2
3	3	7.7	59.382	4.0724	Q9NUT2-5;Q9NUT2-4;Q9NUT2-3;Q9NUT2-2;Q9NUT2	ATP-binding cassette sub-family B member 8, mitochondrial
3	3	9.7	57.002	4.0698	Q9BV44	THUMP domain-containing protein 3
2	2	11.5	17.4	4.0688	O95807	Transmembrane protein 50A
3	3	9.6	32.943	4.0609	O43772	Mitochondrial carnitine/acylcarnitine carrier protein
5	5	5.8	121.69	4.0609	O14776-2;O14776	Transcription elongation regulator 1
1	1	2.2	67.281	4.0606	O15231-4;O15231-7;O15231;O15231-3;O15231-8;O15231-6	Zinc finger protein 185
1	1	3.5	57.218	4.0562	Q9Y2W6-3;Q9Y2W6	Tudor and KH domain-containing protein

2	2	7.6	38.92	4.0551	Q53F19-2;Q53F19	Uncharacterized protein C17orf85
3	3	11.6	38.946	4.0458	Q9NX46	Poly(ADP-ribose) glycohydrolase ARH3
1	1	4.4	44.021	4.0451	P45983-3;P45983-4	
3	3	5.4	79.573	4.0419	P25098;P35626	Beta-adrenergic receptor kinase 1;Beta-adrenergic receptor kinase 2
5	5	11.3	48.547	4.0382	Q15070;Q15070-2	Mitochondrial inner membrane protein OXA1L
3	3	14.7	32.762	4.0341	Q56P03	E2F-associated phosphoprotein
1	1	2	74.217	4.0319	P48449-2;P48449-3;P48449	Lanosterol synthase
3	3	14	19.694	4.0304	P17096-3;P17096	High mobility group protein HMG-I/HMG-Y
3	3	16.6	19.044	4.0235	O14944	Proepiregulin;Epiregulin
2	2	9.2	28.235	4.0226	Q5RKV6	Exosome complex component MTR3
2	2	14.5	17.752	4.0203	P47914	60S ribosomal protein L29
2	2	12.1	26.22	4.0133	Q15011-3;Q15011-4;Q15011-2;Q15011	Homocysteine-responsive endoplasmic reticulum-resident ubiquitin-like domain member 1 protein
2	2	12.7	22.968	4.0101	Q9Y294	Histone chaperone ASF1A
2	2	6.6	50.494	4.0063	Q9NRX5	Serine incorporator 1
3	3	22.3	14.502	4.0034	Q9Y2R5	28S ribosomal protein S17, mitochondrial
2	2	8.7	21.703	4.0021	Q9HD42	Charged multivesicular body protein 1a
3	3	9.2	35.853	4.0015	P15121	Aldose reductase
2	2	13.8	14.551	4.0007	P42766	60S ribosomal protein L35
1	1	19	6.4069	3.9936	Q8TAD7	Overexpressed in colon carcinoma 1 protein
3	3	4.1	140.26	3.9913	Q9UPT8	Zinc finger CCCH domain-containing protein 4
2	2	11	33.337	3.9899	Q9H9Q4;Q9H9Q4-2	Non-homologous end-joining factor 1
2	2	17.9	17.951	3.9887	P36639-4;P36639-3;P36639-2;P36639	7,8-dihydro-8-oxoguanine triphosphatase
4	4	12.6	47.26	3.9833	Q9GZS1;Q9GZS1-1	DNA-directed RNA polymerase I subunit RPA49
1	1	1.7	88.376	3.983	Q7Z3T8-3;Q7Z3T8	Zinc finger FYVE domain-containing protein 16
2	2	10.4	34.833	3.9774	Q15006	ER membrane protein complex subunit 2
3	3	10.2	33.31	3.9729	Q9BPW8	Protein NipSnap homolog 1
3	3	9.3	58.467	3.9716	Q9BVI4	Nucleolar complex protein 4 homolog
1	1	14.9	9.9836	3.9712	P10620-2;P10620	Microsomal glutathione S-transferase 1
2	2	14.6	15.689	3.9669	Q8N138-4;Q8N138	ORM1-like protein 3
1	1	6.1	26.95	3.9656	sp|orf9BT|;P56749	Claudin-12
4	2	36.6	15.954	3.9655	sp|P02070|;CON__P02070	
2	2	17.3	20.423	3.963	Q9BW83-2;Q9BW83	Intraflagellar transport protein 27 homolog
2	2	13.7	22.417	3.9612	Q9NV56	MRG/MORF4L-binding protein
3	3	19.2	19.294	3.9537	P62487	DNA-directed RNA polymerase II subunit RPB7
2	2	2.7	112.13	3.9418	O94804	Serine/threonine-protein kinase 10
4	4	16.8	23.865	3.938	Q8WUY1	Protein THEM6
2	2	20.8	16.162	3.935	P51452-2;P51452	Dual specificity protein phosphatase 3
1	1	8	18.622	3.9329	P03923	NADH-ubiquinone oxidoreductase chain 6
5	4	7.3	76.87	3.9303	P33121-2;P33121-3;P33121	Long-chain-fatty-acid--CoA ligase 1
1	1	6.2	57.811	3.9281	Q96I34	Protein phosphatase 1 regulatory subunit 16A
2	2	23.9	10.094	3.921	Q9P2X0;Q9P2X0-2	Dolichol-phosphate mannosyltransferase subunit 3
3	3	45.5	6.2484	3.9209	Q9P0U1	Mitochondrial import receptor subunit TOM7 homolog
2	2	4.7	64.056	3.9201	Q96RU3-4;Q96RU3-3;Q96RU3-5;Q96RU3-2;Q96RU3	Formin-binding protein 1
2	2	14.9	20.108	3.9121	O43181	NADH dehydrogenase [ubiquinone] iron-sulfur protein 4, mitochondrial
4	3	10.8	53.969	3.9104	Q9NNW7-2;Q9NNW7;Q9NNW7-4;Q9NNW7-3	Thioredoxin reductase 2, mitochondrial
3	3	10.3	52.15	3.9027	Q9HCE5	N6-adenosine-methyltransferase subunit METTL14
6	1	15.2	51.359	3.9004	Q92692-2	Nectin-2
5	4	14.2	55.405	3.896	P20839;P20839-3;P20839-7;P20839-5;P20839-6;P20839-2;P20839-4	Inosine-5-monophosphate dehydrogenase 1
2	2	8.6	26.251	3.894	O95999	B-cell lymphoma/leukemia 10

1	1	4.3	48.91	3.8909	Q12887	Protoheme IX farnesyltransferase, mitochondrial
2	2	9.2	39.596	3.8897	O95630-2;O95630	STAM-binding protein
1	1	8.6	16.691	3.8889	Q04941	Proteolipid protein 2
1	1	4.3	37.609	3.8878	Q96LT4-2;Q96LT4	Sphingomyelin synthase-related protein 1
2	2	6.4	51.307	3.8843	Q12800-2;Q12800-3;Q12800-4;Q12800	Alpha-globin transcription factor CP2
4	4	9.5	62.615	3.8835	P34913;P34913-2;P34913-3	Bifunctional epoxide hydrolase 2;Cytosolic epoxide hydrolase 2;Lipid-phosphate phosphatase
1	1	2.9	47.34	3.8833	Q6GMV2	SET and MYND domain-containing protein 5
2	2	8.4	35.29	3.8802	Q16342-3;Q16342;Q16342-5;Q16342-2;Q16342-4	Programmed cell death protein 2
3	3	24.2	21.188	3.8795	Q9Y3C1;Q9Y3C1-3;Q9Y3C1-2	Nucleolar protein 16
2	2	25	11.955	3.8787	Q5U5X0	Complex III assembly factor LYRM7
1	1	3.5	35.919	3.8761	Q9NWW5;Q9NWW5-2	Ceroid-lipofuscinosis neuronal protein 6
2	2	11.7	28.783	3.876	Q96EY5;Q96EY5-3;Q96EY5-2	Multivesicular body subunit 12A
2	2	5.1	68.94	3.8752	Q96BJ8-2;Q96BJ8;Q96BJ8-3	Engulfment and cell motility protein 3
2	2	8.3	38.33	3.8749	Q9BV23	Monoacylglycerol lipase ABHD6
5	4	10	66.938	3.8666	Q8IXK2;Q8IXK2-2	Polypeptide N-acetylgalactosaminyltransferase 12
4	4	15.2	40.97	3.8589	P49366;P49366-2;P49366-3	Deoxyhypusine synthase
5	5	6.9	93.532	3.8558	Q8TD16;Q8TD16-2	Protein bicaudal D homolog 2
4	1	62.8	12.823	3.8487	O60739	Eukaryotic translation initiation factor 1b
3	3	8.1	59.573	3.8472	Q9NRG9;Q9NRG9-2	Aladin
3	3	6.8	70.1	3.8352	O94923	D-glucuronyl C5-epimerase
1	1	4.7	32.912	3.833	Q5H8A4-5;Q5H8A4-6;Q5H8A4-4;Q5H8A4-3;Q5H8A4-2;Q5H8A4	GPI ethanolamine phosphate transferase 2
2	2	16.8	13.714	3.8323	P61769	Beta-2-microglobulin;Beta-2-microglobulin form pI 5.3
3	3	13.1	34.463	3.8276	O14773-2;O14773	Tripeptidyl-peptidase 1
4	4	9.2	70.392	3.8208	Q08357	Sodium-dependent phosphate transporter 2
2	2	28.6	7.2454	3.8086	P15954	Cytochrome c oxidase subunit 7C, mitochondrial
4	4	18.1	41.35	3.8044	Q9BWD1;Q9BWD1-2	Acetyl-CoA acetyltransferase, cytosolic
2	2	25	16.297	3.8034	Q9Y547	Intraflagellar transport protein 25 homolog
2	2	5	55.397	3.7975	Q6KCM7-2;Q6KCM7-3;Q6KCM7;Q6KCM7-4;Q6KCM7-5	Calcium-binding mitochondrial carrier protein SCaMC-2
3	3	19.1	31.321	3.7969	O60256-4;O60256-2;O60256;O60256-3	Phosphoribosyl pyrophosphate synthase-associated protein 2
2	2	7.9	26.899	3.7967	O75190-2;O75190-3;O75190	DnaJ homolog subfamily B member 6
2	2	21.4	11.402	3.7955	O43676	NADH dehydrogenase [ubiquinone] 1 beta subcomplex subunit 3
2	2	2.9	138.67	3.7925	Q66K14-2;Q66K14	TBC1 domain family member 9B
3	2	14.9	31.698	3.7906	Q96GD0	Pyridoxal phosphate phosphatase
4	4	16.8	44.863	3.7894	Q9GZZ9;Q9GZZ9-2	Ubiquitin-like modifier-activating enzyme 5
2	2	7	35.832	3.7887	Q9Y256	CAAX prenyl protease 2
2	2	5.2	52.488	3.787	P10619-2;P10619	Lysosomal protective protein;Lysosomal protective protein 32 kDa chain;Lysosomal protective protein 20 kDa chain
2	2	6.8	41.905	3.7811	Q969N2-3;Q969N2-4;Q969N2-6;Q969N2-5;Q969N2;Q969N2-2	GPI transamidase component PIG-T
2	2	8.3	50.519	3.7786	Q13325-2;Q13325	Interferon-induced protein with tetratricopeptide repeats 5
5	5	8.6	72.235	3.7683	Q5SNT2;Q5SNT2-2	Transmembrane protein 201
3	3	6.8	81.746	3.7649	Q7Z2T5	TRMT1-like protein
1	1	17.3	9.1703	3.764	Q9NSA3	Beta-catenin-interacting protein 1
4	4	18.2	29.129	3.7615	Q96EU6-2;Q96EU6	Ribosomal RNA processing protein 36 homolog
3	3	6.2	66.99	3.7483	Q5VW38;Q5VW38-2;Q5VW38-3	Protein GPR107
2	2	14.5	26.457	3.7482	Q9BRQ6	MICOS complex subunit MIC25
4	4	16.2	40.241	3.7445	Q14320	Protein FAM50A
5	4	4.4	158.17	3.7392	Q13464	Rho-associated protein kinase 1

1	1	11	18.901	3.7375	Q9NW68-5;Q9NW68-2;Q9NW68;Q9NW68-7;Q9NW68-3	BSD domain-containing protein 1
2	2	14.2	16.073	3.737	Q9P0M9	39S ribosomal protein L27, mitochondrial
2	2	1.2	211.06	3.7327	Q9UIW2;Q9HCM2-4;Q9HCM2	Plexin-A1;Plexin-A4
3	3	3.6	126.55	3.7318	Q9Y613	FH1/FH2 domain-containing protein 1
2	2	7.7	34.814	3.7297	P18065	Insulin-like growth factor-binding protein 2
1	1	14.4	14.604	3.7282	Q7Z422-2;Q7Z422-4;Q7Z422-3;Q7Z422-5;Q7Z422	SUZ domain-containing protein 1
2	2	9.4	26.277	3.726	Q9NX57	Ras-related protein Rab-20
1	1	2.9	49.6	3.7223	Q9H7H0-2;Q9H7H0;Q9H7H0-3	Methyltransferase-like protein 17, mitochondrial
2	2	5	88.975	3.722	Q9UQR1;Q9UQR1-2	Zinc finger protein 148
2	2	6.4	59.612	3.7082	Q9NPQ8-4;Q9NPQ8;Q9NPQ8-3;Q9NPQ8-2	Synembryn-A
5	3	3.8	196.59	3.7025	O14646-2;O14646	Chromodomain-helicase-DNA-binding protein 1
3	3	17.5	25.728	3.6949	Q969E2;Q969E2-2;Q969E2-3	Secretory carrier-associated membrane protein 4
1	1	3.3	58.612	3.6875	Q9Y426-2;Q9Y426-3;Q9Y426	C2 domain-containing protein 2
3	3	5.1	77.242	3.6809	Q9UBB6-2;Q9UBB6;Q9UBB6-3	Neurochondrin
3	3	31.1	11.801	3.6752	Q96FQ6	Protein S100-A16
2	2	4.2	86.908	3.6742	P18074	TFIIH basal transcription factor complex helicase XPD subunit
3	3	9.3	56.223	3.6733	Q96SZ6-2;Q96SZ6-6;Q96SZ6-3;Q96SZ6;Q96SZ6-5;Q96SZ6-4	CDK5 regulatory subunit-associated protein 1
4	4	15.4	50.559	3.6678	Q9UJM3	ERBB receptor feedback inhibitor 1
2	2	18.5	20.116	3.6677	P82663	28S ribosomal protein S25, mitochondrial
2	2	3.9	75.251	3.6579	Q8WZA1;Q8WZA1-2	Protein O-linked-mannose beta-1,2-N-acetylglucosaminyltransferase 1
1	1	6.4	17.271	3.6547	P08962-3;P08962-2;P08962	CD63 antigen
3	3	4.8	96.753	3.6511	Q9ULE6	Paladin
2	2	16.3	19.393	3.6493	P10109	Adrenodoxin, mitochondrial
1	1	5.8	22.202	3.6492	Q9UM19	Hippocalcin-like protein 4
3	3	8.4	64.706	3.6477	P41214;P41214-2	Eukaryotic translation initiation factor 2D
2	2	26.5	12.172	3.6393	Q9BRT8-4;Q9BRT8-2;Q9BRT8-3;Q9BRT8;Q8IUF1;Q5RIA9-3;Q5JTY5-2;Q5RIA9;Q5JTY5;Q4V339	COBW domain-containing protein 1;COBW domain-containing protein 2;COBW domain-containing protein 5;COBW domain-containing protein 3;COBW domain-containing protein 6
4	4	14.2	41.731	3.6378	P49585	Choline-phosphate cytidylyltransferase A
3	3	22.5	20.692	3.6343	Q9BYD1	39S ribosomal protein L13, mitochondrial
4	3	10.3	41.569	3.6323	Q92747;Q92747-2	Actin-related protein 2/3 complex subunit 1A
2	2	5.7	51.328	3.6247	O43194	G-protein coupled receptor 39
2	2	2.6	139.56	3.6246	P54098	DNA polymerase subunit gamma-1
4	4	7.1	65.071	3.6221	Q92696	Geranylgeranyl transferase type-2 subunit alpha
1	1	2	74.469	3.6152	Q9NZ52-2;Q9NZ52	ADP-ribosylation factor-binding protein GGA3
3	3	26.3	20.616	3.615	P82912;P82912-2	28S ribosomal protein S11, mitochondrial
1	1	3.5	58.266	3.6119	Q9Y5Q8-2;Q9Y5Q8;Q9Y5Q8-3	General transcription factor 3C polypeptide 5
4	4	12.3	55.686	3.6084	P31749;P31749-2	RAC-alpha serine/threonine-protein kinase
3	1	7.3	53.651	3.6035	P08670	Vimentin
1	1	3.6	54.545	3.6018	Q5SR56	Hippocampus abundant transcript-like protein 1
1	1	17	17.804	3.6012	Q8WV19	Vesicle transport protein SFT2A
2	2	7.3	57.885	3.6009	Q9HCM4-2;Q9HCM4-4;Q9HCM4-3;Q9HCM4	Band 4.1-like protein 5
1	1	5.1	34.358	3.597	Q96E29-3;Q96E29-2;Q96E29	Transcription termination factor 3, mitochondrial
2	2	3.1	101.85	3.5957	Q01831-2;Q01831	DNA repair protein complementing XP-C cells
1	1	5.2	28.272	3.5904	Q8N2A8	Mitochondrial cardiolipin hydrolase
2	2	11.6	27.209	3.5822	Q7Z309-4;Q7Z309;Q7Z309-2;Q7Z309-3;Q7Z309-5	Protein FAM122B
1	1	4.7	42.216	3.5792	Q15532-2;Q15532	Protein SSXT

1	1	3.5	41.456	3.5718	Q8NEZ2-2;Q8NEZ2	Vacuolar protein sorting-associated protein 37A
2	2	8.6	28.081	3.5669	Q8N4L2	Type 2 phosphatidylinositol 4,5-bisphosphate 4-phosphatase
2	2	19.2	11.261	3.5648	P07311	Acylphosphatase-1
4	4	6	101.97	3.5519	Q15031	Probable leucine--tRNA ligase, mitochondrial
3	3	15	35.079	3.5518	Q6UXN9	WD repeat-containing protein 82
2	2	4	64.473	3.547	Q86U44;Q86U44-2	N6-adenosine-methyltransferase 70 kDa subunit
2	2	9.8	31.484	3.5463	Q9UGV2-3;Q9UGV2-2;Q9UGV2	Protein NDRG3
3	3	7.3	56.307	3.5449	Q5T3F8-2;Q5T3F8-3;Q5T3F8	CSC1-like protein 2
2	2	4.7	66.595	3.541	O43663-2;O43663-4;O43663;O43663-3	Protein regulator of cytokinesis 1
3	3	7.5	50.83	3.535	Q9HB40;Q9HB40-2	Retinoid-inducible serine carboxypeptidase
4	4	16.7	32.447	3.5322	Q9H1Y0	Autophagy protein 5
4	3	7.3	70.472	3.5294	Q08379-2;Q08379	Golgin subfamily A member 2
4	4	6.1	80.214	3.5259	P08582	Melanotransferrin
2	2	28.2	13.735	3.5245	O14907	Tax1-binding protein 3
2	2	10.2	29.937	3.5206	O15194-2;O15194	CTD small phosphatase-like protein
2	2	19.9	15.086	3.5158	O43716	Glutamyl-tRNA(Gln) amidotransferase subunit C, mitochondrial
4	4	19.4	29.172	3.5123	Q14331	Protein FRG1
3	3	3.4	115.18	3.5002	Q8IX01-4;Q8IX01-3;Q8IX01	SURP and G-patch domain-containing protein 2
3	3	2.1	244.29	3.4988	Q8N201	Integrator complex subunit 1
2	2	8.3	37.763	3.4905	Q9Y375	Complex I intermediate-associated protein 30, mitochondrial
1	1	10.8	16.837	3.4857	O95471-2;O95471	Claudin-7
5	4	16	43.066	3.4824	P13861-2;P13861	cAMP-dependent protein kinase type II-alpha regulatory subunit
3	3	11.7	49.404	3.4815	Q9H9T3-4;Q9H9T3-5;Q9H9T3-2;Q9H9T3	Elongator complex protein 3
1	1	5.3	30.855	3.4756	Q8WV22	Non-structural maintenance of chromosomes element 1 homolog
2	2	4.3	67.404	3.4751	P50895	Basal cell adhesion molecule
3	3	5	96.819	3.4684	Q9UBB9	Tuftelin-interacting protein 11
4	4	3.3	180.74	3.4665	Q7Z4S6-6;Q7Z4S6-3;Q7Z4S6-5;Q7Z4S6-2;Q7Z4S6;Q7Z4S6-4	Kinesin-like protein KIF21A
4	4	14.9	33.805	3.4641	Q16775;Q16775-2	Hydroxyacylglutathione hydrolase, mitochondrial
3	2	5.1	72.639	3.4603	Q8IUH5;Q8IUH5-3	Palmitoyltransferase ZDHHC17
2	2	10.4	31.635	3.4477	O75569-3;O75569-2;O75569	Interferon-inducible double-stranded RNA-dependent protein kinase activator A
1	1	8	24.893	3.4413	Q96B77	Transmembrane protein 186
2	2	6.5	48.379	3.4412	Q8NFW8;Q8NFW8-2	N-acylneuraminate cytidylyltransferase
2	2	10.1	32.477	3.4397	Q9BSH4	Translational activator of cytochrome c oxidase 1
1	1	13.3	9.3926	3.4387	P05204	Non-histone chromosomal protein HMG-17
2	2	35.3	7.9916	3.4324	P03928	ATP synthase protein 8
8	2	10.4	109.13	3.4291	Q93084-4;Q93084-2;Q93084-3;Q93084-7;Q93084;Q93084-6;Q93084-5	Sarcoplasmic/endoplasmic reticulum calcium ATPase 3
3	3	24.8	14.226	3.4275	P82932	28S ribosomal protein S6, mitochondrial
11	1	35.6	50.509	3.4267	P50579-2	Methionine aminopeptidase 2
2	2	16.3	22.492	3.4179	P52943;P52943-2	Cysteine-rich protein 2
2	2	2.2	133.28	3.4144	P23458	Tyrosine-protein kinase JAK1
2	2	4.3	80.22	3.4017	Q8IX18-4;Q8IX18-3;Q8IX18;Q8IX18-2	Probable ATP-dependent RNA helicase DHX40
1	1	8.7	23.279	3.3986	Q13158	FAS-associated death domain protein
2	2	11.2	40.817	3.3953	Q9NZN8-2;Q9NZN8-4;Q9NZN8	CCR4-NOT transcription complex subunit 2
3	3	4.9	78.921	3.3947	Q32P28-4;Q32P28;Q32P28-3;Q32P28-2	Prolyl 3-hydroxylase 1
2	2	27	13.104	3.3946	Q9BT73	Proteasome assembly chaperone 3
3	3	5.8	83.593	3.3913	Q6UWE0;Q6UWE0-2	E3 ubiquitin-protein ligase LRSAM1
3	3	7.1	75.965	3.3912	Q6YHU6-4;Q6YHU6-2;Q6YHU6-3;Q6YHU6	Thyroid adenoma-associated protein
2	2	39.4	10.718	3.3894	Q8NI22-2;Q8NI22-3;Q8NI22	Multiple coagulation factor deficiency protein 2
2	2	16.1	22.432	3.3889	Q68D91-2;Q68D91	Metallo-beta-lactamase domain-containing protein 2

1	1	4.7	42.456	3.3854	Q86XS8-2;Q86XS8	E3 ubiquitin-protein ligase RNF130
1	1	4.7	41.036	3.3816	Q9BUA3	Uncharacterized protein C11orf84
4	4	13	32.736	3.3799	Q9Y619	Mitochondrial ornithine transporter 1
6	1	28.5	31.079	3.3791	Q8N2F6-3;Q8N2F6	Armadillo repeat-containing protein 10
3	3	8.1	50.878	3.3765	P26572	Alpha-1,3-mannosyl-glycoprotein 2-beta-N-acetylglucosaminyltransferase
1	1	7.7	20.434	3.3727	A0A024RBG1;Q9NZJ9;Q9NZJ9-2	Diphosphoinositol polyphosphate phosphohydrolase 2
4	4	9.2	64.18	3.3684	Q96DX4	RING finger and SPRY domain-containing protein 1
2	2	4.4	73.91	3.3655	Q9H4E7	Differentially expressed in FDCP 6 homolog
2	2	11.2	26.133	3.3638	sp|Q3Y5Z3|;CON__Q3Y5Z3	
2	2	9	39.992	3.3631	Q8IWE2-2;Q8IWE2	Protein NOXP20
2	2	8.9	28.825	3.3454	Q9Y5Y2	Cytosolic Fe-S cluster assembly factor NUBP2
1	1	4.8	38.95	3.3433	Q96AY3-2;Q96AY3	Peptidyl-prolyl cis-trans isomerase FKBP10
3	3	3.3	145.89	3.3394	Q9C0J8	pre-mRNA 3 end processing protein WDR33
2	2	13.9	20.324	3.3324	P53801	Pituitary tumor-transforming gene 1 protein-interacting protein
20	1	8.8	317.7	3.3255	P50851-2	Lipopolysaccharide-responsive and beige-like anchor protein
2	2	5.5	52.356	3.3237	O95363	Phenylalanine--tRNA ligase, mitochondrial
4	2	16.7	32.307	3.3191	Q15014	Mortality factor 4-like protein 2
1	1	4	47.355	3.3155	Q92947-2;Q92947	Glutaryl-CoA dehydrogenase, mitochondrial
3	3	1.1	436.97	3.3119	Q9NZJ4-2;Q9NZJ4	Sacsin
2	2	7.1	60.519	3.3097	Q96FL9-2;Q96FL9-4;Q96FL9;Q96FL9-3	Polypeptide N-acetylgalactosaminyltransferase 14
2	2	2	160.79	3.3083	Q92729-3;Q92729-2;Q92729-4;Q92729	Receptor-type tyrosine-protein phosphatase U
1	1	3.9	61.438	3.301	O95365	Zinc finger and BTB domain-containing protein 7A
2	2	4.7	53.688	3.2796	P04066	Tissue alpha-L-fucosidase
1	1	9.1	23.583	3.2774	sp|P02666|;CON__P02666	
1	1	8.1	20.648	3.2773	Q9UI14	Prenylated Rab acceptor protein 1
1	1	3.4	56.452	3.2718	Q9NZI7-4;Q9NZI7	Upstream-binding protein 1
3	3	19.1	19.064	3.2689	Q6DKI1-2;Q6DKI1	60S ribosomal protein L7-like 1
4	4	6.4	93.619	3.2618	Q9UKV8-2;Q9UKV8;Q9H9G7-2;Q9UL18;Q9H9G7;Q9HCK5	Protein argonaute-2;Protein argonaute-3;Protein argonaute-1;Protein argonaute-4
6	2	14.8	46.397	3.2527	sp|A2I7N1|;sp|A2I7|;CON__A2I7N1;CON__A2I7N0	
1	1	8.2	23.23	3.2486	Q9H0R3	Transmembrane protein 222
1	1	12.1	13.081	3.2469	Q96K19-5;Q96K19-3;Q96K19-2;Q96K19	E3 ubiquitin-protein ligase RNF170
1	1	2.8	74.138	3.2454	Q9NPI1;Q9NPI1-2	Bromodomain-containing protein 7
2	2	4.8	60.632	3.2441	Q9NPR9	Protein GPR108
3	3	15.2	33.688	3.244	Q9NZ63	Uncharacterized protein C9orf78
3	3	4.4	106.9	3.243	Q9P0K7-4;Q9P0K7-3;Q9P0K7;Q9P0K7-2	Ankycorbin
3	3	10.8	32.214	3.2411	Q5VST6;Q5VST6-2	Alpha/beta hydrolase domain-containing protein 17B
2	2	4.9	75.483	3.2335	Q5PRF9	Protein Smaug homolog 2
3	3	6.6	86.195	3.2291	Q9HAU4	E3 ubiquitin-protein ligase SMURF2
3	3	12.4	32.509	3.2286	Q9NVV0	Trimeric intracellular cation channel type B
3	3	12.7	30.288	3.2271	O95456-2;O95456	Proteasome assembly chaperone 1
4	4	45.6	11.428	3.225	O75964	ATP synthase subunit g, mitochondrial
3	3	10.6	38.087	3.2241	Q8IZV5	Retinol dehydrogenase 10
3	3	12.4	45.679	3.2214	Q6IA17	Single Ig IL-1-related receptor
4	4	6.1	75.042	3.2202	O00139-2;O00139-1;O00139-5;O00139;O00139-4	Kinesin-like protein KIF2A
2	2	15.4	24.418	3.2199	Q8WZ82	Ovarian cancer-associated gene 2 protein
3	3	6.1	66.041	3.2164	O60942-2;O60942;O60942-3;O60942-4	mRNA-capping enzyme;Polynucleotide 5-triphosphatase;mRNA guanylyltransferase
2	2	16.8	14.254	3.212	O15243	Leptin receptor gene-related protein
2	2	1.4	183.71	3.2082	Q9P2K8-2;Q9P2K8	Eukaryotic translation initiation factor 2-alpha kinase 4

2	2	2.2	132.47	3.2074	Q5T1M5-2;Q5T1M5	FK506-binding protein 15
1	1	5.1	29.617	3.2068	Q9UHF1	Epidermal growth factor-like protein 7
2	2	10	27.129	3.1949	Q5T1C6	Acyl-coenzyme A thioesterase THEM4
4	4	30	16.383	3.1932	Q9Y5T4	DnaJ homolog subfamily C member 15
3	3	5.6	83.066	3.1921	Q9Y6D9;Q9Y6D9-3	Mitotic spindle assembly checkpoint protein MAD1
2	2	3.9	89.082	3.1865	Q9H9E3;Q9H9E3-2;Q9H9E3-3	Conserved oligomeric Golgi complex subunit 4
3	3	15	39.248	3.1843	Q15814	Tubulin-specific chaperone C
2	2	2.7	111.48	3.1838	Q9H2U1-3;Q9H2U1-2;Q9H2U1	ATP-dependent RNA helicase DHX36
2	2	5	51.113	3.1834	sp|P17697|;CON__P17697	
3	3	7.4	71.136	3.1832	Q9NV88-3;Q9NV88-2;Q9NV88	Integrator complex subunit 9
3	3	20.3	30.338	3.1793	Q9Y287;Q9Y287-2	Integral membrane protein 2B;BRI2, membrane form;BRI2 intracellular domain;BRI2C, soluble form;Bri23 peptide
1	1	6.4	21.918	3.1733	Q9Y3Y2-4;Q9Y3Y2;Q9Y3Y2-3	Chromatin target of PRMT1 protein
4	2	8.4	66.245	3.1723	Q06787-8;Q06787-6;Q06787-2;Q06787-4;Q06787-5;Q06787-9;Q06787-7;Q06787-3;Q06787;Q06787-11;Q06787-10	Fragile X mental retardation protein 1
4	4	18.5	30.065	3.1624	O95478	Ribosome biogenesis protein NSA2 homolog
2	2	2.3	139.55	3.1614	O60343-2;O60343-3;O60343;O60343-4;O60343-5	TBC1 domain family member 4
3	3	8.8	46.978	3.16	Q9H788-2;Q9H788	SH2 domain-containing protein 4A
5	5	3.2	194.31	3.159	Q9Y5S2	Serine/threonine-protein kinase MRCK beta
3	3	38.6	8.2178	3.1542	P63173	60S ribosomal protein L38
2	2	7.3	56.227	3.1516	Q02252-2;Q02252	Methylmalonate-semialdehyde dehydrogenase [acylating], mitochondrial
2	2	3.5	90.631	3.1516	Q6NUQ1	RAD50-interacting protein 1
2	2	27.9	7.3184	3.1461	P63218	Guanine nucleotide-binding protein G(I)/G(S)/G(O) subunit gamma-5
1	1	14.1	15.489	3.1428	O95139	NADH dehydrogenase [ubiquinone] 1 beta subcomplex subunit 6
2	2	4.2	75.706	3.1388	Q7Z3E5-2;Q7Z3E5	LisH domain-containing protein ARMC9
1	1	8.8	21.508	3.1374	Q13951;Q13951-2	Core-binding factor subunit beta
4	4	6	114.71	3.1357	Q99575	Ribonucleases P/MRP protein subunit POP1
2	2	2.6	107.34	3.1333	Q5JTZ9	Alanine--tRNA ligase, mitochondrial
1	1	13.6	16.73	3.1303	Q14653-3;Q14653-2;Q14653;Q14653-4	Interferon regulatory factor 3
2	2	2.4	91.866	3.1262	P30260;P30260-2	Cell division cycle protein 27 homolog
16	1	24.6	104.71	3.126	A0FGR8-6	Extended synaptotagmin-2
1	1	1.8	61.068	3.1165	P36406-3;P36406-2;P36406	E3 ubiquitin-protein ligase TRIM23
2	2	4.6	51.641	3.116	Q9Y6X5	Bis(5-adenosyl)-triphosphatase ENPP4
2	2	9.3	38.022	3.1141	Q7L592-2;Q7L592	NADH dehydrogenase [ubiquinone] complex I, assembly factor 7
2	2	6.5	43.089	3.1133	Q92604	Acyl-CoA:lysophosphatidylglycerol acyltransferase 1
2	2	10.7	25.577	3.1126	P42696-2;P42696	RNA-binding protein 34
1	1	1.9	96.665	3.1125	Q8TCY9-3;Q8TCY9-4;Q8TCY9-2;Q8TCY9	Up-regulator of cell proliferation
3	3	1.6	221.67	3.1085	Q96PE2	Rho guanine nucleotide exchange factor 17
1	1	8.8	21.444	3.1006	Q9BSY9	Desumoylating isopeptidase 2
1	1	3	50.141	3.0929	Q9NVA4	Transmembrane protein 184C
2	2	8	46.561	3.0911	O75718	Cartilage-associated protein
1	1	8.6	30.156	3.0898	Q13084	39S ribosomal protein L28, mitochondrial
2	2	5.7	46.407	3.0873	Q9HC16	DNA dC->dU-editing enzyme APOBEC-3G
2	2	10.3	33.775	3.087	Q9BW91-2;Q9BW91	ADP-ribose pyrophosphatase, mitochondrial
2	2	10.7	21.834	3.0854	Q9NUP9;Q9HAP6;O14910	Protein lin-7 homolog C;Protein lin-7 homolog B;Protein lin-7 homolog A
1	1	1.9	95.196	3.0817	Q32P44;Q32P44-2	Echinoderm microtubule-associated protein-like 3
3	3	6	60.702	3.0776	P06865	Beta-hexosaminidase subunit alpha
1	1	2.5	67.254	3.0776	Q8IYB7-3;Q8IYB7-2;Q8IYB7	DIS3-like exonuclease 2
3	1	65.6	6.0683	3.0741	P80297	Metallothionein-1X
2	2	50	12.939	3.0717	Q13542	Eukaryotic translation initiation factor 4E-binding protein 2

3	3	19.8	24.635	3.0695	P49795	Regulator of G-protein signaling 19
1	1	5.9	33.245	3.0687	Q8ND76-3;Q8ND76-2;Q8ND76	Cyclin-Y
2	2	2.3	147.09	3.0673	Q5VZK9-2;Q5VZK9	Leucine-rich repeat-containing protein 16A
2	2	2	152.59	3.0641	Q5VT52-2;Q5VT52-3;Q5VT52;Q5VT52-5	Regulation of nuclear pre-mRNA domain-containing protein 2
1	1	2.2	82.317	3.0633	Q9P244	Leucine-rich repeat and fibronectin type III domain-containing protein 1
3	3	5.5	84.34	3.0623	Q9Y2X7;Q9Y2X7-3	ARF GTPase-activating protein GIT1
2	2	3	89.09	3.0607	Q8WUF5	RelA-associated inhibitor
3	3	12.6	29.247	3.0585	P15927;P15927-2;P15927-3	Replication protein A 32 kDa subunit
2	2	4	67.585	3.0576	P10398	Serine/threonine-protein kinase A-Raf
2	2	0	222.76	3.0567	REV__P13535;REV__Q9Y623;REV__Q9UKX2	
2	2	14.3	26.694	3.053	Q14493-2;Q14493	Histone RNA hairpin-binding protein
3	3	3.4	118.49	3.0515	Q5ST30;Q5ST30-4;Q5ST30-2;Q5ST30-3	Valine--tRNA ligase, mitochondrial
1	1	3.2	55.485	3.0513	Q8NEG4	Protein FAM83F
1	1	28.8	5.8998	3.042	P60602-2;P60602	Reactive oxygen species modulator 1
2	2	23.8	9.1275	3.0394	P62312	U6 snRNA-associated Sm-like protein LSm6
1	1	3.5	67.155	3.0389	Q9BU23-3;Q9BU23-2;Q9BU23	Lipase maturation factor 2
3	3	12	33.535	3.0382	P49406	39S ribosomal protein L19, mitochondrial
3	3	16.7	23.135	3.0381	Q16595	Frataxin, mitochondrial;Frataxin intermediate form;Frataxin(56-210);Frataxin(78-210);Frataxin mature form
2	2	7.5	51.582	3.0298	Q9NYL2-2;Q9NYL2-3;Q9NYL2	Mitogen-activated protein kinase kinase kinase MLT
3	3	6	68.997	3.0297	Q9UH65	Switch-associated protein 70
3	3	8	56.361	3.0289	Q96ME7-2;Q96ME7-3;Q96ME7	Zinc finger protein 512
2	2	6.2	58.143	3.0277	Q8TCT8	Signal peptide peptidase-like 2A
2	2	28.1	16.185	3.0265	P32320	Cytidine deaminase
1	1	4	45.228	3.0256	Q9C0D9	Ethanolaminephosphotransferase 1
2	2	10.4	25.098	3.0249	Q9BY43;Q9BY43-2	Charged multivesicular body protein 4a
3	3	7.7	63.922	3.0243	Q14534	Squalene monooxygenase
1	1	1.8	83.734	3.0231	Q5W0V3-2;Q5W0V3	Protein FAM160B1
4	2	14.3	42.429	3.0201	Q06587	E3 ubiquitin-protein ligase RING1
2	2	0.8	394.46	3.0186	P46939;P46939-2;P46939-3;P46939-4	Utrophin
2	2	5.5	60.282	3.0164	P51687	Sulfite oxidase, mitochondrial
1	1	5	40.245	3.0156	A0PK00	Transmembrane protein 120B
2	2	8.7	45.296	3.0139	sp|Q9N2I2|;CON__Q9N2I2	
1	1	7	27.383	3.0126	Q96B36	Proline-rich AKT1 substrate 1
3	1	23.6	12.441	3.0108	P83881	60S ribosomal protein L36a
1	1	3.2	51.289	3.008	P49005	DNA polymerase delta subunit 2
3	3	8.5	46.596	3.0063	Q96FV2;Q96FV2-2	Secernin-2
1	1	19.8	9.2227	3.0047	Q6UWS5	Protein PET117 homolog, mitochondrial
2	2	6.4	38.121	2.9953	Q5MNZ6	WD repeat domain phosphoinositide-interacting protein 3
1	1	12	15.263	2.9845	Q9H1K1-2;Q9H1K1	Iron-sulfur cluster assembly enzyme ISCU, mitochondrial
3	3	25.9	22.318	2.984	Q9Y3A3-2;Q9Y3A3-3;Q9Y3A3	MOB-like protein phocein
2	2	5.5	54.089	2.9689	Q96I59;Q96I59-2	Probable asparagine--tRNA ligase, mitochondrial
1	1	10.1	23.612	2.9666	Q96NC0	Zinc finger matrin-type protein 2
3	3	22.2	23.255	2.9643	P49914;P49914-2	5-formyltetrahydrofolate cyclo-ligase
1	1	0.4	377.59	2.9536	Q70CQ2-3;Q70CQ2-2;Q70CQ2	Ubiquitin carboxyl-terminal hydrolase 34
3	3	8.4	46.228	2.9483	CON__Q95121;sp|Q95121|;P36955	Pigment epithelium-derived factor
2	2	10.6	21.614	2.9438	O75915	PRA1 family protein 3
2	2	9.2	28.808	2.938	Q86VU5	Catechol O-methyltransferase domain-containing protein 1
3	3	20.8	29.233	2.9377	Q9BSF4	Uncharacterized protein C19orf52
3	3	13.2	29.426	2.937	Q9UMY1;Q9UMY1-2	Nucleolar protein 7
2	2	3.7	90.402	2.9351	Q8IWW6-3;Q8IWW6-2;Q8IWW6-4;Q8IWW6	Rho GTPase-activating protein 12
2	2	3.6	74.354	2.925	Q7L2J0;Q7L2J0-2	7SK snRNA methylphosphate capping enzyme

1	1	7.1	21.924	2.9205	Q9H6L4	Armadillo repeat-containing protein 7
4	4	8.1	62.551	2.9174	Q5T9L3-2;Q5T9L3;Q5T9L3-3	Protein wntless homolog
3	3	6.3	72.615	2.909	Q9UBT7-3;Q9UBT7-2;Q9UBT7	Alpha-catulin
1	1	2.1	65.971	2.9088	Q13416	Origin recognition complex subunit 2
2	2	8.1	35.766	2.9035	P08138-2;P08138	Tumor necrosis factor receptor superfamily member 16
4	4	7.8	74.582	2.902	Q7Z4Q2;Q7Z4Q2-2;Q7Z4Q2-3	HEAT repeat-containing protein 3
3	3	7.3	55.468	2.8997	Q9NRY5	Protein FAM114A2
4	4	16.2	33.601	2.8965	Q6P1N9;Q6P1N9-2	Putative deoxyribonuclease TATDN1
31	1	48.1	83.548	2.8948	Q16891-4	MICOS complex subunit MIC60
2	2	7.5	39.038	2.8904	P50613	Cyclin-dependent kinase 7
1	1	4.2	45.551	2.8888	Q8TAF3-5;Q8TAF3-4;Q8TAF3-3;Q8TAF3	WD repeat-containing protein 48
1	1	2.8	65.19	2.8873	sp|E37665|;CON__ENSEMBL:ENSBTAP00000037665	
3	3	12.7	44.803	2.8864	Q9NR50-3;Q9NR50-2;Q9NR50	Translation initiation factor eIF-2B subunit gamma
3	3	4.5	94.369	2.8856	P98175-4;P98175-3;P98175-2;P98175;P98175-5	RNA-binding protein 10
1	1	1.4	101.36	2.8842	Q5T601	Probable G-protein coupled receptor 110
1	1	11.6	19.471	2.8819	O95989	Diphosphoinositol polyphosphate phosphohydrolase 1
3	3	16.5	21.175	2.8812	Q9UM00-1;Q9UM00-2;Q9UM00	Transmembrane and coiled-coil domain-containing protein 1
1	1	2.6	63.747	2.8799	O00255-3;O00255-2;O00255	Menin
2	2	2.3	127.85	2.8796	Q9NSV4-7;Q9NSV4-4;Q9NSV4;Q9NSV4-6;Q9NSV4-5	Protein diaphanous homolog 3
4	4	9.1	64.969	2.8767	P49902;P49902-2	Cytosolic purine 5-nucleotidase
3	3	16.2	18.795	2.8766	Q969H8	Myeloid-derived growth factor
2	2	7.1	38.187	2.8757	Q9Y5B8-2;Q9Y5B8	Nucleoside diphosphate kinase 7
1	1	2.7	63.571	2.8744	Q5T0N5-3;Q5T0N5-4;Q5T0N5-2;Q5T0N5-5;Q5T0N5	Formin-binding protein 1-like
3	3	12.6	47.744	2.8731	O75648;O75648-2	Mitochondrial tRNA-specific 2-thiouridylase 1
6	1	2.9	268.31	2.8723	O15020-2;O15020	Spectrin beta chain, non-erythrocytic 2
1	1	4.4	39.834	2.8712	P34949-2;P34949	Mannose-6-phosphate isomerase
3	3	11.2	40.11	2.8641	Q9H9Y2	Ribosome production factor 1
6	1	6.3	121.14	2.8638	P28370-2;P28370	Probable global transcription activator SNF2L1
2	2	2.5	103.57	2.8637	Q96CW5;Q96CW5-2;Q96CW5-3	Gamma-tubulin complex component 3
2	2	16.2	19.015	2.8609	Q96EL2	28S ribosomal protein S24, mitochondrial
9	1	9.6	65.84	2.8593	Q01546;sp|Q01546|;CON__Q01546;sp|P12035|;CON__P12035;P12035	Keratin, type II cytoskeletal 2 oral;Keratin, type II cytoskeletal 3
1	1	35.1	6.2107	2.8589	Q8WXC6	Myeloma-overexpressed gene 2 protein
1	1	7.6	27.963	2.8548	P13284	Gamma-interferon-inducible lysosomal thiol reductase
2	2	6.1	60.846	2.8543	Q13438-8;Q13438-6;Q13438-5;Q13438-3;Q13438-2;Q13438-7;Q13438-4;Q13438	Protein OS-9
1	1	4.2	36.044	2.8467	Q6UWP7-2;Q6UWP7-3;Q6UWP7	Lysocardiolipin acyltransferase 1
4	3	16.5	33.729	2.8419	P11802;P11802-2	Cyclin-dependent kinase 4
1	1	16.1	9.53	2.8412	Q9BUV8-4;Q9BUV8-3;Q9BUV8-2;Q9BUV8;Q9BUV8-5	Uncharacterized protein C20orf24
4	4	19.1	38.478	2.8393	Q12972;Q12972-2	Nuclear inhibitor of protein phosphatase 1;Activator of RNA decay
3	3	21.1	20.082	2.8374	P62330	ADP-ribosylation factor 6
1	1	5.1	33.915	2.8349	O95755-2;O95755	Ras-related protein Rab-36
3	3	11.3	43.641	2.8332	Q8NAX2	Keratinocyte differentiation factor 1
4	4	1.3	339.59	2.8318	O94915	Protein furry homolog-like
1	1	4.7	35.657	2.828	P20226-2;P20226	TATA-box-binding protein
2	2	2.3	133.63	2.8274	O95487-2;O95487;O95487-3	Protein transport protein Sec24B
2	2	6.6	42.104	2.8262	Q3KQU3-3;Q3KQU3-2;Q3KQU3-4;Q3KQU3	MAP7 domain-containing protein 1

3	3	7.1	57.152	2.8247	Q04771	Activin receptor type-1
2	2	6.6	36.187	2.8245	Q06136	3-ketodihydrosphingosine reductase
1	1	9.8	17.371	2.822	Q9P0S3;Q53FV1	ORM1-like protein 1;ORM1-like protein 2
2	2	4	69.118	2.8189	Q9Y5A7-2;Q9Y5A7	NEDD8 ultimate buster 1
3	3	4.7	86.025	2.8162	P51798-2;P51798	H(+)/Cl(-) exchange transporter 7
1	1	15.3	9.638	2.8096	Q7Z4G1;Q7Z4G1-2	COMM domain-containing protein 6
1	1	28.6	9.2787	2.8082	O95167	NADH dehydrogenase [ubiquinone] 1 alpha subcomplex subunit 3
2	2	14.7	21.703	2.8081	Q99828;Q99828-2	Calcium and integrin-binding protein 1
1	1	8.3	16.68	2.8078	Q9BRU9-2;Q9BRU9	rRNA-processing protein UTP23 homolog
3	3	11.7	28.671	2.8076	O94907	Dickkopf-related protein 1
2	2	14	19.206	2.8069	O95881	Thioredoxin domain-containing protein 12
2	2	3.8	73.2	2.8035	Q9NW82	WD repeat-containing protein 70
2	2	4.6	69.476	2.8028	P18887	DNA repair protein XRCC1
2	2	6.7	38.402	2.797	Q9NYP7-2;Q9NYP7;Q9NYP7-3	Elongation of very long chain fatty acids protein 5
3	3	22.7	24.992	2.7917	Q9H7E9;Q9H7E9-2	UPF0488 protein C8orf33
2	2	7.8	46.105	2.787	P32121;P32121-3;P32121-4;P32121-5;P32121-2	Beta-arrestin-2
6	1	47.1	21.229	2.7863	P01111	GTPase NRas
1	1	21.3	10.191	2.7854	Q9NP84-2;Q9NP84	Tumor necrosis factor receptor superfamily member 12A
2	2	4.2	88.946	2.7846	Q6GQQ9-2;Q6GQQ9	OTU domain-containing protein 7B
1	1	6.9	22.317	2.7834	Q9NWD8-2;Q9NWD8	Transmembrane protein 248
2	2	4.6	70.86	2.7804	Q9UFC0	Leucine-rich repeat and WD repeat-containing protein 1
2	2	7.4	40.285	2.7786	Q9NWT6	Hypoxia-inducible factor 1-alpha inhibitor
1	1	8	21.876	2.7752	Q9BV19	Uncharacterized protein C1orf50
1	1	1.1	165.91	2.7752	P09884	DNA polymerase alpha catalytic subunit
3	3	4.6	81.798	2.7744	Q8IYI6	Exocyst complex component 8
2	2	36.8	7.4016	2.773	O00244	Copper transport protein ATOX1
1	1	7	21.427	2.7721	Q9Y248	DNA replication complex GINS protein PSF2
2	2	2.2	117.4	2.7656	Q9UPN4-3;Q9UPN4-2;Q9UPN4	Centrosomal protein of 131 kDa
2	2	3.2	96.766	2.7654	Q9H1B5;Q9H1B5-2	Xylosyltransferase 2
1	1	3.9	33.885	2.7633	Q5SW96	Low density lipoprotein receptor adapter protein 1
3	3	13.9	27.563	2.7571	O43657	Tetraspanin-6
2	2	13.6	24.086	2.757	Q9UJG1;Q9UJG1-2;Q9UJG1-3	Motile sperm domain-containing protein 1
3	3	6.2	86.435	2.7559	Q2TAL8	Glutamine-rich protein 1
4	4	18.5	34.83	2.7547	Q9NWX6	Probable tRNA(His) guanylyltransferase
3	3	45.6	10.18	2.7533	P06703	Protein S100-A6
3	3	8.1	57.891	2.753	Q8TBB5;Q8TBB5-3;Q8TBB5-2	Kelch domain-containing protein 4
2	2	12.3	31.43	2.7524	Q9Y6N1	Cytochrome c oxidase assembly protein COX11, mitochondrial
4	1	11	28.385	2.7488	P09493-5	Tropomyosin alpha-1 chain
3	3	6	89.277	2.7478	P19447	TFIIH basal transcription factor complex helicase XPB subunit
3	3	23.9	20.749	2.7469	Q96EK6	Glucosamine 6-phosphate N-acetyltransferase
2	2	3.7	74.229	2.7423	Q7Z4V5-2;Q7Z4V5;Q7Z4V5-4;Q7Z4V5-3	Hepatoma-derived growth factor-related protein 2
1	1	3.8	48.984	2.7416	Q86X53	Glutamate-rich protein 1
1	1	2.4	62.033	2.7414	Q8N697	Solute carrier family 15 member 4
3	3	33.3	15.004	2.7379	Q4G0I0	Protein CCSMST1
3	3	10.9	33.932	2.7379	Q9NRD1	F-box only protein 6
3	3	30.7	8.7813	2.7345	P09669	Cytochrome c oxidase subunit 6C
4	4	1.2	447.75	2.7259	Q99996-5;Q99996-3;Q99996;Q99996-1;Q99996-6;Q99996-4	A-kinase anchor protein 9
1	1	9.6	16.694	2.7208	Q9GZY4	Cytochrome c oxidase assembly factor 1 homolog
2	2	24.6	13.635	2.7203	P62837-2;P62837;P61077;P61077-2;P61077-3;Q9Y2X8;P51668	Ubiquitin-conjugating enzyme E2 D2;Ubiquitin-conjugating enzyme E2 D3;Ubiquitin-conjugating enzyme E2 D4;Ubiquitin-conjugating enzyme E2 D1

4	4	18	41.45	2.718	O75683	Surfeit locus protein 6
1	1	6.7	25.843	2.716	Q86YM7-2;Q86YM7	Homer protein homolog 1
3	3	3.3	98.081	2.7137	Q9Y487	V-type proton ATPase 116 kDa subunit a isoform 2
1	1	1.8	88.134	2.7131	P51178-2	
3	3	5.9	51.58	2.7128	P03905	NADH-ubiquinone oxidoreductase chain 4
2	2	7.5	52.901	2.7092	O60678-2;O60678	Protein arginine N-methyltransferase 3
3	3	16	20.05	2.7064	Q9NRX2	39S ribosomal protein L17, mitochondrial
4	4	8.3	51.457	2.7056	Q8WUX1;Q8WUX1-2	Sodium-coupled neutral amino acid transporter 5
2	2	9.4	34.083	2.6971	P54619-2;P54619;P54619-3;Q9UGJ0-2;Q9UGJ0-3;Q9UGJ0	5-AMP-activated protein kinase subunit gamma-1;5-AMP-activated protein kinase subunit gamma-2
2	2	6.2	50.879	2.6961	Q96L92-2;Q96L92-3;Q96L92	Sorting nexin-27
3	3	7.8	62.287	2.6959	O15344-2;O15344	E3 ubiquitin-protein ligase Midline-1
2	2	6.3	40.72	2.6945	Q8WTS6	Histone-lysine N-methyltransferase SETD7
2	2	15.4	18.001	2.6929	P52298;P52298-3;P52298-2	Nuclear cap-binding protein subunit 2
3	3	11.9	33.435	2.6836	Q96BW5-2;Q96BW5	Phosphotriesterase-related protein
2	2	3.9	72.456	2.6814	Q8N8A6	ATP-dependent RNA helicase DDX51
1	1	0.5	300.44	2.6794	P25054-2;P25054-3;P25054	Adenomatous polyposis coli protein
3	3	15.1	34.592	2.6788	Q9NQ48;Q9NQ48-3;Q9NQ48-2	Leucine zipper transcription factor-like protein 1
2	2	5	77.869	2.6704	P14859-2;P14859-4;P14859-5;P14859-3;P14859;P14859-6	POU domain, class 2, transcription factor 1
10	1	42.5	47.687	2.6689	Q13501;Q13501-2	Sequestosome-1
2	2	18.8	12.366	2.6684	Q9NPJ3-2;Q9NPJ3	Acyl-coenzyme A thioesterase 13;Acyl-coenzyme A thioesterase 13, N-terminally processed
1	1	10.6	12.551	2.6613	O95182	NADH dehydrogenase [ubiquinone] 1 alpha subcomplex subunit 7
1	1	3.3	38.493	2.6598	O43464-2;O43464-4;O43464-3;O43464	Serine protease HTRA2, mitochondrial
1	1	4.6	30.77	2.6549	Q9H019-3;Q9H019	Mitochondrial fission regulator 1-like
2	2	10	20.274	2.6542	O43617;O43617-2	Trafficking protein particle complex subunit 3
1	1	4	44.312	2.6514	Q9Y3T6-3;Q9Y3T6	R3H and coiled-coil domain-containing protein 1
2	2	11.4	20.848	2.6458	Q5BJF2	Transmembrane protein 97
2	2	9.4	21.542	2.644	Q9BY50	Signal peptidase complex catalytic subunit SEC11C
3	3	6.2	61.058	2.6405	Q7Z3D6-5;Q7Z3D6-4;Q7Z3D6-3;Q7Z3D6;Q7Z3D6-2	UPF0317 protein C14orf159, mitochondrial
3	3	17.3	22.875	2.6404	O75608-2;O75608	Acyl-protein thioesterase 1
1	1	10.2	12.58	2.6382	Q13541	Eukaryotic translation initiation factor 4E-binding protein 1
2	2	13.6	15.864	2.6308	Q9UKR5	Probable ergosterol biosynthetic protein 28
4	4	7.6	99.918	2.6277	Q14527-2;Q14527	Helicase-like transcription factor
2	2	13.1	28.998	2.6253	Q99618	Cell division cycle-associated protein 3
3	3	5.3	114.54	2.6207	Q13683-13;Q13683-9;Q13683-7;Q13683-3;Q13683-10;Q13683	Integrin alpha-7;Integrin alpha-7 heavy chain;Integrin alpha-7 light chain;Integrin alpha-7 70 kDa form
1	1	6.2	22.134	2.6174	Q86YN1-2;Q86YN1	Dolichyldiphosphatase 1
2	2	9.2	24.348	2.6138	sp|P02663|;CON__P02663	
1	1	12.8	14.758	2.6096	Q9NWH2	Transmembrane protein 242
1	1	4.8	24.241	2.6083	sp|P02777|;CON__P02777	
1	1	2.8	47.283	2.607	Q99640-4;Q99640-2;Q99640-3;Q99640	Membrane-associated tyrosine- and threonine-specific cdc2-inhibitory kinase
2	2	2.6	56.421	2.6058	Q9UKD1	Glucocorticoid modulatory element-binding protein 2
2	1	6.8	51.604	2.6	Q96NM4	TOX high mobility group box family member 2
2	2	2	136.4	2.5906	Q2M389;Q2M389-2	WASH complex subunit 7
3	3	7	66.515	2.5833	Q9BYC5;Q9BYC5-2	Alpha-(1,6)-fucosyltransferase
3	3	11	44.223	2.5805	Q9HB90;Q9NQL2	Ras-related GTP-binding protein C;Ras-related GTP-binding protein D
2	2	5.7	55.215	2.5793	Q9UKZ1	CCR4-NOT transcription complex subunit 11
1	1	8.7	21.362	2.5761	Q9NX70-2;Q9NX70	Mediator of RNA polymerase II transcription subunit 29
2	2	2	154.06	2.564	A3KN83-3;A3KN83-2;A3KN83	Protein strawberry notch homolog 1

1	1	6.6	24.107	2.5635	Q96LD8	Sentrin-specific protease 8
2	2	22.4	13.507	2.5588	Q9Y2Q5;Q9Y2Q5-2	Ragulator complex protein LAMTOR2
2	2	9.2	57.829	2.5559	Q8NC44	Protein FAM134A
1	1	17.8	13.888	2.5511	Q8WW01-2;Q8WW01	tRNA-splicing endonuclease subunit Sen15
22	2	41.1	49.448	2.5487	sp|H92931|;CON__H-INV:HIT000292931	
2	2	13.8	30.658	2.5469	Q96CU9-2;Q96CU9-3;Q96CU9	FAD-dependent oxidoreductase domain-containing protein 1
1	1	21.7	9.4618	2.5462	P60002	Transcription elongation factor 1 homolog
2	1	2.9	77.526	2.5455	P78362;P78362-2;Q9UPE1-2;Q9UPE1-3;Q9UPE1-4;Q9UPE1	SRSF protein kinase 2;SRSF protein kinase 2 N-terminal;SRSF protein kinase 2 C-terminal;SRSF protein kinase 3
1	1	6.9	17.778	2.5398	Q969X5-3;Q969X5-2;Q969X5	Endoplasmic reticulum-Golgi intermediate compartment protein 1
1	1	2.8	59.701	2.5389	P85037-2;P85037	Forkhead box protein K1
2	2	1.8	196.71	2.5348	Q9BY89	Uncharacterized protein KIAA1671
14	2	53.6	36.983	2.53	P36873;P36873-2	Serine/threonine-protein phosphatase PP1-gamma catalytic subunit
2	2	14.9	22.808	2.5297	Q6P1X6-2;Q6P1X6	UPF0598 protein C8orf82
2	1	6.8	38.224	2.5289	P17693;P17693-5;P17693-4	HLA class I histocompatibility antigen, alpha chain G
1	1	10.6	12.825	2.5281	Q969H6-2;Q969H6	Ribonuclease P/MRP protein subunit POP5
3	2	12.5	38.023	2.5258	Q969Z3;Q969Z3-2	Mitochondrial amidoxime reducing component 2
3	3	6.4	75.93	2.5209	Q13546;Q13546-2	Receptor-interacting serine/threonine-protein kinase 1
2	1	9.1	31.424	2.5198	Q9BRL6-2;Q9BRL6	Serine/arginine-rich splicing factor 8
3	3	35.6	11.557	2.5187	P63165	Small ubiquitin-related modifier 1
2	2	1.3	175.38	2.5186	Q9NRL2-2;Q9NRL2	Bromodomain adjacent to zinc finger domain protein 1A
2	2	7.4	40.764	2.5171	P00813	Adenosine deaminase
2	2	5.9	55.307	2.5091	Q8WWK9-4;Q8WWK9-6;Q8WWK9-5;Q8WWK9	Cytoskeleton-associated protein 2
1	1	6.7	22.324	2.5071	P62341	Selenoprotein T
1	1	11.9	11.354	2.5057	Q3ZAQ7	Vacuolar ATPase assembly integral membrane protein VMA21
2	2	4.6	53.741	2.5054	O00443-2;O00443	Phosphatidylinositol 4-phosphate 3-kinase C2 domain-containing subunit alpha
1	1	5	35.914	2.4976	O14734	Acyl-coenzyme A thioesterase 8
2	2	14.3	17.201	2.4918	Q9HBL7	Plasminogen receptor (KT)
1	1	3.3	52.88	2.4914	Q00613-2;Q00613	Heat shock factor protein 1
69	1	28.8	367.42	2.4902	Q14789-4	Golgin subfamily B member 1
1	1	7.4	15.709	2.4884	Q8IWT0-2;Q8IWT0	Protein archease
1	1	6.7	22.474	2.4855	Q5SGD2-4;Q5SGD2	Protein phosphatase 1L
4	3	9.7	59.681	2.4853	P21397;P21397-2	Amine oxidase [flavin-containing] A
3	3	53.1	7.7059	2.4827	Q9NPE3	H/ACA ribonucleoprotein complex subunit 3
2	2	3.3	62.402	2.4821	Q7L9B9	Endonuclease/exonuclease/phosphatase family domain-containing protein 1
2	2	6.1	51.964	2.4814	O60427	Fatty acid desaturase 1
1	1	6.6	21.627	2.4794	Q53FT3	Protein Hikeshi
2	2	3.5	78.576	2.4754	Q6IA86-4;Q6IA86-7;Q6IA86-2;Q6IA86-3;Q6IA86-5;Q6IA86;Q6IA86-6	Elongator complex protein 2
2	2	8.8	25.498	2.4719	Q6PI78	Transmembrane protein 65
1	1	3.3	46.668	2.4708	P29353-3;P29353-2;P29353-7;P29353;P29353-6	SHC-transforming protein 1
2	2	14.6	28.728	2.4671	Q9BVC4-3;Q9BVC4-5;Q9BVC4;Q9BVC4-4	Target of rapamycin complex subunit LST8
1	1	2.6	59.367	2.4662	Q9NX95-5;Q9NX95-2;Q9NX95-4;Q9NX95-3;Q9NX95	Syntabulin
1	1	12.5	14.678	2.4642	Q8N6L1;Q8N6L1-2	Keratinocyte-associated protein 2
3	1	23.1	18	2.4584	P51965-2;P51965;P51965-3	Ubiquitin-conjugating enzyme E2 E1
5	1	6.4	61.802	2.4505	sp|Q7Z794|;CON__Q7Z794;Q7Z794	Keratin, type II cytoskeletal 1b
2	2	8.9	34.76	2.4494	Q96GD4-4;Q96GD4-2;Q96GD4;Q96GD4-5;Q96GD4-3	Aurora kinase B
1	1	2.9	87.434	2.448	A8MVW0	Protein FAM171A2
1	1	25.2	11.602	2.4475	Q9UK45	U6 snRNA-associated Sm-like protein LSm7

1	1	0.7	212.27	2.4433	Q9Y4I1-2;Q9ULV0;Q9Y4I1;Q9Y4I1-3	Unconventional myosin-Va;Unconventional myosin-Vb
2	2	24.1	9.6601	2.4385	P61024	Cyclin-dependent kinases regulatory subunit 1
3	3	5.6	97.742	2.437	Q9BZL6-3;Q9BZL6;Q9BZL6-2	Serine/threonine-protein kinase D2
2	2	7.6	36.694	2.4325	O95900;O95900-2	Probable tRNA pseudouridine synthase 2
2	2	19.4	15.876	2.4324	Q86WQ0;Q86WQ0-2	Nuclear receptor 2C2-associated protein
2	2	18.6	16.445	2.4312	P0DI82;P0DI81;P0DI81-3	Trafficking protein particle complex subunit 2B;Trafficking protein particle complex subunit 2
1	1	3.7	39.499	2.431	Q9BPY3	Protein FAM118B
1	1	14.9	12.029	2.428	Q9NZ42	Gamma-secretase subunit PEN-2
2	2	3.8	77.672	2.4258	O15226;O15226-2	NF-kappa-B-repressing factor
1	1	3.4	55.031	2.4249	O75071	EF-hand calcium-binding domain-containing protein 14
2	2	7.1	39.611	2.4244	P48556	26S proteasome non-ATPase regulatory subunit 8
1	1	13.6	9.0714	2.4223	Q15843	NEDD8
1	1	2.9	43.669	2.4223	Q13137-5;Q13137-2;Q13137;Q13137-3;Q13137-4	Calcium-binding and coiled-coil domain-containing protein 2
1	1	12.1	10.758	2.4203	Q9HD34	LYR motif-containing protein 4
3	2	9.5	36.938	2.42	Q00534	Cyclin-dependent kinase 6
1	1	2.1	72.453	2.4195	O00178	GTP-binding protein 1
3	3	13	48.927	2.4176	Q8N0Z6	Tetratricopeptide repeat protein 5
1	1	2.9	62.543	2.4173	Q9P270	SLAIN motif-containing protein 2
2	2	3.9	73.588	2.4156	Q9UER7-3;Q9UER7-5;Q9UER7-4;Q9UER7-2;Q9UER7	Death domain-associated protein 6
1	1	1.2	155.19	2.412	Q9UKJ3-2;Q9UKJ3	G patch domain-containing protein 8
3	3	4.1	124.9	2.4115	Q8IUD2-3;Q8IUD2;Q8IUD2-4;Q8IUD2-2	ELKS/Rab6-interacting/CAST family member 1
1	1	11.9	9.74	2.4095	Q9BTE1-3;Q9BTE1-2;Q9BTE1	Dynactin subunit 5
2	2	6.4	51.129	2.4044	Q6NZY4-2;Q6NZY4	Zinc finger CCHC domain-containing protein 8
1	1	4.9	25.431	2.404	Q8TAA5	GrpE protein homolog 2, mitochondrial
1	1	5.6	25.337	2.4036	Q96D31-2;Q96D31	Calcium release-activated calcium channel protein 1
1	1	1.9	73.747	2.4036	Q9BW19	Kinesin-like protein KIFC1
3	3	13.8	46.571	2.4023	Q5TDH0-3;Q5TDH0	Protein DDI1 homolog 2
3	3	12.1	29.665	2.3984	Q96HV5	Transmembrane protein 41A
1	1	3.2	51.925	2.3954	Q16566	Calcium/calmodulin-dependent protein kinase type IV
1	1	18.4	11.006	2.3951	P01040	Cystatin-A;Cystatin-A, N-terminally processed
1	1	4.1	39.514	2.394	O43251-9;Q9NWB1-3;O43251-4;O43251-5;O43251-10;O43251-2;O43251;Q9NWB1-4;Q9NWB1-5;Q9NWB1;O43251-7;Q9NWB1-2;O43251-6;O43251-8	RNA binding protein fox-1 homolog 2;RNA binding protein fox-1 homolog 1
2	2	3.9	85.99	2.394	Q9NVE7	Pantothenate kinase 4
1	1	4.3	32.172	2.3939	Q9UIC8-3;Q9UIC8;Q9UIC8-2	Leucine carboxyl methyltransferase 1
1	1	12.6	14.03	2.3935	Q9NVS2-3;Q9NVS2;Q9NVS2-2	28S ribosomal protein S18a, mitochondrial
1	1	7.7	31.88	2.393	Q9Y448-2;Q9Y448	Small kinetochore-associated protein
2	2	4	70.678	2.3928	O95834;O95834-2;O95834-3	Echinoderm microtubule-associated protein-like 2
2	2	20.9	12.017	2.3893	Q9BV81	ER membrane protein complex subunit 6
2	2	1.6	143	2.3833	Q15047-3;Q15047	Histone-lysine N-methyltransferase SETDB1
1	1	2.3	67.638	2.3818	Q9H2C0	Gigaxonin
2	2	6.6	50.66	2.3812	sp|IQ0VBK2|;CON__Q0VBK2;sp|Q6KB66-1|;CON__Q6KB66-1;Q6KB66-2;Q6KB66;Q6KB66-3	Keratin, type II cytoskeletal 80
1	1	2.6	65.533	2.3776	Q9UPW0-2;Q9UPW0	Forkhead box protein J3
2	2	22	20.189	2.3703	O94760-2;O94760	N(G),N(G)-dimethylarginine dimethylaminohydrolase 1
3	3	7.8	55.261	2.3676	Q86WB0;Q86WB0-2;Q86WB0-3	Nuclear-interacting partner of ALK
2	2	9.6	29.95	2.3668	P00414	Cytochrome c oxidase subunit 3

1	1	8.1	16.829	2.3644	Q9NRP0;Q9NRP0-2	Oligosaccharyltransferase complex subunit OSTC
1	1	6.1	31.192	2.3616	Q13643	Four and a half LIM domains protein 3
2	2	5	42.717	2.3596	P00156	Cytochrome b
3	1	15.1	20.828	2.3557	Q93045;Q93045-2	Stathmin-2
1	1	4.5	27.679	2.3529	P31944	Caspase-14;Caspase-14 subunit p17, mature form;Caspase-14 subunit p10, mature form;Caspase-14 subunit p20, intermediate form;Caspase-14 subunit p8, intermediate form
2	2	15.8	20.281	2.3494	Q5TC12-2;Q5TC12-3;Q5TC12	ATP synthase mitochondrial F1 complex assembly factor 1
3	3	1.7	316.05	2.3483	Q6KC79;Q6KC79-2	Nipped-B-like protein
2	2	5.2	64.84	2.3468	O14545;O14545-2	TRAF-type zinc finger domain-containing protein 1
2	2	7.2	61.554	2.3465	Q9UK59;Q9UK59-2	Lariat debranching enzyme
3	3	4.2	89.098	2.3461	Q96JM3	Chromosome alignment-maintaining phosphoprotein 1
3	3	12.8	33.264	2.3442	P08397-4;P08397-3;P08397-2;P08397	Porphobilinogen deaminase
5	3	15.9	40.058	2.3426	Q9P0M6	Core histone macro-H2A.2
1	1	1.5	116.95	2.3389	Q01804-5;Q01804-3;Q01804	OTU domain-containing protein 4
4	4	0.2	3653	2.3389	Q8WZ42-5;Q8WZ42-11;Q8WZ42-4;Q8WZ42-7;Q8WZ42-2;Q8WZ42;Q8WZ42-8;Q8WZ42-13;Q8WZ42-12;Q8WZ42-3;Q8WZ42-10;Q8WZ42-9	Titin
2	2	10.6	19.481	2.3381	Q9Y5Z9-2;Q9Y5Z9	UbiA prenyltransferase domain-containing protein 1
3	3	5	97.684	2.3359	O00192-2;O00192	Armadillo repeat protein deleted in velo-cardio-facial syndrome
7	1	16.7	45.771	2.3341	sp|E77550|;CON__ENSEMBL:ENSP00000377550;P13646-3;P13646;sp|IP13646-1|;CON__P13646-1;P13646-2	Keratin, type I cytoskeletal 13
2	2	18.8	19.053	2.333	Q8N9N8	Probable RNA-binding protein EIF1AD
3	3	4.8	105.06	2.3295	P48200	Iron-responsive element-binding protein 2
1	1	5.7	33.395	2.3268	O75792	Ribonuclease H2 subunit A
1	1	8.2	18.409	2.3256	Q8TBK6-2;Q8TBK6	Zinc finger CCHC domain-containing protein 10
2	2	3	111.43	2.3163	Q13625-2;Q13625;Q13625-3	Apoptosis-stimulating of p53 protein 2
1	1	2.2	73.152	2.3161	Q12772-2;Q12772	Sterol regulatory element-binding protein 2;Processed sterol regulatory element-binding protein 2
10	1	39.3	38.15	2.3158	Q15366-6;Q15366-3;Q15366-7;Q15366-4	Poly(rC)-binding protein 2
1	1	2.7	60.145	2.3154	Q9HBI6	Phylloquinone omega-hydroxylase CYP4F11
1	1	3.4	61.978	2.314	Q8IZ69-2;Q8IZ69	tRNA (uracil-5-)-methyltransferase homolog A
2	2	16.3	12.655	2.3023	Q96PC3-3;Q96PC3-4;Q96PC3;Q96PC3-2	AP-1 complex subunit sigma-3
2	2	22.9	10.921	2.2906	Q9NP97;Q8TF09	Dynein light chain roadblock-type 1;Dynein light chain roadblock-type 2
1	1	3.8	29.282	2.2891	Q7Z7H8;Q7Z7H8-2	39S ribosomal protein L10, mitochondrial
1	1	15.9	9.9062	2.282	O14949	Cytochrome b-c1 complex subunit 8
2	2	7.2	54.404	2.2818	P15260	Interferon gamma receptor 1
1	1	11.4	15.733	2.2803	Q8N4P3-2;Q8N4P3	Guanosine-3,5-bis(diphosphate) 3-pyrophosphohydrolase MESH1
1	1	2.3	95.478	2.2803	Q15276-2;Q15276	Rab GTPase-binding effector protein 1
1	1	3.7	54.303	2.279	Q9BZD4	Kinetochore protein Nuf2
4	4	4.1	117.6	2.2789	Q9H4L7-2;Q9H4L7;Q9H4L7-3	SWI/SNF-related matrix-associated actin-dependent regulator of chromatin subfamily A containing DEAD/H box 1
1	1	2.5	83.755	2.2735	Q7Z388	Probable C-mannosyltransferase DPY19L4
1	1	2.1	67.038	2.2726	A2RU67	Uncharacterized protein KIAA1467
2	2	0.7	331.77	2.2725	Q96L91-4;Q96L91-3;Q96L91-5;Q96L91-2;Q96L91	E1A-binding protein p400
1	1	1.9	108.64	2.2701	O94985-2;O94985	Calsyntenin-1;Soluble Alc-alpha;CTF1-alpha
2	2	4.2	70.459	2.2682	Q9H7D7-2;Q9H7D7	WD repeat-containing protein 26
2	2	3.1	82.691	2.2679	Q96MU7-2;Q96MU7	YTH domain-containing protein 1
3	3	2	212.57	2.2649	P35658-2;P35658-4;P35658;P35658-3;P35658-5	Nuclear pore complex protein Nup214
1	1	2.2	64.639	2.2629	P22455-3;P22455-2;P22455	Fibroblast growth factor receptor 4
1	1	6.6	31.636	2.2623	B1AK53-2;B1AK53	Espin
4	4	19.8	25.938	2.2585	Q9HAC8;Q8WUN7	Ubiquitin domain-containing protein 1;Ubiquitin domain-containing protein 2

2	2	4.6	41.544	2.2556	Q9NZJ7;Q9NZJ7-2;Q9NZJ7-3	Mitochondrial carrier homolog 1
1	1	12	18.733	2.2524	P61966	AP-1 complex subunit sigma-1A
1	1	7	26.51	2.2489	Q9H6S1-3;Q9H6S1-5;Q9H6S1-4;Q9H6S1	5-azacytidine-induced protein 2
2	2	2.8	145.94	2.2489	Q12913;Q12913-2	Receptor-type tyrosine-protein phosphatase eta
1	1	1.9	58.452	2.2477	P48029-4;P48029	Sodium- and chloride-dependent creatine transporter 1
1	1	3.6	52.485	2.2456	Q14202-3;Q14202-2;Q14202	Zinc finger MYM-type protein 3
5	5	14.7	51.657	2.2453	P04424;P04424-2;P04424-3	Argininosuccinate lyase
1	1	0	35.619	2.245	REV__Q9H9B4	
1	1	0.4	344.15	2.2386	O60673-2;O60673	DNA polymerase zeta catalytic subunit
2	2	0	43.06	2.2385	REV__P37268-5;REV__P37268;REV__P37268-4;REV__P37268-3;REV__P37268-2	
3	3	2.3	209.48	2.2355	P49750-3;P49750-1;P49750	YLP motif-containing protein 1
1	1	12.4	27.084	2.2325	Q8IYP2	Serine protease 58
5	1	41.4	14.728	2.2323	P62987;P0CG47;P0CG48	Ubiquitin-60S ribosomal protein L40;Ubiquitin;60S ribosomal protein L40;Polyubiquitin-B;Ubiquitin;Polyubiquitin-C;Ubiquitin
3	1	17	29.173	2.2321	Q8TDQ7-3;Q8TDQ7;Q8TDQ7-4;Q8TDQ7-5;Q8TDQ7-2	Glucosamine-6-phosphate isomerase 2
4	1	10.3	45.517	2.2311	A6NHL2-2;A6NHL2	Tubulin alpha chain-like 3
3	3	8.1	55.882	2.2301	Q9UJX2-3;Q9UJX2	Cell division cycle protein 23 homolog
1	1	5.9	18.894	2.2253	Q07817-2;Q07817-3;Q07817	Bcl-2-like protein 1
2	2	7.4	45.285	2.2251	O75600;O75600-2	2-amino-3-ketobutyrate coenzyme A ligase, mitochondrial
1	1	8.2	21.225	2.2208	P02794	Ferritin heavy chain;Ferritin heavy chain, N-terminally processed
2	2	38.2	7.5217	2.2204	O60262	Guanine nucleotide-binding protein G(I)/G(S)/G(O) subunit gamma-7
1	1	2	62.165	2.2203	Q12899	Tripartite motif-containing protein 26
3	3	7.6	54.471	2.2178	Q658P3-3;Q658P3;Q658P3-2;Q658P3-4	Metalloreductase STEAP3
2	2	23.5	13.37	2.2175	Q16864;Q16864-2	V-type proton ATPase subunit F
2	2	32.1	6.6767	2.2142	P62273;P62273-2	40S ribosomal protein S29
4	1	10.6	60.646	2.2086	Q13153;Q13153-2	Serine/threonine-protein kinase PAK 1
2	2	2.6	105.13	2.2063	O75110-2;O75110	Probable phospholipid-transporting ATPase IIA
1	1	14.9	9.6008	2.2053	O14519-2;O14519	Cyclin-dependent kinase 2-associated protein 1
3	3	9.7	40.526	2.197	Q9BRQ8;Q9BRQ8-2	Apoptosis-inducing factor 2
3	3	5.8	59.659	2.1956	Q9H0X4;Q9H0X4-2	Protein ITFG3
3	3	1.9	236.83	2.1954	P42356	Phosphatidylinositol 4-kinase alpha
2	2	31.8	5.0526	2.1943	P62328	Thymosin beta-4;Hematopoietic system regulatory peptide
2	2	10.5	33.232	2.1935	P54920	Alpha-soluble NSF attachment protein
1	1	4	33.702	2.1892	Q13952-6;Q13952-7;Q13952-2;Q13952-5;Q13952-3;Q13952	Nuclear transcription factor Y subunit gamma
2	2	3.2	88.197	2.1869	Q01433-3;Q01433-5;Q01433-2;Q01433-4;Q01433	AMP deaminase 2
1	1	5.4	23.973	2.1837	O14569	Cytochrome b561 domain-containing protein 2
1	1	3.2	54.724	2.1804	Q9UJ83-3;Q9UJ83-4;Q9UJ83-2;Q9UJ83	2-hydroxyacyl-CoA lyase 1
2	2	2.2	160.99	2.1779	Q96P48-3;Q96P48;Q96P48-7;Q96P48-2;Q96P48-4;Q96P48-1	Arf-GAP with Rho-GAP domain, ANK repeat and PH domain-containing protein 1
2	2	18.7	14.234	2.1757	E9PRG8	Uncharacterized protein C11orf98
1	1	15.3	12.899	2.1689	Q9HD47-4;Q9HD47-2;Q9HD47-3;Q9HD47	Ran guanine nucleotide release factor
1	1	12.8	13.157	2.1629	O95164	Ubiquitin-like protein 3
2	2	4.3	72.759	2.1611	Q9BRK4	Leucine zipper putative tumor suppressor 2
2	2	2.7	109.22	2.1595	Q9UPR0-2;Q9UPR0-3;Q9UPR0	Inactive phospholipase C-like protein 2
2	2	2.4	105.63	2.1519	Q9BSJ2-4;Q9BSJ2;Q9BSJ2-3	Gamma-tubulin complex component 2
1	1	3.7	53.076	2.151	Q92993-2;Q92993-4;Q92993;Q92993-3	Histone acetyltransferase KAT5
1	1	7.6	17.083	2.1502	Q6NS38-2;Q6NS38	Alpha-ketoglutarate-dependent dioxygenase alkB homolog 2

1	1	20.9	7.7019	2.1479	Q8N5G0;Q8N5G0-2	Small integral membrane protein 20
1	1	4.5	41.924	2.1453	P42765	3-ketoacyl-CoA thiolase, mitochondrial
2	2	12.1	22.531	2.1436	Q9NQ34;Q9NQ34-2	Transmembrane protein 9B
2	2	3.7	70.111	2.1425	Q9Y2P4	Long-chain fatty acid transport protein 6
2	2	12.5	23.283	2.1422	Q08623-3;Q08623;Q08623-4;Q08623-2	Pseudouridine-5-phosphatase
2	2	6.9	35.375	2.1388	Q9BSK2;Q96CQ1-2;Q96CQ1-3;Q96CQ1	Solute carrier family 25 member 33;Solute carrier family 25 member 36
1	1	1.7	97.181	2.138	Q99549;Q99549-2	M-phase phosphoprotein 8
2	2	10.8	22.724	2.1365	O14653-3;O14653;O14653-2	Golgi SNAP receptor complex member 2
3	2	5.4	72.397	2.1339	Q96AC1-2;Q96AC1;Q96AC1-3	Fermitin family homolog 2
3	3	4.7	103.71	2.131	Q6P3W7	SCY1-like protein 2
1	1	2.6	65.424	2.1265	Q9HBM0-2;Q9HBM0	Vezatin
2	2	5.5	74.508	2.124	Q9BSC4-2;Q9BSC4-4;Q9BSC4	Nucleolar protein 10
1	1	2.6	64.467	2.1203	Q5VTL8	Pre-mRNA-splicing factor 38B
2	2	10.9	23.457	2.116	P46736-4;P46736-2;P46736-3;P46736;P46736-5	Lys-63-specific deubiquitinase BRCC36
2	2	8	26.537	2.1117	P55061;P55061-2	Bax inhibitor 1
2	2	1.8	141.46	2.1111	Q9H9B1;Q9H9B1-4;Q96KQ7-2;Q9H9B1-3;Q96KQ7	Histone-lysine N-methyltransferase EHMT1;Histone-lysine N-methyltransferase EHMT2
1	1	14	12.114	2.1077	Q53S33	BolA-like protein 3
1	1	4	37.137	2.107	Q96L58	Beta-1,3-galactosyltransferase 6
2	2	5.4	51.78	2.1025	Q6PCB7-2;Q6PCB7	Long-chain fatty acid transport protein 1
2	2	6.4	33.356	2.1022	A1L3X0	Elongation of very long chain fatty acids protein 7
2	2	6	45.501	2.0987	O43581;O43581-6;O43581-2;O43581-5;O43581-4;O43581-3	Synaptotagmin-7
1	1	3.8	55.399	2.0916	O60779	Thiamine transporter 1
2	2	7.4	38.628	2.091	Q9Y5Y5;Q9Y5Y5-2	Peroxisomal membrane protein PEX16
2	2	6.2	61.731	2.0885	Q86TM6-2;Q86TM6-3;Q86TM6	E3 ubiquitin-protein ligase synoviolin
2	2	3.8	69.153	2.0858	Q08426-2;Q08426	Peroxisomal bifunctional enzyme;Enoyl-CoA hydratase/3,2-trans-enoyl-CoA isomerase;3-hydroxyacyl-CoA dehydrogenase
1	1	3.9	46.575	2.0857	Q13309-2;Q13309	S-phase kinase-associated protein 2
3	3	12.1	29.842	2.0839	P82914	28S ribosomal protein S15, mitochondrial
1	1	4	51.033	2.0835	P08603-2;P08603	Complement factor H
3	2	8.2	33.304	2.0814	Q00535;Q00535-2	Cyclin-dependent-like kinase 5
2	2	2	153.1	2.0809	Q99570	Phosphoinositide 3-kinase regulatory subunit 4
2	2	22	15.789	2.0802	Q9Y244	Proteasome maturation protein
1	1	2.8	43.091	2.0785	Q9NYF3	Protein FAM53C
2	2	14.8	23.423	2.0765	Q8TEA8	D-tyrosyl-tRNA(Tyr) deacylase 1
1	1	9.6	15.688	2.0763	Q9BTT4	Mediator of RNA polymerase II transcription subunit 10
3	3	7.4	44.98	2.0746	Q96IG2-2;Q96IG2	F-box/LRR-repeat protein 20
2	2	5.3	54.567	2.0729	Q9H9S5	Fukutin-related protein
2	2	1.4	192.23	2.0686	O60229-2;O60229	Kalirin
2	2	9.4	35.509	2.0629	O75208;O75208-2	Ubiquinone biosynthesis protein COQ9, mitochondrial
2	2	6	56.213	2.0598	Q9BZI7-2;Q9BZI7	Regulator of nonsense transcripts 3B
2	2	5.3	36.804	2.0592	Q8WVY7	Ubiquitin-like domain-containing CTD phosphatase 1
1	1	17.2	10.086	2.057	O14933-2;O14933	Ubiquitin/ISG15-conjugating enzyme E2 L6
3	3	5.5	66.615	2.0562	Q8NAT1	Protein O-linked-mannose beta-1,4-N-acetylglucosaminyltransferase 2
1	1	1.1	128.56	2.0551	Q9UJF2;Q9UJF2-2	Ras GTPase-activating protein nGAP
2	2	9.2	29.162	2.052	P24468-3;P24468-2;P24468;P10589	COUP transcription factor 2;COUP transcription factor 1
2	2	4.7	46.587	2.0518	Q9BRR3	Transmembrane protein 246
1	1	0	42.298	2.0507	REV__Q2TAA8-2;REV__Q2TAA8	
1	1	3.5	31.381	2.0492	Q96FX7	tRNA (adenine(58)-N(1))-methyltransferase catalytic subunit TRMT61A
1	1	3.6	34.632	2.0488	P09486	SPARC

2	2	6	57.214	2.0484	P51649;P51649-2	Succinate-semialdehyde dehydrogenase, mitochondrial
4	1	12.5	40.149	2.0479	Q9BQ04	RNA-binding protein 4B
1	1	6.8	24.536	2.0479	sp|Q1RMN8|;CON__Q1RMN8	
2	2	9.4	25.261	2.0459	Q9BVK8;Q9BVK8-2	Transmembrane protein 147
1	1	17.6	7.7412	2.0434	P60059	Protein transport protein Sec61 subunit gamma
2	2	12.9	26.152	2.0414	Q9HBM1	Kinetochore protein Spc25
2	2	3.8	88.519	2.0359	Q9ULV3-5;Q9ULV3-3;Q9ULV3-4;Q9ULV3-2;Q9ULV3	Cip1-interacting zinc finger protein
1	1	1.8	81.688	2.0353	Q9BYX2-4;Q9BYX2-3;Q9BYX2-2;Q9BYX2	TBC1 domain family member 2A
2	2	3.8	81.544	2.0344	O00459	Phosphatidylinositol 3-kinase regulatory subunit beta
2	2	10.4	44.123	2.0246	Q9UBU6	Protein FAM8A1
1	1	8.1	17.522	2.0233	Q96B96;Q96B96-2	Promethin
2	2	2.9	98.099	2.021	O95235-2;O95235	Kinesin-like protein KIF20A
1	1	2.8	56.684	2.0096	P98170	E3 ubiquitin-protein ligase XIAP
2	2	12.7	17.936	2.0085	P56557	Transmembrane protein 50B
1	1	3.2	43.885	2.007	O15533-2;O15533;O15533-3	Tapasin
1	1	2.6	68.264	2.0066	P55199	RNA polymerase II elongation factor ELL
2	2	9.6	37.866	2.0047	O15121	Sphingolipid delta(4)-desaturase DES1
2	2	3.7	69.5	2.0034	O95340;O95340-2	Bifunctional 3-phosphoadenosine 5-phosphosulfate synthase 2;Sulfate adenylyltransferase;Adenylyl-sulfate kinase
1	1	3	44.126	2.001	P55085	Proteinase-activated receptor 2;Proteinase-activated receptor 2, alternate cleaved 1;Proteinase-activated receptor 2, alternate cleaved 2
2	2	9.5	36.312	2	O75688-5;O75688-4;O75688-2;O75688	Protein phosphatase 1B
1	1	5.5	35.867	1.9999	Q9UET6-2;Q9UET6	Putative tRNA (cytidine(32)/guanosine(34)-2-O)-methyltransferase
1	1	2.7	46.359	1.9993	Q8IX90	Spindle and kinetochore-associated protein 3
1	1	2.3	64.804	1.9991	Q9UGN5-2;Q9UGN5	Poly [ADP-ribose] polymerase 2
1	1	6.3	20.894	1.9978	Q969E8	Pre-rRNA-processing protein TSR2 homolog
1	1	11.8	10.834	1.9958	P05109	Protein S100-A8;Protein S100-A8, N-terminally processed
1	1	1.2	92.967	1.9954	Q13488	V-type proton ATPase 116 kDa subunit a isoform 3
2	2	4.2	80.76	1.9948	O43815-2;O43815	Striatin
2	2	6	45.016	1.9916	Q8WV24	Pleckstrin homology-like domain family A member 1
4	2	11.7	34.803	1.991	A6NHQ2	rRNA/tRNA 2-O-methyltransferase fibrillarin-like protein 1
2	2	10.5	35.505	1.9908	Q9BQ69	O-acetyl-ADP-ribose deacetylase MACROD1
2	1	6	39.154	1.9905	Q4G0F5	Vacuolar protein sorting-associated protein 26B
3	1	15.3	34.196	1.9897	P0DMN0;P0DMM9;P0DMM9-3;P0DMM9-2	Sulfotransferase 1A4;Sulfotransferase 1A3
2	2	9	35.957	1.9885	P35813-2;P35813;P35813-3	Protein phosphatase 1A
1	1	3.4	46.121	1.9854	A6NJ78	Probable methyltransferase-like protein 15
1	1	4.2	43.448	1.9853	Q8N8R5	UPF0565 protein C2orf69
2	2	10.4	34.412	1.9835	Q9HA64	Ketosamine-3-kinase
2	2	1.2	251.74	1.9764	Q6Q759	Sperm-associated antigen 17
2	2	7.4	38.418	1.9758	Q8N335	Glycerol-3-phosphate dehydrogenase 1-like protein
1	1	10.1	13.369	1.9704	B2RBV5	
3	3	11.3	34.114	1.9655	O95159	Zinc finger protein-like 1
3	3	24.6	21.764	1.9653	Q9H0A8	COMM domain-containing protein 4
2	2	3.7	72.413	1.9637	Q14244-3;Q14244-2;Q14244;Q14244-5;Q14244-6;Q14244-4;Q14244-7	Ensconsin
1	1	1.7	107.52	1.9607	O76039-2;O76039	Cyclin-dependent kinase-like 5
1	1	14.1	9.467	1.96	Q9NQG1	Protein MANBAL
1	1	3.5	37.84	1.96	A8MUA0	Putative UPF0607 protein ENSP00000381514
1	1	1.9	73.421	1.96	Q8N3R9-2;Q8N3R9	MAGUK p55 subfamily member 5
3	3	1.2	355.88	1.9567	O95613-2;O95613	Pericentrin
2	2	8	39.271	1.9566	Q96A46	Mitoferrin-2

1	1	4.8	35.634	1.9547	Q9H0V1-2;Q9H0V1	Transmembrane protein 168
5	0	44.9	16.495	1.9546	Q13404;Q13404-7;Q13404-2;Q13404-1	Ubiquitin-conjugating enzyme E2 variant 1
2	2	7	48.049	1.9525	Q9ULD2-7;Q9ULD2-6;Q9ULD2-2;Q9ULD2;Q9ULD2-4;Q9ULD2-3	Microtubule-associated tumor suppressor 1
3	3	16.4	24.711	1.9514	Q9H0V9-3;Q9H0V9;Q9H0V9-2	VIP36-like protein
2	2	10.9	38.725	1.9504	Q01664	Transcription factor AP-4
1	1	5.9	30.491	1.9488	Q9NXK8-2;Q9NXK8	F-box/LRR-repeat protein 12
1	1	2.8	68.546	1.9474	Q8NHQ9	ATP-dependent RNA helicase DDX55
1	1	4.3	23.85	1.9445	Q9NZD2	Glycolipid transfer protein
3	3	12.8	55.492	1.9443	Q8TF71	Monocarboxylate transporter 10
1	1	7.8	16.884	1.9441	Q8NCS4	Uncharacterized protein ZMYM6NB
3	3	10.3	33.05	1.9437	Q9NR56-3;Q9NR56-4;Q9NR56-7;Q9NR56-6;Q9NR56-2;Q9NR56-5;Q9NR56;Q5VZF2-3;Q5VZF2-2;Q5VZF2	Muscleblind-like protein 1;Muscleblind-like protein 2
1	1	5.4	32.952	1.9425	P49069	Calcium signal-modulating cyclophilin ligand
2	2	6.7	57.1	1.9411	P43005	Excitatory amino acid transporter 3
1	1	1.5	77.499	1.9404	Q13671-2;Q13671	Ras and Rab interactor 1
3	3	6.8	60.335	1.9395	Q96AA3	Protein RFT1 homolog
1	1	8	15.645	1.9377	Q9HAB8-2;Q9HAB8	Phosphopantothenate--cysteine ligase
1	1	6.7	26.377	1.9341	Q86YH6-2;Q86YH6	Decaprenyl-diphosphate synthase subunit 2
3	3	11.4	33.116	1.9337	Q9BQT8-2;Q9BQT8	Mitochondrial 2-oxodicarboxylate carrier
2	2	22.3	13.238	1.9336	Q96D05;Q96D05-2	Uncharacterized protein C10orf35
2	2	4.9	49.764	1.9314	Q6N075;Q6N075-2	Molybdate-anion transporter
1	1	1.1	141.65	1.9307	O95382-3;O95382	Mitogen-activated protein kinase kinase kinase 6
1	1	13.6	12.395	1.9294	Q9BSY4;Q9BSY4-2	Coiled-coil-helix-coiled-coil-helix domain-containing protein 5
2	2	17.6	18.419	1.924	Q9BQ61	Uncharacterized protein C19orf43
2	2	5.5	74.712	1.9195	Q9UPT5-2;Q9UPT5-5;Q9UPT5-1;Q9UPT5-6;Q9UPT5;Q9UPT5-4	Exocyst complex component 7
3	3	6.5	72.593	1.9163	Q96G46;Q96G46-2	tRNA-dihydrouridine(47) synthase [NAD(P)(+)]-like
2	2	21.3	10.378	1.9157	Q9Y5J7	Mitochondrial import inner membrane translocase subunit Tim9
1	1	4.8	23.607	1.9132	Q9H201-2;Q9H201	Epsin-3
1	1	3.3	62.577	1.9114	Q6GPH6-3;Q6GPH6;Q6GPH6-2	Inositol 1,4,5-trisphosphate receptor-interacting protein-like 1
1	1	5.1	32.011	1.9093	O95070	Protein YIF1A
2	2	3	111.76	1.9074	Q68CR1-3;Q68CR1-2;Q68CR1	Protein sel-1 homolog 3
1	1	1	122.84	1.9049	P82094;P82094-2	TATA element modulatory factor
2	2	6.5	47.558	1.9041	Q7Z698;Q7Z698-2	Sprouty-related, EVH1 domain-containing protein 2
1	1	0	96.513	1.9	REV__Q9Y2J2-2;REV__Q9Y2J2-4;REV__Q9Y2J2	
1	1	2.7	54.097	1.8988	Q8IZ73-2;Q8IZ73	RNA pseudouridylate synthase domain-containing protein 2
2	2	3	115.08	1.8971	P40818-2;P40818	Ubiquitin carboxyl-terminal hydrolase 8
1	1	26.3	6.9371	1.8949	Q96Q11-3;Q96Q11-2;Q96Q11	CCA tRNA nucleotidyltransferase 1, mitochondrial
2	2	23.8	17.84	1.8901	Q8TDP1	Ribonuclease H2 subunit C
5	1	15.2	49.765	1.889	Q8IUI8	Cytokine receptor-like factor 3
2	2	10	29.633	1.885	Q30201-2;Q30201-8;Q30201-5;Q30201;Q30201-4;Q30201-3	Hereditary hemochromatosis protein
11	1	9.9	192.79	1.8845	sp|P01030|;CON__P01030	
3	3	15.1	33.845	1.8787	O43709-3;O43709;O43709-2	Probable 18S rRNA (guanine-N(7))-methyltransferase
8	1	30.6	21.865	1.8748	P10412	Histone H1.4
3	3	2.2	238.84	1.8732	Q9BZ29-4;Q9BZ29-3;Q9BZ29-5;Q9BZ29	Dedicator of cytokinesis protein 9
9	1	19.6	73.46	1.8689	O94925	Glutaminase kidney isoform, mitochondrial
1	1	2.3	53.261	1.8689	Q9NUQ7	Ufm1-specific protease 2
1	1	3.3	65.111	1.8684	Q5VV42	Threonylcarbamoyladenosine tRNA methylthiotransferase

1	1	5.3	27.55	1.8676	Q5SWH9	Transmembrane protein 69
3	3	7.7	51.49	1.8661	Q9UHJ6	Sedoheptulokinase
2	2	1	340.67	1.8623	Q96Q15-3;Q96Q15-2;Q96Q15;Q96Q15-4	Serine/threonine-protein kinase SMG1
1	1	13.5	18.62	1.8579	Q9NWV8-3;Q9NWV8	BRISC and BRCA1-A complex member 1
1	1	2.7	57.799	1.8567	Q9Y2H2-4;Q9Y2H2	Phosphatidylinositide phosphatase SAC2
2	1	8.1	43.519	1.8563	Q92599-3;Q92599-2;Q92599-4;Q92599	Septin-8
3	3	17.6	24.121	1.8561	Q9GZP4-2;Q9GZP4	PITH domain-containing protein 1
8	1	15	68.964	1.8523	sp|Q2KJ62|;CON__Q2KJ62	
1	1	0.7	216.5	1.8482	Q9H1A4	Anaphase-promoting complex subunit 1
1	1	1.6	96.904	1.8451	Q06418	Tyrosine-protein kinase receptor TYRO3
1	1	0.6	160.25	1.8451	Q9H6S0	Probable ATP-dependent RNA helicase YTHDC2
1	1	8.8	23.782	1.8445	Q8ND25;Q8ND25-2	E3 ubiquitin-protein ligase ZNRF1
3	3	10.1	37.14	1.8385	Q5HYK3	2-methoxy-6-polyprenyl-1,4-benzoquinol methylase, mitochondrial
2	2	2.7	98.655	1.8384	P42566;P42566-2	Epidermal growth factor receptor substrate 15
2	2	5.2	61.661	1.8376	Q6ZWJ1	Syntaxin-binding protein 4
1	1	0	52.488	1.8371	REV__P10619-2;REV__P10619	
3	3	2.5	172.46	1.8321	Q6DT37	Serine/threonine-protein kinase MRCK gamma
176	1	46.4	518.03	1.8312	Q15149-3	Plectin
2	2	3.9	49.721	1.8304	sp|FA9515222|;P02679-2;P02679	Fibrinogen gamma chain
2	2	5.6	65.582	1.8271	Q9BRS2	Serine/threonine-protein kinase RIO1
3	3	10.6	33.814	1.8269	O75880	Protein SCO1 homolog, mitochondrial
2	2	10.3	32.679	1.8258	O95407	Tumor necrosis factor receptor superfamily member 6B
3	3	9.8	34.624	1.8223	O94766-2;O94766	Galactosylgalactosylxylosylprotein 3-beta-glucuronosyltransferase 3
1	1	6.1	17.082	1.8212	Q9H446-2;Q9H446	RWD domain-containing protein 1
1	1	3.6	60.031	1.816	Q9H267-2;Q9H267	Vacuolar protein sorting-associated protein 33B
2	2	18.1	12.474	1.815	Q99643-5;Q99643-3;Q99643	Succinate dehydrogenase cytochrome b560 subunit, mitochondrial
1	1	3	37.718	1.8111	Q9NZC3	Glycerophosphodiester phosphodiesterase 1
2	2	6.9	42.548	1.8056	Q96RQ1	Endoplasmic reticulum-Golgi intermediate compartment protein 2
2	2	6.9	38.484	1.8039	B7ZAQ6-2;B7ZAQ6-3;P0CG08;B7ZAQ6	Golgi pH regulator A;Golgi pH regulator B
2	2	8.6	30.549	1.8036	Q8IY95-2;Q8IY95	Transmembrane protein 192
2	2	2.5	124.26	1.8023	Q7Z6B7;Q7Z6B7-2;O75044	SLIT-ROBO Rho GTPase-activating protein 1;SLIT-ROBO Rho GTPase-activating protein 2
2	2	6.3	48.195	1.8022	Q9UK39	Nocturnin
2	2	28.8	11.986	1.802	Q15004;Q15004-2	PCNA-associated factor
2	2	2.4	87.378	1.8007	Q7Z3C6-2;Q7Z3C6	Autophagy-related protein 9A
1	1	7.7	20.031	1.797	Q9Y584	Mitochondrial import inner membrane translocase subunit Tim22
1	1	7.3	17.486	1.7947	P24390-2;P33947-2;P33947;P24390	ER lumen protein-retaining receptor 1;ER lumen protein-retaining receptor 2
1	1	3.4	49.724	1.7929	Q9H8M7	Protein FAM188A
2	2	10.3	13.426	1.7888	Q92844-2;Q92844-3;Q92844	TRAF family member-associated NF-kappa-B activator
3	3	19.6	17.811	1.7873	Q9GZT6-3;Q9GZT6-2;Q9GZT6	Coiled-coil domain-containing protein 90B, mitochondrial
1	1	22.2	16.872	1.787	Q16611-2;Q16611	Bcl-2 homologous antagonist/killer
2	2	10.9	41.007	1.7861	P78381-2;P78381;P78381-3;P78381-5;P78381-4	UDP-galactose translocator
1	1	2	66.86	1.7829	O00391-2;O00391	Sulfhydryl oxidase 1
2	2	8.6	39.828	1.7819	Q96GZ6-8;Q96GZ6-6;Q96GZ6-7;Q96GZ6-5;Q96GZ6-9;Q96GZ6-2;Q96GZ6	Solute carrier family 41 member 3
2	2	3.2	100.4	1.7819	P20936-2;P20936-4;P20936;P20936-3	Ras GTPase-activating protein 1
1	1	2	59.095	1.7792	P35240-4;P35240-6;P35240-5;P35240-8;P35240-3;P35240;P35240-2	Merlin
2	2	23.9	11.75	1.7778	Q8NFU3-3;Q8NFU3;Q8NFU3-2;Q8NFU3-4	Thiosulfate sulfurtransferase/rhodanese-like domain-containing protein 1
3	3	1.8	274.38	1.7753	Q9BV73-2;Q9BV73	Centrosome-associated protein CEP250
2	2	4.6	55.807	1.7732	Q969P0-3;Q969P0	Immunoglobulin superfamily member 8
1	1	18.5	6.3133	1.771	A0A0B4J2F0	

2	2	9.5	49.967	1.7691	Q49AR2;Q49AR2-2	UPF0489 protein C5orf22
1	1	9.7	10.648	1.7654	Q6NW29-2;Q6NW29	RWD domain-containing protein 4
2	2	3.7	78.233	1.7654	Q9Y2D4-2;Q9Y2D4	Exocyst complex component 6B
2	2	16.9	17.779	1.7644	O95562	Vesicle transport protein SFT2B
2	2	2.9	81.165	1.7618	Q96SU4-7;Q96SU4-2;Q96SU4;Q96SU4-6;Q96SU4-5;Q96SU4-4;Q96SU4-3	Oxysterol-binding protein-related protein 9
2	2	2.4	129.39	1.7584	P46020-3;P46020-2;P46020	Phosphorylase b kinase regulatory subunit alpha, skeletal muscle isoform
2	2	14.5	17.114	1.7568	Q9UI09;Q9UI09-2	NADH dehydrogenase [ubiquinone] 1 alpha subcomplex subunit 12
1	1	1.5	75.806	1.7539	P51511	Matrix metalloproteinase-15
1	1	1	121.72	1.7526	Q9NNW5	WD repeat-containing protein 6
3	3	6.4	64.071	1.7482	O96013;O96013-2;O96013-3;O96013-4	Serine/threonine-protein kinase PAK 4
1	1	2.1	71.158	1.7463	Q8NCL4	Polypeptide N-acetylgalactosaminyltransferase 6
6	1	46	21.656	1.7448	P01116	GTPase KRas;GTPase KRas, N-terminally processed
1	1	11.7	13.188	1.7434	Q96A57;Q96A57-2	Transmembrane protein 230
1	1	2.6	78.579	1.7419	Q9NR19;Q9NR19-2	Acetyl-coenzyme A synthetase, cytoplasmic
1	1	7.2	15.819	1.7414	Q6P161	39S ribosomal protein L54, mitochondrial
2	2	8.1	23.181	1.7337	P09497-2;P09497	Clathrin light chain B
2	2	0	137.17	1.7317	REV__Q9NS87-4;REV__Q9NS87-2;REV__Q9NS87	
1	1	1.4	133.67	1.7278	Q86SQ4-2;Q86SQ4;Q86SQ4-4;Q86SQ4-3	G-protein coupled receptor 126
2	2	14	25.673	1.7262	Q9HD15	Steroid receptor RNA activator 1
1	1	2.5	44.098	1.7252	Q9GZX3	Carbohydrate sulfotransferase 6
2	2	8.4	37.643	1.7235	P51946	Cyclin-H
1	1	7	27.334	1.7227	P45984-5;P45984-2;P45984	Mitogen-activated protein kinase 9
3	3	9.7	47.574	1.719	P29590-14;P29590-10;P29590-4;P29590-12;P29590-2;P29590-5;P29590-9;P29590-13;P29590-3;P29590-8;P29590-11;P29590	Protein PML
1	1	2.4	55.799	1.7182	P42785;P42785-2	Lysosomal Pro-X carboxypeptidase
2	2	0.8	259.84	1.7119	P46100-2;P46100-5;P46100-3;P46100-4;P46100	Transcriptional regulator ATRX
1	1	3.3	33.886	1.7083	Q9BY42	Protein RTF2 homolog
3	3	4	96.257	1.7079	P19021-2;P19021-4;P19021-3;P19021-6;P19021;P19021-5	Peptidyl-glycine alpha-amidating monooxygenase;Peptidylglycine alpha-hydroxylating monooxygenase;Peptidyl-alpha-hydroxyglycine alpha-amidating lyase
3	3	5.1	83.642	1.7071	Q9BXB4	Oxysterol-binding protein-related protein 11
1	1	1.9	60.37	1.7063	Q13042-4;Q13042-3;Q13042-2;Q13042	Cell division cycle protein 16 homolog
3	3	14.4	34.645	1.7054	P13051;P13051-2	Uracil-DNA glycosylase
2	2	9.5	42.129	1.7052	Q9BTY7	Protein HGH1 homolog
1	1	4	48.886	1.7029	Q9NPH0	Lysophosphatidic acid phosphatase type 6
1	1	12.7	13.952	1.7018	O60927	Protein phosphatase 1 regulatory subunit 11
2	1	4	57.249	1.698	sp|Q3SY84|;CON__Q3SY84;Q3SY84	Keratin, type II cytoskeletal 71
2	2	7.3	36.271	1.6975	Q9NRZ7-2;Q9NRZ7;Q9NRZ7-3	1-acyl-sn-glycerol-3-phosphate acyltransferase gamma
1	1	2.4	65.581	1.6933	Q6PCB5-2;Q6PCB5	Round spermatid basic protein 1-like protein
1	1	3.8	38.864	1.6904	Q15119-2;Q15119	[Pyruvate dehydrogenase (acetyl-transferring)] kinase isozyme 2, mitochondrial
1	1	12.5	15.128	1.6879	Q7Z7F7;Q7Z7F7-2	39S ribosomal protein L55, mitochondrial
1	1	3.6	47.819	1.6848	Q14642	Type I inositol 1,4,5-trisphosphate 5-phosphatase
1	1	2.6	63.574	1.6831	Q9BWE0;Q9BWE0-4	Replication initiator 1
1	1	12.4	11.188	1.6801	A1XBS5-5;A1XBS5-2;A1XBS5-4;A1XBS5-3;A1XBS5	Protein FAM92A1
2	2	3.1	81.086	1.6788	Q6NSJ0	Uncharacterized family 31 glucosidase KIAA1161
1	1	7	22.745	1.6778	P78560	Death domain-containing protein CRADD
2	2	4.5	61.956	1.6744	Q9BW71;Q9BW71-2;Q9BW71-3	HIRA-interacting protein 3

3	3	2.4	185.85	1.6718	Q7Z3U7-2;Q7Z3U7-6;Q7Z3U7-5;Q7Z3U7;Q7Z3U7-3	Protein MON2 homolog
2	2	2.4	134.88	1.6715	Q3B7T1-5;Q3B7T1;Q3B7T1-4;Q3B7T1-3	Erythroid differentiation-related factor 1
1	1	8.2	18.89	1.6704	Q8IUX1-4;Q8IUX1	Complex I assembly factor TMEM126B, mitochondrial
1	1	5.4	26.289	1.67	Q86W33-2;Q86W33-3;Q86W33	Transmembrane protein adipocyte-associated 1
2	2	3.9	73.158	1.6693	Q96ME1-2;Q96ME1-4;Q96ME1;Q96ME1-3	F-box/LRR-repeat protein 18
1	1	6.4	29.798	1.6663	Q9UH17-2;Q9UH17-3;Q9UH17;Q96AK3	DNA dC->dU-editing enzyme APOBEC-3B;DNA dC->dU-editing enzyme APOBEC-3D
1	1	2.2	54.646	1.6649	Q8NBM8	Prenylcysteine oxidase-like
2	2	4.9	44.678	1.66	O95801	Tetratricopeptide repeat protein 4
1	1	8.8	24.951	1.6597	Q9NRZ5-2;Q9NRZ5	1-acyl-sn-glycerol-3-phosphate acyltransferase delta
1	1	5.4	27.626	1.6591	Q96EK7-3;Q96EK7-2;Q96EK7	Constitutive coactivator of peroxisome proliferator-activated receptor gamma
2	2	10.1	34.475	1.6589	P56937-3;P56937-2;P56937	3-keto-steroid reductase
1	1	5.7	41.452	1.6509	Q86SR1-3;Q86SR1-2;Q49A17-2;Q49A17;Q86SR1	Polypeptide N-acetylgalactosaminyltransferase 10;Polypeptide N-acetylgalactosaminyltransferase-like 6
2	2	0.6	443.21	1.6448	Q86XX4;Q86XX4-2	Extracellular matrix protein FRAS1
7	2	15.1	64.436	1.6442	Q9H0L4	Cleavage stimulation factor subunit 2 tau variant
1	1	1.4	120.01	1.6426	Q9NQW6-2;Q9NQW6	Actin-binding protein anillin
1	1	4	60.069	1.6414	Q96D53	AarF domain-containing protein kinase 4
1	1	1.5	98.918	1.6412	P53814-5;P53814;P53814-6	Smoothelin
2	2	1.5	206.69	1.64	Q8WWQ0;Q9NSI6-3;Q9NSI6-2;Q9NSI6	PH-interacting protein;Bromodomain and WD repeat-containing protein 1
3	1	8.9	55.96	1.6395	Q13555-10;Q13555-3;Q13555-5;Q13555-4;Q13555-7;Q13555-11;Q13555-9;Q13555-2;Q13555-8;Q13555;Q13555-6	Calcium/calmodulin-dependent protein kinase type II subunit gamma
1	1	5.3	17.158	1.6381	Q15041-3;Q15041-2;Q15041	ADP-ribosylation factor-like protein 6-interacting protein 1
2	2	2.9	113.06	1.6368	Q9P206-2	
1	1	1.1	114.99	1.636	Q6PJG2	ELM2 and SANT domain-containing protein 1
20	1	42.9	57.944	1.6357	P08195-2	4F2 cell-surface antigen heavy chain
20	1	47.9	62.656	1.6318	O60506-3;O60506-4;O60506-5	Heterogeneous nuclear ribonucleoprotein Q
2	2	3.9	67.94	1.6314	Q13421-4;Q13421-3;Q13421;Q13421-2	Mesothelin;Megakaryocyte-potentiating factor;Mesothelin, cleaved form
2	2	4	79.917	1.6288	Q8IZ83-3;Q8IZ83	Aldehyde dehydrogenase family 16 member A1
2	2	27	15.614	1.6287	P60604-2;P60604	Ubiquitin-conjugating enzyme E2 G2
2	2	3.2	83.541	1.6256	Q99567	Nuclear pore complex protein Nup88
2	1	7.4	46.302	1.6254	P31323	cAMP-dependent protein kinase type II-beta regulatory subunit
2	2	0.2	1005.2	1.6245	Q8NF91-4;Q8NF91;Q8NF91-10;Q8NF91-8;Q8NF91-2;Q8NF91-7	Nesprin-1
2	2	8.4	24.817	1.6216	P00846	ATP synthase subunit a
3	3	6	85.561	1.621	P42892-3;P42892-2;P42892-4;P42892	Endothelin-converting enzyme 1
1	1	5.6	30.818	1.6202	O75712	Gap junction beta-3 protein
1	1	5	45.496	1.613	O75056	Syndecan-3
1	1	4	37.031	1.6129	O14662	Syntaxin-16
1	1	3.2	54.178	1.6122	Q53EZ4	Centrosomal protein of 55 kDa
1	1	2	82.221	1.6106	Q04726-2;Q04726-7;Q04726-3;Q04726-6;Q04726-5;Q04726-4;Q04726	Transducin-like enhancer protein 3
1	1	4.1	36.431	1.6105	Q16651	Prostasin;Prostasin light chain;Prostasin heavy chain
1	1	7.8	13.286	1.61	Q14210	Lymphocyte antigen 6D
1	1	2.1	80.674	1.6091	sp|E06074|;CON__ENSEMBL:ENSBTAP00000006074;P49747-2;P49747;P35443	Cartilage oligomeric matrix protein;Thrombospondin-4
1	1	8.6	15.095	1.6077	Q4U2R6	39S ribosomal protein L51, mitochondrial
1	1	3	44.794	1.6074	Q9NZH0;Q9NZH0-2	G-protein coupled receptor family C group 5 member B
2	2	12.3	30.446	1.6057	Q9Y3A2;Q9Y3A2-2	Probable U3 small nucleolar RNA-associated protein 11
1	1	2.9	50.98	1.6032	P49840	Glycogen synthase kinase-3 alpha
1	1	1.3	91.131	1.6025	Q68CJ6	Nuclear GTPase SLIP-GC

1	1	6.2	24.936	1.6016	Q8N2K1-2;Q8N2K1;Q8N2K1-3	Ubiquitin-conjugating enzyme E2 J2
1	1	2.1	63.62	1.6005	Q86W50	Methyltransferase-like protein 16
3	3	5.7	88.06	1.6002	P25440;P25440-2;P25440-4;P25440-3	Bromodomain-containing protein 2
1	1	0.5	271.04	1.5998	P11532-3;P11532-2;P11532-4;P11532-11;P11532	Dystrophin
2	2	2.5	103.64	1.5988	Q07617	Sperm-associated antigen 1
2	2	4.8	77.153	1.5929	Q96RR1;Q96RR1-3;Q96RR1-2	Twinkle protein, mitochondrial
1	1	1.9	75.69	1.5925	Q8NEM2	SHC SH2 domain-binding protein 1
3	3	12.4	43.603	1.5913	Q53HC9	Protein TSSC1
3	3	1	357.52	1.5912	P49454	Centromere protein F
1	1	6.7	24.39	1.5911	Q96BD8-2;Q96BD8	Spindle and kinetochore-associated protein 1
3	3	3.5	117.04	1.59	Q9UP95-5;Q9UP95-2;Q9UP95-6;Q9UP95;Q9UP95-4;Q9UP95-3;Q9UP95-7	Solute carrier family 12 member 4
1	1	4.7	51.581	1.5899	Q9H9C1-2;Q9H9C1	Spermatogenesis-defective protein 39 homolog
1	1	3.8	34.854	1.5847	Q96HP4	Oxidoreductase NAD-binding domain-containing protein 1
1	1	5.7	19.835	1.583	Q8N0U8;Q8N0U8-2	Vitamin K epoxide reductase complex subunit 1-like protein 1
1	1	5.4	19.752	1.583	O75352-2;O75352	Mannose-P-dolichol utilization defect 1 protein
1	1	8.5	12.941	1.5825	Q9UHA4-2;Q9UHA4	Ragulator complex protein LAMTOR3
1	1	11.4	19.55	1.5768	O15182	Centrin-3
1	1	2.3	61.884	1.5756	Q8NHP8-2;Q8NHP8	Putative phospholipase B-like 2;Putative phospholipase B-like 2 32 kDa form;Putative phospholipase B-like 2 45 kDa form
1	1	5.2	37.882	1.5748	Q9H920	RING finger protein 121
1	1	8.6	15.805	1.5723	Q6GMV3	Putative peptidyl-tRNA hydrolase PTRHD1
1	1	4.4	31.71	1.5723	O95873	Uncharacterized protein C6orf47
1	1	6.1	34.304	1.5696	P85298-2;P85298-5;P85298-4;P85298-3;P85298	Rho GTPase-activating protein 8
2	2	5.6	47.44	1.5692	P25116	Proteinase-activated receptor 1
1	1	0	32.477	1.5689	REV__Q9BSH4	
1	1	9.8	14.606	1.5685	Q6P5R6	60S ribosomal protein L22-like 1
1	1	5.9	34.687	1.5669	Q9NVM6	DnaJ homolog subfamily C member 17
1	1	4.7	28.736	1.5642	Q96H20-2;Q96H20	Vacuolar-sorting protein SNF8
2	2	3.5	92.622	1.5641	Q9C0C4	Semaphorin-4C
1	1	10.4	20.574	1.5624	Q9P0T7	Transmembrane protein 9
3	2	15.2	23.712	1.5591	P57729	Ras-related protein Rab-38
1	1	1.2	121.74	1.5575	Q9Y3P9	Rab GTPase-activating protein 1
1	1	14.9	13.857	1.5527	Q9UIL1-3;Q9UIL1-2;Q9UIL1	Short coiled-coil protein
1	1	3.8	26.756	1.5525	Q5SVS4-2;Q5SVS4	Kidney mitochondrial carrier protein 1
2	2	3.7	75.561	1.5374	Q99661-2;Q99661	Kinesin-like protein KIF2C
1	1	6.8	20.776	1.5362	Q99836-3;Q99836-5;Q99836;Q99836-6	Myeloid differentiation primary response protein MyD88
3	3	2	181.79	1.5341	Q9C0C2;Q9C0C2-2	182 kDa tankyrase-1-binding protein
1	1	2.2	72.262	1.5321	O94887-3;O94887-2;O94887	FERM, RhoGEF and pleckstrin domain-containing protein 2
1	1	2.2	69.473	1.5321	Q6UWB1	Interleukin-27 receptor subunit alpha
2	2	1.8	174.91	1.5303	O15438-4;O15438;O15438-2	Canalicular multispecific organic anion transporter 2
2	2	11.6	27.388	1.5271	Q96EY8	Cob(I)yrinic acid a,c-diamide adenosyltransferase, mitochondrial
1	1	10.3	13.467	1.5267	O15392-5;O15392-4;O15392;O15392-2	Baculoviral IAP repeat-containing protein 5
1	1	13.5	10.476	1.5264	Q6P1K1-2;Q6P1K1	Heme transporter HRG1
1	1	11	18.986	1.525	Q3ZCW2	Galectin-related protein
1	1	4.3	37.84	1.5222	O60293-4;O60293-2;O60293	Zinc finger C3H1 domain-containing protein
1	1	4	33.548	1.5202	O75911	Short-chain dehydrogenase/reductase 3
6	2	4.3	182.66	1.5195	Q02880-2;Q02880	DNA topoisomerase 2-beta
1	1	5.4	19.539	1.5186	Q8NB37-3;Q8NB37;Q8NB37-2	Parkinson disease 7 domain-containing protein 1
2	2	0	279.04	1.5158	REV__Q7Z2Y8	

1	1	4.9	29.234	1.5144	Q8NC54	Keratinocyte-associated transmembrane protein 2
1	1	5.8	25.76	1.513	Q68EM7-4;Q68EM7-2;Q68EM7-6;Q68EM7-5;Q68EM7	Rho GTPase-activating protein 17
1	1	12.1	16.533	1.4982	Q9NVX0-2;Q9NVX0-3;Q9NVX0	HAUS augmin-like complex subunit 2
1	1	0	327.82	1.4975	REV__Q8NFP9	
1	1	3.4	46.956	1.4937	P41586-5;P41586-3;P41586;P41586-4;P41586-2	Pituitary adenylate cyclase-activating polypeptide type I receptor
1	1	5.7	54.826	1.4882	O00401	Neural Wiskott-Aldrich syndrome protein
1	1	1.7	66.59	1.4877	Q96GW9	Methionine--tRNA ligase, mitochondrial
1	1	4.1	35.232	1.4875	Q96PU8-5;Q96PU8-9;Q96PU8-8;Q96PU8-6;Q96PU8-3;Q96PU8	Protein quaking
1	1	13.3	8.6312	1.4867	Q9BQ49	Small integral membrane protein 7
2	2	9	21.514	1.4847	Q9NZE8;Q9NZE8-2	39S ribosomal protein L35, mitochondrial
1	1	3.6	35.14	1.4832	Q9NXF8	Palmitoyltransferase ZDHHC7
1	1	6.9	22.09	1.4829	Q96SK2-4;Q96SK2-3;Q96SK2-2;Q96SK2	Transmembrane protein 209
1	1	1.3	105.89	1.4827	O60566-2;O60566;O60566-3	Mitotic checkpoint serine/threonine-protein kinase BUB1 beta
1	1	6.9	33.973	1.4819	Q9UJJ9	N-acetylglucosamine-1-phosphotransferase subunit gamma
1	1	5	22.354	1.4799	Q9NWT8	Aurora kinase A-interacting protein
2	2	6.7	39.416	1.4762	Q9BRP1	Programmed cell death protein 2-like
1	1	3.6	34.842	1.4715	P28328	Peroxisome biogenesis factor 2
1	1	3.2	56.549	1.4693	Q96HW7-2;Q96HW7-3;Q96HW7	Integrator complex subunit 4
1	1	10.7	13.788	1.4692	P28907-2;P28907	ADP-ribosyl cyclase/cyclic ADP-ribose hydrolase 1
1	1	2.9	48.339	1.4679	Q14728	Major facilitator superfamily domain-containing protein 10
2	2	10.8	37.375	1.4663	Q9UJ70;Q9UJ70-2	N-acetyl-D-glucosamine kinase
1	1	0	93.089	1.4636	REV__sp|Q29RQ1|;REV__CON__Q29RQ1	
4	1	39.3	9.4771	1.4627	Q71UM5	40S ribosomal protein S27-like
1	1	4.4	28.957	1.4627	Q9UHJ9-3;Q9UHJ9;Q9UHJ9-5;Q9UHJ9-2	Post-GPI attachment to proteins factor 2
1	1	3.2	37.048	1.4627	Q96B23-2;Q96B23	Uncharacterized protein C18orf25
1	1	4.6	28.669	1.4617	Q9P031	Thyroid transcription factor 1-associated protein 26
1	1	3.4	38.952	1.4607	Q9H1E5	Thioredoxin-related transmembrane protein 4
1	1	0.6	227.84	1.4606	P48634-2;P48634-3;P48634	Protein PRRC2A
1	1	5.9	31.546	1.4591	P80217;P80217-2	Interferon-induced 35 kDa protein
2	2	2.4	120.18	1.4531	Q69YQ0-2;Q69YQ0	Cytospin-A
1	1	1.4	85.379	1.4459	O95785-2;O95785-4;O95785-3;O95785	Protein Wiz
2	2	12.7	25.087	1.4445	Q53TN4-3;Q53TN4	Cytochrome b reductase 1
1	1	4.6	26.754	1.4367	Q8N9N7	Leucine-rich repeat-containing protein 57
1	1	5.1	27.759	1.4348	Q9NW97	Transmembrane protein 51
6	1	46	13.952	1.4334	Q99880;Q99879;Q99877;Q93079;Q5QNW6;P62807;P58876;P57053;O60814;Q5QNW6-2;Q96A08	Histone H2B type 1-L;Histone H2B type 1-M;Histone H2B type 1-N;Histone H2B type 1-H;Histone H2B type 2-F;Histone H2B type 1-C/E/F/G/I;Histone H2B type 1-D;Histone H2B type F-S;Histone H2B type 1-K;Histone H2B type 1-A
1	1	10	25.918	1.4326	P16083	Ribosyldihydronicotinamide dehydrogenase [quinone]
1	1	4	29.105	1.4318	Q96JY6-4;Q96JY6;Q96JY6-3;Q96JY6-5	PDZ and LIM domain protein 2
4	2	16.8	36.979	1.4312	P22694-10;P22694-4;P22694-3;P22694;P22694-5;P22694-7;P22694-6;P22694-9;P22694-2;P22694-8	cAMP-dependent protein kinase catalytic subunit beta
2	2	3.8	56.415	1.4275	Q8N465;Q8N465-2;Q8N465-3	D-2-hydroxyglutarate dehydrogenase, mitochondrial
1	1	5.9	41.496	1.4266	O75503	Ceroid-lipofuscinosis neuronal protein 5
2	2	5.2	80.021	1.4265	Q9UJC3-2;Q9UJC3	Protein Hook homolog 1
2	2	2.3	147.87	1.4226	P16885	1-phosphatidylinositol 4,5-bisphosphate phosphodiesterase gamma-2
1	1	5.2	27.354	1.422	Q9H3H3-1;Q9H3H3;Q9H3H3-3	UPF0696 protein C11orf68
1	1	7.4	23.093	1.4198	P50897-2;P50897	Palmitoyl-protein thioesterase 1
2	1	12.7	17.977	1.4179	P24666-2;P24666-3	Low molecular weight phosphotyrosine protein phosphatase

3	3	1.3	301.79	1.4179	Q02224-3;Q02224	Centromere-associated protein E
12	2	66.2	24.393	1.4162	P62491;P62491-2	Ras-related protein Rab-11A
3	3	0	89.077	1.4152	REV__Q9NXL2	
2	2	6.2	35.759	1.4147	P78383;P78383-2	Solute carrier family 35 member B1
1	1	2.9	52.8	1.4099	P57764	Gasdermin-D
2	2	4.5	49.107	1.4061	Q9BX95	Sphingosine-1-phosphate phosphatase 1
2	2	0	83.021	1.4049	REV__Q96ED9-2;REV__Q96ED9	
1	1	17.7	10.984	1.4031	Q9NPA8-2;Q9NPA8	Transcription and mRNA export factor ENY2
1	1	6.1	20.82	1.4006	Q96HA4-4;Q96HA4-5;Q96HA4	Uncharacterized protein C1orf159
3	1	19.6	21.855	1.3952	Q9UL26	Ras-related protein Rab-22A
1	1	10.9	13.51	1.3869	O75414-2;O75414	Nucleoside diphosphate kinase 6
1	1	1.5	90.043	1.3866	Q63ZY3-3;Q63ZY3;Q63ZY3-2	KN motif and ankyrin repeat domain-containing protein 2
1	1	12.9	11.748	1.3849	Q8WUH6	Transmembrane protein 263
1	1	3	50.244	1.3848	Q15172-2;Q15172	Serine/threonine-protein phosphatase 2A 56 kDa regulatory subunit alpha isoform
2	2	4.8	80.104	1.38	Q5VV41;Q5VV41-2	Rho guanine nucleotide exchange factor 16
2	2	0	110.56	1.3753	REV__O15083	
2	2	5.4	41.084	1.3739	Q9Y2C4;Q9Y2C4-4;Q9Y2C4-3	Nuclease EXOG, mitochondrial
2	2	7.1	53.435	1.3728	Q9NUN5-3;Q9NUN5;Q9NUN5-4;Q9NUN5-2	Probable lysosomal cobalamin transporter
1	1	2.5	73.449	1.3724	O75427	Leucine-rich repeat and calponin homology domain-containing protein 4
1	1	2.4	92.152	1.3717	Q96F46-2;Q96F46	Interleukin-17 receptor A
1	1	7	19.904	1.3714	Q16626	Male-enhanced antigen 1
1	1	2.7	49.955	1.3711	P19971;P19971-2	Thymidine phosphorylase
1	1	10.9	10.165	1.37	Q9BQ48	39S ribosomal protein L34, mitochondrial
1	1	2	93.169	1.3681	Q9Y5B6-2;Q9Y5B6	PAX3- and PAX7-binding protein 1
1	1	3.7	52.44	1.3657	P51608;P51608-2	Methyl-CpG-binding protein 2
2	1	2.9	124.71	1.3633	O60518	Ran-binding protein 6
1	1	1.9	52.787	1.3567	Q5R3I4	Tetratricopeptide repeat protein 38
1	1	1.4	75.993	1.3567	Q8WU17	E3 ubiquitin-protein ligase RNF139
2	2	3.8	88.105	1.3565	Q05209;Q05209-2;Q05209-3	Tyrosine-protein phosphatase non-receptor type 12
1	1	0.5	219.14	1.3565	Q14686	Nuclear receptor coactivator 6
2	2	1.8	208.39	1.3556	Q9UIF9-2;Q9UIF9-3;Q9UIF9	Bromodomain adjacent to zinc finger domain protein 2A
176	1	46.5	516.19	1.3555	Q15149-4	Plectin
2	2	3.4	85.667	1.3543	Q15398-1;Q15398-3;Q15398	Disks large-associated protein 5
1	1	9.8	18.429	1.3484	P54852	Epithelial membrane protein 3
1	1	6.7	25.43	1.3451	Q9BXK1	Krueppel-like factor 16
1	1	2.5	66.306	1.3444	Q14807-2;Q14807	Kinesin-like protein KIF22
2	2	7.3	51.016	1.3433	P23443-4;P23443-3;P23443-5;P23443-2;P23443	Ribosomal protein S6 kinase beta-1
1	1	2.1	71.988	1.3425	Q13049	E3 ubiquitin-protein ligase TRIM32
1	1	11.7	12.388	1.3404	Q9NYM9	BET1-like protein
1	1	7.8	18.824	1.3404	Q8WXD5	Gem-associated protein 6
2	2	4.1	85.756	1.3379	Q96JM7-2;Q96JM7	Lethal(3)malignant brain tumor-like protein 3
2	2	7.3	33.742	1.3341	O75323;O75323-2	Protein NipSnap homolog 2
2	2	4.5	76.18	1.3338	O14523;O14523-2	C2 domain-containing protein 2-like
7	1	70.5	11.418	1.3308	P62937-2	Peptidyl-prolyl cis-trans isomerase A;Peptidyl-prolyl cis-trans isomerase A, N-terminally processed
2	2	15.6	16.476	1.3307	Q86U28	Iron-sulfur cluster assembly 2 homolog, mitochondrial
2	2	2.9	111.17	1.3292	Q96JG6;Q96JG6-2;Q96JG6-3	Coiled-coil domain-containing protein 132
1	1	6.7	23.671	1.3248	Q9UK53-3;Q9UK53-4;Q9UK53-5;Q9UK53-2;Q9UK53	Inhibitor of growth protein 1
3	3	12.9	33.165	1.3246	Q5VZE5-2;Q5VZE5	N-alpha-acetyltransferase 35, NatC auxiliary subunit
1	1	2.7	100.28	1.3245	Q9NV70-2;Q9NV70	Exocyst complex component 1

2	2	17.9	13.293	1.3222	P52435;Q9H1A7;Q9GZM3	DNA-directed RNA polymerase II subunit RPB11-a;DNA-directed RNA polymerase II subunit RPB11-b2;DNA-directed RNA polymerase II subunit RPB11-b1
2	2	0	127.5	1.3152	REV__O75762	
1	1	0	81.199	1.3143	REV__Q969S9-2;REV__Q969S9-3;REV__Q969S9	
1	1	2.4	46.908	1.313	P00749-2;P00749	Urokinase-type plasminogen activator;Urokinase-type plasminogen activator long chain A;Urokinase-type plasminogen activator short chain A;Urokinase-type plasminogen activator chain B
2	2	0	88.183	1.3128	REV__Q92805	
1	1	0	387.32	1.3102	REV__Q7Z407-3;REV__Q7Z407-4;REV__Q7Z407-2;REV__Q7Z407	
2	2	0	41.883	1.3093	REV__Q8IWA4-2;REV__Q8IWA4-3;REV__Q8IWA4	
2	2	8.5	48.955	1.3084	Q15796-2;Q15796	Mothers against decapentaplegic homolog 2
1	1	4.5	43.666	1.3046	Q9GZT9-2;Q9GZT9	Egl nine homolog 1
2	2	4.4	54.722	1.3036	Q12834	Cell division cycle protein 20 homolog
2	2	2.5	104.7	1.3034	Q9NVH2-3;Q9NVH2-2;Q9NVH2;Q9NVH2-4	Integrator complex subunit 7
1	1	1.9	57.296	1.3033	Q8IXS2;Q8IXS2-2	Coiled-coil domain-containing protein 65
1	1	0.6	148.25	1.3002	Q5TZA2-2;Q5TZA2	Rootletin
2	2	1.8	123.92	1.2992	Q15386	Ubiquitin-protein ligase E3C
1	1	8.3	18.246	1.2989	Q9UBK9;Q9UBK9-2	Protein UXT
12	1	8.8	197.48	1.2986	A6NKT7;Q7Z3J3	RanBP2-like and GRIP domain-containing protein 3;RanBP2-like and GRIP domain-containing protein 4
3	3	12.1	33.231	1.2969	Q6PK04	Coiled-coil domain-containing protein 137
1	1	15.4	10.079	1.296	Q96DA6-2;Q96DA6	Mitochondrial import inner membrane translocase subunit TIM14
1	1	1.5	100.89	1.296	O00462	Beta-mannosidase
2	2	6.3	39.87	1.2958	Q6UWU4;Q6UWU4-3;Q6UWU4-2	Bombesin receptor-activated protein C6orf89
2	2	1.7	205.15	1.2934	Q8NDV7-6;Q8NDV7;Q8NDV7-2;Q8NDV7-5	Trinucleotide repeat-containing gene 6A protein
1	1	3.5	41.883	1.2925	Q8IWA4-2;Q8IWA4-3;Q8IWA4	Mitofusin-1
2	2	5.1	47.968	1.292	Q8N370-4;Q8N370-2;Q8N370;Q8N370-3	Large neutral amino acids transporter small subunit 4
2	2	5.7	35.964	1.2888	Q92820	Gamma-glutamyl hydrolase
3	3	0	986.67	1.2883	REV__P20929-3;REV__P20929-2;REV__P20929-4;REV__P20929	
1	1	8.3	21.995	1.2853	Q15126	Phosphomevalonate kinase
1	1	1.9	58.688	1.2847	Q5JPH6;Q5JPH6-2	Probable glutamate--tRNA ligase, mitochondrial
1	1	8.3	23.468	1.2818	Q8WUZ0;Q8WUZ0-2	B-cell CLL/lymphoma 7 protein family member C
2	2	2	135.19	1.2794	O75054;O75054-2	Immunoglobulin superfamily member 3
2	2	0	504.6	1.2778	REV__Q07954	
3	3	28.7	16.676	1.2774	O00762-3;O00762	Ubiquitin-conjugating enzyme E2 C
2	2	1.9	164.9	1.2771	Q8IWU2	Serine/threonine-protein kinase LMTK2
1	1	2.6	61.007	1.277	Q6T423	Solute carrier family 22 member 25
1	1	2.6	38.96	1.276	P03891	NADH-ubiquinone oxidoreductase chain 2
2	2	4.9	44.073	1.2752	A6NKF2	AT-rich interactive domain-containing protein 3C
1	1	0	50.077	1.2752	REV__Q13093	
2	2	10	28	1.2678	Q9BSH5	Haloacid dehalogenase-like hydrolase domain-containing protein 3
2	2	3.2	100.28	1.2672	Q70JA7	Chondroitin sulfate synthase 3
2	2	4.7	68.262	1.2639	P41743	Protein kinase C iota type
1	1	1.5	61.687	1.2639	Q8IU81	Interferon regulatory factor 2-binding protein 1
1	1	2.9	44.392	1.2601	P25929	Neuropeptide Y receptor type 1
2	2	3.9	98.986	1.2594	Q9UL03-3;Q9UL03;Q5JSJ4-4;Q5JSJ4	Integrator complex subunit 6;Protein DDX26B
1	1	2.9	40.05	1.2584	Q9H5K3	Protein O-mannose kinase
1	1	0	123.82	1.2584	REV__Q93100-4;REV__Q93100-2;REV__Q93100-3;REV__Q93100	
2	2	5.4	57.47	1.258	Q96SY0;Q96SY0-4;Q96SY0-3	von Willebrand factor A domain-containing protein 9

1	1	0	25.5	1.255	REV__O15266-2;REV__O15266	
1	1	5.1	22.662	1.2548	Q9H6V9-4;Q9H6V9-3;Q9H6V9;Q9H6V9-2	UPF0554 protein C2orf43
1	1	0	64.069	1.2535	REV__Q86VF2-4;REV__Q86VF2;REV__Q86VF2-5	
1	1	15	14.089	1.2533	Q5BJD5-3;Q5BJD5-2;Q5BJD5	Transmembrane protein 41B
3	3	17.6	24.476	1.2518	Q5VWZ2-2;Q5VWZ2	Lysophospholipase-like protein 1
1	1	14.8	24.594	1.2459	Q9H2V7-5;Q9H2V7-3;Q9H2V7-2;Q9H2V7	Protein spinster homolog 1
1	1	2.4	75.997	1.2449	Q8WXX7-5;Q8WXX7-2;Q8WXX7	Autism susceptibility gene 2 protein
1	1	1.9	97.486	1.2441	O95049-5;O95049;O95049-4;O95049-3	Tight junction protein ZO-3
1	1	4.7	39.327	1.2432	O75818-2;O75818	Ribonuclease P protein subunit p40
1	1	0	85.944	1.2432	REV__O14920-2	
2	2	13.8	18.82	1.2408	Q9UFW8	CGG triplet repeat-binding protein 1
1	1	2.7	54.705	1.2406	Q7LGC8	Carbohydrate sulfotransferase 3
1	1	2.5	48.713	1.24	Q9HA92	Radical S-adenosyl methionine domain-containing protein 1, mitochondrial
1	1	1.3	110.26	1.2393	Q8N3X1;Q8N3X1-2	Formin-binding protein 4
2	2	11.6	27.544	1.2392	Q9H825-2;Q9H825	Methyltransferase-like protein 8
1	1	4.6	32.334	1.2388	Q9Y3A4	Ribosomal RNA-processing protein 7 homolog A
1	1	0	515.6	1.238	REV__P04114	
1	1	4.2	52.965	1.2377	Q9BVS5	tRNA (adenine(58)-N(1))-methyltransferase, mitochondrial
2	2	5.9	42.188	1.2365	O60507	Protein-tyrosine sulfotransferase 1
1	1	7.8	11.133	1.235	L0R6Q1	
1	1	5.1	21.012	1.2335	P13498	Cytochrome b-245 light chain
4	2	11.5	50.377	1.2328	O43929;O43929-2;O43929-3	Origin recognition complex subunit 4
1	1	5.4	34.741	1.2284	Q8N1S5-2;Q8N1S5	Zinc transporter ZIP11
2	2	4.4	65.076	1.2284	Q9NRW7;Q9NRW7-2	Vacuolar protein sorting-associated protein 45
2	2	8.2	31.932	1.2269	Q9HAN9	Nicotinamide/nicotinic acid mononucleotide adenylyltransferase 1
1	1	6.3	16.279	1.2267	Q96HR3-2;Q96HR3	Mediator of RNA polymerase II transcription subunit 30
1	1	3.2	53.533	1.2262	Q9UBG3	Cornulin
2	2	3.2	91.079	1.2261	Q8IXB1;Q8IXB1-3;Q8IXB1-2	DnaJ homolog subfamily C member 10
1	1	8.9	18.478	1.2258	Q8TCT6-3;Q8TCT6	Signal peptide peptidase-like 3
1	1	5.1	36.566	1.2258	Q7L523	Ras-related GTP-binding protein A
3	1	5.1	87.376	1.2252	Q5MIZ7-3;Q5MIZ7-2;Q5MIZ7	Serine/threonine-protein phosphatase 4 regulatory subunit 3B
1	1	1.4	127.12	1.2224	Q6ZRV2	Protein FAM83H
1	1	0	141.54	1.2214	REV__Q9UQE7	
2	2	10.1	19.996	1.2195	Q9H6K4	Optic atrophy 3 protein
3	3	4.3	108.6	1.2193	O95259-2;O95259	Potassium voltage-gated channel subfamily H member 1
5	1	34.3	19.827	1.2191	P24844;P24844-2	Myosin regulatory light polypeptide 9
1	1	6.6	15.669	1.2167	Q12974-3;Q12974;Q93096	Protein tyrosine phosphatase type IVA 2;Protein tyrosine phosphatase type IVA 1
8	1	25.8	41.003	1.2165	sp|HLAHLA00097|	
1	1	16.3	11.069	1.2153	Q9P2W1-3;Q9P2W1-2;Q9P2W1	Homologous-pairing protein 2 homolog
3	3	2.7	184.86	1.2147	Q4KWH8-2;Q4KWH8-3;Q4KWH8-4;Q4KWH8	1-phosphatidylinositol 4,5-bisphosphate phosphodiesterase eta-1
1	1	24.4	5.2288	1.2146	P0CG35;P0CG34	Thymosin beta-15B;Thymosin beta-15A
2	2	3.6	74.977	1.2108	O43301	Heat shock 70 kDa protein 12A
1	1	1.4	155.64	1.2068	O14802	DNA-directed RNA polymerase III subunit RPC1
1	1	3	53.97	1.2051	Q9H1P3-2;Q9H1P3	Oxysterol-binding protein-related protein 2
1	1	3	29.139	1.2044	Q96G27	WW domain-binding protein 1
1	1	0	109.73	1.2007	REV__O75460	
2	2	4.9	46.432	1.1994	O75063	Glycosaminoglycan xylosylkinase
2	2	1.1	177.44	1.1967	P48681	Nestin
1	1	3.9	43.703	1.1964	O14757-2;O14757-3;O14757	Serine/threonine-protein kinase Chk1

1	1	1.8	64.195	1.1945	Q9NZQ3-5;Q9NZQ3-4;Q9NZQ3-3;Q9NZQ3-2;Q9NZQ3	NCK-interacting protein with SH3 domain
1	1	1.2	140	1.1942	Q9UK61-2;Q9UK61-3;Q9UK61-4;Q9UK61	Protein FAM208A
2	1	15.3	14.806	1.1941	O95626	Acidic leucine-rich nuclear phosphoprotein 32 family member D
2	2	0.5	596.48	1.1931	Q63HN8-4;Q63HN8;Q63HN8-6	E3 ubiquitin-protein ligase RNF213
1	1	0.8	237.67	1.1916	Q5JSL3	Dedicator of cytokinesis protein 11
2	2	4	68.297	1.1873	Q9H9F9	Actin-related protein 5
1	1	2.4	84.099	1.1861	Q86VM9-2;Q86VM9	Zinc finger CCCH domain-containing protein 18
1	1	5.3	26.839	1.1858	Q96BI3-2;Q96BI3	Gamma-secretase subunit APH-1A
2	2	2	166.46	1.1821	Q9BXW9-1;Q9BXW9;Q9BXW9-3	Fanconi anemia group D2 protein
1	1	1.1	113.76	1.1813	Q14997-3;Q14997-2;Q14997	Proteasome activator complex subunit 4
1	1	0.7	162.12	1.1795	O94910-2;O94910	Latrophilin-1
2	2	1.6	200.54	1.1769	Q8IWV8;Q8IWV8-4;Q8IWV8-2	E3 ubiquitin-protein ligase UBR2
1	1	10.4	22.144	1.1766	sp|Q2KIS7|;CON__Q2KIS7;P05452	Tetranectin
2	2	5.6	62.717	1.1757	Q8WZA9	Immunity-related GTPase family Q protein
2	2	6.7	60.524	1.1733	Q9Y6F7;Q9Y6F8;Q9Y6F8-2	Testis-specific chromodomain protein Y 2;Testis-specific chromodomain protein Y 1
2	2	0	578.27	1.1723	REV__O75445-3;REV__O75445-2;REV__O75445	
1	1	12	11.318	1.1703	Q6P1Q0-5;Q6P1Q0-4;Q6P1Q0-2;Q6P1Q0;Q6P1Q0-7	LETM1 domain-containing protein 1
1	1	17.8	5.2159	1.1683	P84101-4;P84101	Small EDRK-rich factor 2
1	1	1	160.6	1.1657	O60244	Mediator of RNA polymerase II transcription subunit 14
1	1	4.3	36.231	1.1635	Q96NB2	Sideroflexin-2
1	1	1.6	75.926	1.1631	Q96RN5-3;Q96RN5-2;Q96RN5	Mediator of RNA polymerase II transcription subunit 15
2	2	1.3	290.38	1.1599	O15078;O15078-2	Centrosomal protein of 290 kDa
1	1	20.6	7.1432	1.1595	Q9BTM9-3;Q9BTM9;Q9BTM9-2	Ubiquitin-related modifier 1
1	1	1.7	74.494	1.1585	O75330-4;O75330-2;O75330;O75330-3	Hyaluronan mediated motility receptor
2	2	7.6	39.563	1.1581	Q5T7N3-2;Q5T7N3	KN motif and ankyrin repeat domain-containing protein 4
1	1	2.8	44.402	1.1562	Q9H814	Phosphorylated adapter RNA export protein
2	2	6.5	30.666	1.156	Q9NRA2-2;Q9NRA2	Sialin
2	2	2.7	87.81	1.1548	Q96CN9	GRIP and coiled-coil domain-containing protein 1
1	1	10.6	16.661	1.1521	Q9Y6G3	39S ribosomal protein L42, mitochondrial
2	1	12.7	23.318	1.1519	O15551	Claudin-3
4	2	6.6	82.564	1.1512	Q9BQ39	ATP-dependent RNA helicase DDX50
1	1	1.6	82.303	1.1502	Q8TB52	F-box only protein 30
1	1	11	17.177	1.1468	Q8IVP5	FUN14 domain-containing protein 1
1	1	4.3	39.8	1.1465	Q969V5	Mitochondrial ubiquitin ligase activator of NFKB 1
2	2	10	26.099	1.1443	O00165-5;O00165-3;O00165;O00165-2;O00165-4	HCLS1-associated protein X-1
1	1	6.7	22.696	1.1422	Q9H008-2;Q9H008	Phospholysine phosphohistidine inorganic pyrophosphate phosphatase
3	3	8	55.568	1.1411	O75052-3;O75052	Carboxyl-terminal PDZ ligand of neuronal nitric oxide synthase protein
1	1	4.3	26.439	1.1406	Q53H96-2;Q53H96	Pyrroline-5-carboxylate reductase 3
1	1	7.3	23.862	1.14	Q8IZ16	Uncharacterized protein C7orf61
1	1	3.2	45.524	1.1388	O75530-3;O75530;O75530-2	Polycomb protein EED
1	1	3.7	44.195	1.1375	Q7L1V2-2;Q7L1V2	Vacuolar fusion protein MON1 homolog B
1	1	8.8	15.689	1.1353	Q8WUK0-2;Q8WUK0-3;Q8WUK0	Phosphatidylglycerophosphatase and protein-tyrosine phosphatase 1
2	2	4.9	57.034	1.1343	Q9NUJ3	T-complex protein 11-like protein 1
1	1	2.6	55.539	1.1324	P11362-13;P11362-4;P11362;P11362-19;P11362-7;P11362-2;P11362-20;P11362-3;P11362-14;P11362-12;P11362-8;P11362-5;P11362-6;P11362-9;P11362-10;P11362-11;P11362-21	Fibroblast growth factor receptor 1
1	1	1.9	107.38	1.131	Q86UU1-3;Q86UU1-2;Q86UU1	Pleckstrin homology-like domain family B member 1

1	1	6	42.689	1.1309	Q8NAN2-2;Q8NAN2	Protein FAM73A
1	1	2.3	75.665	1.1266	Q96FV9	THO complex subunit 1
1	1	5.4	23.743	1.1256	Q6I9Y2	THO complex subunit 7 homolog
2	2	24.1	9.8602	1.1251	P33552	Cyclin-dependent kinases regulatory subunit 2
1	1	2.3	59.903	1.1245	O00141-2	
1	1	2.8	73.912	1.1227	O14777	Kinetochore protein NDC80 homolog
1	1	3.6	36.223	1.1207	P16260	Graves disease carrier protein
1	1	11.9	16.799	1.1194	Q9P242-3;Q9P242	Neuronal tyrosine-phosphorylated phosphoinositide-3-kinase adapter 2
1	1	1.2	146.93	1.1182	A1KZ92-2;A1KZ92	Peroxidasin-like protein
1	1	1.3	109.79	1.1169	Q66K74-2;Q66K74	Microtubule-associated protein 1S;MAP1S heavy chain;MAP1S light chain
1	1	1.1	128.4	1.1166	Q9P2R3;Q9P2R3-2;Q9P2R3-4	Rabankyrin-5
1	1	2.8	62.027	1.1141	Q5F1R6;Q5F1R6-3;Q5F1R6-2	DnaJ homolog subfamily C member 21
2	2	10.9	20.086	1.1137	Q5J8M3;Q5J8M3-2	ER membrane protein complex subunit 4
3	1	7.6	45.413	1.1129	Q9HCP0-2;Q9HCP0	Casein kinase I isoform gamma-1
1	1	0	64.195	1.1128	REV__Q9NZQ3-5;REV__Q9NZQ3-4;REV__Q9NZQ3-3;REV__Q9NZQ3-2;REV__Q9NZQ3	
1	1	0	265.4	1.1127	REV__Q04721	
1	1	5.4	23.589	1.1112	Q9NY26-2;Q9NY26	Zinc transporter ZIP1
1	1	4.7	40.51	1.1111	Q96S82	Ubiquitin-like protein 7
1	1	3.3	37.928	1.1107	Q9NNX1-3;Q9NNX1-2;Q9NNX1	Tuftelin
2	2	0	226.53	1.1096	REV__P35579;REV__P35579-2	
1	1	5.7	43.395	1.1088	Q9HC52	Chromobox protein homolog 8
1	1	6.4	17.776	1.1086	P11441	Ubiquitin-like protein 4A
2	2	14.2	12.615	1.1069	O14521-2;O14521;O14521-4	Succinate dehydrogenase [ubiquinone] cytochrome b small subunit, mitochondrial
1	1	6.6	29.341	1.1044	Q9NTM9	Copper homeostasis protein cutC homolog
1	1	10.1	10.741	1.1033	Q0VGL1	Ragulator complex protein LAMTOR4;Ragulator complex protein LAMTOR4, N-terminally processed
1	1	10.4	15.199	1.1023	O00488	Zinc finger protein 593
1	1	1.2	131.29	1.1017	Q86YC2	Partner and localizer of BRCA2
2	2	4.1	44.96	1.1016	P13473;P13473-2;P13473-3	Lysosome-associated membrane glycoprotein 2
2	2	0	55.873	1.1015	REV__Q96A33;REV__Q96A33-2	
1	1	3.6	34.947	1.1005	Q96IW7	Vesicle-trafficking protein SEC22a
16	1	28.2	85.251	1.1003	P27816-5	Microtubule-associated protein 4
2	2	0	110.97	1.0979	REV__Q01954	
2	2	1.1	250.2	1.0973	Q14667-2;Q14667;Q14667-4;Q14667-3	Protein KIAA0100
1	1	1.8	66.335	1.0918	Q0VD83-2	
1	1	1.5	80.472	1.0916	Q96RQ3	Methylcrotonoyl-CoA carboxylase subunit alpha, mitochondrial
1	1	2.2	114.16	1.0896	P57737-3;Q9Y3D7	Mitochondrial import inner membrane translocase subunit TIM16
1	1	2.1	79.548	1.0876	Q3YEC7;Q3YEC7-2	Rab-like protein 6
1	1	1.3	88.183	1.0875	Q92805	Golgin subfamily A member 1
1	1	3.6	36.923	1.0856	P52799	Ephrin-B2
2	2	2.1	165.53	1.0848	Q5JSZ5-5;Q5JSZ5	Protein PRRC2B
1	1	7.8	28.306	1.0843	P0DPD7;P0DPD8	
1	1	1.6	59.076	1.082	O43300;Q86VH4-2;Q86VH4	Leucine-rich repeat transmembrane neuronal protein 2;Leucine-rich repeat transmembrane neuronal protein 4
1	1	1.1	80.409	1.082	Q15746-9;Q15746-11;Q15746-7;Q15746-4;Q15746-5;Q15746-2;Q15746-3;Q15746-6;Q15746	Myosin light chain kinase, smooth muscle;Myosin light chain kinase, smooth muscle, deglutamylated form
1	1	6	23.382	1.0819	Q8TCD5	5(3)-deoxyribonucleotidase, cytosolic type
1	1	0.7	180.43	1.0819	O75581	Low-density lipoprotein receptor-related protein 6
1	1	0	305.28	1.0819	REV__Q12802-4;REV__Q12802;REV__Q12802-2	
1	1	2.9	51.999	1.0817	Q86TW2-3;Q86TW2-2;Q86TW2	Uncharacterized aarF domain-containing protein kinase 1

1	1	0.7	330.63	1.0812	Q68DQ2	Very large A-kinase anchor protein
1	1	7.9	21.688	1.0803	Q9NXA8-4;Q9NXA8-2;Q9NXA8	NAD-dependent protein deacylase sirtuin-5, mitochondrial
1	1	0.7	132.67	1.0776	Q9H7Z3	Protein NRDE2 homolog
1	1	5.1	22.372	1.0774	Q9NWX5-2;Q9NWX5	Ankyrin repeat and SOCS box protein 6
1	1	5.9	28.912	1.0773	Q9GZU8	Protein FAM192A
2	2	4.8	74.024	1.0764	Q8IUZ0-3;Q8IUZ0-4;Q8IUZ0;Q8IUZ0-2	Leucine-rich repeat-containing protein 49
1	1	8.8	20.305	1.0752	Q567V2-2;Q567V2	Mpv17-like protein 2
2	2	7.8	29.48	1.0746	O00584;O00584-2	Ribonuclease T2
1	1	7.3	20.482	1.0706	Q9UNL4-8;Q9UNL4-3;Q8WYH8-2;Q8WYH8;Q9UNL4-4;Q9UNL4-7;Q9UNL4-5;Q9UNL4-6;Q9UNL4-2;Q9UNL4	Inhibitor of growth protein 4;Inhibitor of growth protein 5
2	2	0.8	343.9	1.0688	P24043	Laminin subunit alpha-2
2	2	1.3	159.09	1.0687	A4FU69-3;A4FU69;A4FU69-6;A4FU69-5;A4FU69-2;A4FU69-4	EF-hand calcium-binding domain-containing protein 5
1	1	14.5	13.693	1.0676	O95059	Ribonuclease P protein subunit p14
14	1	55.3	31.548	1.0674	P04406-2	Glyceraldehyde-3-phosphate dehydrogenase
1	1	14.1	7.0662	1.0673	Q9Y2S6	Translation machinery-associated protein 7
2	2	8.3	46.422	1.0667	Q86XQ3	Cation channel sperm-associated protein 3
1	1	3.7	42.319	1.0655	Q16828	Dual specificity protein phosphatase 6
14	1	47.8	37.498	1.0645	Q99832-2	T-complex protein 1 subunit eta
2	2	2.3	170.56	1.0645	P41229-3;P41229-2;P41229-5;P41229;Q9BY66-2;Q9BY66;Q9BY66-3	Lysine-specific demethylase 5C;Lysine-specific demethylase 5D
1	1	2.5	72.525	1.0633	Q9UJX4-3;Q9UJX4	Anaphase-promoting complex subunit 5
1	1	0	183.15	1.0628	REV__Q05707-3;REV__Q05707-2;REV__Q05707	
2	2	5.6	59.152	1.0625	Q15291;Q15291-2	Retinoblastoma-binding protein 5
2	2	0.3	868.47	1.0624	Q5VST9;Q5VST9-2;Q5VST9-6;Q5VST9-3	Obscurin
1	1	4.1	47.091	1.0618	Q9H553	Alpha-1,3/1,6-mannosyltransferase ALG2
1	1	4.7	26.736	1.06	P49427	Ubiquitin-conjugating enzyme E2 R1
1	1	3.8	29.213	1.0598	Q9Y483-2;Q9Y483-3;Q9Y483-4;Q9Y483	Metal-response element-binding transcription factor 2
1	1	2.2	50.91	1.0598	Q8WUX9	Charged multivesicular body protein 7
2	2	2.2	107.33	1.0587	Q9Y4C8	Probable RNA-binding protein 19
2	2	3.2	64.002	1.0578	Q8WUA4-2;Q8WUA4	General transcription factor 3C polypeptide 2
2	2	13.2	21.054	1.0571	O14683	Tumor protein p53-inducible protein 11
2	2	5.4	48.699	1.0571	Q8NFZ5	TNFAIP3-interacting protein 2
2	2	3.6	56.811	1.0569	P09923;P10696;P05187	Intestinal-type alkaline phosphatase;Alkaline phosphatase, placental-like;Alkaline phosphatase, placental type
2	2	1.5	119.7	1.0558	Q8IZL8	Proline-, glutamic acid- and leucine-rich protein 1
1	1	0.5	191.42	1.0544	Q68CP9-3;Q68CP9	AT-rich interactive domain-containing protein 2
3	3	3.8	115.56	1.0541	Q8TD43-2;Q8TD43-3;Q8TD43	Transient receptor potential cation channel subfamily M member 4
1	1	0	60.029	1.0515	REV__O76050-2;REV__O76050	
1	1	0	204.92	1.0508	REV__P21675-6;REV__P21675-7;REV__P21675-3;REV__P21675-5;REV__P21675-9;REV__P21675;REV__P21675-2;REV__P21675-12;REV__P21675-4	
2	2	0	71.337	1.0506	REV__Q9Y6Q3-3;REV__Q9Y6Q3;REV__Q9Y6Q3-2	
1	1	2.8	51.839	1.0504	Q8N4S9-2;Q8N4S9-3;Q8N4S9	MARVEL domain-containing protein 2
1	1	4.4	32.704	1.0491	Q96JZ2-2	
1	1	0	92.02	1.0487	REV__P04035-2;REV__P04035;REV__P04035-3	
1	1	1.4	73.475	1.0477	P24386	Rab proteins geranylgeranyltransferase component A 1
2	2	3.1	97.344	1.0466	Q96DR7;Q96DR7-4;Q96DR7-3	Rho guanine nucleotide exchange factor 26
1	1	10.9	11.216	1.046	Q9P021	Cysteine-rich PDZ-binding protein

2	2	7.4	56.294	1.046	Q96M29	Tektin-5
1	1	1.5	81.498	1.046	Q14494-2;Q14494	Nuclear factor erythroid 2-related factor 1
1	1	5.4	27.941	1.0458	Q9P0P8	Uncharacterized protein C6orf203
1	1	0.8	125.3	1.0447	Q8IWB9;Q8IWB9-2	Testis-expressed sequence 2 protein
1	1	2.3	51.172	1.0428	Q9H993	Protein-glutamate O-methyltransferase
1	1	2.3	59.204	1.0388	Q9H6X2-5;Q9H6X2	Anthrax toxin receptor 1
2	2	0	224.13	1.0381	REV__Q9Y566-3;REV__Q9Y566	
3	1	28.2	15.257	1.0379	P69905	Hemoglobin subunit alpha
1	1	5.3	17.102	1.0368	O14684	Prostaglandin E synthase
2	2	0.6	513.25	1.0366	O75592-2;O75592	E3 ubiquitin-protein ligase MYCBP2
1	1	2.3	40.1	1.0353	Q9UH03-2;Q9UH03	Neuronal-specific septin-3
1	1	10.1	9.0788	1.035	P61165	Transmembrane protein 258
1	1	10.9	10.809	1.0344	A6NNX1	RIIa domain-containing protein 1
1	1	6.5	24.625	1.0335	Q9NUM3-3;Q9NUM3-2;Q9NUM3	Zinc transporter ZIP9
2	2	2.7	92.22	1.0319	Q9UBN4-6;Q9UBN4-4;Q9UBN4-3;Q9UBN4-2;Q9UL62;Q9UBN4;Q9UBN4-5	Short transient receptor potential channel 4;Short transient receptor potential channel 5
2	2	3.5	93.784	1.0317	Q8IWC1-2;Q8IWC1-3;Q8IWC1-4;Q8IWC1	MAP7 domain-containing protein 3
1	1	5.3	25.607	1.0307	Q92913-5	
1	1	6	22.133	1.0295	sp|FA35-18_XP002713367|;P05976-2;P05976;P08590	Myosin light chain 1/3, skeletal muscle isoform;Myosin light chain 3
1	1	33.3	2.8092	1.0265	Q5TGZ0-2;Q5TGZ0	MICOS complex subunit MIC10
1	1	8.4	15.794	1.0261	Q93062-4;Q93062-5;Q93062;Q93062-2;Q93062-3	RNA-binding protein with multiple splicing
2	2	6.9	65.26	1.0246	Q8WXI9	Transcriptional repressor p66-beta
1	1	3.5	37.63	1.0246	O14772-2;O14772	Fucose-1-phosphate guanylyltransferase
1	1	0	158.21	1.0235	REV__Q76I76	
1	1	6.2	19.258	1.0226	O60831	PRA1 family protein 2
1	1	3.4	37.335	1.0226	Q9Y388	RNA-binding motif protein, X-linked 2
1	1	2.2	115.2	1.022	Q13873	Bone morphogenetic protein receptor type-2
2	2	9.2	31.54	1.0214	P22676	Calretinin
12	1	42.9	38.737	1.016	P42167-2	Lamina-associated polypeptide 2, isoforms beta/gamma;Thymopoietin;Thymopentin
3	3	0	95.877	1.0152	REV__Q14721	
1	1	0	110.28	1.0145	REV__Q8N9B5-2;REV__Q8N9B5	
4	1	22.1	23.461	1.0138	Q9NRW1;Q9NRW1-2	Ras-related protein Rab-6B
1	1	4.7	40.929	1.0134	O95551;O95551-2	Tyrosyl-DNA phosphodiesterase 2
1	1	3.4	45.563	1.0122	O00499-9;O00499-10;O00499-7;O00499-8;O00499-4;O00499-6;O00499-11;O00499-3;O00499-2;O00499-5;O00499	Myc box-dependent-interacting protein 1
1	1	0.9	168	1.0122	Q2KHR3-2;Q2KHR3	Glutamine and serine-rich protein 1
2	2	5.7	109.8	1.0121	A0A0J9YWL9	
1	1	2.9	72.073	1.012	Q9P0L2-2;P27448-4;Q7KZI7;Q7KZI7-8;Q7KZI7-11;P27448-7;Q9P0L2-3;Q7KZI7-6;P27448;P27448-2;Q96L34;Q7KZI7-3;Q7KZI7-14;Q7KZI7-12;Q7KZI7-10;Q7KZI7-9;P27448-3;Q7KZI7-4;Q7KZI7-16;Q7KZI7-5;P27448-6;Q7KZI7-15;Q7KZI7-2;Q7KZI7-7;Q7KZI7-13;Q96L34-2;Q9P0L2	Serine/threonine-protein kinase MARK1;MAP/microtubule affinity-regulating kinase 3;Serine/threonine-protein kinase MARK2;MAP/microtubule affinity-regulating kinase 4
8	1	26.4	43.236	1.0119	O96019-2	Actin-like protein 6A
1	1	11.6	18.322	1.0106	Q9UK58-5;Q9UK58-4;Q9UK58-6;Q9UK58	Cyclin-L1
1	1	9.5	14.289	1.0082	Q9Y3E2	BolA-like protein 1
2	2	14.8	15.136	1.0075	P56556	NADH dehydrogenase [ubiquinone] 1 alpha subcomplex subunit 6

1	1	7.6	26.702	1.0062	Q76EJ3-2;Q76EJ3	UDP-N-acetylglucosamine/UDP-glucose/GDP-mannose transporter
1	1	2.6	52.781	1.0061	Q8IYT4-2;Q8IYT4	Katanin p60 ATPase-containing subunit A-like 2
1	1	3	61.007	1.0052	P43004-3;P43004	Excitatory amino acid transporter 2
9	1	26.9	46.424	1.0037	Q9Y383-2	Putative RNA-binding protein Luc7-like 2
2	2	8.5	44.885	1.0016	Q96KP6-3;Q96KP6-2;Q96KP6	TNFAIP3-interacting protein 3
1	1	3.3	45.591	0.99994	Q9BSW2;Q9BSW2-2	EF-hand calcium-binding domain-containing protein 4B
1	1	0	84.092	0.99733	REV__O60733-2;REV__O60733	
1	1	3.9	31.834	0.99542	P78345	Ribonuclease P protein subunit p38
2	2	4.9	76.763	0.99302	Q8IZW8	Tensin-4
1	1	8.2	21.09	0.99236	Q9NX08	COMM domain-containing protein 8
1	1	3.1	50.316	0.99202	P04062-4;P04062-2;P04062	Glucosylceramidase
1	1	7.4	34.769	0.99184	Q3SYG4-5;Q3SYG4-6;Q3SYG4-2;Q3SYG4-4;Q3SYG4-7;Q3SYG4-3;Q3SYG4	Protein PTHB1
1	1	2.9	42.871	0.99113	Q5VW32-2;Q5VW32	BRO1 domain-containing protein BROX
1	1	3.9	44.486	0.98984	Q86YV6-2;Q86YV6	Myosin light chain kinase family member 4
1	1	9.4	14.542	0.9886	O43414-3;O43414-2;O43414	ERI1 exoribonuclease 3
2	2	10.5	21.848	0.98744	Q5VYY1	Ankyrin repeat domain-containing protein 22
1	1	15.8	8.4977	0.98602	Q9Y5V0	Zinc finger protein 706
1	1	4.1	63.553	0.98505	Q14332	Frizzled-2
3	1	63.3	5.7407	-2	P56134-4;P56134-2	ATP synthase subunit f, mitochondrial
19	1	58.1	44.965	-2	Q8NC51;Q8NC51-2	Plasminogen activator inhibitor 1 RNA-binding protein
9	1	56.8	19.438	-2	P35613-3	Basigin
30	1	55.1	65.134	-2	P02545-2	Prelamin-A/C;Lamin-A/C
6	1	46	13.908	-2	Q8N257	Histone H2B type 3-B
27	1	45.6	68.08	-2	P31948-2	Stress-induced-phosphoprotein 1
12	1	40.9	37.277	-2	P16070-18	CD44 antigen
58	1	39.6	202.16	-2	P16144	Integrin beta-4
3	1	36.9	11.842	-2	Q13404-6	Ubiquitin-conjugating enzyme E2 variant 1
17	1	32.9	71.214	-2	O43390-2;O43390-4	Heterogeneous nuclear ribonucleoprotein R
3	1	23.7	28.814	-2	Q6NZ63;Q6NZ63-2	STEAP family member 1B
15	1	22.2	60.044	-2	CON__P02538;CON__P48668;P02538;P48668;sp|P02538|;sp|P48668|;CON__P04259;sp|P04259|	Keratin, type II cytoskeletal 6A;Keratin, type II cytoskeletal 6C
1	1	21.5	8.7931	-2	Q96PI1	Small proline-rich protein 4
1	1	21	9.3135	-2	Q14587-3	Zinc finger protein 268
1	1	20.2	10.354	-2	Q96PG1-2	
3	1	18.9	18.018	-2	Q6DRA6;Q6DN03	Putative histone H2B type 2-D;Putative histone H2B type 2-C
1	1	18.3	12.013	-2	O96001-2;O96001	Protein phosphatase 1 regulatory subunit 17
3	1	14.6	23.122	-2	sp|092267|;CON__REFSEQ:XP_092267	
3	1	12.7	30.216	-2	P51809-2	Vesicle-associated membrane protein 7
1	1	12.6	12.041	-2	Q5JRK9	Putative G antigen family E member 3
1	1	11.3	11.089	-2	Q7Z4B0-2;Q7Z4B0	Putative uncharacterized protein encoded by LINC00305
1	1	10.7	11.84	-2	Q9NS25	Sperm protein associated with the nucleus on the X chromosome B/F
17	2	9.4	223.57	-2	P35749-4;P35749-3;P35749;P35749-2	Myosin-11
1	1	9.3	30.372	-2	Q53HI1	Protein unc-50 homolog
1	1	9.2	14.622	-2	P24522-2;P24522	Growth arrest and DNA damage-inducible protein GADD45 alpha
1	1	8.9	15.737	-2	Q9Y587-3;Q9Y587;Q9Y587-4;Q9Y587-2	AP-4 complex subunit sigma-1
1	1	8.5	17.783	-2	Q6ZST4	Lipocalin-like 1 protein
1	1	7.9	21.989	-2	P32881	Interferon alpha-8
1	1	7.9	16.479	-2	Q15528-2;Q15528	Mediator of RNA polymerase II transcription subunit 22
1	1	7.9	25.089	-2	Q96PB7-4;Q96PB7-2;Q96PB7-3;Q96PB7	Noelin-3

1	1	7.8	17.446	-2	Q15131-6;Q15131-5;Q15131	Cyclin-dependent kinase 10
1	1	7.8	26.341	-2	Q9H2S1-2;Q9H2S1	Small conductance calcium-activated potassium channel protein 2
1	1	7.6	31.959	-2	P13942-9	
37	0	7.3	838.3	-2	Q9UPN3;Q9UPN3-4	Microtubule-actin cross-linking factor 1, isoforms 1/2/3/5
1	1	7.2	36.468	-2	P06734	Low affinity immunoglobulin epsilon Fc receptor;Low affinity immunoglobulin epsilon Fc receptor membrane-bound form;Low affinity immunoglobulin epsilon Fc receptor soluble form
1	1	7	44.889	-2	Q6ZMG9;Q6ZMG9-2	Ceramide synthase 6
1	1	6.8	34.714	-2	Q8NGT9	Olfactory receptor 2A1/2A42
1	1	6.6	21.142	-2	Q9BXU8	Ferritin heavy polypeptide-like 17
1	1	6.5	18.974	-2	sp|P02668|;CON__P02668	
1	1	6.5	37.072	-2	Q6DHV5-1;Q6DHV5-2;Q6DHV5	Protein CC2D2B
5	1	6.4	117.39	-2	P0CG39	POTE ankyrin domain family member J
1	1	6.4	32.953	-2	P56705-2;P56705	Protein Wnt-4
1	1	6	22.109	-2	Q7LBR1	Charged multivesicular body protein 1b
1	1	5.6	28.994	-2	Q16623-3;Q16623-2;Q16623	Syntaxin-1A
1	1	5.6	24.713	-2	Q9P0W2-2;Q9P0W2-3;Q9P0W2	SWI/SNF-related matrix-associated actin-dependent regulator of chromatin subfamily E member 1-related
1	1	5.4	26.397	-2	Q8N5S1-2;Q8N5S1	Solute carrier family 25 member 41
1	1	5.2	22.1	-2	Q6ZTC4	Putative uncharacterized protein FLJ44790
1	1	5.2	30.386	-2	Q96EV8-3;Q96EV8-2;Q96EV8	Dysbindin
1	1	5.1	32.206	-2	Q5T0U0;Q5T0U0-2	Coiled-coil domain-containing protein 122
1	1	4.8	22.49	-2	Q8WW32	High mobility group protein B4
1	1	4.8	38.501	-2	Q9HCH5-4;Q9HCH5-9;Q9HCH5-2;Q9HCH5-15;Q9HCH5-12;Q9HCH5-14;Q9HCH5-6;Q9HCH5;Q9HCH5-13;Q9HCH5-11;Q9HCH5-7;Q9HCH5-8	Synaptotagmin-like protein 2
1	1	4.7	35.933	-2	Q8N4F4	Solute carrier family 22 member 24
1	1	4.6	43.556	-2	O15204-2;O15204	ADAM DEC1
1	1	4.6	49.192	-2	Q9H0U9	Testis-specific Y-encoded-like protein 1
1	1	4.6	39.035	-2	Q9P2A4	ABI gene family member 3
3	1	4.5	83.404	-2	Q15700-3;Q15700;Q15700-4;Q15700-2	Disks large homolog 2
1	1	4.3	60.497	-2	Q96NG8	Zinc finger protein 582
1	1	4.3	23.721	-2	Q9BRV8;Q9BRV8-2	Suppressor of IKBKE 1
1	1	4.3	42.692	-2	Q9C0C6	CLOCK-interacting pacemaker
1	1	4.1	25.494	-2	Q6ZWH5-3;Q6ZWH5-4;Q6ZWH5-2;Q6ZWH5	Serine/threonine-protein kinase Nek10
3	1	3.9	125.6	-2	sp|FA50-20a|	
1	1	3.8	70.224	-2	Q7Z353-2;Q7Z353	Highly divergent homeobox
1	1	3.7	47.583	-2	P20749	B-cell lymphoma 3 protein
1	1	3.7	39.699	-2	Q8N0S2	Synaptonemal complex central element protein 1
4	1	3.6	115.51	-2	P54707;P54707-2	Potassium-transporting ATPase alpha chain 2
1	1	3.5	70.897	-2	Q99759;Q99759-2	Mitogen-activated protein kinase kinase kinase 3
1	1	3.4	39.912	-2	Q13702-2;Q13702	43 kDa receptor-associated protein of the synapse
1	1	3.4	62.126	-2	Q96ST8-2	
2	2	3.3	85.242	-2	Q8NHX9	Two pore calcium channel protein 2
1	1	3.2	60.944	-2	O00522-2;O00522-3;O00522	Krev interaction trapped protein 1
1	1	3.1	37.796	-2	Q8N9M1-2;Q8N9M1	Uncharacterized protein C19orf47
1	1	3.1	54.74	-2	Q8NFT6-3	
1	1	3.1	48.569	-2	Q9ULG6-3;Q9ULG6-5	
1	1	3	62.466	-2	P35612-2;P35612-8;P35612-9;P35612-4;P35612-3;P35612	Beta-adducin
1	1	3	37.152	-2	P52655-2	
1	1	3	52.813	-2	Q01740-2;Q01740	Dimethylaniline monooxygenase [N-oxide-forming] 1

1	1	3	61.986	-2	Q8ND07	Basal body-orientation factor 1
2	2	2.9	99.924	-2	Q4L180-5;Q4L180-3;Q4L180-7;Q4L180-6;Q4L180-2;Q4L180	Filamin A-interacting protein 1-like
2	2	2.9	109.3	-2	Q7Z7B0-3;Q7Z7B0-2;Q7Z7B0	Filamin-A-interacting protein 1
1	1	2.9	34.765	-2	Q8NHC8	Olfactory receptor 2T6
1	1	2.9	62.72	-2	Q99741	Cell division control protein 6 homolog
1	1	2.8	61.57	-2	O94929-2;O94929-3;O94929	Actin-binding LIM protein 3
1	1	2.8	56.867	-2	Q9P2K6	Kelch-like protein 42
1	1	2.8	58.282	-2	Q9UBE8	Serine/threonine-protein kinase NLK
1	1	2.7	42.162	-2	Q8N609	Translocating chain-associated membrane protein 1-like 1
1	1	2.7	68.26	-2	Q96NJ3	Zinc finger protein 285
1	1	2.6	60.29	-2	Q6PI77	Protein BHLHb9
1	1	2.6	57.691	-2	Q9BYG8	Gasdermin-C
1	1	2.5	60.601	-2	P04150-7;P04150-5	
1	1	2.5	70.977	-2	Q05D60	Deuterosome protein 1
1	1	2.5	73.98	-2	Q9H4Q3	PR domain zinc finger protein 13
1	1	2.4	67.807	-2	P0C7X5	Zinc finger protein 806
1	1	2.4	48.654	-2	Q8N9Z0-2;Q8N9Z0	Zinc finger protein 610
1	1	2.4	74.877	-2	Q9Y222-5;Q9Y222	Cyclin-D-binding Myb-like transcription factor 1
1	1	2.3	34.365	-2	Q5JVS0-2;Q5JVS0	Intracellular hyaluronan-binding protein 4
1	1	2.3	92.06	-2	Q6PFW1-6;Q6PFW1-5;Q6PFW1-7;Q6PFW1-4;Q6PFW1-3;Q6PFW1-2;Q6PFW1	Inositol hexakisphosphate and diphosphoinositol-pentakisphosphate kinase 1
1	1	2.3	111.55	-2	Q96JN2-2;Q96JN2;Q96JN2-4	Coiled-coil domain-containing protein 136
3	2	2.2	150.19	-2	Q5JR59	Microtubule-associated tumor suppressor candidate 2
2	2	2.2	135.84	-2	Q8NDV3-2;Q8NDV3-3;Q8NDV3	Structural maintenance of chromosomes protein 1B
2	2	2.2	160.77	-2	Q8NEY1-5;Q8NEY1-7;Q8NEY1-4;Q8NEY1-2;Q8NEY1-3;Q8NEY1;Q8NEY1-6	Neuron navigator 1
1	1	2.2	100.63	-2	P0C881	Radial spoke head 10 homolog B
1	1	2.2	52.221	-2	Q8TAL5	Uncharacterized protein C9orf43
1	1	2.1	91.652	-2	O15169-2;O15169	Axin-1
1	1	2	51.92	-2	Q56UN5-7;Q56UN5-4;Q56UN5-5;Q56UN5-3;Q56UN5	Mitogen-activated protein kinase kinase kinase 19
1	1	2	76.396	-2	Q5JTW2;Q5JTW2-5	Centrosomal protein of 78 kDa
1	1	2	77.224	-2	Q9Y2L9-2;Q9Y2L9;Q9Y2L9-3	Leucine-rich repeat and calponin homology domain-containing protein 1
1	1	1.9	107.4	-2	Q6W2J9-3;Q6W2J9-4;Q6W2J9-2;Q6W2J9	BCL-6 corepressor
1	1	1.9	138.18	-2	Q9UBZ9-2;Q9UBZ9	DNA repair protein REV1
1	1	1.8	56.438	-2	P80404	4-aminobutyrate aminotransferase, mitochondrial
1	1	1.8	107.7	-2	Q6PIJ6-3;Q6PIJ6-2;Q6PIJ6	F-box only protein 38
1	1	1.8	91.012	-2	Q8IYM0-2;Q8IYM0	Protein FAM186B
1	1	1.8	93.012	-2	Q92831	Histone acetyltransferase KAT2B
1	1	1.8	112.88	-2	Q9P232	Contactin-3
1	1	1.8	58.836	-2	Q9UBI9	Headcase protein homolog
1	1	1.8	82.69	-2	Q9ULU4-4	
1	1	1.7	69.236	-2	O60765	Zinc finger protein 354A
1	1	1.7	89.196	-2	Q8IW93	Rho guanine nucleotide exchange factor 19
1	1	1.7	114.39	-2	Q9H2E6;Q9H2E6-2	Semaphorin-6A
1	1	1.7	46.916	-2	sp|Nop5|	
1	1	1.6	94.804	-2	O76074-2;O76074	cGMP-specific 3,5-cyclic phosphodiesterase
1	1	1.6	49.182	-2	O94822-2;O94822	E3 ubiquitin-protein ligase listerin
1	1	1.6	93.881	-2	Q6ZW49-2;Q6ZW49-1;Q6ZW49	PAX-interacting protein 1
1	1	1.6	76.647	-2	Q96T17-5	

1	1	1.6	111.69	-2	Q9UQ05	Potassium voltage-gated channel subfamily H member 4
1	1	1.5	91.861	-2	O43196-4;O43196;O43196-2;O43196-3	MutS protein homolog 5
2	2	1.4	281.29	-2	Q9HCE0-2;Q9HCE0	Ectopic P granules protein 5 homolog
1	1	1.4	56.682	-2	sp|Q9NSB4|;CON__Q9NSB4;Q9NSB4	Keratin, type II cuticular Hb2
1	1	1.4	81.839	-2	O15409-9	
1	1	1.4	126.75	-2	P35228-2;P35228	Nitric oxide synthase, inducible
1	1	1.4	137.87	-2	Q2KHR2-2;Q2KHR2	DNA-binding protein RFX7
1	1	1.4	144.96	-2	Q96MR6	Cilia- and flagella-associated protein 57
1	1	1.4	133.7	-2	Q9NQ66-2;Q9NQ66	1-phosphatidylinositol 4,5-bisphosphate phosphodiesterase beta-1
1	1	1.4	143.02	-2	Q9UHF7-2	
2	1	1.3	223.6	-2	Q9UKX3	Myosin-13
1	1	1.3	111.18	-2	Q14289-2;Q14289	Protein-tyrosine kinase 2-beta
2	2	1.2	173.74	-2	Q9P2K1-2;Q9P2K1	Coiled-coil and C2 domain-containing protein 2A
1	1	1.2	96.133	-2	Q4LDE5-2;Q4LDE5-4;Q4LDE5	Sushi, von Willebrand factor type A, EGF and pentraxin domain-containing protein 1
1	1	1.1	168.46	-2	P58397-3;P58397	A disintegrin and metalloproteinase with thrombospondin motifs 12
1	1	1.1	157.23	-2	Q86UL8-2;Q86UL8	Membrane-associated guanylate kinase, WW and PDZ domain-containing protein 2
1	1	1.1	103.46	-2	Q92622-2	
1	1	1.1	93.613	-2	Q9BQK8;Q9BQK8-2	Phosphatidate phosphatase LPIN3
1	1	1.1	175.96	-2	Q9HCJ0;Q9HCJ0-2	Trinucleotide repeat-containing gene 6C protein
1	1	1	104.99	-2	Q9NQS7-2;Q9NQS7	Inner centromere protein
1	1	0.9	128.61	-2	O75095-2	
1	1	0.9	119.49	-2	P0DP91;Q8N328	PiggyBac transposable element-derived protein 3
1	1	0.9	99.791	-2	Q13474-2	
1	1	0.9	177.29	-2	Q9P2S2-2;Q9P2S2	Neurexin-2
1	1	0.9	145.12	-2	Q9UQD0-4;Q9UQD0-5;Q9UQD0-2;Q9UQD0;Q9UQD0-3	Sodium channel protein type 8 subunit alpha
1	1	0.8	122.02	-2	Q92540-2;Q92540;Q92540-4	Protein SMG7
1	1	0.8	237.13	-2	Q9Y2I7	1-phosphatidylinositol 3-phosphate 5-kinase
1	1	0.8	234.95	-2	Q9Y3S1-2;Q9Y3S1-4;Q9Y3S1	Serine/threonine-protein kinase WNK2
1	1	0.8	220.62	-2	Q9Y5Y9	Sodium channel protein type 10 subunit alpha
1	1	0.7	185.64	-2	H7BZ55	Putative ciliary rootlet coiled-coil protein-like 3 protein
1	1	0.7	144.72	-2	Q9H7P9-3;Q9H7P9	Pleckstrin homology domain-containing family G member 2
1	1	0.6	315.22	-2	Q8IWI9-3;Q8IWI9	MAX gene-associated protein
1	1	0.6	249.49	-2	Q96BY6-3;Q96BY6	Dedicator of cytokinesis protein 10
1	1	0.6	337.83	-2	Q9Y485	DmX-like protein 1
2	2	0.5	305.28	-2	Q12802-4;Q12802;Q12802-2	A-kinase anchor protein 13
1	1	0.5	224.2	-2	Q15643-2;Q15643	Thyroid receptor-interacting protein 11
1	1	0.5	201.11	-2	Q8TD84-2;Q8TD84	Down syndrome cell adhesion molecule-like protein 1
1	1	0.3	270.88	-2	Q96AY4	Tetratricopeptide repeat protein 28
1	1	0.2	564.56	-2	Q92736;Q92736-2	Ryanodine receptor 2
1	1	0	77.467	-2	REV__P17035-2;REV__P17035	
1	1	0	427.73	-2	REV__Q03164-2;REV__Q03164;REV__Q03164-3	
1	1	0	143.23	-2	REV__Q14683	
1	1	0	29.329	-2	REV__Q7RTY3	
1	1	0	81.097	-2	REV__Q9H9E3-3;REV__Q9H9E3	
1	1	0	174.73	-2	REV__Q9NYU1	
1	1	0	87.253	-2	REV__Q9Y5E7	
